# Optimization of the Central α-Amino Acid in Cystobactamids to the Broad-Spectrum, Resistance-Breaking Antibiotic CN-CC-861

**DOI:** 10.1021/acs.jmedchem.4c00927

**Published:** 2024-09-20

**Authors:** Daniel Kohnhäuser, Tim Seedorf, Katarina Cirnski, Dominik Heimann, Janetta Coetzee, Sylvie Sordello, Jana Richter, Moritz Stappert, Jean-Francois Sabuco, David Corbett, Eric Bacqué, Katharina Rox, Jennifer Herrmann, Aurelie Vassort, Rolf Müller, Andreas Kirschning, Mark Brönstrup

**Affiliations:** † Department of Chemical Biology, Helmholtz Centre for Infection Research, Inhoffenstraße 7, 38124 Braunschweig, Germany; ‡ Institute of Organic Chemistry and Biomolecular Drug Research Centre (BMWZ), Leibniz University Hannover, Schneiderberg 1B, 30167 Hannover, Germany; § Microbial Natural Products, Helmholtz Institute for Pharmaceutical Research Saarland (HIPS), Helmholtz Centre for Infection Research (HZI), Department of Pharmacy at Saarland University, Campus Building E8.1, 66123 Saarbrücken, Germany; ∥ German Center for Infection Research (DZIF), Site Hannover-Braunschweig, Inhoffenstraße 7, 38124 Braunschweig, Germany; ⊥ Evotec ID, 1541 Avenue Marcel Merieux, 69289 Marcy l’Etoile, France; # Uppsala Biomedical Center (BMC), Uppsala University, Husargatan 3, 752 37 Uppsala, Sweden

## Abstract

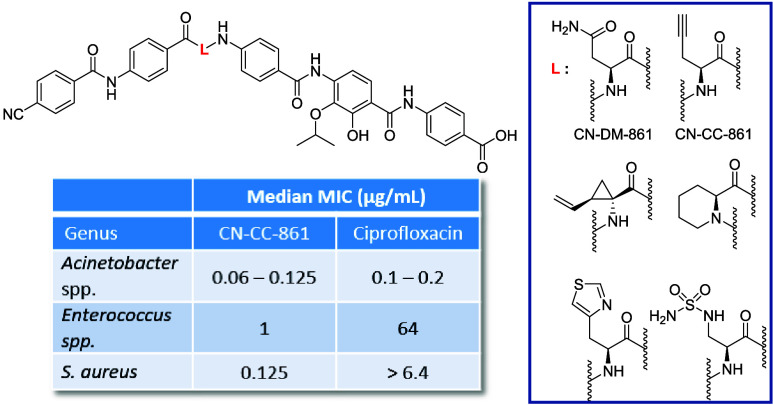

Cystobactamids have a unique oligoarylamide structure and exhibit broad-spectrum activity against Gram-negative and Gram-positive bacteria. In this study, the central α-amino acid of the cystobactamid scaffold was modified to address the relevance of stereochemistry, hydrogen bonding and polarity by 33 derivatives. As demonstrated by three matched molecular pairs, l-amino acids were preferred over d-amino acids. A rigidification to a six-membered system stabilized the bioactive conformation for the on-target *Escherichia coli* gyrase, but did not improve antimicrobial activity. Compound CN-CC-861, carrying a propargyl side chain, had more than 16-fold lower minimal inhibitory concentration (MIC) values against *Enterococcus faecalis*, *Staphylococci* and *Acinetobacter* strains, compared to known analogues. Moreover, CN-CC-861 retained activity against multidrug-resistant enterococci, displayed strong bactericidal activity, moderate-low frequencies of resistance and *in vivo* efficacy in a neutropenic thigh infection model with *E. coli*. Overall, the findings will guide the design of new promising structures with higher activities and broader spectrum.

## Introduction

**Figure 1:**
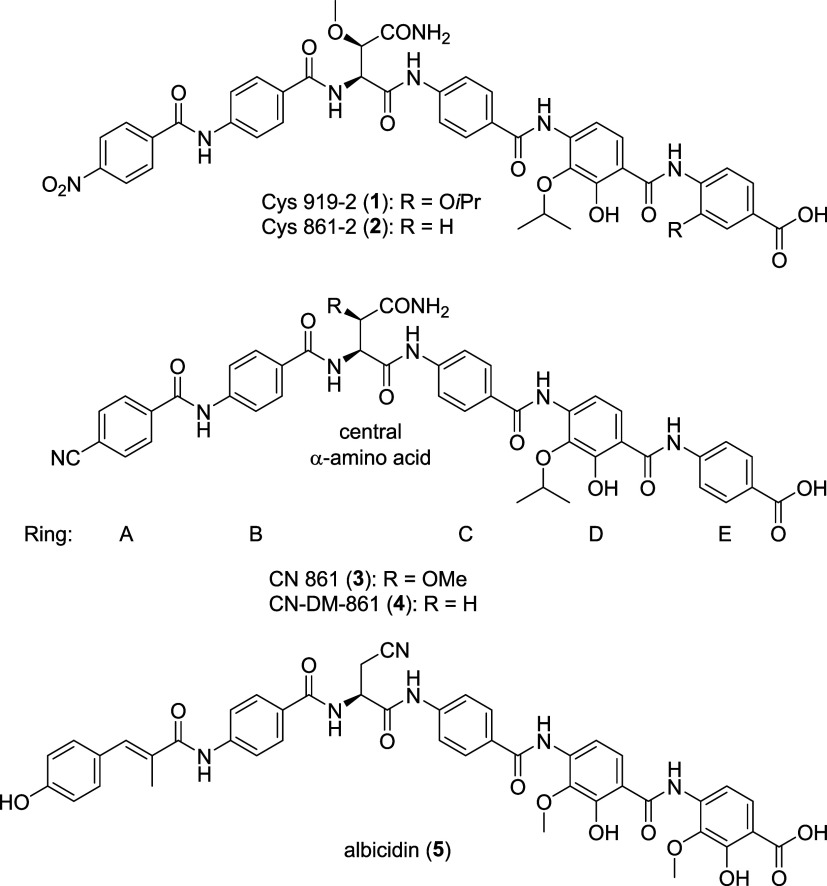
Structures of natural cystobactamids 919–2 (**1**) and 861–2 (**2**), synthetic analogues CN 861 (**3**) and CN-DM-861 (**4**) and albicidin.

Infections, due to multidrug-resistant bacterial pathogens, are a major cause of morbidity and mortality.^1^ Among the “priority pathogens”, as designated by the WHO,^2^ many belong to the group of Gram-negative bacteria. Owing to their complex membrane structure, general rules for the development of permeable antibacterial drugs are just emerging.^3−5^ Consequently, no novel antibacterial scaffold with significant activity against Gram-negative bacteria was commercialized since the discovery of quinolones in the 1960s.^6^ Recent efforts to explore microbes as sources of novel antibacterials^7^ have led to the discovery of cystobactamids and the structurally related albicidins (Figure 1).^8,9^ Their unique hexapeptidic structures feature linearly connected *para*-amino benzoic acids (PABAs)^10^ as well as a central α-amino acid derived from asparagine. Although relatively large, this novel compound class shows remarkably high and resistance-breaking activity against Gram-positive and -negative bacteria. The compounds inhibit the bacterial gyrase and bacterial topoisomerase IV, with a binding mode that is unique and distinct from other classes.^11^

In recent years, the establishment of total syntheses for cystobactamids^12,13^ and albicidins^14,15^ opened the possibility to improve *in vitro* activity and spectrum. First, structure–activity relationship (SAR) investigations of the central amino acid revealed *inter alia* that the methoxy group in natural cystobactamid 861–2 can be omitted, and that the cyano group of albicidin can be replaced by a triazole among other residues,^16−18^ moieties that were kept in first congeners with *in vivo* activity^16,19^ such as CN-DM-861 (Figure 1). Due to the high relevance of the central α-amino acid, this work was devoted to the study of the SAR in a systematic manner through 33 new cystobactamid analogues. The design was guided by the biological assessment of the minimal inhibitory concentration (MIC) on a small panel of five bacterial strains. Highly active analogues were further evaluated on secondary panels comprising a higher variety of bacteria. Through this process, a new promising lead compound with a surprisingly simple functional group, enhanced broad-spectrum activity and resistance-breaking properties was found.

## Results and Discussion

**Figure 2:**
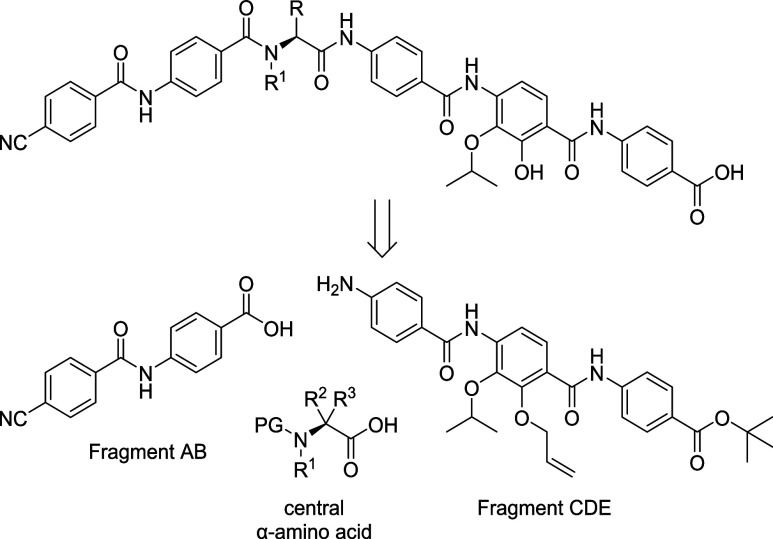
Retrosynthesis of cystobactamids with modifications at the central amino acid. PG = protecting group.

**Figure 3:**
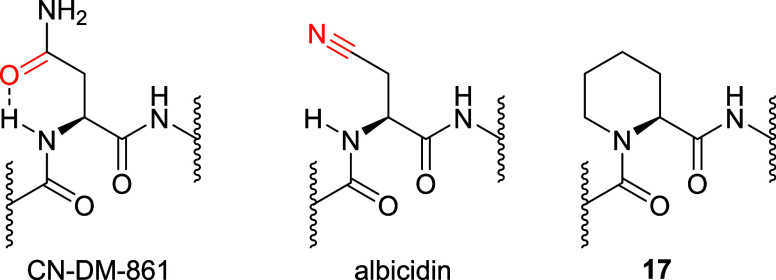
Structures of the central α-amino acid moiety in CN-DM-861 (**4**) and albicidin (**5**) and their HBA (red) as well as the rigidified l-picolinic acid derivative **17**.

**Scheme 1:**
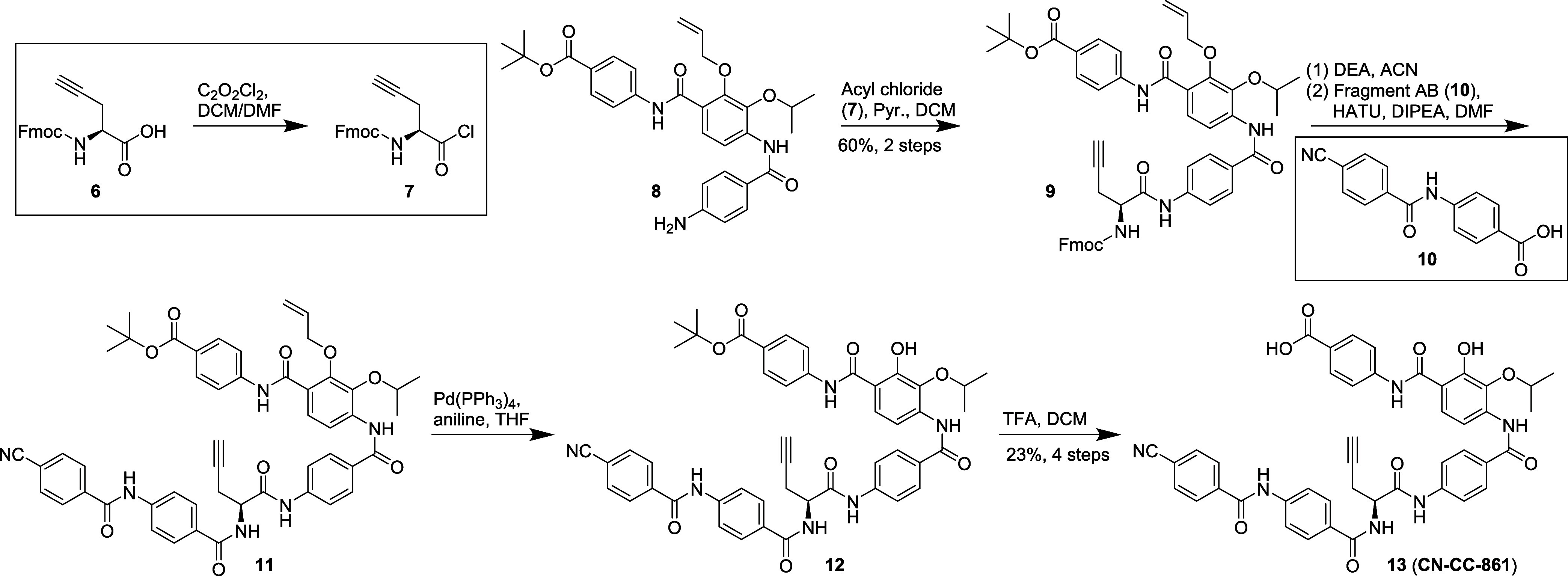
Fragment Connection to the Full-Length Cystobactamid and Global Deprotection Exemplified by the Synthesis of CN-CC-861

The introduction of the central α-amino acid variations were carried out at a late stage, as the longest linear sequence in the synthesis of cystobactamids currently involves about 15 steps. For this purpose, the central moiety was linked to complete, fully functionalized AB and CDE fragments (Figure 2).^20^ For the connection of the central α-amino acid to the CDE fragment, the former was converted to the respective acyl chloride.^21^ To avoid racemization, pyridine was employed in the subsequent amide coupling. Alternatively, the central amino acid was directly coupled to the CDE fragment with ethyl 2-ethoxyquinoline-1(2H)-carboxylate (EEDQ) or propanephosphonic acid anhydride (T3P).^20^ Upon the usage of EEDQ, a significant conversion of the amino acid to the ethyl ester was observed as side reaction due to the low nucleophilicity of the aniline. The assembly of the three fragments followed by global deprotection is exemplified for compound CN-CC-861 (Scheme 1). It is worth mentioning that the previously used phenylsilane as scavenger can be substituted by aniline during allyl deprotection, which leads to fewer side products.^20^ For alkyne-bearing analogues, the application of silanes was disadvantageous, since an unwanted reduction of the alkyne to the alkene was observed during deallylation. Both albicidin (**5**) as well as CN-DM-861 (**4**) have a hydrogen bond acceptor (HBA) in the side chain of the central amino acid (Figure 3). To explore its relevance, in a first step compounds **13**–**30** were investigated for their antibacterial activity (Table 1).

The introduction of the thiazole as in **14** or the *N*,*N*-dimethyl asparagine as in **15** was well-tolerated but led to a loss of activity against the *Pseudomonas aeruginosa* wild type. Additionally, the exchange of the amide for a sulfonamide as in **16** was well tolerated. Notably, alkyne CN-CC-861 (**13**) depicted high activity against all strains with equal or better MIC values than the reference compound CN-DM-861 (**4**), including *P. aeruginosa*. The latter finding implies that the HBA/hydrogen bond donor (HBD) abilities of the original amide moiety were not essential for activity on the tested panel. We then rigidified the central amino acid. The six-membered l-picolinic acid derivative **17** was chosen to mimic a cyclic conformation in CN-DM-861 (**4**) resulting from an intramolecular hydrogen bond (Figure 3).

**Figure 4:**
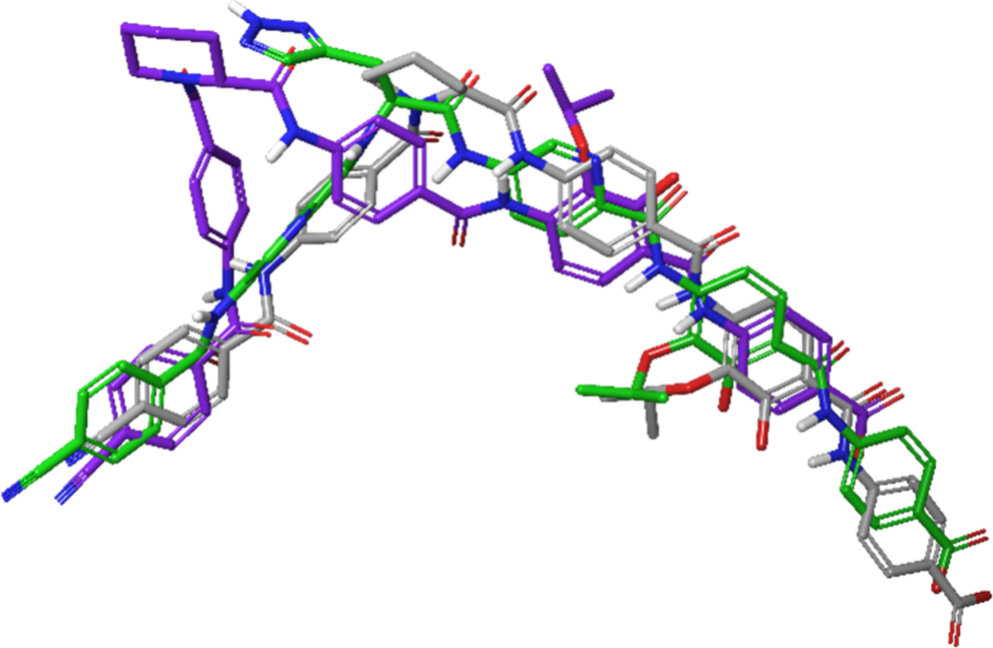
Overlay of the docking poses of Albi-1 (green ligand), compound **17** (gray ligand) and **18** (purple ligand) with Glide^23^ in the cryo-EM structure of *E. coli* gyrase holocomplex with 217 bp DNA (PDB: 7Z9K).^22^

To determine the importance of the configuration, the respective d-enantiomer **18** was also synthesized and tested. The comparison of the activities indicated a slight preference for the l-configurated amino acid **17**. More precisely, **17** shows a nearly 6-fold lower IC_50_ value on the *Escherichia coli* gyrase and more than a 30-fold lower IC_50_ on topoisomerase IV. The low IC_50_ value on gyrase of 0.18 μM, similar to CN-DM-861 (**4**), suggests that the rigidification in **17** stabilizes the bioactive conformation in the *E. coli* gyrase compared to its enantiomer **18**, but also compared to the open chain analogs **22** and **23** with similar alkyl chain length, yet slightly higher IC_50_ values of 0.28 and 0.45 μM, respectively, that might reflect higher entropy costs upon binding. The hypothesis, regarding the stereochemical preference for **17***vs***18**, was affirmed by docking the two compounds into the published *E. coli* gyrase binding site of the albicidin analogue Albi-1.^22^ Preceding docking studies with Albi-1 showed that the double deprotonated form adopts a comparable pose to its cryogenic electron microscopy (cryo-EM) structure (Figure S1). While the docked l-picolinic acid analogue **17** with the same ionization state was able to maintain the overall conformation of Albi-1, the best predicted pose of the respective d-picolinic acid **18** scored significantly worse (Table S6) and adopted a strongly deviating pose (Figure S2). Although both picolinic acid analogues were situated in the DNA binding region with their *N*-terminal fragment, the altered positioning of the central amino acid in **18** resulted in a completely different alignment of the B, C, D and E rings compared to Albi-1 (Figure 4).

An introduction of a 4-oxo functionality into the l-pipecolinic ring was well tolerated and led to equal or higher activity of compound **19** (Table 1). Morpholine derivative **20**, on the other hand, performed worse than the piperidine **17** against the *E. coli* wild type. A rigidification and ring contraction to a 2-azabicyclo[2.1.1]hexane as in the constitutional isomer **31** substantially diminished the activity (Table 2). As the alkyne CN-CC-861 (**13**) demonstrated a positive impact of an aliphatic system on antibacterial activity, reduced analogues **21** and **22** were synthesized for comparison.

**Table 1:**
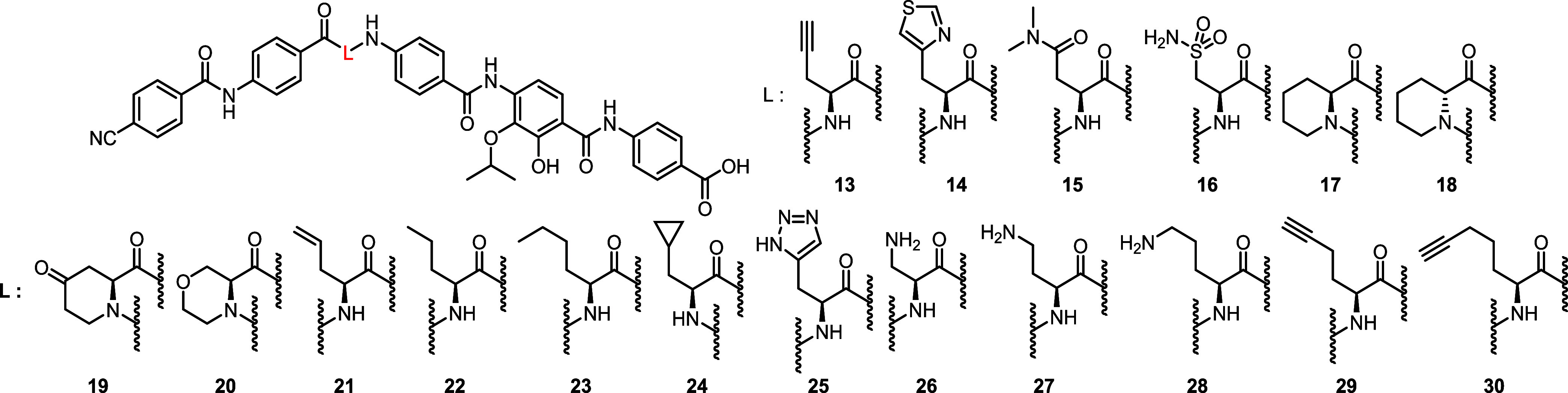
MIC and IC_50_ Values of Cystobactamids CN-CC-861 (**13**)-30 with Modified Central Amino Acid Compared to CN-DM-861 and Ciprofloxacin (CIP)

	MIC [μg/mL]						
compound	E. coli WT (BW25113)	E. coli Δ*acrB*	E. coli LM705 (S83L, D87N, S80I, Δ*acrR*, Δ*marR*)	Staphylococcus aureus (ATCC 29213)	P. aeruginosa WT (Pa14)	P. aeruginosa Pa14Δ*mexAB*	IC_50_ [μM] E. coli gyrase/TI IV^a^
**13** (CN-CC-861)	≤0.03	≤0.03	≤0.03–0.2	0.02	0.5	≤0.03	0.23:0.47
**14**	n.d.	≤0.03	8	0.5	>64	0.25	0.34
**15**	0.25	≤0.03	32	8	>64	0.5	1.47
**16**	0.04	0.0125	0.25	1	16	≤0.03–1	n.d.
**17**	0.06	≤0.03	>64	2	64	2	0.18:0.08
**18**	2	≤0.03	>64	4	64	4	1.07:2.44
**19**	≤0.03	≤0.03	32	2	16	1	0.86
**20**	1	≤0.03	>64	0.8	>64	4–8	n.d.
**21**	0.04	0.0125	0.5	≤0.03	0.5	0.125	0.49
**22**	0.16	0.08	1	≤0.03	>64	0.5	0.28
**23**	≤0.03	≤0.03	8	0.25	>64	4	0.45
**24**	0.16	0.16	2	0.25	0.5	0.25	n.d.
**25**	≤0.03	≤0.03	0.5	0.5	1	≤0.03	0.33:0.37
**26**	0.125	0.006	0.05	0.5	0.25	0.25	0.86
**27**	≤0.03	0.125	3.2	3.2	4	1	n.d.
**28**	≤0.03	0.08	>64	>64	>64	2	0.63
**29**	0.04	0.02	0.25	≤0.03	32	2	0.21
**30**	≤0.03	≤0.03	0.5	0.06	64	2	0.23
CIP	0.02	0.001	>6.4	0.2	0.05	≤0.03	0.18:4.95
CN-DM-861 (4)	0.08	0.004	0.125	1	2	0.25	0.13:3.16

a (a) *n* = 1. n.d.: not determined. IC_50_ values determined by the gyrase supercoiling inhibition assay. For selected compounds, IC_50_′s were also determined by a topoisomerase IV relaxation assay (values given after the ‘/’ sign).

**Table 2:**
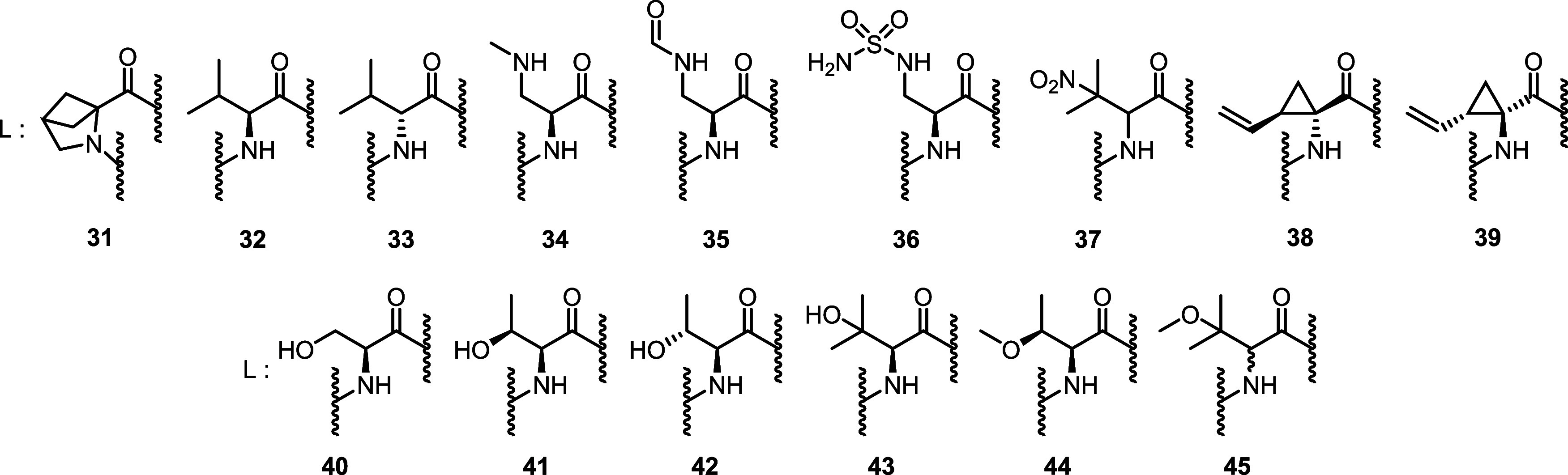
MIC and IC_50_ Values of Cystobactamids **31**-**45** with Modified Central Amino Acid Compared to CN-CC-861 (**13**), CN-DM-861 (**4**) and Ciprofloxacin (CIP)

	MIC [μg/mL]						
compound	*E. coli* WT (BW25113)	*E. coli* Δ*acrB*	*E. coli* LM705 (S83L, D87N, S80I, Δ*acrR*, Δ*marR*)	*S. aureus* (ATCC 29213)	*P. aeruginosa* WT (Pa14)	*P. aeruginosa* Pa14Δ*mexAB*	IC_50_ [μM] *E. coli* gyrase/TI IV^a^
**31**	8	0.5	>64	16	>64	32	n.d.
**32**	1.6	0.2	0.5	0.05	>6.4	6.4	n.d.
**33**	8	1	>64	>64	>64	>64	n.d.
**34**	0.06	≤0.03	>64	0.25	>64	>64	n.d.
**35**	0.16	0.08	≤0.03	0.125	8	0.5	n.d.
**36**	0.125	≤0.03	0.25	2	>64	0.5	n.d.
**37**	0.06	≤0.03	1	≤0.06	1	8	n.d.
**38**	0.06	≤0.03	0.125	≤0.03	8	0.125	0.18/0.27
**39**	n.d.	n.d.	2	0.5	>64	>64	n.d.
**40**	n.d.	n.d	0.06	0.125	n.d.	n.d.	n.d.
**41**	0.0125	≤0.003	0.06	≤0.03	8	0.5	0.09/0.09
**42**	0.04	0.006	0.5	≤0.03	>64	0.5	0.14/0.67
**43**	n.d.	n.d.	0.125–0.25	n.d.	n.d.	0.04	n.d.
**44**	0.16	0.005	0.25	≤0.03	16	1	n.d.
**45**	0.06	≤0.03	2	≤0.03	64	0.25	n.d.
**CN-CC-**861 (13)	≤0.03	≤0.03	≤0.03–0.2	0.02	0.5	≤0.03	0.23/0.47
**CIP**	0.02	0.001	>6.4	0.2–1	0.05	≤0.03	0.65/4.95
**CN-DM-**861 (4)	0.08	0.004	0.125–0.25	1	2	0.25	0.45/3.16

a n.d.: not determined. IC_50_ values determined for selected compounds by the gyrase supercoiling inhibition assay/topoisomerase IV relaxation assay.

Alkene **21** turned out to be more potent than saturated analogue **22** against *P. aeruginosa* strains. However, cyclopropyl analogue **24**, blending properties of unsaturated and saturated systems, showed activities comparable to **21** and **22**. Branched derivative **32** based on l-valine proved to be less active (Table, Table S1) while d-valine analogue **33** was mostly inactive. Both triazole **25** and primary amine **26** possess an HBD in addition to at least one HBA, which probably contributes to their high activity comparable to alkyne CN-CC-861 (**13**). Similar to the extension of the alkyl chain from **22** to **23**, neither the elongations of the alkyne from **13** to **29** and **30**, nor of the amine side chain from **26** to **27** and **28** led to higher activities (Table 1). Therefore, we concluded that the optimal location of both substituents was at the β-position of the amino acid. Methylation as well as formylation and sulfamylation of the β-amine also resulted in considerably increased MIC values in **34**, **35** and **36** (Table 2). A formal nitration of the β-hydrogen of valine resulted in **37**, a racemic mixture with moderate activity in the primary panel, but high potency against additionally tested *Acinetobacter baumannii* strains (Table S2).

By integration of the α-carbon atom into a cyclopropane ring, **38** as rigidified analogue of the β-vinyl derivative **21** was obtained. While the 1*S*,2*R* derivative **38** retained broad-spectrum activity, the enantiomer **39**, resembling the respective d-configuration, showed significantly reduced or total loss of activity on many strains (Table S2).

Finally, six additional derivatives **40**–**45** containing an oxygenated β-position were prepared with an increasing degree of 0–3 methyl substitutions at the β-carbon and oxygen. All derivatives exhibited robust broad-spectrum activity; an increasing level of methyl substitutions led to lower activities against the multidrug-resistant *E. coli* strain LM705, but improved activities against *A. baumannii* strains (Tables 2 and S3).

Based on the results of the small panel, a set of six promising cystobactamids underwent further MIC testing on an extended panel of clinically relevant Gram-negative and -positive pathogens (Table 3). Amino acid analogues CN-CC-861 (**13**), **25** and **26** depicted the broadest spectrum coverage. Particularly, alkyne CN-CC-861 (**13**) exhibited notable improvements compared to CN-DM-861 (**4**) in terms of enhanced activity against *A. baumannii*, *Enterococcus faecalis* and *S. aureus*. Amine **26** shared a very similar activity pattern to CN-DM-861 (**4**) with exceeding potency against *Proteus mirabilis*, but insufficient activity against *P. aeruginosa* ESBL2. Triazole **25** was lacking activity against *Enterobacter aerogenes*, but mostly retained potency against other strains with improvements against *S. aureus*. Derivatives CN-CC-861 (**13**), **21**, **22** and **24** also impressed by their excellent activity against *A. baumannii*, including ciprofloxacin- and CN-DM-861 (**4**) resistant strains (Tables S1 and S4).

**Table 3:** MIC Values of Selected Cystobactamid Analogues on an Extended Panel of Pathogenic Bacteria Compared to CN-DM-861 and Ciprofloxacin (CIP)

	MIC (μg/mL)							
	CN-DM-861 (**4**)	CIP	CN-CC-861 (**13**)	**21**	**22**	**24**	**25**	**26**
gram-negative strains								
*A. baumannii* DSM 30008	0.5	0.2–0.32	≤0.03	≤0.03	≤0.03	≤0.03	0.125	0.125
*Citrobacter freundii* DSM 30039	≤0.03–0.125	≤0.03	≤0.03	≤0.03	0.06	≤0.03	0.06	≤0.03
*E. aerogenes* DSM 30053	0.25–0.5	0.08–0.1	0.5	16	>64	>64	>64	0.5
*Enterobacter cloacae* DSM 30054	0.25–1	0.1–0.5	0.5	4	0.125	0.5	1	0.125
*E. coli* DSM 1116	≤0.03	0.01	≤0.03	≤0.03	0.125	≤0.03	≤0.03	≤0.03
*E. coli* WT-3 [*gyrA*(S83L,D87G)]^a^	0.06–0.125	0.32–0.8	≤0.03	0.125	0.25	1	0.25	0.06
*Klebsiella pneumoniae* DSM 30104	0.25–64	0.01–0.1	>64	>64	>64	>64	0.25	>64
*P. aeruginosa* ESBL1 DSM 24600	64	3.2–6.4	64	>64	>64	>64	32	>64
*P. aeruginosa* ESBL2 DSM 46316	1	0.1–0.4	0.5	2	>64	>64	16	64
*P. mirabilis* DSM 4479	32–64	0.02	64	64	0.25	0.125	32	1
*Proteus vulgaris* DSM 2140	0.25–0.5	≤0.06	0.125	0.06	0.25	0.25	0.06	0.25
*Serratia marcescens* DSM 30121	64	0.2	>64	>64	>64	>64	64	64
gram-positive strains								
*E. faecalis* ATCC 29212	0.5	0.8	≤0.03	≤0.03	≤0.03	≤0.03	≤0.03	0.5
*S. aureus* ATCC 29213	0.25–1	0.4–0.8	≤0.03	≤0.03	≤0.03	≤0.03	≤0.03	0.25
*Staphylococcus epidermidis* DSM 28765	≤0.06	0.2–0.32	≤0.03	≤0.03	≤0.03	≤0.03	≤0.03	0.06
*Streptococcus pneumoniae* DSM 20566	≤0.03–0.125	0.8	≤0.03	≤0.03	≤0.03	≤0.03	≤0.03	0.06

a *E. coli* strain with the mentioned mutations in the gyrA subunit.

### Microbiological Profile of CN-CC-861

Because of the superior efficacy of alkyne CN-CC-861 (**13**), the derivative was further evaluated against multidrug-resistant clinical strains (Tables 4 and S5). The results demonstrate that CN-CC-861 (**13**) surpassed the activity of CN-DM-861 (**4**) against most of the tested strains. The clinical isolate panels disclosed reduced median MICs of CN-DM-861 (**4**) and ciprofloxacin toward Gram-positive *Enterococci* and *Staphylococci*, whereas the antibacterial activity of CN-CC-861 (**13**) remained high against these strains. A high activity of CN-CC-861 (**13**) was also shown against several *Acinetobacter* strains that were less susceptible to CN-DM-861 (**4**). In contrast, moderate median MIC’s were observed for *K. pneumoniae* and *P. aeruginosa*. For *P. aeruginosa*, the pairwise testing of PA14 wild type versus the Pa14Δ*mexAB* strain demonstrated a significant increase of activity of many cystobactamid analogues in the efflux-deficient mutant. This implied contribution of efflux to the intrinsic resistance of *P. aeruginosa*. A hint for a cause behind the moderate activity against *K. pneumoniae* was obtained by a heptose lacking LPS mutant (*K. pneumoniae* KP10581; waaC::Tn30), which was susceptible (MIC = 0.25 μg/mL). This implies that the penetration of the outer membrane was a key issue hampering activity for *K. pneumoniae*.

**Table 4:** Antibiotic Activities of CN-DM-861 (**4**), CN-CC-861 (**13**) and Ciprofloxacin (CIP) against Susceptible and Multidrug-Resistant Bacteria^a^

		median MIC (μg/mL)			
genus	species	CN-DM-861 (**4**)	CN-CC-861 (**13**)	CIP	resistance phenotypes
*Acinetobacter* 8 strains	*Acinetobacter johnsonii*, *Acinetobacter lwoffii*, *Acinetobacter ursingii* and *A. baumannii*	4	0.06–0.125	0.1–0.2	4× susceptible, 4× 3MRGN
*Klebsiella* 5 strains	*Klebsiella oxytoca*	0.5	0.125	0.0125	2× susceptible, 2× 2MRGN, 1× 4MRGN
*Klebsiella* 5 strains	*K. pneumoniae*	2	8	>6.4	1× susceptible, 1× 2MRGN, 1× 3MRGN, 2× 4MRGN
*Pseudomonas* 11 strains	*P. aeruginosa*	>64	8	3.2	5× 3MRGN, 6× 4MRGN
*Enterococcus* 17 strains	not specified	64	1	64	17× VRE
*Staphylococcus* 10 strains	*S. aureus*	4–8	0.125	>6.4	5× MSSA, 5× MRSA

a 2/3/4 MRGN: Multidrug-resistant Gram-negative bacteria with resistance against 2, 3, or 4 of the 4 antibiotic groups acylureidopenicillins, third-generation cephalosporins, carbapenems or fluoroquinolones; example 3MRGN: Resistance against 3 of the 4 antibiotic groups. VRE: Vancomycin-resistant Enterococcus. MSSA: Methicillin-susceptible *Staphylococcus aureus*. MRSA: Methicillin-resistant *Staphylococcus aureus*.

**Figure 5:**
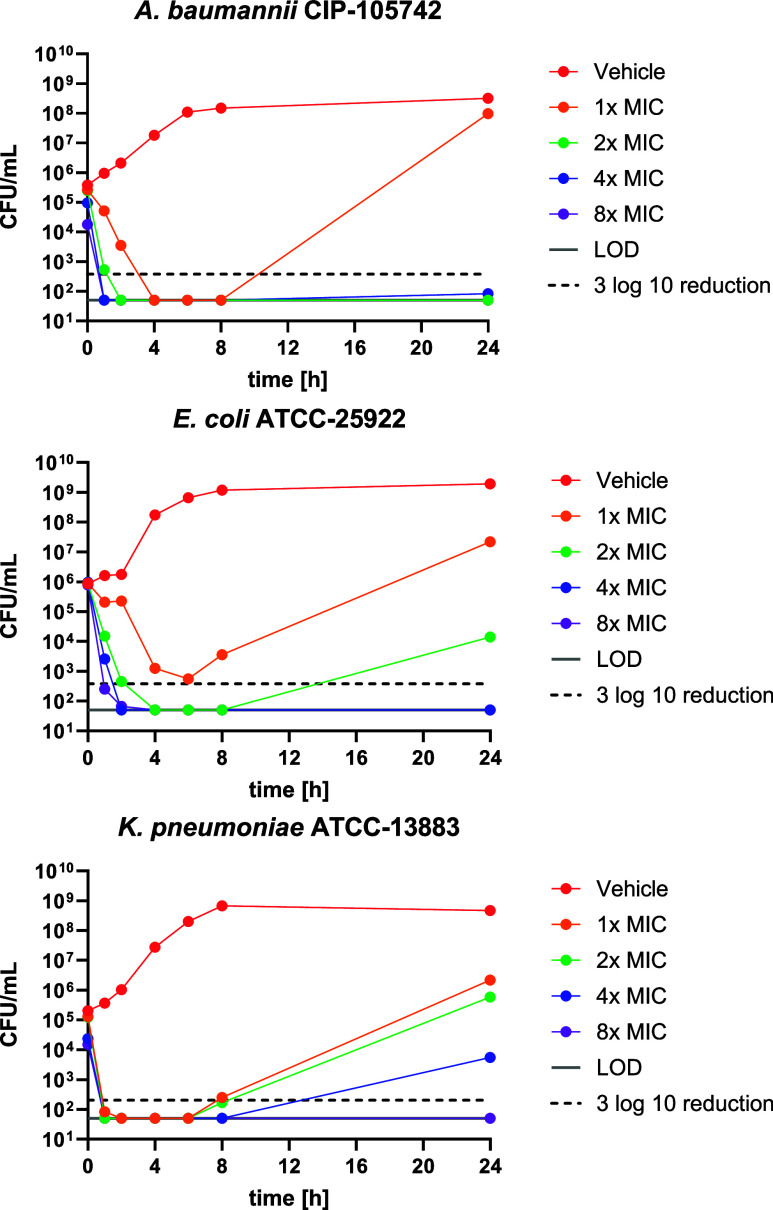
Time-dependent killing of bacteria by CN-CC-861 (**13**). Time-kill curves for *A. baumannii* CIP-105742 (MIC = 0.03 μg/mL), *E. coli* ATCC-25922 (MIC = 0.016 μg/mL) and *K. pneumoniae* ATCC-13883 (MIC = 2 μg/mL). LOD = Limit of detection.

In order to assess the cidality of CN-CC-861 (**13**), time-kill curve experiments were performed with *A. baumannii*, *K. pneumoniae* and *E. coli*. CN-CC-861 (**13**) exerted a strong and rapid bactericidal activity that led to a reduction of colony-forming units (cfu) by three log_10_ units within 1–2 h in all three species at 4× MIC (Figure 5). A regrowth was observed to a stronger extent for *K. pneumoniae* (4× MIC or lower) than for *E. coli* (2× MIC or lower) or *A. baumannii* (1× MIC only). The generation of resistance to CN-CC-861 (**13**) was studied further in six different strains of *A. baumannii*, *P. aeruginosa* and *E. coli* (Table 5). At 4× MIC, resistant clones formed with frequencies between 4 × 10^–8^ and 2 × 10^–10^. Such frequencies of resistance were comparable but, on average, slightly higher than those found for ciprofloxacin. The MICs of resistant mutants were determined and turned out to decrease in the order *E. coli* (32–200 μg/mL) ≈ *P. aeruginosa* (8–256 μg/mL) > *A. baumannii* (2–25 μg/mL) (Table 5). These pronounced differences imply that there were no uniform mechanisms of resistance against CN-CC-861 (**13**), but strain dependent ones.

**Table 5:** Frequencies of Resistance (FoR) and MIC Shifts Associated with Resistant Mutants for Six Bacterial Strains against CN-CC-861 and Ciprofloxacin

	FoR (4× MIC)		MIC shift CysR mutants *vs* WT^a^	
	CN-CC-861 (**13**)	CIP	CN-CC-861 (**13**)	CIP
*A. baumannii* DSM 30008	2 × 10^–10^	7 × 10^–10^	6–25	1–2
*A. baumannii* CIP105742	1 × 10^–8^	1 × 10^–9^	<2	1–4
*E. coli* MG1655/K12	4 × 10^–8^	2 × 10^–10^	32–128	0.5–4
*E. coli* ATCC 25922 Δ*tolC*	4 × 10^–8^	3 × 10^–10^	50–200	1–2
*P. aeruginosa* Pa14Δ*mexAB*	3 × 10^–9^	2 × 10^–8^	>256	20–160
*P. aeruginosa* PAO750 (Δ*mexAB*-*oprM*, Δ*mexCD*-*oprJ*, Δ*mexEF*-*oprN*, Δ*mexJK*, Δ*mexXY*)	6 × 10^–9^	1 × 10^–11^	8–32	0.5–8

a *n* = 9 for CN-CC-861 and *n* = 18 for CIP, with *n* being the number of clones tested in the MIC shift determination.

### Physicochemical Profiling of Selected Cystobactamids

We first assessed experimental log *D*_7.4_ values: they were between 1.35 and 2.47, an expected range for antibacterials active against Gram-positive bacteria but rather high for molecules active against Gram-negatives (Table 6).^24^ Next, the thermodynamic solubility at pH 7.4 and pH 9.0 was measured. For the profiling, we selected analogues with promising antimicrobial activity. Additionally, we included cystobactamids with hydrophilic side chains to investigate their influence on physicochemical parameters, especially solubility and plasma protein binding. Several compounds with unpolar side chains, including CN-CC-861 (**13**), alkene **21** as well as alkanes **22** and **23**, were neither detectable at pH 7.4 nor at pH 9.0. Surprisingly, the amines in **26** and **27** did not contribute to a higher solubility, presumably due to their zwitterionic character in the investigated pH range. On the other hand, solubility was clearly enhanced by polar side chains as the morpholine **20** as well as alcohol **41**.

**Table 6:** Thermodynamic Solubility, Plasma Protein Binding and log *D*_7.4_ Values of Selected Cystobactamid Derivatives

	aq solubility [μg/mL]		plasma protein binding [%]		
compound	pH 7.4	pH 9.0	M^a^	H^b^	log *D*_7.4_
**CN-CC-861** (**13**)	-	-	100.0 ± 0.0	100.0 ± 0.0	2.26
**26**	<1	14	99.89 ± 0.1	100.0 ± 0.0	1.41
**24**	<1	270	100.0 ± 0.0	98.17 ± 2.7	n.d.
**27**	<1	22	n.d.	n.d.	1.35
**38**	<1	10	100.0 ± 0.0	100.0 ± 0.0	2.47
**39**	<1	11	100.0 ± 0.0	100.0 ± 0.0	n.d.
**20**	37	980	n.d.	n.d.	n.d.
**16**	<1	24	99.54 ± 0.7	99.95 ± 0.0	1.56
**41**	163	311	94.36 ± 2.0	99.39 ± 0.2	n.d.
**42**	1	46	86.43 ± 2.4	100.0 ± 0.0	n.d.
CN-DM-861 (**4**)	<1	56	98.39 ± 2.3	99.71 ± 0.18	1.49

a Mouse.

b Human. n.d.: not determined.

Finally, the mouse and human plasma protein binding (PPB) was determined (Table 6). In line with the low solubility, the lipophilic amino acid analogues (*e.g*., CN-CC-861) increased the PPB to 100%, when compared to the PPB of 98.4% in mouse plasma for CN-DM-861 (**4**). Sulfonamide **16** retained high binding, while alcohol **41** as well as its diastereomer **42** showed a decreased bound fraction, although not for human plasma protein.

### *In Vivo* Efficacy Study of CN-CC-861

**Figure 6:**
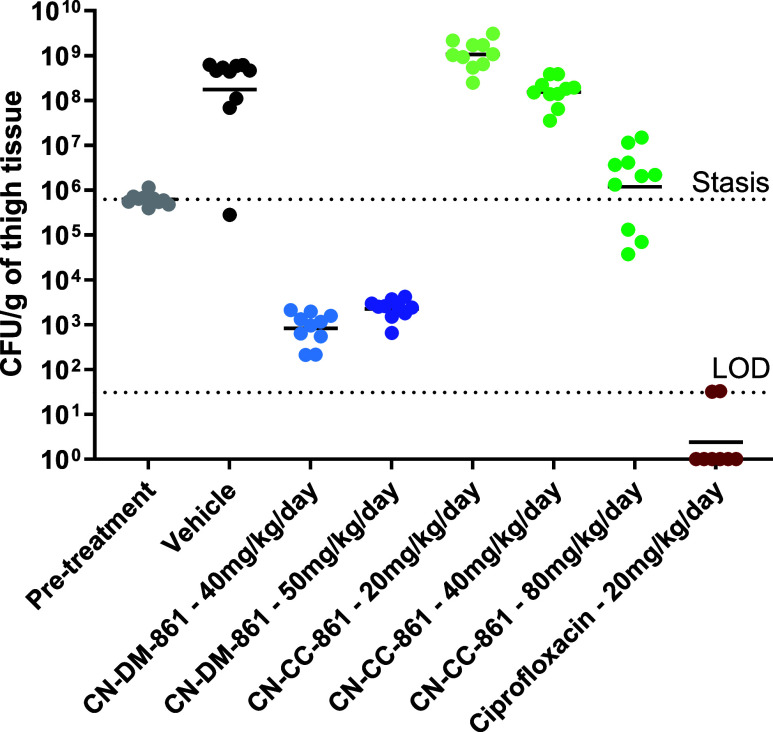
*In vivo* efficacy study of cystobactamids and ciprofloxacin in a neutropenic mice thigh infection model (*n* = 5/group; *n* = 4 in pretreatment group) with *E. coli* ATCC 25922 (5 × 10^5^ cfu/thigh). Dosing q6h *i.v.* starting 1 h after infection. LOD = Limit of detection.

Due to its high *in vitro* activity, CN-CC-861 (**13**) was profiled in an *in vivo* mouse model. The cytotoxicity assay indicated a low cytotoxicity against the HepG2 and CHO cell lines with IC_50_’s of 30 and ≥100 μM, respectively. The murine plasma stability was sufficient, with around 80% of the parent compound remaining after 4 h. Moreover, dosing 20 mg/kg four times at an interval of 6 h (q6h), summing up to a daily dose of 80 mg/kg, was well tolerated in healthy mice. To determine the *in vivo* efficacy, a neutropenic thigh infection model with *E. coli* ATCC 25922 was carried out with CN-CC-861 (**13**) (MIC = 0.03 μg/mL in dosing solution) and the lead structure CN-DM-861 (**4**) (MIC = 0.125 μg/mL in dosing solution) as a reference (Figure 6).

The cystobactamids were administered to male CD-1 mice q6h starting with the first injection 1 h post infection. The animals were divided into three groups, and single doses of 5, 10, and 12.5/20 mg/kg were applied, respectively. Twenty-five hours after infection, bacterial burden in thigh was determined (Figure 6). However, as animals reached the human end point, vehicle-treated group was terminated 20 h and CN-CC-861 (**13**) groups 21 h post infection. While the two lower doses were not efficacious, a reduction of bacterial load by two log_10_ units compared to the vehicle control was observed for the highest dose of 80 mg/kg/day; here, the bacterial load reduction was close to stasis. However, compared to the previously published CN-DM-861 (**4**), where a reduction of the bacterial load was observed in all dosing groups,^16^ similar as observed in this study, albeit different end points, CN-CC-861 (**13**) did not exhibit similar efficacy. We attributed this lack of efficacy to the poor pharmacokinetic properties of CN-CC-861 (**13**) and in particular to the low solubility and the strong mouse protein binding (see above) that prevented the high *in vitro* potency of CN-CC-861 to fully translate into high *in vivo* efficacy.

### Structure–Activity Relationships

For the determination of the structure–activity relationships, the broad-spectrum antibiotic coverage was the most important metric in this work. The functional inhibition data on *E. coli* gyrase were secondary parameters to investigate the inhibitor binding. While first-generation natural cystobactamids inhibited gyrase much stronger than topoisomerase (TI) IV, we note that the compounds described herein possess a more balanced gyrase *vs* TI IV profile, and that the absolute potency exceeded that of ciprofloxacin and other references (Tables 1 and 2). This might contribute to their superior antibiotic potency. However, target inhibition was tested only for the *E. coli* sequence, and because the values are species-dependent, we did not attempt to correlate them with the broad-spectrum antibiotic profile and therefore excluded them in the following SAR discussion.

**Figure 7:**
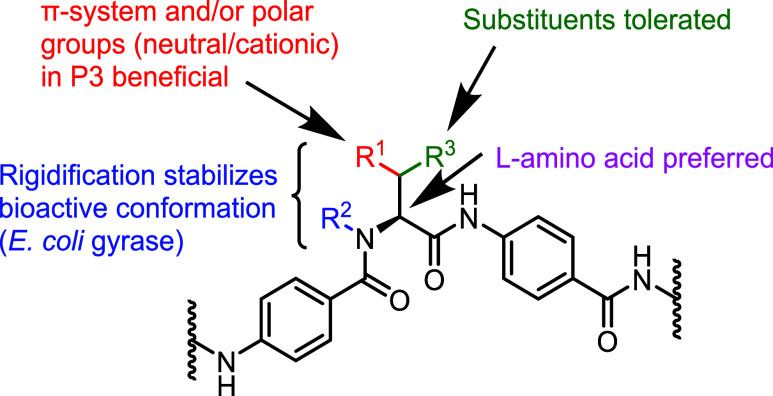
Structure–activity relationships at the central amino acid.

Various residues in the central α-amino acid were tolerated, and an amide in the side chain was not mandatory for antibacterial activity (Figure 7). Positions 3–4 of the amino acid are ideal for functionalities such as a π-system or polar, neutral or cationic groups, while elongated side chains were less potent. Alkyne CN-CC-861 (**13**) showed superior *in vitro* activity, spectrum and resistance-breaking properties compared to other analogues. Basic amine side chains were tolerated, implying that the bacterial membrane allowed for the passage of zwitterionic compounds. The configuration of the α-carbon had a strong impact on the activity. As demonstrated by the pairs **17** and **18**, **32** and **33**, or **38** and **39**, l-amino acids were preferred over the less active d-amino acids. This preference for l-amino acids was affirmed by docking studies of **17** and **18** in the published binding pocket of Albi-1.^22^ Herein, the rigidification to a six-membered system in **17** stabilized the bioactive conformation for *E. coli* gyrase. However, the target-based differences were not linked to an overall increased antimicrobial activity. Possible explanations include the bioactive conformation being different from the permeable conformation or involvement of additional targets in the mechanism of action.

## Conclusions

Through a series of structural modifications, the SAR at the central amino acid of cystobactamids could be elucidated. Several novel analogues with high broad-spectrum activity were discovered and characterized, including the alkyne derivative CN-CC-861 (**13**), that exhibited resistance-breaking properties. However, the modest performance of CN-CC-861 (**13**) in the *in vivo* thigh infection model indicates that future studies must focus on the physiochemical and pharmacokinetic optimization of cystobactamids. In summary, our findings indicate that the cystobactamid scaffold provides a remarkable basis for the development of broad-spectrum antibiotics.

## Experimental Section

### Chemistry

All nonaqueous reactions were carried out in dried glassware in dry solvents under inert conditions unless otherwise noted. Light sensitive reactions were carried out under light exclusion. Commercially available reagents were used without prior purification. Dry solvents (MeCN, DMF, Et_2_O) were taken from a MBraun solvent purification system. THF was freshly distilled over sodium (benzophenone as indicator). Petroleum ether was distilled (60 °C). Et_3_N was freshly distilled over KOH. Other commercially available (dry) solvents were purchased from Merck or Acros Organics.

For reactions under microwave irradiation a CEM Discover S-Class was used with a power maximum of 300 W.

Chromatographic separations by flash chromatography were carried out on a Grace Reveleris X2 (Büchi) with FlashPure EcoFlex cartridges (Büchi) or conducted with the flash purification system Sepacore (Büchi) or Biotage SP using prepacked cartridges (puriFlash by Interchim or chromobond by Macherey-Nagel). A Pure C-850 FlashPrep (Büchi) with FlashPure EcoFlex cartridges (Büchi) was utilized for reversed phase flash chromatography. For manual columns silica gel 60 0.04–0.063 mm; 230–400 mesh (Macherey-Nagel) was used.

Purifications by high performance liquid chromatography (HPLC) were performed by a Thermo Scientific Dionex UltiMate 3000 system with a Phenomenex Luna C18 column (250 mm × 21.2 mm, 5 μm) column under basic (10 mM NH_4_HCO_3_) or acidic (0.1% formic acid or acetic acid) conditions. Alternatively, semipreparative HPLC was performed by using a Waters Alliance 2695 HPLC-system with a 996 diode array detector (λ = 200–350 nm) and a Macherey-Nagel Nucleodur C18 ISIS column (5 μm, 250 mm, diameter = 8 mm). Mass detection was conducted with a Waters Quattro micro API mass spectrometer in negative ionization mode.

Thin-layer chromatography analytics were carried out on precoated silica gel 60 F_254_ plates (Merck) or on Macherey-Nagel aluminum plates coated with silica gel 60 F245. The sample was detected by ultraviolet (UV) light at 254 or 366 nm. Non-UV-absorbent samples were stained by a cerium-ammonium-molybdate, potassium permanganate, ninhydrin, vanillin and anisaldehyde solution.

NMR spectra were measured on different instruments, *i.e*., Bruker Advance-III HD 500 MHz and Bruker Advance-III HD 700 MHz spectrometer, Bruker Ascend 600 MHz with Avance Neo console, Ultrashield 500 MHz with Avance-III HD console, Ascend 400 MHz with Avance- III console, Ascend 400 MHz with Avance-III HD console or Ultrashield 400 MHz with Avance-I console. The chemical shifts for ^1^H, ^13^C and ^19^F spectra are reported in ppm at a temperature of 300 K. ^19^F spectra lack an internal reference. One dimensional ^13^C were measured with ^1^H decoupling. Multiplicities are specified with following abbreviations: s = singlet, d = doublet, t = triplet, q = quartet, hept./sept. = septet, oct = octet, m = multiplet, br = broad signal and combinations thereof.

HPLC-MS reaction controls were carried out by Agilent 1260 Infinity II LC connected to an Agilent 6130 (quadrupole MS) in ESI mode by a Phenomenex Gemini NX-C18 (50 mm × 2 mm, 3 μm) column. The gradient went from 0–100% acetonitrile to water with 0.1% formic acid in both solvents over three minutes at a flow rate of 1.5 mL/min. All isolated compounds were analyzed by HPLC to confirm a purity of ≥95%.

High-resolution mass spectra were measured at a Bruker maXis HD spectrometer in positive or negative ESI mode or at a Micromass LCT with lock-spray unit and injection *via* loop modus in a Waters (Alliance 2695) HPLC device. Alternatively, a Micromass Q-TOF was used in combination with a Waters Aquity UPLC device. The ionization occurred through electron spray ionization. Calculated and found masses are reported.

The specific optical rotation [α] was measured with a polarimeter type 341 from PerkinElmer at λ = 589.3 nm (sodium D line) in a 10 cm quartz cuvette. It is given in 10^–1^ cm^2^ g^–1^. The concentration c is given in 10 mg mL^–1^.

A Christ α 1–4 LCSbasic was used for the lyophilization of the products after purification by HPLC.

### General Procedures

#### General Procedure 1: Amide Coupling with T3P

0.18 mmol of amine (1.00 equiv) and 0.27 mmol of the desired acid (1.50 equiv) were added to a dry flask and further dried under high vacuum. 0.61 mmol dry pyridine (3.40 equiv) and 0.4 mL dry EtOAc were added under nitrogen atmosphere. The reaction mixture was cooled down to 0 °C. 0.25 mL T3P solution (50 wt % in EtOAc, 0.42 mmol, 2.30 equiv) was added very slowly while keeping the temperature below 0 °C. The reaction was stirred at 0 °C overnight and controlled by liquid chromatography–mass spectrometry (LCMS). After completion, the reaction was quenched with 4 mL 1 M HCl and 12 mL brine and extracted with 3 × 6 mL EtOAc. The combined organic phases were washed with a sat. NaHCO_3_ solution and the solvent was removed under reduced pressure. The crude product was purified by flash chromatography with petroleum ether and EtOAc or used without further purification.

#### General Procedure 2: Fmoc Deprotection

0.09 mmol (1.00 equiv) of the crude carbamate was dissolved in 0.8 mL ACN and 0.3 mL diethylamine (2.9 mmol, 31.5 equiv) at 0 °C and stirred for 1 h. The reaction was controlled by LCMS. The solvent was evaporated under reduced pressure and coevaporated with ACN 3 times. Optionally, the crude product was purified by RP flash or RP HPLC with ACN and water mixed with 0.1% HCOOH.

#### General Procedure 3: Amide Coupling with HATU

82.6 μmol of the desired carboxylic acid (1.20 equiv) and 78.9 μmol HATU (1.20 equiv) were added to a separate flask and dried under high vacuum. 0.5 mL dry DMF and 38 μL DIPEA (3.00 equiv) were added under nitrogen atmosphere and the reaction was stirred for 30 min. The solution was added to the amine or amine hydrochloride and stirred at 0 °C. The reaction was controlled by LCMS. After completion, the reaction was quenched with 8 mL of 0.1 M HCl and 4 mL brine. The inorganic layer was extracted with 3 × 6 mL of EtOAc. The organic phases were combined and washed with 2 × 5 mL brine. The solvent was removed under reduced pressure. The crude product was used without further purification.

#### General Procedure 4: Amide Coupling with HATU

65.5 μmol of the desired amine (hydrochloride) (1.00 equiv), 79.0 μmol HATU (1.20 equiv) and 79.0 μmol of the desired carboxylic acid (1.20 equiv) were added to a dry flask and further dried under high vacuum. 0.4 mL dry DMF and 35 μL DIPEA (3.1 equiv) were added under nitrogen atmosphere at 0 °C. The solution was stirred at 0 °C and controlled by LCMS. After completion, the reaction was quenched with 6 mL of 0.1 M HCl and 10 mL brine. The inorganic layer was extracted with 3 × 4 mL of EtOAc. The organic phases were combined and washed with 2 × 4 mL brine. The crude product was used without further purification.

#### General Procedure 5: Allyl Deprotection with Palladium and Phenylsilane

65.5 μmol of the desired allyl protected alcohol and 198 μmol phenylsilane (3.00 equiv) were added to a dry flask under nitrogen atmosphere. 1.2 mL dry THF and 6.5 μmol tetrakis(triphenylphosphine)palladium(0) (0.10 equiv) were added and the mixture was stirred for 3 h at rt. The reaction was controlled by LCMS. After completion, the solvent was removed under reduced pressure. Three ml 0.1 M HCl and 10 mL brine were added to the residue and extracted with 3 × 4 mL EtOAc. The combined organic phases purified by flash chromatography with petroleum ether and EtOAc mixed with 2% acetic acid.

#### General Procedure 6: Allyl Deprotection with Palladium and Aniline

65.5 μmol of the desired allyl protected alcohol and 198 μmol aniline (3.00 equiv) were added to a dry flask under nitrogen atmosphere. 1.2 mL dry THF and 6.5 μmol tetrakis(triphenylphosphine)palladium(0) (0.10 equiv) were added and the mixture was stirred for 3 h at rt. The reaction was controlled by LCMS. After completion, the solvent was removed under reduced pressure. Three ml 0.1 M HCl and 10 mL brine were added to the residue and extracted with 3 × 4 mL EtOAc. The combined organic phases were purified by flash chromatography with petroleum ether and EtOAc mixed with 2% acetic acid or with CH_2_Cl_2_ and methanol.

#### General Procedure 7: *tert*-Butyl Ester Deprotection with TFA

65.5 μmol of the desired *tert*-butyl protected acid (1.00 equiv) was added to a dry flask and further dried under high vacuum. 0.5 mL dry CH_2_Cl_2_ and, if necessary, 14 μL anisole (2 equiv) were added under nitrogen atmosphere and the solution was cooled down to 0 °C. 0.24 mL of trifluoroacetic acid (3.1 mmol, 54 equiv) was added under nitrogen atmosphere. The solution was stirred for 3 h at 0 °C and controlled by LCMS. After completion, the solvent was removed under reduced pressure. The residue was coevaporated with CH_2_Cl_2_ twice. The crude product was purified by RP-HPLC.

#### General Procedure 8: Amide Coupling CDE to Central AA with EEDQ

0.09 mmol of the desired aniline (1 equiv) and 0.14 mmol of the desired carboxylic acid (1.5 equiv) were added to a dry flask and were further dried under high vacuum. 0.15 mL dry CH_2_Cl_2_ was added under nitrogen atmosphere and the mixture was cooled down to 0 °C. 33.0 mg EEDQ (0.13 mmol, 1.5 equiv) dissolved in 0.15 mL dry CH_2_Cl_2_ was added to the stirring solution. The reaction was stirred at 0 °C for 30 min and slowly warmed up to rt, afterward. The reaction was controlled by LCMS. After completion, the reaction was quenched with 2 mL 1 M HCl and 6 mL brine and extracted with 3 × 3 mL CH_2_Cl_2_. The combined organic phases were washed with brine and the solvent was removed under reduced pressure. The crude product was purified by flash chromatography.

#### General Procedure 9: Amide Coupling with Acid Chloride

0.11 mmol of the desired Fmoc-protected amino acid (1 equiv), 0.4 dry DCM and one drop of dry DMF were added to a dry flask under nitrogen atmosphere. The mixture was cooled to 0 °C and 17 μL oxalyl chloride (0.2 mmol, 1.4 equiv) was slowly added to the stirring mixture. The reaction was controlled by quenching a sample in methanol and running a TLC with petroleum ether and ethyl acetate. After completion, the solvent was evaporated under reduced pressure. The crude product was dried under high vacuum overnight and directly used without further purification.

0.09 mmol of aniline **8** (1 equiv) and 0.14 mmol of the preformed acid chloride (1.5 equiv) were added to a dry flask and further dried under high vacuum. 1.0 mL dry DCM was added under nitrogen atmosphere and the mixture was cooled down to 0 °C. Twenty-three μL pyridine (0.29 mmol, 3.1 equiv) was added under nitrogen atmosphere and the solution was kept at 0 °C for the whole reaction. The reaction was controlled over LCMS. After completion, the reaction was quenched with 2 mL 1 M HCl and 4 mL water. The aqueous phase was extracted with 3 × 4 mL of ethyl acetate. The organic phases were combined and the solvent was removed under reduced pressure. The crude product was purified by flash chromatography with petroleum ether and ethyl acetate.

#### General Procedure 10: Fmoc Deprotection and Amide Coupling with HATU

68.0 μmol of the desired Fmoc protected central amino acid (1 equiv) was dissolved in 0.4 mL acetonitrile and 105 μL diethylamine (1 mmol, 15.1 equiv) at 0 °C and stirred for 1 h. The solvent was evaporated under reduced pressure. One ml acetonitrile was added to the residue and the solvent was removed under reduced pressure again. This was repeated twice. The crude residue was dried under high vacuum overnight [residue 1].

82.6 μmol of the desired fragment AB (1.2 equiv) and 78.9 μmol HATU (1.2 equiv) were added to a separate flask and dried under high vacuum. 0.5 mL dry DMF and 38 μL DIPEA (3 equiv) were added under nitrogen atmosphere and the reaction was stirred for 30 min. The solution was added to the residue [1] and stirred at 0 °C. The reaction was controlled over LCMS. After completion, the reaction was quenched with 8 mL of 0.1 M HCl and 4 mL brine. The inorganic layer was extracted with 3 × 6 mL of ethyl acetate. The organic phases were combined and washed with 2 × 5 mL brine. The solvent was removed under reduced pressure. The crude product was used without further purification.

#### General Procedure 11: Amide Coupling CDE to Central AA with IIDQ

0.09 mmol of the desired aniline (1 equiv) and 0.14 mmol of the desired carboxylic acid (1.5 equiv) were added to a dry flask and were further dried under high vacuum. 0.15 mL dry DCM was added under nitrogen atmosphere and the mixture was cooled down to 0 °C. 40 μL IIDQ (0.13 mmol, 1.5 equiv) dissolved in 0.15 mL dry DCM was added to the stirring solution. The reaction was kept at 0 °C for 30 min and slowly allowed to reach room temperature afterward. The reaction was stirred overnight and controlled over LCMS. The reaction was quenched with 2 mL 1 M HCl and 6 mL brine and extracted with 3 × 3 mL DCM. The combined organic phases were washed with brine and the solvent was removed under reduced pressure. The crude product was used in the next reaction.

##### *tert*-Butyl 4-[4-(4-Aminobenzamido)-2-(prop-2-en-1-yloxy)-3-(propan-2-yloxy)benzamido]benzoate (**8**)

The compound was prepared according to the established literature procedure; see ref (20).

##### 4-(4-Cyanobenzamido)benzoic Acid (**10**)

The compound was prepared according to the established literature procedure; see Dong, Y. et al.; *Bioorg. Med. Chem. Lett.***2014**, 24, 3, 944–948.

##### *tert*-Butyl 4-(4-{4-[(2*S*)-2-({[(9*H*-Fluoren-9-yl)methoxy]carbonyl}amino)pent-4-ynamido]benzamido}-2-(prop-2-en-1-yloxy)-3-(propan-2-yloxy)benzamido)benzoate (**9**)

After a suspension of the aniline **8** (1.53 g, 2.81 mmol, 1.00 equiv) in dry EtOAc (20.5 mL) was cooled down to 0 °C, (2*S*)-2-({[(9*H*-fluoren-9-yl)methoxy]carbonyl}amino)pent-4-ynoic acid (1.32 g, 3.94 mmol, 1.40 equiv) and dry pyridine (770 μL, 9.56 mmol, 3.40 equiv) were added. T3P solution (50% in EtOAc, 3.35 mL, 5.62 mmol, 2.00 equiv) was added dropwise over 15 min and the resulting solution was stirred for 2.5 h at 0 °C. The reaction mixture was diluted with aq HCl (1 M, 30 mL) and extracted with EtOAc (3 × 30 mL). The combined organic layers were washed with NaHCO_3_ solution (30 mL) and brine (30 mL), dried (Na_2_SO_4_) and concentrated *in vacuo*. The resulting residue was purified by flash chromatography (petroleum ether/EtOAc). Colorless solid, 2.11 g (87%).

^1^H NMR (500 MHz, CDCl_3_, 300 K): δ (ppm) = 10.16 (s, 1H), 8.74 (s, 1H), 8.49 (d, 1H, *J* = 8.9 Hz), 8.07 (d, 1H, *J* = 8.9 Hz), 7.98 (d, 2H, *J* = 8.8 Hz), 7.89 (d, 2H, *J* = 8.7 Hz), 7.77 (d, 2H, *J* = 7.5 Hz), 7.73 (d, 2H, *J* = 8.8 Hz), 7.69 (d, 2H, *J* = 8.8 Hz), 7.59 (d, 2H, *J* = 7.4 Hz), 7.40 (t, 2H, *J* = 7.5 Hz), 7.30 (t, 2H, *J* = 7.4 Hz), 6.14 (ddt, 1H, *J* = 5.9 Hz, 10.4 Hz, 17.1 Hz), 5.64–5.57 (m, 1H), 5.49 (dq, 1H, *J* = 1.4 Hz, 17.1 Hz), 5.41 (dq, 1H, *J* = 1.1 Hz, 10.4 Hz), 4.75 (hept., 1H, *J* = 6.2 Hz), 4.69 (dt, 2H, *J* = 1.2 Hz, 5.9 Hz), 4.57–4.46 (m, 3H), 4.25 (t, 1H, *J* = 6.7 Hz), 2.94–2.68 (m, 2H), 2.17 (t, 1H, *J* = 2.6 Hz), 1.60 (s, 9H), 1.38 (d, 6H, *J* = 6.2 Hz).^13^C NMR (126 MHz, CDCl_3_, 300 K): δ (ppm) = 168.5, 165.6, 164.3, 162.8, 156.7, 149.4, 143.6, 142.3, 141.5, 141.0, 139.1, 137.6, 132.3, 130.8, 130.4, 128.3, 128.0, 127.6, 127.5, 127.3, 125.1, 121.7, 120.3, 120.2, 120.0, 119.2, 115.8, 81.0, 79.0, 76.9, 75.1, 72.5, 67.7, 54.2, 47.2, 29.8, 28.4, 23.0.

##### 4-(4-{4-[(2*S*)-2-{[4-(4-Cyanobenzamido)phenyl]formamido}pent-4-ynamido]benzamido}-2-hydroxy-3-(propan-2-yloxy)benzamido)benzoic Acid (**13**)

Step 1: A solution of the Fmoc protected amine **9** (1.93 g, 2.24 mmol, 1.00 equiv) in ACN (9.7 mL) was cooled down to 0 °C and diethylamine (3.64 mL, 35.2 mmol, 15.75 equiv) was added. After stirring at 0 °C was continued for 1 h, all volatiles were removed under reduced pressure. The crude product was used without further purification. Step 2: The amino acid derivative (54.5 μmol) was coupled with the carboxylic acid **10** using general procedure 3. Step 3 and 4: The product was obtained by deprotection with general procedures 6 and 7. Yellowish solid, 10 mg (23% over 3 steps). ^1^H NMR (700 MHz, DMSO-*d*_6_, 300 K): δ (ppm) = 12.80 (br s, 1H), 12.30 (s, 1H), 10.71 (s, 1H), 10.58 (s, 1H), 9.40 (s, 1H), 8.77 (d, 1H, *J* = 7.5 Hz), 8.13 (d, 2H, *J* = 8.4 Hz), 8.05 (d, 2H, *J* = 8.4 Hz), 7.98–7.95 (m, 6H), 7.90 (d, 2H, *J* = 8.8 Hz), 7.87–7.84 (m, 3H), 7.83 (d, 2H, *J* = 8.8 Hz), 7.70 (d, 1H, *J* = 8.8 Hz), 4.81 (dd, 1H, *J* = 7.6 Hz, 14.7 Hz), 4.55 (hept., 1H, *J* = 6.1 Hz), 2.94 (t, 1H, *J* = 2.6 Hz), 2.79 (dddd, 2H, *J* = 2.6 Hz, 7.4 Hz, 11.1 Hz, 16.8 Hz), 1.27 (d, 6H, *J* = 6.1 Hz). ^13^C NMR (176 MHz, DMSO-*d*_6_, 300 K): δ (ppm) = 169.7, 168.5, 166.9, 166.0, 164.5, 164.2, 154.2, 142.2, 142.0, 141.7, 138.7, 137.0, 136.4, 132.5, 130.2, 128.9, 128.6, 128.5, 128.4, 128.4, 126.2, 122.8, 120.7, 119.5, 119.0, 118.3, 114.0, 112.1, 80.6, 74.8, 73.2, 53.5, 22.3, 21.4. HRMS (ESI) calcd 793.2622 [M + H^+^], 793.2617 found. HPLC purity 98.1%.

##### *tert*-Butyl 4-(4-{4-[(2*S*)-2-({[(9*H*-Fluoren-9-yl)methoxy]carbonyl}amino)-3-(1,3-thiazol-4-yl)propanamido]benzamido}-2-(prop-2-en-1-yloxy)-3-(propan-2-yloxy)benzamido)benzoate (**46**)

Amine **8** (0.09 mmol) was coupled with (2*S*)-2-({[(9*H*-Fluoren-9-yl)methoxy]carbonyl}amino)-3-(1,3-thiazol-4-yl)propanoic acid using general procedure 9. Yellow orange solid, 62 mg (74%).^1^H NMR (700 MHz, CDCl_3_, 300 K): δ (ppm) = 10.17 (s, 1H), 9.53 (s, 1H), 8.88 (s, 1H), 8.73 (s, 1H), 8.49 (d, 1H, *J* = 8.9 Hz), 8.06 (d, 1H, *J* = 8.9 Hz), 7.98 (d, 2H, *J* = 8.7 Hz), 7.87 (d, 2H, *J* = 8.7 Hz), 7.79–7.75 (m, 2H), 7.73 (d, 2H, *J* = 8.7 Hz), 7.69 (d, 2H, *J* = 7.9 Hz), 7.61–7.57 (m, 2H), 7.43–7.38 (m, 2H), 7.33–7.28 (m, 2H), 7.20 (s, 1H), 6.51 (d, 1H, *J* = 5.6 Hz), 6.14 (ddt, 1H, *J* = 5.9 Hz, 10.4 Hz, 16.3 Hz), 5.49 (dd, 1H, *J* = 1.3 Hz, 17.1 Hz), 5.40 (dd, 1H, *J* = 1.1 Hz, 10.4 Hz), 4.77–4.72 (m, 2H), 4.69 (d, 2H, *J* = 5.9 Hz), 4.49–4.39 (m, 2H), 4.23 (t, 1H, *J* = 6.6 Hz), 3.46–3.34 (m, 2H), 1.60 (s, 9H), 1.38 (d, 6H, *J* = 6.1 Hz).^13^C NMR (176 MHz, CDCl_3_, 300 K): δ (ppm) = 169.6, 165.6, 164.4, 162.8, 156.7, 153.6, 152.6, 149.4, 143.8, 142.3, 141.5, 141.5 141.5, 139.1, 137.7, 132.3, 130.8, 130.0, 128.2, 128.0, 127.6, 127.4, 127.3, 125.2, 121.7, 120.2, 120.2, 119.8, 119.2, 116.5, 115.8, 80.9, 76.9, 75.1, 67.5, 55.3, 47.3, 33.4, 28.4, 23.0. HRMS (ESI) calcd 922.3486 [M + H^+^], 922.3456 found. HPLC purity 95.9%.

##### 4-(4-{4-[(2*S*)-2-{[4-(4-Cyanobenzamido)phenyl]formamido}-3-(1,3-thiazol-4-yl)propanamido]benzamido}-2-hydroxy-3-(propan-2-yloxy)benzamido)benzoic Acid (**14**)

The Fmoc protected amino acid **46** (67.5 μmol) was deprotected and coupled with carboxylic acid **10** using general procedure 10. The product was obtained by deprotection with general procedures 5 and 7. White solid, 9 mg (16% over 3 steps). ^1^H NMR (700 MHz, DMSO-*d*_6_, 300 K): δ (ppm) = 12.81 (br s, 1H), 12.30 (s, 1H), 10.69 (s, 1H), 10.62 (br s, 1H), 10.56 (s, 1H), 9.40 (s, 1H), 9.06 (d, 1H, *J* = 1.9 Hz), 8.73 (d, 1H, *J* = 7.6 Hz), 8.12 (d, 2H, *J* = 8.5 Hz), 8.04 (d, 2H, *J* = 8.5 Hz), 7.98–7.94 (m, 4H), 7.90–7.87 (m, 4H), 7.87–7.84 (m, 3H), 7.82 (d, 2H, *J* = 8.8 Hz), 7.71 (d, 1H, *J* = 8.8 Hz), 7.49 (d, 1H, *J* = 1.9 Hz), 5.02 (dd, 1H, *J* = 7.8 Hz, 14.5 Hz), 4.55 (hept., 1H, *J* = 6.1 Hz), 3.40–3.34 (m, 2H), 1.27 (d, 6H, *J* = 6.1 Hz). ^13^C NMR (176 MHz, DMSO-*d*_6_, 300 K): δ (ppm) = 170.8, 168.5, 166.9, 165.9, 164.5, 164.2, 154.2, 153.8, 153.1, 142.4, 142.0, 141.6, 138.7, 137.0, 136.3, 132.5, 130.2, 129.1, 128.6, 128.4, 128.3, 126.3, 122.8, 120.7, 119.5, 119.0, 118.3, 115.9, 114.0, 112.4, 112.1, 74.8, 54.3, 32.9, 22.3. HRMS (ESI) calcd 852.2452 [M + H^+^], 852.2448 found. HPLC purity 95.7%.

##### *tert*-Butyl (2*S*)-3-(Dimethylcarbamoyl)-2-({[(9*H*-fluoren-9-yl)methoxy]carbonyl}amino)propanoate (**49**)

200 mg (3*S*)-4-(*tert*-butoxy)-3-({[(9*H*-fluoren-9-yl)methoxy]carbonyl}amino)-4-oxobutanoic acid (0.49 mmol, 1 equiv) and 185 mg HATU (0.49 mmol, 1.0 equiv) were added to a dry flask and further dried under high vacuum. The flask was cooled to 0 °C. 3.5 mL dry DMF and 0.09 mL DIPEA (66.8 mg, 0.52 mmol, 1.1 equiv) were added under nitrogen atmosphere. The reaction was stirred for 30 min at 0 °C. 0.27 mL 2 M dimethylamine in THF (0.54 mmol, 1.1 equiv) was added to the stirring solution. The mixture was stirred at 0 °C for the whole reaction. The reaction was controlled over TLC. After completion, 8 mL of 0.1 M HCl and 6 mL brine were added. The aqueous layer was extracted with 3 × 5 mL of ethyl acetate. The organic phases were combined and washed with 2 × 5 mL brine. The crude product was purified by flash chromatography (petroleum ether/ethyl acetate) and used directly in the next reaction. 239 mg (crude). HRMS (ESI) calcd 439.2233 [M + H^+^], 439.2223 found.

##### (2*S*)-3-(Dimethylcarbamoyl)-2-({[(9*H*-fluoren-9-yl)methoxy]carbonyl}amino)propanoic Acid (**50**)

238 mg ester **49** (0.54 mmol, 1 equiv) was dissolved in 6 mL dry DCM under nitrogen atmosphere. 0.77 mL TFA (1147 mg, 10.1 mmol, 22.0 equiv) was added to the stirring mixture. The reaction was controlled over LCMS. After completion, the solvent was removed under reduced pressure. The excess of TFA was removed by coevaporation with DCM. 166 mg (89% over 2 steps). ^1^H NMR (700 MHz, DMSO-*d*_6_, 300 K): δ (ppm) = 7.89 (d, 2H, *J* = 7.5 Hz), 7.71 (d, 2H, *J* = 7.5 Hz), 7.42 (t, 2H, *J* = 7.5 Hz), 7.36 (d, 1H, *J* = 8.4 Hz), 7.33 (t, 2H, *J* = 7.4 Hz), 4.42–4.37 (m, 1H), 4.29 (dd, 2H, *J* = 3.0 Hz, 7.0 Hz), 4.22 (t, 1H, *J* = 7.0 Hz), 2.94 (s, 3H), 2.82 (s, 3H), 2.77 (dd, 1H, *J* = 7.3 Hz, 16.4 Hz), 2.72–2.69 (m, 1H). ^13^C NMR (176 MHz, DMSO-*d*_6_, 300 K): δ (ppm) = 173.2, 169.2, 155.8, 143.8, 140.7, 127.6, 127.1, 125.3, 120.1, 65.7, 50.5, 46.6, 38.3, 36.6, 34.9, 34.6. HRMS (ESI) calcd 383.1607 [M + H^+^], 383.1601 found. HPLC purity 95.1%.

##### *tert*-Butyl 4-(4-{4-[(2*S*)-2-Amino-3-(dimethylcarbamoyl)propanamido]benzamido}-2-(prop-2-en-1-yloxy)-3-(propan-2-yloxy)benzamido)benzoate (**51**)

Amine **8** (0.09 mmol) was coupled with carboxylic acid **50** using general procedure 9 and deprotected using general procedure 2. Faint yellow solid, 18 mg (29% over 2 steps). ^1^H NMR (500 MHz, CDCl_3_, 300 K): δ (ppm) = 10.16 (s, 1H), 8.73 (s, 1H), 8.48 (d, 1H, *J* = 8.9 Hz), 8.05 (d, 1H, *J* = 8.9 Hz), 7.97 (d, 2H, *J* = 8.8 Hz), 7.87 (d, 2H, *J* = 8.8 Hz), 7.79 (d, 2H, *J* = 8.5 Hz), 7.72 (d, 2H, *J* = 8.8 Hz), 6.14 (ddt, 1H, *J* = 5.9 Hz, 10.4 Hz, 16.3 Hz), 5.49 (ddd, 1H, *J* = 1.4 Hz, 2.8 Hz, 17.1 Hz), 5.40 (ddd, 1H, *J* = 1.0 Hz, 2.1 Hz, 10.4 Hz), 4.74 (hept., 1H, *J* = 6.2 Hz), 4.69 (d, 2H, *J* = 5.9 Hz), 3.97 (s), 3.05–2.87 (m, 8H), 1.59 (s, 9H), 1.37 (d, 6H, *J* = 6.2 Hz). ^13^C NMR (126 MHz, CDCl_3_, 300 K): δ (ppm) = 172.4, 170.8, 165.5, 164.5, 162.8, 149.4, 142.3, 141.7, 139.1, 137.8, 132.3, 130.8, 129.6, 128.2, 127.6, 127.4, 121.6, 120.1, 119.4, 119.1, 115.8, 80.9, 76.9, 75.1, 52.5, 37.5, 37.3, 35.6, 28.4, 22.9. HRMS (ESI) calcd 688.3346 [M + H^+^], 688.3345 found. HPLC purity 98.4%.

##### 4-(4-{4-[(2*S*)-2-{[4-(4-Cyanobenzamido)phenyl]formamido}-3-(dimethylcarbamoyl)propanamido]benzamido}-2-hydroxy-3-(propan-2-yloxy)benzamido)benzoic Acid (**15**)

The amine **51** (25.6 μmol) was coupled with carboxylic acid **10** using general procedure 4. The product was obtained by deprotection with general procedures 5 and 7. White solid, 7 mg (34% over 3 steps). ^1^H NMR (700 MHz, DMSO-*d*_6_, 300 K): δ (ppm) = 12.83 (br s, 1H), 12.29 (s, 1H), 10.70 (s, 1H), 10.60 (s, 1H), 10.46 (s, 1H), 9.39 (s, 1H), 8.62 (d, 1H, *J* = 7.1 Hz), 8.13 (d, 2H, *J* = 8.4 Hz), 8.04 (d, 2H, *J* = 8.4 Hz), 7.97 (d, 2H, *J* = 8.7 Hz), 7.95 (d, 2H, *J* = 8.8 Hz), 7.93 (d, 2H, *J* = 8.8 Hz), 7.89 (d, 2H, *J* = 8.8 Hz), 7.87–7.84 (m, 3H), 7.83 (d, 2H, *J* = 8.7 Hz), 7.70 (d, 1H, *J* = 8.8 Hz), 5.00 (quart., 1H, *J* = 6.9 Hz), 4.54 (hept., 1H, *J* = 6.1 Hz), 3.01 (s, 3H), 2.95–2.88 (m, 2H), 2.85 (s, 3H), 1.26 (d, 6H, *J* = 6.1 Hz). ^13^C NMR (176 MHz, DMSO-*d*_6_, 300 K): δ (ppm) = 170.8, 169.2, 168.5, 166.9, 165.7, 164.4, 164.2, 154.1, 142.7, 142.0, 141.6, 138.7, 137.1, 136.3, 132.5, 130.2, 129.2, 128.6, 128.4, 128.3, 128.2, 126.3, 122.8, 120.7, 119.5, 118.9, 118.3, 114.0, 112.4, 112.2, 74.9, 51.6, 36.6, 34.9, 34.5, 22.3. HRMS (ESI) calcd 840.2993 [M + H^+^], 840.2988 found. HPLC purity 98.8%.

##### (2*R*)-2-({[(9*H*-Fluoren-9-yl)methoxy]carbonyl}amino)-3-oxo-3-(prop-2-en-1-yloxy)propane-1-sulfonic Acid (**54**)

200.0 mg carboxylic acid **53** (0.51 mmol, 1 equiv) was added to a dry vial and further dried at high vacuum. Two ml allyl alcohol (1.71 g, 29.4 mmol, 57.6 equiv) was added and the vial was cooled down to 0 °C. 0.23 mL chlorotrimethylsilane (1.81 mmol, 3.6 equiv) was added under argon atmosphere and the reaction was slowly allowed to warm up to room temperature. The reaction was stirred overnight and controlled by LCMS. After completion, the solvent was removed under reduced pressure. The residue was coevaporated with *n*-heptane. The crude product was dried under high vacuum. White solid, 221 mg (quant.). ^1^H NMR (500 MHz, DMSO-*d*_6_, 300 K): δ (ppm) = 7.89 (d, 2H, *J* = 7.5 Hz), 7.69 (dd, 2H, *J* = 2.4 Hz, 7.4 Hz), 7.55 (d, 1H, *J* = 7.0 Hz), 7.42 (t, 2H, *J* = 7.4 Hz), 7.33 (tdd, 2H, *J* = 1.1 Hz, 2.5 Hz, 7.4 Hz), 5.89 (ddt, 1H, *J* = 5.3 Hz, 10.6 Hz, 17.3 Hz), 5.31 (dq, 1H, *J* = 1.7 Hz, 17.3 Hz), 5.17 (dq, 1H, *J* = 1.5 Hz, 10.6 Hz), 4.56–4.53 (m, 2H), 4.37–4.33 (m, 1H), 4.29–4.23 (m, 3H), 2.87 (ddd, 2H, *J* = 5.7 Hz, 13.8 Hz, 18.2 Hz). ^13^C NMR (126 MHz, DMSO-*d*_6_, 300 K): δ (ppm) = 170.6, 155.6, 143.7, 140.7, 132.6, 127.7, 127.1, 125.2, 120.1, 117.5, 65.9, 64.9, 51.5, 50.7, 46.6. HPLC purity 96.7%.

##### Prop-2-en-1-yl (2*R*)-3-{Bis[(4-methoxyphenyl)methyl]sulfamoyl}-2-({[(9*H*-fluoren-9-yl)methoxy]carbonyl}amino)propanoate (**55**)

210 mg sulfonic acid **54** (0.49 mmol, 1 equiv) was added to a dry vial and further dried under high vacuum. 1.6 mL dry DCM and 2 drops of dry DMF were added under argon atmosphere. The mixture was cooled to 0 °C and 65 μL oxalyl chloride (0.76 mmol, 1.56 equiv) was slowly added to the stirring mixture. The reaction was warmed up to room temperature and stirred for 2.5 h. The solvent was concentrated under reduced pressure and the residue was dried under high vacuum overnight. 150 mg bis(4-methoxybenzyl)amine (0.58 mmol, 1.2 equiv) and 1.6 mL dry DCM were added under argon atmosphere and the mixture was cooled down to 0 °C. 0.14 mL dry triethylamine (1.0 mmol, 2.1 equiv) was added and stirring was continued at 0 °C. After completion, 60 μL acetic acid (63 mg, 1.1 mmol, 2.2 equiv) and 1 mL acetone were added and the solution was concentrated under reduced pressure. The product was directly purified by chromatography. White solid, 158.4 mg (49%). ^1^H NMR (500 MHz, CDCl_3_, 300 K): δ (ppm) = 7.76 (d, 2H, *J* = 7.5 Hz), 7.62 (d, 2H, *J* = 6.8 Hz), 7.39 (t, 2H, *J* = 7.5 Hz), 7.31 (tdd, 2H, *J* = 0.9 Hz, 3.2 Hz, 7.4 Hz), 7.21 (d, 4H, *J* = 8.6 Hz), 6.88 (d, 4H, *J* = 8.7 Hz), 5.98–5.89 (m, 2H), 5.35 (dd, 1H, *J* = 1.3 Hz, 17.2 Hz), 5.26 (ddd, 1H, *J* = 1.1 Hz, 2.3 Hz, 10.5 Hz), 4.75–4.69 (m, 3H), 4.42–4.34 (m, 2H), 4.24 (s, 4H), 3.80 (s, 6H), 3.41 (ddd, 2H, *J* = 5.0 Hz, 14.2 Hz, 18.5 Hz). ^13^C NMR (126 MHz, CDCl_3_, 300 K): δ (ppm) = 169.0, 159.6, 155.9, 143.9, 143.9, 141.4, 131.5, 130.3, 127.9, 127.3, 125.4, 120.1, 119.5, 114.4, 67.8, 67.1, 55.5, 54.0, 50.8, 49.2, 47.2.

##### (2*R*)-3-{Bis[(4-methoxyphenyl)methyl]sulfamoyl}-2-({[(9*H*-fluoren-9-yl)methoxy]carbonyl}amino)propanoic Acid (**56**)

200 mg ester **55** (0.3 mmol, 1 equiv) was added to a dry vial and further dried under high vacuum. Two mL dry THF, 110 μL phenylsilane (0.9 mmol, 3 equiv) and 6.9 mg tetrakis(triphenylphosphine)palladium(0) (6 μmol, 0.02 equiv) were added under argon atmosphere. The reaction was stirred for 3 h at room temperature and controlled over TLC. After completion, 0.53 mL saturated NaHCO_3_ solution (0.6 mmol, 2 equiv) was added and the mixture was stirred for 30 min. All volatiles were removed under reduced pressure. Twelve mL brine and 2 mL 1 M HCl was added. The aqueous layer was extracted with 3 × 6 mL ethyl acetate. The combined organic layers were concentrated under reduced pressure and the crude product was purified by flash chromatography (DCM/MeOH). The crude product was used without further purification.

##### *tert*-Butyl 4-(4-{4-[(2*R*)-2-Amino-3-{bis[(4-methoxyphenyl)methyl]sulfamoyl}propanamido]benzamido}-2-(prop-2-en-1-yloxy)-3-(propan-2-yloxy)benzamido)benzoate (**57**·HCl)

Aniline **8** (0.20 mol) was coupled with carboxylic acid **56** using general procedure 1 and deprotected by general procedure 2. The crude product was precipitated from diethyl ether with 2 M HCl in diethyl ether. The solid was suspended in 20 mL saturated NaHCO_3_ solution and extracted with 4 × 6 mL DCM. The combined organic layers were concentrated under reduced pressure and dried under high vacuum. Yellowish solid, 117.5 mg (62%).^1^H NMR (500 MHz, CDCl_3_, 300 K): δ (ppm) = 10.17 (s, 1H), 9.92 (s, 1H), 8.75 (s, 1H), 8.50 (d, 1H, *J* = 8.9 Hz), 8.07 (d, 1H, *J* = 8.9 Hz), 7.98 (d, 2H, *J* = 8.7 Hz), 7.91 (d, 2H, *J* = 8.7 Hz), 7.77 (d, 2H, *J* = 8.7 Hz), 7.75–7.72 (m, 3H), 7.29 (d, 1H, *J* = 8.6 Hz), 7.22 (d, 4H, *J* = 8.7 Hz), 6.91–6.88 (m, 4H), 6.14 (ddt, 1H, *J* = 5.9 Hz, 10.4 Hz, 16.3 Hz), 5.50 (dd, 1H, *J* = 1.3 Hz, 17.1 Hz), 5.41 (dd, 1H, *J* = 1.1 Hz, 10.4 Hz), 4.75 (hept., 1H, *J* = 6.2 Hz), 4.69 (d, 2H, *J* = 5.9 Hz), 4.34 (d, 2H, *J* = 15.1 Hz), 4.24 (d, 2H, *J* = 15.2 Hz), 3.81 (s, 6H), 3.08–3.03 (m, 1H), 1.60 (s, 9H), 1.38 (d, 6H, *J* = 6.2 Hz). ^13^C NMR (126 MHz, CDCl_3_, 300 K): δ (ppm) = 170.4, 165.6, 164.3, 162.8, 159.6, 149.4, 142.3, 141.0, 139.1, 137.7, 132.3, 130.8, 130.3, 128.4, 127.7, 127.4, 127.3, 121.7, 120.2, 119.5, 119.1, 115.8, 114.4, 114.1, 80.9, 75.1, 55.5, 49.3, 28.4, 23.0.

##### 4-(4-{4-[(2*R*)-2-{[4-(4-Cyanobenzamido)phenyl]formamido}-3-sulfamoylpropanamido]benzamido}-2-hydroxy-3-(propan-2-yloxy)benzamido)benzoic Acid (**16**)

The amine **57**·HCl (117.5 μmol) was coupled with carboxylic acid **10** using general procedure 4. The product was obtained by deprotection with general procedures 6 and 7. Off-white solid, 34.4 mg (35% over 3 steps). ^1^H NMR (700 MHz, DMSO-*d*_6_, 300 K): δ (ppm) = 12.72 (br s, 1H), 12.29 (br s, 1H), 10.71 (s, 1H), 10.43 (s, 1H), 9.40 (s, 1H), 8.83 (d, 1H, *J* = 7.3 Hz), 8.13 (d, 2H, *J* = 8.5 Hz), 8.05 (d, 2H, *J* = 8.5 Hz), 7.98–7.95 (m, 4H), 7.94 (d, 2H, *J* = 8.8 Hz), 7.90 (d, 2H, *J* = 8.8 Hz), 7.87–7.83 (m, 3H), 7.81 (d, 2H, *J* = 8.8 Hz), 7.68 (d, 1H, *J* = 8.6 Hz), 7.03 (br s, 2H), 5.05 (dd, 1H, *J* = 7.5 Hz, 12.6 Hz), 4.55 (quint., 1H, *J* = 5.9 Hz), 3.65 (ddd, 2H, *J* = 6.4 Hz, 14.3 Hz, 22.2 Hz), 1.26 (d, 6H, *J* = 6.1 Hz). ^13^C NMR (176 MHz, DMSO-*d*_6_, 300 K): δ (ppm) = 168.7, 168.5, 166.9, 166.0, 164.5, 164.2, 142.1, 141.7, 138.7, 136.9, 136.4, 132.5, 130.2, 129.0, 128.7, 128.6, 128.5, 128.3, 126.2, 122.9, 120.6, 119.5, 119.4, 118.3, 114.1, 112.6, 74.7, 55.1, 50.9, 22.3. HRMS (ESI) calcd 848.2345 [M + H^+^], 848.2335 found. HPLC purity 97.7%.

##### *tert*-Butyl 4-(4-{4-[(2*S*)-Piperidine-2-amido]benzamido}-2-(prop-2-en-1-yloxy)-3-(propan-2-yloxy)benzamido)benzoate (**59**)

Amine **8** (0.09 mmol) was coupled with (2*S*)-1-{[(9*H*-Fluoren-9-yl)methoxy]carbonyl}piperidine-2-carboxylic acid using general procedure 9 and deprotected using general procedure 2. Yellowish solid, 24 mg (39% over 2 steps). ^1^H NMR (700 MHz, CDCl_3_, 300 K): δ (ppm) = 10.17 (s, 1H), 9.47 (s, 1H), 8.73 (s, 1H), 8.46 (d, 1H, *J* = 8.9 Hz), 8.04 (d, 1H, *J* = 8.9 Hz), 7.97 (d, 2H, *J* = 8.7 Hz), 7.86 (d, 2H, *J* = 8.7 Hz), 7.76 (d, 2H, *J* = 8.7 Hz), 7.72 (d, 2H, *J* = 8.7 Hz), 6.14 (ddt, 1H, *J* = 5.9 Hz, 10.5 Hz, 16.3 Hz), 5.49 (d, 1H, *J* = 1.3 Hz, 17.1 Hz), 5.40 (dd, 1H, *J* = 1.1 Hz, 10.4 Hz), 4.74 (quart., 1H, *J* = 6.1 Hz), 4.69 (d, 2H, *J* = 5.9 Hz), 3.52 (d, 1H, *J* = 7.8 Hz), 3.12 (d, 1H, *J* = 12.1 Hz), 2.81 (t, 1H, *J* = 11.1 Hz), 2.06 (dd, 1H, *J* = 2.9 Hz, 13.0 Hz), 1.85–1.81 (m, 1H), 1.67–1.63 (m, 2H), 1.59 (s, 9 H), 1.53–1.49 (m, 2H), 1.37 (d, 6H, *J* = 6.2 Hz). ^13^C NMR (176 MHz, CDCl_3_, 300 K): δ (ppm) = 172.0, 165.5, 164.4, 162.8, 149.4, 142.3, 141.7, 139.1, 137.7, 132.2, 130.8, 129.6, 128.2, 127.6, 127.4, 121.6, 120.1, 119.5, 119.2, 115.8, 80.9, 76.9, 75.1, 60.1, 45.4, 29.2, 28.4, 25.5, 23.5, 23.0. HRMS (ESI) calcd 657.3288 [M + H^+^], 657.3293 found. HPLC purity 99.3%.

##### 4-(4-{4-[(2*S*)-1-[4-(4-Cyanobenzamido)benzoyl]piperidine-2-amido]benzamido}-2-hydroxy-3-(propan-2-yloxy)benzamido)benzoic Acid (**17**)

The amine **59** (35.5 μmol) was coupled with carboxylic acid **10** using general procedure 4. The product was obtained by deprotection with general procedures 5 and 7. White solid, 9 mg (31% over 3 steps). ^1^H NMR (700 MHz, DMSO-*d*_6_, 300 K): δ (ppm) = 12.82 (br s, 1H), 12.30 (s, 1H), 10.65 (s, 1H), 10.61 (s, 1H), 10.38 (s, 1H), 9.42 (s, 1H), 8.11 (d, 2H, *J* = 7.6 Hz), 8.04 (d, 2H, *J* = 8.2 Hz), 7.99–7.96 (m, 4H), 7.89–7.85 (m, 5H), 7.84–7.79 (m, 2H), 7.71 (d, 1H, *J* = 8.8 Hz), 7.50–7.43 (m, 1H), 5.24 (br s, 1H), 4.55 (hept., 1H, *J* = 6.1 Hz), 3.67–3.53 (m, 2H), 2.26–2.17 (m, 1H), 1.90–1.82 (m, 1H), 1.70 (dd, 1H, *J* = 3.2 Hz, 9.3 Hz), 1.52–1.42 (m, 2H), 1.27 (d, 6H, *J* = 6.1 Hz), 1.25–1.21 (m, 2H). ^13^C NMR (176 MHz, DMSO-*d*_6_, 300 K): δ (ppm) = 170.6, 168.5, 166.9, 164.4, 164.2, 154.1, 142.4, 142.0, 139.9, 138.8, 137.0, 136.3, 132.5, 130.2, 128.6, 128.4, 127.8, 126.3, 122.8, 120.7, 120.0, 119.0, 118.3, 114.0, 112.4, 112.2, 74.9, 53.1, 45.7, 28.7, 27.3, 22.3, 20.2. HRMS (ESI) calcd 809.2935 [M + H^+^], 809.2930 found. HPLC purity 99.0%.

##### *tert*-Butyl 4-(4-{4-[(2*R*)-Piperidine-2-amido]benzamido}-2-(prop-2-en-1-yloxy)-3-(propan-2-yloxy)benzamido)benzoate (**61**)

Amine **8** (0.09 mmol) was coupled with (2*R*)-1-{[(9*H*-Fluoren-9-yl)methoxy]carbonyl}piperidine-2-carboxylic acid using general procedure 9 and deprotected using general procedure 2. White solid, 11 mg (18% over 2 steps). ^1^H NMR (700 MHz, CDCl_3_, 300 K): δ (ppm) = 10.17 (s, 1H), 9.36 (s, 1H), 8.74 (s, 1H), 8.48 (d, 1H, *J* = 8.9 Hz), 8.05 (d, 1H, *J* = 8.9 Hz), 7.98 (d, 2H, *J* = 8.7 Hz), 7.88 (d, 2H, *J* = 8.7 Hz), 7.77 (d, 2H, *J* = 8.7 Hz), 7.73 (d, 2H, *J* = 8.8 Hz), 6.14 (ddt, 1H, *J* = 5.9 Hz, 10.4 Hz, 16.3 Hz), 5.49 (dd, 1H, *J* = 1.3 Hz, 17.1 Hz), 5.40 (dd, 1H, *J* = 1.0 Hz, 10.4 Hz), 4.75 (hept., 1H, *J* = 6.1 Hz), 4.69 (d, 2H, *J* = 5.9 Hz), 3.47 (d, 1H, *J* = 7.4 Hz), 3.10 (d, 1H, *J* = 12.1 Hz), 2.83–2.77 (m, 1H), 2.05 (dd, 1H, *J* = 3.3 Hz, 13.1 Hz), 1.86–1.80 (m, 1H), 1.67–1.63 (m, 2H), 1.60 (s, 9 H), 1.53–1.49 (m, 2H), 1.38 (d, 6H, *J* = 6.1 Hz). ^13^C NMR (176 MHz, CDCl_3_, 300 K): δ (ppm) = 172.3, 165.6, 164.4, 162.8, 149.4, 142.3, 141.7, 139.1, 137.7, 132.2, 130.8, 129.7, 128.3, 127.6, 127.4, 121.6, 120.2, 119.4, 119.2, 115.8, 80.9, 76.9, 75.1, 60.3, 45.5, 29.4, 28.4, 25.7, 23.6, 23.0. HRMS (ESI) calcd 657.3288 [M + H^+^], 657.3281 found. HPLC purity 99.9%.

##### 4-(4-{4-[(2*R*)-1-[4-(4-Cyanobenzamido)benzoyl]piperidine-2-amido]benzamido}-2-hydroxy-3-(propan-2-yloxy)benzamido)benzoic Acid (**18**)

The amine **61** (15.2 μmol) was coupled with carboxylic acid **10** using general procedure 4. The product was obtained by deprotection with general procedures 5 and 7. Beige solid, 7 mg (57% over 3 steps). ^1^H NMR (700 MHz, DMSO-*d*_6_, 300 K): δ (ppm) = 12.82 (br s, 1H), 12.30 (s, 1H), 10.66 (s, 1H), 10.61 (s, 1H), 10.38 (br s, 1H), 9.42 (s, 1H), 8.11 (d, 2H, *J* = 7.7 Hz), 8.04 (d, 2H, *J* = 8.2 Hz), 7.98–7.96 (m, 4H), 7.89–7.84 (m, 5H), 7.84–7.80 (m, 2H), 7.71 (d, 1H, *J* = 8.8 Hz), 7.50–7.43 (m, 2H), 5.24 (br s, 1H), 4.55 (hept., 1H, *J* = 6.1 Hz), 2.25–2.20 (m, 1H), 1.90–1.81 (m, 1H), 1.70 (dd, 1H, *J* = 3.1 Hz, 9.4 Hz), 1.50–1.43 (m, 2H), 1.27 (d, 6H, *J* = 6.1 Hz), 1.25–1.21 (m, 3H). ^13^C NMR (176 MHz, DMSO-*d*_6_, 300 K): δ (ppm) = 170.6, 168.5, 166.9, 164.4, 164.2, 154.1, 142.4, 142.0, 139.9, 138.8, 137.0, 136.3, 132.5, 130.2, 128.6, 128.4, 127.8, 126.3, 122.8, 120.7, 120.0, 119.0, 118.3, 114.0, 112.4, 112.2, 74.9, 53.1, 45.7, 28.7, 27.3, 22.3, 20.2. HRMS (ESI) calcd 809.2935 [M + H^+^], 809.2929 found. HPLC purity 98.4%.

##### *tert*-Butyl (2*S*)-2-[(4-{[4-({4-[(*tert*-Butoxy)carbonyl]phenyl}carbamoyl)-3-(prop-2-en-1-yloxy)-2-(propan-2-yloxy)phenyl]carbamoyl}phenyl)carbamoyl]-4-oxopiperidine-1-carboxylate (**63**)

50 mg Aniline **8** (0.09 mmol, 1 equiv) and 26.8 mg (*S*)-1-(*tert*-butoxycarbonyl)-4-oxopiperidine-2-carboxylic acid (0.11 mmol, 1.2 equiv) were added to a dry flask and further dried under high vacuum. 0.64 mL dry DCM and 38 μL triethylamine (27.6 mg, 0.27 mmol, 3 equiv) were added under nitrogen atmosphere and the solution was cooled down to 0 °C. 10.0 μL phosphoryl chloride (16.5 mg, 0.11 mmol, 1.2 equiv) was slowly added and the mixture was kept at 0 °C. The reaction was controlled over LCMS. After completion, the reaction was quenched with 4 mL water and 1 mL 1 M HCl. The aqueous phase was extracted with 3 × 4 mL of DCM. The organic phases were combined and the solvent was removed under reduced pressure. The crude product was purified by flash chromatography (petroleum ether/ethyl acetate) and directly used without further purification. 106 mg (crude).

##### *tert*-Butyl 4-(4-{4-[(2*S*)-4-Oxopiperidine-2-amido]benzamido}-2-(prop-2-en-1-yloxy)-3-(propan-2-yloxy)benzamido)benzoate (**64**)

70 mg crude carbamate **63** (0.09 mmol, 1 equiv) was dissolved in 0.4 mL *tert*-butyl acetate (3.0 mmol, 32.6 equiv) and 0.1 mL dry DCM under nitrogen atmosphere. Sixteen μL trifluoromethanesulfonic acid (27.1 mg, 0.18 mmol, 2.0 equiv) was slowly added to the stirring solution under nitrogen atmosphere. The reaction was controlled over LCMS. After completion, the reaction was quenched with Na_2_CO_3_ and 4 mL water. The aqueous phase was extracted with 3 × 2 mL ethyl acetate. The solvent was removed under reduced pressure and the residue was purified by RP HPLC. 9.5 mg (crude). HRMS (ESI) calcd 671.3081 [M + H^+^], 671.3075 found.

##### 4-(4-{4-[(2*S*)-1-[4-(4-Cyanobenzamido)benzoyl]-4-oxopiperidine-2-amido]benzamido}-2-hydroxy-3-(propan-2-yloxy)benzamido)benzoic Acid (**19**)

The amine **64** (14.0 μmol) was coupled with carboxylic acid **10** using general procedure 4. The product was obtained by deprotection with general procedures 6 and 7. White solid, 3 mg (25% over 3 steps). ^1^H NMR (700 MHz, DMSO-*d*_6_, 300 K): δ (ppm) = 12.82 (br s, 1H), 12.29 (s, 1H), 10.68 (s, 1H), 10.60 (s, 1H), 10.58 (1H), 9.43 (s, 1H), 8.12 (d, 2H, *J* = 8.3 Hz), 8.05 (d, 2H, *J* = 8.4 Hz), 7.99–7.96 (m, 4H), 7.89 (d, 2H, *J* = 8.1 Hz), 7.87–7.84 (m, 3H), 7.81–7.77 (m, 2H), 7.70 (d, 1H, *J* = 8.8 Hz), 7.55 (d, 2H, *J* = 6.8 Hz), 5.15 (br s, 1H), 4.54 (hept., 1H, *J* = 6.1 Hz), 3.99–3.91 (m, 1H), 3.88–3.83 (m, 1H), 3.09 (dd, 1H, *J* = 6.9 Hz, 15.8 Hz), 2.75 (d, 1H, *J* = 12.5 Hz), 2.54 (dd, 2H, *J* = 4.5 Hz, 6.4 Hz), 1.27 (d, 6H, *J* = 6.1 Hz). ^13^C NMR (176 MHz, DMSO-*d*_6_, 300 K): δ (ppm) = 206.3, 170.1, 170.0, 168.5, 166.9, 164.5, 164.2, 154.1,142.0, 140.4, 138.7, 137.0, 136.4, 132.5, 130.7, 130.2, 128.6, 128.4, 128.1, 126.3, 122.8, 120.7, 119.9, 119.0, 118.3, 114.0, 112.5, 112.3, 74.9, 55.0, 43.4, 41.1, 39.5, 22.3. HRMS (ESI) calcd 823.2728 [M + H^+^], 823.2722 found. HPLC purity 97.6%.

##### Methyl (2*S*)-2-[(2,2-Dimethoxyethyl)({[(9*H*-fluoren-9-yl)methoxy]carbonyl})amino]-3-hydroxypropanoate (**67**)

Methyl l-serinate hydrochloride (**66**·HCl) (500 mg, 3.21 mmol) was dissolved in MeOH (10 mL). Et_3_N (450 μL, 3.21 mmol, 1.00 equiv), 2,2-dimethoxyacetaldehyde (60% in H_2_O, 558 mg, 3.21 mmol, 1.00 equiv) and 10% Pd/C (45.0 mg) were added subsequently. The mixture was stirred under an H_2_ atmosphere for 17 h before filtration through a short plug of Celite. The filtrate was concentrated under reduced pressure. The crude product was dissolved in H_2_O (6 mL) and NaHCO_3_ (540 mg, 6.42 mmol, 2.00 equiv) and FmocCl (815 mg, 3.15 mmol, 1.00 equiv) were added. The mixture was diluted with EtOAc (7 mL) at 0 °C. After stirring for 1 h at 0 °C the mixture was warmed to rt and stirring was continued for 21 h. EtOAc was added and the phases were separated. The organic phase was washed with a 1 M HCl solution, brine, dried over MgSO_4_, filtered and concentrated under reduced pressure. The crude product was purified by column chromatography (dry load, PE/EtOAc = 2:1) to furnish tertiary amine (1.16 g, 2.69 mmol, 84%) as colorless oil. The analytical data are consistent with those reported in the literature (F. Sladojevich, A. Trabocchi, A. Guarna, *J. Org. Chem.***2007**, 72, 4254–4257). [α]_D_^24^ = −29.1° (c 1.1Cl_3_). ^1^H NMR (400 MHz, CDCl_3_) = δ (3:2 mixture of rotamers) 7.78–7.76 (d, *J* = 7.4 Hz, 2H), 7.61–7.60 (d, *J* = 7.3 Hz, 1H), 7.56–7.53 (m, 1H), 7.43–7.29 (m, 4H), 4.77–4.69 (m, 2H), 4.63–4.45 (m, 2H), 4.24–4.22 (m, 1H), 3.96–3.94 (m, 1H), 3.86–3.80 (m, 1H), 3.70–3.60 (m, 4H), 3.48–3.44 (m, 2.5H), 3.22–3.11 (m, 4H), 2.99–2.94 (dd, *J* = 7.3, 15.1 Hz, 0.5H) ppm. HRMS (ESI^+^) calcd for C_23_H_27_NO_7_Na [M + Na]^+^: 452.1685; found: 452.1678.

##### 4-(9*H*-Fluoren-9-yl)methyl 3-Methyl (3*S*)-3,4-Dihydro-2*H*-oxazine-3,4-dicarboxylate (**68**)

Alcohol **67** (459 mg, 1.07 mmol) was dissolved in PhMe (15 mL) and *p*TsOH·H_2_O (20.3 mg, 0.11 mmol, 0.10 equiv) was added. The reaction flask was equipped with a dropping funnel including MS (4 Å, 5.2 g) and a condenser. The mixture was stirred at 123 °C for 3 h, before it was filtered through a short plug of NaHCO_3_. The filtrate was concentrated under reduced pressure and the residue was purified by column chromatography (PE/EtOAc = 4:1) to furnish the product (265 mg, 0.73 mmol, 68%) as colorless foam. The analytical data are consistent with those reported in the literature (F. Sladojevich, A. Trabocchi, A. Guarna, *J. Org. Chem.***2007**, 72, 4254–4257). ^1^H NMR (400 MHz, CDCl_3_) = δ (3:2 mixture of rotamers) 7.79–7.75 (t, *J* = 7.6 Hz, 2H), 7.63–7.59 (m, 1H), 7.52–7.49 (dd, *J* = 2.7, 7.2 Hz, 1H), 7.44–7.39 (q, *J* = 6.9 Hz, 2H), 7.35–7.29 (m, 2H), 6.42–6.41 (dd, *J* = 1.1, 5.0 Hz, 0.4H), 6.42–6.41 (dd, *J* = 1.1, 5.0 Hz, 0.6H), 6.02–6.01 (d, *J* = 4.9 Hz, 0.4H), 5.98–5.97 (d, *J* = 5.0 Hz, 0.6H), 4.98 (s, 0.6H), 4.70–4.67 (dd, *J* = 1.2, 10.9 Hz, 0.6H), 4.61–4.40 (m, 3H), 3.34–4.30 (t, *J* = 7.2 Hz, 0.6H), 4.23–4.22 (t, *J* = 6.1 Hz, 0.4H), 4.01–3.98 (dd, *J* = 2.8, 11.1 Hz, 0.6H), 3.89–3.86 (dd, *J* = 2.8, 11.1 Hz, 0.4H), 3.79 (s, 1.9H), 3.71 (s, 1.1H) ppm. HRMS (ESI^+^) calcd for C_21_H_19_NO_5_Na [M + Na]^+^: 388.1161; found: 388.1161.

##### 4-(9*H*-Fluoren-9-yl)methyl 3-Methyl (3*S*)-Morpholine-3,4-dicarboxylate (**69**)

Alkene **68** (160 mg, 0.44 mmol) was dissolved in MeOH (3.1 mL) and CH_2_Cl_2_ (1.6 mL). Pt/C (10%, 19.7 mg) was added and the mixture was stirred at rt under an H_2_ atmosphere for 15 h. The mixture was filtered through a short plug of Celite. The filtrate was concentrated under reduced pressure and the residue was purified by column chromatography (dry load, PE/EtOAc = 4:1) to furnish the morpholine (149 mg, 0.41 mmol, 93%) as colorless foam.

The analytical data are consistent with those reported in the literature (F. Sladojevich, A. Trabocchi, A. Guarna, *J. Org. Chem.***2007**, 72, 4254–4257). ^1^H NMR (400 MHz, CDCl_3_) = δ (mixture of rotamers) 7.78–7.75 (t, *J* = 7.0 Hz, 2H), 7.62–7.58 (m, 1H), 7.52–7.48 (t, *J* = 6.9 Hz, 1H), 7.43–7.38 (m, 2H), 7.35–7.28 (m, 2H), 4.66–4.65 (d, *J* = 2.8 Hz, 0.6H), 4.56–4.45 (m, 1.6H), 4.43–4.37 (m, 1.4H), 4.30–4.28 (m, 1.6H), 4.24–4.21 (t, *J* = 7.2 Hz, 0.5H), 3.91–3.84 (m, 1.4H), 3.78 (s, 1.5H), 3.73 (s, 1.5H), 3.69–3.65 (dd, *J* = 3.7, 11.7 Hz, 0.7H), 3.61–3.57 (dd, *J* = 3.9, 11.9 Hz, 0.5H), 3.51–3.41 (m, 1.8H), 3.32–3.25 (ddt, *J* = 3.7, 12.8 Hz, 0.5H) ppm. HRMS (ESI^+^) calcd for C_21_H_21_NO_5_Na [M + Na]^+^: 390.1317; found: 390.1317.

##### (3*S*)-4-{[(9*H*-Fluoren-9-yl)methoxy]carbonyl}morpholine-3-carboxylic Acid (**70**)

Ester **69** (147 mg, 0.40 mmol) was dissolved in 1,4-dioxane (1 mL) and a 5 M HCl solution (1 mL) was added. The mixture was stirred at 110 °C for 16 h. A 5% Na_2_CO_3_ solution was added at rt and the aqueous phase was washed with Et_2_O, acidified with conc. HCl and extracted with CH_2_Cl_2_ (3×). The combined organic phases were dried over MgSO_4_, filtered and concentrated under reduced pressure. The acid (127 mg, 0.36 mmol, 90%) was obtained as colorless amorphous solid, which was used in the next step without further purification. The peaks in the ^1^H NMR spectrum are shifted compared to the literature (F. Sladojevich, A. Trabocchi, A. Guarna, *J. Org. Chem.***2007**, 72, 4254–4257), what might be caused by 1,4-dioxane impurities. [α]_D_^25^ = −35.2° (c 1.0_2_Cl_2_). ^1^H NMR (400 MHz, CDCl_3_) = δ (mixture of rotamers) 7.78–7.76 (d, *J* = 7.6 Hz, 1H), 7.75–7.71 (t, *J* = 6.8 Hz, 1H), 7.60–7.57 (m, 1H), 7.53–7.48 (m, 1H), 7.42–7.36 (m, 2H), 7.34–7.28 (m, 2H), 4.71–4.70 (d, *J* = 3.0 Hz, 0.5H), 4.59–4.50 (m, 1.5H), 4.47–4.40 (m, 1H), 4.32–4.21 (m, 2H), 3.93–3.90 (m, 1H), 3.81–3.73 (m, 1.5H), 3.68–3.63 (m, 1H), 3.60–3.56 (dd, *J* = 4.0, 11.9 Hz, 0.5H), 3.52–3.40 (m, 1.5H), 3.31–3.23 (ddt, *J* = 3.4, 13.4 Hz, 0.5H) ppm. HRMS (ESI^+^) calcd for C_20_H_18_NO_5_ [M – H]^−^: 352.1185; found: 352.1185.

##### (9*H*-Fluoren-9-yl)methyl (3*S*)-3-[(4-{[4-({4-[(*tert*-Butoxy)carbonyl]phenyl}carbamoyl)-3-(prop-2-en-1-yloxy)-2-(propan-2-yloxy)phenyl]carbamoyl}phenyl)carbamoyl]morpholine-4-carboxylate (**71**)

Step 1: Carboxylic acid **70** (90.0 mg, 0.25 mmol) was stirred in SOCl_2_ (1 mL) at 80 °C for 30 min. The solvent was removed under reduced pressure to furnish the acyl chloride (101 mg, quant.) as yellow oil, which was used in the next step without further purification. Step 2: Amine **8** (139.0 mg, 0.25 mmol) and 2,6-lutidine (136 μL, 1.17 mmol, 4.6 equiv) were dissolved in CH_2_Cl_2_ (2.5 mL). The acid chloride (94.7 mg, 0.25 mmol, 1.00 equiv) in CH_2_Cl_2_ (2.5 mL) was added at 0 °C and the mixture was stirred at rt for 18 h. Afterward the mixture was washed with a 1 M HCl solution, a 5% Na_2_CO_3_ solution and brine, dried over MgSO_4_, filtered and concentrated under reduced pressure. The residue was purified by column chromatography (PE/EtOAc = 1:1) to furnish the product (142 mg, 0.16 mmol, 63%) as yellow amorphous solid. [α]_D_^25^ = −72.0° (c 0.3_2_Cl_2_). ^1^H NMR (400 MHz, DMSO-*d*_6_) = (mixture of rotamers) 10.53 (s, 1H), 10.45 (s, 0.5H), 10.39 (s, 0.5H), 9.58 (s, 0.5H), 9.54 (s, 0.5H), 8.05–8.03 (d, *J* = 8.4 Hz, 1H), 7.99–7.98 (d, *J* = 8.4 Hz, 1H), 7.92–7.88 (m, 3H), 7.84–7.80 (m, 5H), 7.76–7.74 (d, *J* = 8.5 Hz, 2H), 7.71–7.68 (t, *J* = 7.7 Hz, 1H), 7.57–7.55 (m, 1H), 7.45–7.27 (m, 4H), 7.09–7.03 (m, 1H), 6.06–5.98 (m, 1H), 5.39–5.36 (d, *J* = 17.2 Hz, 1H), 5.21–5.19 (d, *J* = 10.5 Hz, 1H), 4.61 (s, 2H), 4.56–4.54 (m, 1H), 4.49 (bs, 1H), 4.40–4.20 (m, 4H), 3.92–3.86 (m, 1H), 3.79–3.71 (m, 2H), 3.62–3.58 (m, 1H), 3.50–3.38 (m, 2H), 1.55 (s, 9H), 1.27–1.25 (m, 6H) ppm. ^13^C NMR (101 MHz, DMSO-*d*_6_) = (mixture of rotamers) 169.0, 164.6, 164.6, 164.3, 156.0, 155.5, 149.5, 143.7, 143.0, 140.7, 135.6, 133.7, 130.1, 128.5, 127.7, 127.6, 127.2, 127.0, 126.0, 125.1 (t, *J* = 12.4 Hz), 123.6, 120.1, 118.8, 118.7, 117.8, 80.3, 76.3, 74.3, 67.8–67.1, 65.9, 65.5, 59.7, 55.2, 54.6, 46.6, 46.5, 27.9, 22.3 ppm. HRMS (ESI) calcd for C_51_H_52_N_4_O_10_ Na[M + Na]^+^: 903.3581; found: 903.3572.

##### *tert*-Butyl 4-(4-{4-[(3*S*)-4-[4-(4-Cyanobenzamido)benzoyl]morpholine-3-amido]benzamido}-2-(prop-2-en-1-yloxy)-3-(propan-2-yloxy)benzamido)benzoate (**72**)

Step 1: Carbamate **71** (132 mg, 0.15 mmol) was dissolved in MeCN/piperidine (4:1, 1.8 mL). The solution was stirred at rt for 90 min and then concentrated under reduced pressure. The residue was coevaporated with MeCN (3×) to furnish the crude product, which was used in the next step without further purification. Step 2: DIPEA (88 μL, 0.50 mmol, 5.00 equiv) was added dropwise to a stirred solution of HATU (95.7 mg, 0.25 mmol, 2.50 equiv) and carboxylic acid **10** (67.0 mg, 0.25 mmol, 2.50 equiv) in DMF (2.5 mL). The solution was stirred for 5 min and was then transferred to a stirred solution of the amine (66.3 mg, 0.10 mmol) in DMF (1.4 mL). The reaction mixture was stirred at rt for 16 h. The mixture was diluted with EtOAc and washed with a 1 M HCl solution, brine, dried over MgSO_4_, filtered and concentrated under reduced pressure. The crude product was purified by column chromatography (dry load, MeOH in CH_2_Cl_2_ = 0.5, 1, 3%) to furnish the product (63.8 mg, 0.07 mmol, 70%) as yellow amorphous solid. [α]_D_^27^ = −8.0° (c 0.1, MeOH). ^1^H NMR (500 MHz, DMSO-*d*_6_) = δ 10.67 (s, 1H), 10.53 (s, 1H), 10.47 (s, 1H), 9.55 (s, 1H), 8.12–8.11 (m, 2H), 8.05–8.04 (d, *J* = 8.2 Hz, 2H), 8.01–8.00 (m, 2H), 7.90–7.88 (m, 4H), 7.84–7.69 (m, 6H), 7.50 (bs, 1H), 7.42–7.40 (d, *J* = 8.5 Hz, 1H), 6.06–5.98 (m, 1H), 5.40–5.35 (dq, *J* = 1.7, 17.2 Hz, 1H), 5.22–5.19 (dq, *J* = 1.6, 10.5 Hz, 1H), 5.03 (bs, 0.5H), 4.62–4.61 (d, *J* = 5.4 Hz, 2H), 4.53–4.46 (sept, *J* = 6.1 Hz, 1H), 4.43–4.40 (m, 1H), 4.35–4.18 (m, 0.5H), 3.96–3.80 (m, 3H), 3.54–3.42 (m, 2H), 1.55 (s, 9H), 1.27–1.26 (d, *J* = 6.0 Hz, 6H) ppm. ^13^C NMR (126 MHz, DMSO-*d*_6_) = δ 170.7, 168.7, 164.6, 164.5, 164.4, 164.3, 149.5, 143.0, 142.6, 142.1, 140.2, 138.7, 135.6, 133.7, 132.5, 130.1, 128.6, 128.5, 128.1, 127.2, 126.0, 123.6, 119.9, 119.0, 118.8, 118.3, 117.8, 114.0, 80.3, 76.2, 74.3, 67.9, 65.6, 53.3, 45.5, 27.9, 22.3 ppm. HRMS (ESI) calcd for C_51_H_50_N_6_O_10_Na [M + Na]^+^: 929.3486; found: 929.3494.

##### *tert*-Butyl 4-(4-{4-[(3*S*)-4-[4-(4-Cyanobenzamido)benzoyl]morpholine-3-amido]benzamido}-2-hydroxy-3-(propan-2-yloxy)benzamido)benzoate (**73**)

Allyl ether **72** (60.9 mg, 0.07 mmol) was dissolved in THF (3 mL). Aniline (20 μL, 0.22 mmol, 3.30 equiv) and Pd(PPh_3_)_4_ (7.8 mg, 7 μmol, 0.10 equiv) were added subsequently and the resulting mixture was stirred at rt for 90 min. The mixture was concentrated under reduced pressure. The crude product was purified by column chromatography (dry load, MeOH in CH_2_Cl_2_ = 2, 3%) to furnish the product (53.0 mg, 0.06 mmol, 91%) as yellow amorphous solid. [α]_D_^27^ = −10.6° (c 0.2, THF). ^1^H NMR (600 MHz, DMSO-*d*_6_) = 12.28 (s, 1H), 10.67–10.20 (m, 3H), 9.43 (s, 1H), 8.11 (bs, 2H), 8.05–8.04 (d, *J* = 7.9 Hz, 2H), 7.98 (bs, 2H), 7.94–7.92 (d, *J* = 8.8 Hz, 2H), 7.90–7.73 (m, 7H), 7.70–7.68 (d, *J* = 8.8 Hz, 1H), 7.50–7.38 (m, 2H), 5.03 (bs, 0.5H), 4.58–4.52 (sept, *J* = 6.1 Hz, 1H), 4.43–4.18 (m, 1.5H), 3.98–3.80 (m, 3H), 3.54–3.50 (m, 2H), 1.55 (s, 9H), 1.27–1.26 (d, *J* = 5.7 Hz, 6H) ppm. ^13^C NMR (126 MHz, DMSO-*d*_6_) = 170.7, 168.7, 168.4, 164.5, 164.4, 164.2, 154.2, 142.2, 142.0, 140.2, 138.7, 137.0, 136.4, 132.5, 131.5, 131.4, 130.5, 129.9, 128.6, 128.4, 128.1, 126.8, 122.8, 120.7, 119.9, 118.9, 118.3, 114.0, 112.5, 112.2, 80.5, 74.8, 67.9, 66.0, 53.3, 45.5, 27.8, 22.3 ppm. HRMS (ESI) calcd for C_48_H_46_N_6_O_10_Na [M + Na]^+^: 889.3173; found: 889.3176.

##### 4-(4-{4-[(3*S*)-4-[4-(4-Cyanobenzamido)benzoyl]morpholine-3-amido]benzamido}-2-hydroxy-3-(propan-2-yloxy)benzamido)benzoic Acid (**20**)

*tert*-Butyl ester **73** (48.0 mg, 0.06 mmol) was dissolved in precooled TFA (3 mL) at 0 °C with stirring. The solution was warmed up to rt over 30 min. Et_2_O was added at 0 °C. The precipitate was filtered off, washed with an excess of Et_2_O and dried *in vacuo* to furnish the product (35.4 mg, 0.04 mmol, 79%) as colorless amorphous solid. [α]_D_^27^ = −7.9° (c 0.1, MeOH). ^1^H NMR (600 MHz, DMSO-*d*_6_) = 12.82 (bs, 1H), 12.28 (s, 1H), 10.67 (s, 1H), 10.60 (s, 1H), 10.49–10.20 (m, 1H), 9.44 (s, 1H), 8.11 (bs, 2H), 8.05–8.04 (d, *J* = 7.9 Hz, 2H), 7.98–7.96 (m, 5H), 7.89–7.73 (m, 8H), 7.71–7.69 (d, *J* = 8.8 Hz, 1H), 7.50–7.38 (m, 2H), 5.04 (bs, 0.7H), 4.58–4.52 (sept, *J* = 6.1 Hz, 1H), 4.41–4.17 (m, 2H), 3.96–3.80 (m, 4.3H), 1.28–1.27 (d, *J* = 6.1 Hz, 6H) ppm. ^13^C NMR (126 MHz, DMSO-*d*_6_) = 170.7, 168.7, 168.5, 166.9, 164.4, 164.2, 154.1, 142.2, 142.0, 140.2, 138.7, 137.0, 136.4, 132.5, 130.2, 128.6, 128.4, 128.1, 126.3, 122.8, 120.7, 119.9, 118.9, 118.3, 114.0, 112.5, 112.3, 74.9, 67.9, 66.0, 53.3, 45.5, 22.3 ppm. HRMS (ESI) calcd for C_44_H_37_N_6_O_10_ [M-H]^−^: 809.2571; found: 809.2578.

##### *tert*-Butyl 4-(4-{4-[(2*S*)-2-Aminopent-4-enamido]benzamido}-2-(prop-2-en-1-yloxy)-3-(propan-2-yloxy)benzamido)benzoate (**74**)

Amine **8** (0.09 mmol) was coupled with (2*S*)-2-({[(9*H*-Fluoren-9-yl)methoxy]carbonyl}amino)pent-4-enoic acid using general procedure 9 and deprotected using general procedure 2. Yellow to orange solid, 32 mg (51% over 2 steps). ^1^H NMR (500 MHz, CDCl_3_, 300 K): δ (ppm) = 10.17 (s, 1H), 9.85 (s, 1H), 8.75 (s, 1H), 8.49 (d, 1H, *J* = 8.9 Hz), 8.05 (d, 1H, *J* = 8.9 Hz), 7.98 (d, 2H, *J* = 8.8 Hz), 7.89 (d, 2H, *J* = 8.8 Hz), 7.79 (d, 2H, *J* = 8.8 Hz), 7.73 (d, 2H, *J* = 8.8 Hz), 6.14 (ddt, 1H, *J* = 5.9 Hz, 10.4 Hz, 16.3 Hz), 5.84–5.74 (m, 1H), 5.49 (dq, 1H, *J* = 1.4 Hz, 17.1 Hz), 5.40 (ddd, 1H, *J* = 1.0 Hz, 2.2 Hz, 10.4 Hz), 5.22–5.18 (m, 2H), 4.75 (hept., 1H, *J* = 6.1 Hz), 4.69 (d, 2H, *J* = 5.9 Hz), 3.59 (dd, 1H, *J* = 3.5 Hz, 8.3 Hz), 2.75–2.67 (m, 1H), 2.45–2.38 (m, 1H), 1.59 (s, 9H), 1.38 (dd, 6H, *J* = 1.7 Hz, 6.1 Hz). ^13^C NMR (126 MHz, CDCl_3_, 300 K): δ (ppm) = 173.1, 165.5, 164.4, 162.8, 149.4, 142.3, 141.5, 139.1, 137.8, 134.0, 132.3, 130.8, 129.7, 128.3, 127.6, 127.4, 121.6, 120.2, 119.6, 119.6, 119.3, 115.8, 80.9, 76.9, 75.1, 54.5, 39.3, 28.4, 23.0. HRMS (ESI) calcd 643.3132 [M + H^+^], 643.3126 found. HPLC purity 97.3%.

##### 4-(4-{4-[(2*S*)-2-{[4-(4-Cyanobenzamido)phenyl]formamido}pent-4-enamido]benzamido}-2-hydroxy-3-(propan-2-yloxy)benzamido)benzoic Acid (**21**)

The amine **74** (49.8 μmol) was coupled with carboxylic acid **10** using general procedure 4. The product was obtained by deprotection with general procedures 6 and 7. Beige solid, 13 mg (35% over 3 steps). ^1^H NMR (700 MHz, DMSO-*d*_6_, 300 K): δ (ppm) = 12.82 (br s, 1H), 12.30 (s, 1H), 10.70 (s, 1H), 10.60 (s, 1H), 10.50 (s, 1H), 9.40 (s, 1H), 8.62 (d, 1H, *J* = 7.5 Hz), 8.13 (d, 2H, J = 8.5 Hz), 8.05 (d, 2H, *J* = 8.5 Hz), 7.98–7.95 (m, 6H), 7.89 (d, 2H, *J* = 8.8 Hz), 7.87–7.85 (m, 3H), 7.82 (d, 2H, *J* = 8.7 Hz), 7.71 (d, 1H, *J* = 8.8 Hz), 5.89 (ddt, 1H, J = 6.9 Hz, 10.2 Hz, 17.1 Hz), 5.20 (dd, 1H, *J* = 1.8 Hz, 17.2 Hz), 5.09 (dd, 1H, *J* = 1.9 Hz, 10.2 Hz), 4.70 (quart., 1H, *J* = 7.5 Hz), 4.55 (hept., 1H, *J* = 6.1 Hz), 2.62 (t, 2H, *J* = 7.1 Hz), 1.27 (d, 6H, *J* = 6.1 Hz). ^13^C NMR (176 MHz, DMSO-*d*_6_, 300 K): δ (ppm) = 171.0, 168.5, 166.9, 166.0, 164.4, 164.2, 154.1, 142.3, 142.0, 141.6, 138.7, 137.0, 136.3, 134.3, 132.5, 130.2, 129.1, 128.6, 128.4, 128.4, 126.3, 122.8, 120.7, 119.5, 118.8, 118.3, 117.8, 114.0, 112.4, 112.1, 74.8, 54.1, 35.7, 22.3. HRMS (ESI) calcd 795.2779 [M + H^+^], 795.2774 found. HPLC purity 99.0%.

##### *tert*-Butyl 4-(4-{4-[(2*S*)-2-Aminopentanamido]benzamido}-2-(prop-2-en-1-yloxy)-3-(propan-2-yloxy)benzamido)benzoate (**76**)

Amine **8** (0.09 mmol) was coupled with (2*S*)-2-({[(9*H*-fluoren-9-yl)methoxy]carbonyl}amino)pentanoic acid using general procedure 8 and deprotected using general procedure 2. The product was a yellowish solid. Twenty-seven mg (42% over 2 steps). ^1^H NMR (500 MHz, CDCl_3_, 300 K): δ (ppm) = 10.29 (br s, 1H), 10.15 (s, 1H), 8.72 (s, 1H), 8.37 (d, 1H, *J* = 8.1 Hz), 7.97–7.92 (m, 3H), 7.82–7.73 (m, 4H), 7.69 (d, 2H, *J* = 8.6 Hz), 6.10 (dq, 1H, *J* = 5.8 Hz, 10.9 Hz), 5.46 (d, 1H, *J* = 17.1 Hz), 5.38 (d, 1H, *J* = 10.4 Hz), 4.74–4.61 (m, 3H), 4.20–4.02 (m, 1H), 2.02–1.90 (m, 1H), 1.86–1.74 (m, 1H), 1.58 (s, 9H), 1.52–1.40 (m, 2H), 1.33 (d, 6H, *J* = 5.6 Hz), 0.93–0.86 (m, 3H). ^13^C NMR (126 MHz, CDCl_3_, 300 K): δ (ppm) = 170.6, 165.3, 164.5, 162.7, 149.3, 142.0, 141.4, 139.1, 137.4, 132.1, 130.6, 129.6, 128.0, 127.4, 127.2, 121.6, 120.0, 119.7, 119.1, 115.7, 80.8, 76.7, 74.9, 54.8, 35.1, 28.2, 22.7, 18.5, 13.7. HRMS (ESI) calcd 645.3288 [M + H^+^], 645.3284 found.

##### 4-(4-{4-[(2*S*)-2-{[4-(4-Cyanobenzamido)phenyl]formamido}pentanamido]benzamido}-2-hydroxy-3-(propan-2-yloxy)benzamido)benzoic Acid (**22**)

The amine **76** (40.3 μmol) was coupled with carboxylic acid **10** using general procedure 4. The product was obtained by deprotection with general procedures 6 and 7. Beige solid, 14 mg (47% over 3 steps). ^1^H NMR (700 MHz, DMSO-*d*_6_, 300 K): δ (ppm) = 12.82 (br s, 1H), 12.30 (s, 1H), 10.69 (s, 1H), 10.60 (s, 1H), 10.47 (s, 1H), 9.40 (s, 1H), 8.57 (d, 1H, *J* = 7.4 Hz), 8.13 (d, 2H, *J* = 8.5 Hz), 8.05 (d, 2H, *J* = 8.4 Hz), 7.98–7.95 (m, 6H), 7.89 (d, 2H, *J* = 8.8 Hz), 7.87–7.85 (m, 3H), 7.82 (d, 2H, *J* = 8.8 Hz), 7.71 (d, 1H, *J* = 8.9 Hz), 4.61 (dd, 1H, *J* = 7.4 Hz, 14.7 Hz), 4.55 (hept., 1H, *J* = 6.1 Hz), 1.87–1.78 (m, 2H), 1.55–1.48 (m, 1H), 1.43–1.36 (m, 1H), 1.27 (dd, 6H, *J* = 0.9 Hz, 6.1 Hz), 0.95 (t, 3H, *J* = 7.4 Hz). ^13^C NMR (176 MHz, DMSO-*d*_6_, 300 K): δ (ppm) = 171.9, 168.5, 166.9, 166.1, 164.4, 164.2, 154.1, 142.5, 142.0, 141.5, 138.7, 137.1, 136.3, 132.5, 130.2, 129.2, 128.6, 128.4, 128.4, 128.3, 126.3, 122.8, 120.7, 119.5, 118.8, 118.3, 114.0, 112.4, 112.2, 74.9, 54.4, 33.5, 22.3, 19.2, 13.7. HRMS (ESI) calcd 797.2935 [M + H^+^], 797.2929 found. HPLC purity 95.8%.

##### *tert*-Butyl 4-(4-{4-[(2*S*)-2-Aminohexanamido]benzamido}-2-(prop-2-en-1-yloxy)-3-(propan-2-yloxy)benzamido)benzoate (**78**)

Amine **8** (0.09 mmol) was coupled with (2*S*)-2-({[(9*H*-Fluoren-9-yl)methoxy]carbonyl}amino)hexanoic acid using general procedure 9 and deprotected using general procedure 2. Slightly yellow solid, 19 mg (30% over 2 steps). ^1^H NMR (500 MHz, CDCl_3_, 300 K): δ (ppm) = 10.17 (s, 1H), 9.98 (s, 1H), 8.74 (s, 1H), 8.46 (d, 1H, *J* = 8.9 Hz), 8.03 (d, 1H, *J* = 8.9 Hz), 7.97 (d, 2H, *J* = 8.7 Hz), 7.86 (d, 2H, *J* = 8.4 Hz), 7.78 (d, 2H, *J* = 8.4 Hz), 7.72 (d, 2H, *J* = 8.8 Hz), 6.13 (ddt, 1H, *J* = 5.9 Hz, 10.4 Hz, 16.3 Hz), 5.49 (dd, 1H, *J* = 1.4 Hz, 17.2 Hz), 5.40 (dd, 1H, *J* = 1.1 Hz, 10.4 Hz), 4.74 (hept., 1H, *J* = 6.1 Hz), 4.69 (d, 2H, *J* = 5.8 Hz), 3.75–3.63 (m, 1H), 2.03–1.94 (m, 1H), 1.73–1.63 (m, 1H), 1.59 (s, 9H), 1.45–1.39 (m, 2H), 1.39–1.33 (m, 8H), 0.91 (t, 3H, *J* = 7.0 Hz). ^13^C NMR (126 MHz, CDCl_3_, 300 K): δ (ppm) = 173.0, 165.6, 165.5, 162.8, 149.5, 142.3, 141.6, 139.1, 137.7, 132.3, 130.8, 129.7, 128.3, 127.5, 127.5, 121.7, 120.2, 119.5, 119.2, 115.8, 80.9, 76.9, 75.1, 55.6, 34.1, 28.4, 27.9, 22.9, 22.6, 14.0. HRMS (ESI) calcd 659.3445 [M + H^+^], 659.3439 found.

##### 4-(4-{4-[(2*S*)-2-{[4-(4-Cyanobenzamido)phenyl]formamido}hexanamido]benzamido}-2-hydroxy-3-(propan-2-yloxy)benzamido)benzoic Acid (**23**)

The amine **78** (33.2 μmol) was coupled with carboxylic acid **10** using general procedure 4. The product was obtained by deprotection with general procedures 6 and 7. Beige solid, 11 mg (50% over 3 steps). ^1^H NMR (700 MHz, DMSO-*d*_6_, 300 K): δ (ppm) = 12.82 (br s, 1H), 12.30 (s, 1H), 10.69 (s, 1H), 10.60 (s, 1H), 10.47 (s, 1H), 9.40 (s, 1H), 8.57 (d, 1H, *J* = 7.4 Hz), 8.13 (d, 2H, *J* = 8.5 Hz), 8.05 (d, 2H, *J* = 8.5 Hz), 7.98–7.95 (m, 6H), 7.89 (d, 2H, *J* = 8.8 Hz), 7.87–7.84 (m, 3H), 7.82 (d, 2H, *J* = 8.8 Hz), 7.71 (d, 1H, *J* = 8.8 Hz), 4.59 (dd, 1H, *J* = 7.3 Hz, 14.7 Hz), 4.55 (hept., 1H, *J* = 6.1 Hz), 1.84 (dd, 2H, *J* = 7.3 Hz, 14.5 Hz), 1.50–1.44 (m, 1H), 1.38–1.33 (m, 3H), 1.27 (d, 6H, *J* = 6.1 Hz), 0.90 (t, 3H, *J* = 7.1 Hz). ^13^C NMR (176 MHz, DMSO-*d*_6_, 300 K): δ (ppm) = 171.9, 168.5, 166.9, 166.1, 164.4, 164.2, 154.1, 142.5, 142.0, 141.5, 138.7, 137.1, 136.3, 132.5, 130.2, 129.2, 128.6, 128.4, 128.4, 128.3, 126.3, 122.8, 120.7, 119.5, 118.8, 118.3, 114.0, 112.4, 112.2, 74.9, 54.7, 31.2, 28.1, 22.3, 21.9, 13.9. HRMS (ESI) calcd 811.3092 [M + H^+^], 811.3086 found. HPLC purity 97.3%.

##### *tert*-Butyl 4-(4-{4-[(2*S*)-3-Cyclopropyl-2-({[(9*H*-fluoren-9-yl)methoxy]carbonyl}amino)propanamido]benzamido}-2-(prop-2-en-1-yloxy)-3-(propan-2-yloxy)benzamido)benzoate (**80**)

Amine **8** (400 mg, 0.73 mmol), (2*S*)-3-cyclopropyl-2-({[(9*H*-fluoren-9-yl)methoxy]carbonyl}amino)propanoic acid (438 mg, 1.25 mmol, 1.70 equiv) and EEDQ (290 mg, 1.17 mmol, 1.60 equiv) were dissolved in precooled CHCl_3_ (3.7 mL) at 0 °C. The mixture was stirred for 19 h while warming to rt. The solution was concentrated on silica and purified by column chromatography (20% Et_2_O in CH_2_Cl_2_, then 1% MeOH in CH_2_Cl_2_) to furnish the carbamate (547 g, 0.62 mmol, 85%) was yellow amorphous solid, which contained minor impurities. The compound was used in the next step without further purification. HRMS (ESI) calcd for C_52_H_54_N_4_O_9_Na [M + Na]^+^: 901.3788; found: 901.3812.

##### *tert*-Butyl 4-(4-{4-[(2*S*)-2-{[4-(4-Cyanobenzamido)phenyl]formamido}-3-cyclopropylpropanamido]benzamido}-2-(prop-2-en-1-yloxy)-3-(propan-2-yloxy)benzamido)benzoate (**81**)

Step 1: Carbamate **80** (500 mg, 0.57 mmol) was dissolved in MeCN (5.3 mL). Et_2_NH (1.3 mL) was added. The solution was stirred at rt for 30 min. The mixture was concentrated under reduced pressure. The residue was diluted with MeCN and concentrated three times. The crude product was dried *in vacuo* to furnish the amine, which was used in the next step without further purification. Step 2: DIPEA (435 μL, 2.49 mmol, 5.00 equiv) was added dropwise to a stirred solution of HATU (473 mg, 1.24 mmol, 2.50 equiv) and carboxylic acid **10** (331 mg, 1.24 mmol, 2.50 equiv) in DMF (12 mL). The solution was stirred for 30 min and was then transferred to a stirred solution of the amine (327 mg, 0.50 mmol) in DMF (7 mL). The reaction mixture was stirred at rt for 19 h. The mixture was diluted with EtOAc and washed with a 0.1 M HCl solution, brine, dried over MgSO_4_, filtered and concentrated under reduced pressure. The crude product was purified by column chromatography (dry load, MeOH in CH_2_Cl_2_ = 1, 5%) to furnish the product (405.2 mg, 0.45 mmol, 90%) as yellow amorphous solid. [α]_D_^22^ = +13.6° (c 0.3, MeOH). ^1^H NMR (500 MHz, DMSO-*d*_6_) = δ 10.69 (s, 1H), 10.53 (s, 1H), 10.46 (s, 1H), 9.51 (s, 1H), 8.62–8.61 (d, *J* = 7.1 Hz, 1H), 8.14–8.12 (d, *J* = 8.6 Hz, 2H), 8.06–8.04 (d, *J* = 8.6 Hz, 2H), 7.99–7.97 (m, 4H), 7.90–7.88 (d, *J* = 7.8 Hz, 4H), 7.84–7.80 (m, 5H), 7.42–7.40 (d, *J* = 8.5 Hz, 1H), 6.05–5.99 (m, 1H), 5.39–5.36 (dq, *J* = 1.7, 17.2 Hz, 1H), 5.21–5.19 (dq, *J* = 1.7, 10.5 Hz, 1H), 4.71–4.67 (q, *J* = 7.5 Hz, 1H), 4.61–4.60 (d, *J* = 5.4 Hz, 2H), 4.53–4.47 (sept, *J* = 6.1 Hz, 1H), 1.96–1.92 (m, 1H), 1.60–1.57 (m, 1H), 1.55 (s, 9H), 1.27–1.26 (d, J = 6.1 Hz, 6H), 0.91–0.88 (m, 1H), 0.49–0.38 (m, 2H), 0.25–0.14 (m, 2H) ppm. ^13^C NMR (500 MHz, DMSO-d_6_) = δ 171.7, 166.0, 164.6, 164.5, 164.4, 164.3, 149.5, 143.0, 142.5, 142.4, 141.5, 138.7, 135.7, 133.6, 132.5, 130.1, 129.3, 128.6, 128.5, 128.4, 128.3, 127.1, 126.0, 123.6, 119.5, 118.9, 118.8, 118.7, 118.3, 117.8, 114.0, 80.3, 76.2, 74.3, 55.3, 36.3, 27.9, 22.3, 8.2, 4.7, 4.0 ppm. HRMS (ESI) calcd for C_52_H_52_N_6_O_9_Na [M + Na]^+^: 927.3693; found: 927.3712.

##### *tert*-Butyl 4-(4-{4-[(2*S*)-2-{[4-(4-Cyanobenzamido)phenyl]formamido}-3-cyclopropylpropanamido]benzamido}-2-hydroxy-3-(propan-2-yloxy)benzamido)benzoate (**82**)

Allyl ether **81** (374 mg, 0.41 mmol) was dissolved in THF (18 mL). Aniline (124 μL, 1.36 mmol, 3.30 equiv) and Pd(PPh_3_)_4_ (47.7 mg, 0.04 mmol, 0.10 equiv) were added subsequently and the resulting mixture was stirred at rt for 2 h. The mixture was concentrated under reduced pressure. The crude product was purified by column chromatography (dry load, MeOH in CH_2_Cl_2_ = 2, 5%) to furnish the product (286 mg, 0.33 mmol, 80%) as yellow amorphous solid. [α]_D_^22^ = −10.2° (c 0.3, MeOH). ^1^H NMR (500 MHz, DMSO-*d*_6_) = δ 12.29 (s, 1H), 10.69 (s, 1H), 10.62 (bs, 1H), 10.48 (s, 1H), 9.39 (s, 1H), 8.62–8.61 (d, *J* = 7.4 Hz, 1H), 8.14–8.12 (d, *J* = 8.6 Hz, 2H), 8.05–8.04 (d, *J* = 8.6 Hz, 2H), 7.98–7.95 (m, 4H), 7.93–7.92 (d, *J* = 8.9 Hz, 2H), 7.90–7.88 (d, *J* = 8.9 Hz, 2H), 7.86–7.84 (m, 3H), 7.82–7.81 (d, *J* = 8.9 Hz, 2H), 7.71–7.70 (d, *J* = 8.9 Hz, 1H), 4.71–4.68 (q, *J* = 7.5 Hz, 1H), 4.57–4.53 (sept, *J* = 6.1 Hz, 1H), 1.96–1.91 (m, 1H), 1.60–1.58 (m, 1H), 1.55 (s, 9H), 1.27–1.26 (dd, *J* = 1.7, 6.1 Hz, 6H), 0.91–0.87 (m, 1H), 0.49–0.38 (m, 2H), 0.25–0.13 (m, 2H) ppm. ^13^C NMR (151 MHz, DMSO-*d*_6_) = δ 171.7, 168.5, 166.0, 164.5, 164.4, 164.2, 154.1, 142.5, 142.0, 141.5, 138.7, 137.0, 136.3, 132.5, 129.9, 129.3, 128.6, 128.4, 128.4, 128.3, 126.8, 122.8, 120.7, 119.5, 118.8, 118.3, 114.0, 112.4, 112.1, 80.5, 74.8, 55.3, 36.3, 27.8, 22.3, 8.1, 4.6, 4.0 ppm. HRMS (ESI) calcd for C_49_H_48_N_6_O_9_Na [M + Na]^+^: 887.3380; found: 887.3381.

##### 4-(4-{4-[(2*S*)-2-{[4-(4-Cyanobenzamido)phenyl]formamido}-3-cyclopropylpropanamido]benzamido}-2-hydroxy-3-(propan-2-yloxy)benzamido)benzoic Acid (**24**)

*tert*-Butyl ester **82** (250 mg, 0.29 mmol) was dissolved in precooled TFA (10 mL) at 0 °C with stirring. The solution was warmed up to rt over 30 min. Et_2_O was added at 0 °C. The precipitate was filtered off, washed with an excess of Et_2_O and dried *in vacuo* to furnish the acid (205 mg, 0.25 mmol, 88%) as colorless amorphous solid. [α]_D_^22^ = +3.8° (c 0.1, MeOH). ^1^H NMR (600 MHz, DMSO-*d*_6_) = δ 12.82 (bs, 1H), 12.29 (s, 1H), 10.69 (s, 1H), 10.60 (s, 1H), 10.48 (s, 1H), 9.40 (s, 1H), 8.62–8.61 (d, *J* = 7.4 Hz, 1H), 8.14–8.12 (d, *J* = 8.6 Hz, 2H), 8.06–8.04 (d, *J* = 8.6 Hz, 2H), 7.98–7.95 (m, 6H), 7.90–7.88 (d, *J* = 9.1 Hz, 2H), 7.86–7.84 (m, 3H), 7.82–7.81 (d, *J* = 8.8 Hz, 2H), 7.72–7.70 (d, *J* = 8.9 Hz, 1H), 4.71–4.68 (q, *J* = 6.9 Hz, 1H), 4.58–4.52 (sept, *J* = 6.1 Hz, 1H), 1.96–1.91 (m, 1H), 1.60–1.55 (m, 1H), 1.27–1.26 (dd, *J* = 1.5, 6.1 Hz, 6H), 0.90–0.87 (m, 1H), 0.49–0.37 (m, 2H), 0.25–0.13 (m, 2H) ppm. ^13^C NMR (151 MHz, DMSO-*d*_6_) = 171.7, 168.5, 166.9, 166.0, 164.4, 164.2, 154.1, 142.5, 142.0, 141.5, 138.7, 137.0, 136.3, 132.5, 130.2, 129.2, 128.6, 128.4, 128.4, 128.3, 126.3, 122.8, 120.7, 119.5, 118.8, 118.3, 114.0, 112.4, 112.2, 74.9, 55.3, 36.3, 22.3, 8.1, 4.6, 4.0 ppm. HRMS (ESI) calcd for C_45_H_39_N_6_O_9_ [M – H]^−^: 807.2779; found: 807.2767. HPLC purity 98,0%.

##### 4-(4-{4-[(2*S*)-2-{[4-(4-Cyanobenzamido)phenyl]formamido}-3-(2*H*-1,2,3-triazol-4-yl)propanamido]benzamido}-2-hydroxy-3-(propan-2-yloxy)benzamido)benzoic Acid (**25**)

7.4 mg alkyne **13** (9.3 μmol, 1 equiv), 0.15 mg copper(II) sulfate (0.9 μmol, 0.1 equiv), 1.1 mg sodium ascorbate (5.6 μmol, 0.6 equiv) and 2.0 mg TBTA (3.7 μmol, 0.4 equiv) were added to a dry flask and further dried under high vacuum. 0.2 mL DMSO and 0.1 mg THF were added under nitrogen atmosphere. 6.1 mg sodium azide was dissolved in 1.0 mL water and 0.1 mL of the solution was added to the mixture (9.3 μmol, 1 equiv) under nitrogen atmosphere. The reaction was stirred at room temperature and controlled over LCMS. After completion, the crude product was purified by RP HPLC. White solid, 4 mg (49%). ^1^H NMR (700 MHz, DMSO-d_6_, 300 K): δ (ppm) = 15.00 (br s, 0.3 H), 14.64 (s, 0.6 H), 12.82 (br s, 1H), 12.29 (s, 1H), 10.70 (s, 1H), 10.60 (s, 1H), 10.55 (s, 1H), 9.41 (s, 1H), 8.77 (d, 1H, *J* = 7.5 Hz), 8.13 (d, 2H, *J* = 8.4 Hz), 8.05 (d, 2H, *J* = 8.5 Hz), 7.98–7.95 (m, 4H), 7.92 (d, 2H, *J* = 8.8 Hz), 7.88 (d, 2H, *J* = 8.7 Hz), 7.87–7.85 (m, 3H), 7.81 (d, 2H, *J* = 8.6 Hz), 7.71 (d, 1H, *J* = 8.8 Hz), 7.65 (s, 0.6 H), 4.93 (dd, 1H, *J* = 7.7 Hz, 14.3 Hz), 4.55 (hept., 1H, *J* = 6.0 Hz), 3.30–3.21 (m, 2H), 1.27 (d, 6H, *J* = 6.1 Hz). ^13^C NMR (176 MHz, DMSO-*d*_6_, 300 K): δ (ppm) = 170.6, 168.5, 166.9, 166.0, 164.5, 164.2, 154.1, 143.3, 142.3,142.0, 141.6, 138.7, 137.0, 136.3, 132.9, 132.5, 130.2, 129.0, 128.6, 128.5, 128.4, 128.4, 126.3, 122.8, 120.7, 119.5, 119.0, 118.3, 114.0, 112.4, 112.2, 74.9, 54.3, 27.5, 22.3. HRMS (ESI) calcd 836.2792 [M + H^+^], 836.2788 found. HPLC purity 95.0%.

##### *tert*-Butyl 4-(4-{4-[(2*S*)-2-Amino-3-{[(prop-2-en-1-yloxy)carbonyl]amino}propanamido]benzamido}-2-(prop-2-en-1-yloxy)-3-(propan-2-yloxy)benzamido)benzoate (**83**)

Amine **8** (0.09 mmol) was coupled with (2*S*)-2-({[(9*H*-Fluoren-9-yl)methoxy]carbonyl}amino)-3-{[(prop-2-en-1-yloxy)carbonyl]amino}propanoic acid using general procedure 9 and deprotected using general procedure 2. Yellow solid, 14 mg (21% over 2 steps). ^1^H NMR (500 MHz, CDCl_3_, 300 K): δ (ppm) = 10.16 (s, 1H), 9.99 (br s, 1H), 8.74 (s, 1H), 8.46 (d, 1H, *J* = 8.9 Hz), 8.04 (d, 1H, *J* = 8.9 Hz), 7.97 (d, 2H, *J* = 8.7 Hz), 7.88 (d, 2H, *J* = 8.6 Hz), 7.77 (d, 2H, *J* = 8.6 Hz), 7.72 (d, 2H, *J* = 8.8 Hz), 6.13 (ddt, 1H, *J* = 5.9 Hz, 10.4 Hz, 16.3 Hz), 5.89 (ddd, 1H, *J* = 5.7 Hz, 11.0 Hz, 16.1 Hz), 5.49 (dd, 1H, *J* = 1.3 Hz, 17.1 Hz), 5.40 (dd, 1H, *J* = 1.1 Hz, 10.4 Hz), 5.29 (dd, 1H, *J* = 1.5 Hz, 17.2 Hz), 5.21 (dd, 1H, *J* = 1.2 Hz, 10.4 Hz), 4.74 (quart., 1H, *J* = 6.2 Hz), 4.69 (d, 2H, *J* = 5.9 Hz), 4.57 (d, 2H, *J* = 4.7 Hz), 3.78–3.72 (m, 1H), 3.71–3.65 (m, 2H), 1.59 (s, 9H), 1.37 (d, 6H, *J* = 6.2 Hz). ^13^C NMR (126 MHz, CDCl_3_, 300 K): δ (ppm) = 171.4, 165.6, 164.4, 162.8, 157.8, 149.4, 142.3, 141.2, 139.1, 137.7, 132.6, 132.3, 130.8, 130.0, 128.3, 127.6, 127.5, 121.7, 120.2, 119.5, 119.2, 118.2, 115.8, 80.9, 76.9, 75.1, 66.2, 56.4, 44.7, 28.4, 23.0. HRMS (ESI) calcd 716.3296 [M + H^+^], 716.3289 found. HPLC purity 99.9%.

##### 4-(4-{4-[(2*S*)-3-Amino-2-{[4-(4-cyanobenzamido)phenyl]formamido}propanamido]benzamido}-2-hydroxy-3-(propan-2-yloxy)benzamido)benzoic Acid (**26**)

The amine **83** (19.8 μmol) was coupled with carboxylic acid **10** using general procedure 4. After deprotection of the *tert*-butyl ester using general procedure 7, the crude product was purified by RP HPLC. The product was obtained by deprotection with general procedure 5 followed by RP HPLC purification. White solid, 2 mg (15% over 3 steps). ^1^H NMR (700 MHz, DMSO-*d*_6_, 300 K): δ (ppm) = 10.73 (s, 1H), 9.31 (s, 1H), 8.83 (d, 1H, *J* = 6.9 Hz), 8.13 (d, 2H, *J* = 8.4 Hz), 8.05 (d, 2H, *J* = 8.4 Hz), 7.99 (d, 2H, *J* = 8.7 Hz), 7.97–7.94 (m, 4H), 7.92 (d, 2H, *J* = 8.8 Hz), 7.84–7.81 (m, 4H), 7.77 (d, 1H, *J* = 7.5 Hz), 7.59–7.52 (m, 1H), 4.90 (d, 1H, *J* = 5.2 Hz), 4.68–4.59 (m, 1H), 3.21 (dd, 1H, *J* = 8.2 Hz, 13.0 Hz), 1.25 (d, 6H, *J* = 6.1 Hz). ^13^C NMR (176 MHz, DMSO-*d*_6_, 300 K): δ (ppm) = 168.4, 168.2, 167.0, 166.5, 164.5, 164.0, 141.9, 138.6, 136.7, 132.5, 130.3, 129.1, 128.9, 128.6, 128.6, 128.2, 123.0, 120.2, 119.5, 119.4, 118.3, 114.1, 52.7, 40.0, 22.4. HRMS (ESI) calcd 784.2731 [M + H^+^], 784.2727 found. HPLC purity 95.1%.

##### *tert*-Butyl 4-(4-{4-[(2*S*)-2-Amino-4-{[(*tert*-butoxy)carbonyl]amino}butanamido]benzamido}-2-(prop-2-en-1-yloxy)-3-(propan-2-yloxy)benzamido)benzoate (**85**)

Amine **8** (0.09 mmol) was coupled with (2*S*)-4-{[(*tert*-Butoxy)carbonyl]amino}-2-({[(9*H*-fluoren-9-yl)methoxy]carbonyl}amino)butanoic acid using general procedure 8 and deprotected using general procedure 2. Yellow solid, 38 mg (52% over 2 steps). ^1^H NMR (500 MHz, CDCl_3_, 300 K): δ (ppm) = 10.16 (s, 1H), 10.11 (br s, 1H), 8.74 (s, 1H), 8.48 (d, 1H, *J* = 8.9 Hz), 8.05 (d, 1H, *J* = 8.9 Hz), 7.97 (d, 2H, *J* = 8.8 Hz), 7.89 (d, 2H, *J* = 8.8 Hz), 7.80 (d, 2H, *J* = 8.6 Hz), 7.72 (d, 2H, *J* = 8.8 Hz), 6.13 (ddt, 1H, *J* = 5.9 Hz, 10.4 Hz, 16.3 Hz), 5.49 (dd, 1H, *J* = 1.3 Hz, 17.1 Hz), 5.40 (dd, 1H, *J* = 1.0 Hz, 10.4 Hz), 4.89 (t, 1H, *J* = 6.3 Hz), 4.74 (hept., 1H, *J* = 6.2 Hz), 4.69 (d, 2H, *J* = 5.9 Hz), 3.60–3.48 (m, 1H), 3.37–3.29 (d, 2H, *J* = 6.0 Hz), 1.83–1.74 (m, 1H), 1.59 (s, 9H), 1.42 (s, 9H), 1.38 (d, 6H, *J* = 6.1 Hz).^13^C NMR (126 MHz, CDCl_3_, 300 K): δ (ppm) = 173.7, 165.5, 164.4, 162.8, 157.0, 149.4, 142.3, 141.7, 139.1, 137.8, 132.3, 130.8, 129.6, 128.2, 127.6, 127.4, 121.6, 120.1, 119.4, 119.1, 115.8, 80.9, 80.0, 76.9, 75.1, 53.2, 37.1, 36.1, 28.5, 28.4, 22.9. HRMS (ESI) calcd 746.3765 [M + H^+^], 746.3759 found.

##### 4-(4-{4-[(2*S*)-4-Amino-2-{[4-(4-cyanobenzamido)phenyl]formamido}butanamido]benzamido}-2-hydroxy-3-(propan-2-yloxy)benzamido)benzoic Acid (**27**)

The amine **85** (49.6 μmol) was coupled with carboxylic acid **10** using general procedure 4. The product was obtained by deprotection with general procedures 5 and 7. Slightly beige solid, 19 mg (51% over 3 steps). ^1^H NMR (500 MHz, DMSO-*d*_6_, 300 K): δ (ppm) = 10.73 (s, 1H), 10.52 (br s, 1H), 9.22 (s, 1H), 8.81 (d, 1H, J = 6.7 Hz), 8.13 (d, 2H, *J* = 8.5 Hz), 8.04 (d, 2H, *J* = 8.5 Hz), 7.98 (d, 2H, *J* = 8.7 Hz), 7.96–7.89 (m, 6H), 7.84–7.80 (m, 4H), 7.72 (d, 1H, *J* = 8.8 Hz), 7.49 (d, 1H, *J* = 8.5 Hz), 4.76–4.67 (m, 2H), 3.00–2.93 (m, 2H), 2.25–2.19 (m, 1H), 2.17–2.09 (m, 1H), 1.24 (d, 6H, *J* = 6.2 Hz). ^13^C NMR (126 MHz, DMSO-*d*_6_, 300 K): δ (ppm) = 170.4, 168.0, 167.2, 166.3, 164.5, 163.8, 158.0, 143.0, 142.0, 141.7, 138.6, 136.9, 136.0, 132.5, 130.3, 129.0, 128.9, 128.6, 128.5, 128.1, 125.6, 123.2, 119.8, 119.5, 119.1, 118.3, 114.1, 113.7, 73.3, 52.2, 36.4, 29.4, 22.4. HRMS (ESI) calcd 798.2888 [M + H^+^], 798.2884 found. HPLC purity 97.0%.

##### *tert*-Butyl 4-(4-{4-[(2*S*)-2-Amino-5-{[(prop-2-en-1-yloxy)carbonyl]amino}pentanamido]benzamido}-2-(prop-2-en-1-yloxy)-3-(propan-2-yloxy)benzamido)benzoate (**87**)

Aniline **8** (0.09 mmol) was coupled with (2*S*)-2-({[(9*H*-fluoren-9-yl)methoxy]carbonyl}amino)-5-{[(prop-2-en-1-yloxy)carbonyl]amino}pentanoic acid and deprotected using general procedure 11. Yellow solid, 14 mg (19% over 2 steps). ^1^H NMR (500 MHz, CDCl_3_, 300 K): δ (ppm) = 10.14 (br s, 1H), 8.72 (s, 1H), 8.39 (d, 1H, *J* = 8.7 Hz), 7.96 (m, 3H), 7.84–7.74 (m, 4H), 7.70 (d, 2H, *J* = 8.7 Hz), 6.16–6.07 (m, 1H), 5.85 (ddd, 1H, *J* = 5.5 Hz, 10.7 Hz, 22.5 Hz), 5.47 (dd, 1H, *J* = 1.1 Hz, 17.1 Hz), 5.39 (d, 1H, *J* = 10.4 Hz), 5.25 (d, 1H, *J* = 17.2 Hz), 5.15 (d, 1H, *J* = 10.3 Hz), 4.74–4.64 (m, 3H), 4.53 (d, 2H, *J* = 5.3 Hz), 4.25–4.15 (m, 1H), 3.36–3.24 (m, 1H), 3.22–3.09 (m, 1H), 2.12–1.99 (m, 1H), 1.98–1.86 (m, 1H), 1.82–1.65 (m, 2H), 1.59 (s, 9H), 1.35 (d, 6H, *J* = 6.1 Hz). ^13^C NMR (126 MHz, CDCl_3_, 300 K): δ (ppm) = 165.5, 164.5, 162.8, 157.4, 149.5, 142.2, 141.5, 139.3, 137.5, 132.7, 132.3, 130.8, 130.0, 128.2, 127.5, 127.4, 121.8, 120.1, 119.8, 119.2, 117.9, 115.8, 81.0, 76.9, 75.1, 66.0, 54.1, 53.6, 39.9, 28.4, 26.1, 22.9. HRMS (ESI) calcd 744.3609 [M + H^+^], 744.3603 found. HPLC purity 98.8%.

##### 4-(4-{4-[(2*S*)-5-Amino-2-{[4-(4-cyanobenzamido)phenyl]formamido}pentanamido]benzamido}-2-hydroxy-3-(propan-2-yloxy)benzamido)benzoic Acid (28)

The amine **87** (17.5 μmol) was coupled with carboxylic acid **10** using general procedure 4. After deprotection of the *tert*-butyl ester using general procedure 7, the crude product was purified by RP HPLC. The product was obtained by deprotection with general procedure 5 followed by RP HPLC purification. White solid, 6.4 mg (48% over 3 steps). ^1^H NMR (700 MHz, DMSO-*d*_6_, 300 K): δ (ppm) = 10.72 (s, 1H), 10.53 (s, 1H), 9.08 (br s, 1H), 8.67 (d, 1H, *J* = 7.6 Hz), 8.13 (d, 2H, *J* = 8.5 Hz), 8.05 (d, 2H, *J* = 8.4 Hz), 7.98 (d, 2H, *J* = 8.8 Hz), 7.92–7.88 (m, 6H), 7.82 (d, 2H, *J* = 8.7 Hz), 7.79 (d, 2H, *J* = 8.6 Hz), 7.62–7.58 (m, 1H), 7.36–7.30 (m, 1H), 4.84 (br s, 1H), 4.66 (dd, 1H, *J* = 7.9 Hz, 14.2 Hz), 2.86 (t, 2H, *J* = 7.7 Hz), 1.95–1.85 (m, 2H), 1.77–1.64 (m, 2H), 1.22 (dd, 6H, *J* = 2.5 Hz, 6.1 Hz). ^13^C NMR (176 MHz, DMSO-*d*_6_, 300 K): δ (ppm) = 171.2, 167.8, 167.2, 166.1, 164.5, 163.6, 144.1, 142.0, 141.7, 138.7, 137.1, 135.4, 132.5, 130.4, 129.2, 129.1, 128.6, 128.5, 128.0, 128.0, 123.4, 119.5, 119.2, 118.9, 118.3, 114.1, 72.1, 53.8, 38.7, 28.5, 24.0, 22.5. HRMS (ESI) calcd 812.3044 [M + H^+^], 812.3040 found. HPLC purity 97.7%.

##### *tert*-Butyl 4-(4-{4-[(2*S*)-2-Aminohex-5-ynamido]benzamido}-2-(prop-2-en-1-yloxy)-3-(propan-2-yloxy)benzamido)benzoate (**89**)

Amine **8** (0.09 mmol) was coupled with (2*S*)-2-({[(9*H*-fluoren-9-yl)methoxy]carbonyl}amino)hex-5-ynoic acid and deprotected using general procedure 8 and deprotected using general procedure 2. Yellow solid, 35 mg (56% over 2 steps). ^1^H NMR (500 MHz, CDCl_3_, 300 K): δ (ppm) = 10.17 (s, 1H), 9.88 (br s, 1H), 8.74 (s, 1H), 8.46 (d, 1H, *J* = 8.9 Hz), 8.03 (d, 1H, *J* = 8.9 Hz), 7.97 (d, 2H, *J* = 8.7 Hz), 7.87 (d, 2H, *J* = 8.8 Hz), 7.77 (d, 2H, *J* = 8.7 Hz), 7.72 (d, 2H, *J* = 8.8 Hz), 6.13 (ddt, 1H, *J* = 5.9 Hz, 10.4 Hz, 16.3 Hz), 5.49 (dd, 1H, *J* = 1.3 Hz, 17.1 Hz), 5.40 (dd, 1H, *J* = 1.1 Hz, 10.4 Hz), 4.74 (hept., 1H, *J* = 6.1 Hz), 4.69 (d, 2H, *J* = 5.9 Hz), 3.71 (dd, *J* = 4.4 Hz, 8.4 Hz), 2.44–2.39 (m, 2H), 2.26 (ddd, 1H, *J* = 4.5 Hz, 7.3 Hz, 11.8 Hz), 2.03 (t, 1H, *J* = 2.6 Hz), 1.81 (td, 1H, *J* = 6.5 Hz, 14.8 Hz), 1.59 (s, 9H), 1.37 (dd, 6H, *J* = 1.2 Hz, 6.1 Hz). ^13^C NMR (126 MHz, CDCl_3_, 300 K): δ (ppm) = 172.8, 165.5, 164.4, 162.8, 149.4, 142.3, 141.5, 139.1, 137.7, 132.3, 130.8, 129.7, 128.2, 127.5, 127.4, 121.6, 120.1, 119.4, 119.1, 115.7, 83.1, 80.9, 76.9, 75.0, 70.1, 55.1, 33.0, 28.4, 22.9, 15.7. HRMS (ESI) calcd 655.3132 [M + H^+^], 655.3126 found.

##### 4-(4-{4-[(2*S*)-2-{[4-(4-Cyanobenzamido)phenyl]formamido}hex-5-ynamido]benzamido}-2-hydroxy-3-(propan-2-yloxy)benzamido)benzoic Acid (29)

The amine **89** (51.9 μmol) was coupled with carboxylic acid **10** using general procedure 4. The product was obtained by deprotection with general procedures 6 and 7. Beige solid, 21 mg (55% over 3 steps). ^1^H NMR (500 MHz, DMSO-*d*_6_, 300 K): δ (ppm) = 12.81 (br s, 1H), 12.30 (s, 1H), 10.69 (s, 1H), 10.60 (s, 1H), 10.47 (s, 1H), 9.40 (s, 1H), 8.63 (d, 1H, *J* = 7.4 Hz), 8.13 (d, 2H, *J* = 8.6 Hz), 8.04 (d, 2H, *J* = 8.4 Hz), 7.99–7.95 (m, 6H), 7.90 (d, 2H, *J* = 8.8 Hz), 7.87–7.84 (m, 3H), 7.82 (d, 2H, *J* = 8.8 Hz), 7.71 (d, 1H, *J* = 8.9 Hz), 4.67 (dd, 1H, *J* = 7.4 Hz, 14.5 Hz), 4.55 (hept., 1H, *J* = 6.1 Hz), 2.85 (t, 1H, *J* = 2.6 Hz), 2.43–2.30 (m, 2H), 2.07 (dd, 2H, *J* = 7.6 Hz, 14.7 Hz), 1.27 (dd, 6H, *J* = 6.1 Hz). ^13^C NMR (126 MHz, DMSO-*d*_6_, 300 K): δ (ppm) = 171.1, 168.5, 166.9, 166.3, 164.4, 164.2, 154.1, 142.4, 142.0, 141.6, 138.7, 137.0, 136.3, 132.5, 130.2, 129.1, 128.6, 128.5, 128.4, 128.3, 126.3, 122.8, 120.7, 119.5, 119.0, 118.3, 114.0, 112.4, 112.1, 83.4, 74.9,71.8, 53.9, 30.3, 22.3, 15.2. HRMS (ESI) calcd 807.2779 [M + H^+^], 807.2773 found. HPLC purity 98.8%.

##### *tert*-Butyl 4-(4-{4-[(2*S*)-2-aminohept-6-ynamido]benzamido}-2-(prop-2-en-1-yloxy)-3-(propan-2-yloxy)benzamido)benzoate (**91**)

Amine **8** (0.09 mmol) was coupled with (2*S*)-2-({[(9*H*-fluoren-9-yl)methoxy]carbonyl}amino)hept-6-ynoic acid using general procedure 8 and deprotected using general procedure 2. Yellow solid, 41 mg (63% over 2 steps). ^1^H NMR (500 MHz, CDCl_3_, 300 K): δ (ppm) = 10.17 (s, 1H), 9.88 (br s, 1H), 8.74 (s, 1H), 8.46 (d, 1H, *J* = 8.9 Hz), 8.04 (d, 1H, *J* = 8.9 Hz), 7.97 (d, 2H, *J* = 8.7 Hz), 7.87 (d, 2H, *J* = 8.7 Hz), 7.77 (d, 2H, *J* = 8.6 Hz), 7.72 (d, 2H, *J* = 8.8 Hz), 6.13 (ddt, 1H, 5.9 Hz, 10.4 Hz, 16.3 Hz), 5.49 (dd, 1H, *J* = 1.3 Hz, 17.1 Hz), 5.40 (dd, 1H, *J* = 1.1 Hz, 10.4 Hz), 4.74 (hept., 1H, *J* = 6.1 Hz), 4.69 (d, 2H, *J* = 5.9 Hz), 3.66–3.59 (m, 1H), 2.29–2.24 (m, 2H), 2.13–2.04 (m, 1H), 1.98 (t, 1H, *J* = 2.6 Hz), 1.82–1.73 (m, 1H), 1.73–1.65 (m, 2H), 1.59 (s, 9H), 1.37 (dd, 6H, *J* = 0.9 Hz, 6.1 Hz). ^13^C NMR (126 MHz, CDCl_3_, 300 K): δ (ppm) = 173.0, 165.5, 164.5, 162.8, 149.4, 142.3, 141.5, 139.1, 137.7, 132.3, 130.8, 129.7, 128.3, 127.5, 127.4, 121.6, 120.1, 119.4, 119.2, 115.8, 83.7, 80.9, 76.9, 75.0, 69.3, 55.2, 33.9, 28.4, 24.8, 22.9, 18.4. HRMS (ESI) calcd 669.3288 [M + H^+^], 669.3283 found.

##### 4-(4-{4-[(2*S*)-2-{[4-(4-Cyanobenzamido)phenyl]formamido}hept-6-ynamido]benzamido}-2-hydroxy-3-(propan-2-yloxy)benzamido)benzoic Acid (**30**)

The amine **91** (55.3 μmol) was coupled with carboxylic acid **10** using general procedure 4. The product was obtained by deprotection with general procedures 6 and 7. Beige solid, 14 mg (29% over 3 steps). ^1^H NMR (700 MHz, DMSO-*d*_6_, 300 K): δ (ppm) = 12.82 (br s, 1H), 12.30 (s, 1H), 10.70 (s, 1H), 10.61 (s, 1H), 10.49 (s, 1H), 9.40 (s, 1H), 8.62 (d, 1H, *J* = 7.3 Hz), 8.13 (d, 2H, *J* = 8.5 Hz), 8.05 (d, 2H, *J* = 8.5 Hz), 7.99–7.95 (m, 6H), 7.89 (d, 2H, *J* = 8.8 Hz), 7.87–7.84 (m, 3H), 7.82 (d, 2H, *J* = 8.8 Hz), 7.71 (d, 1H, *J* = 8.8 Hz), 4.61 (dd, 1H, *J* = 7.3 Hz, 14.6 Hz), 4.55 (hept., 1H, *J* = 6.1 Hz), 2.81 (t, 1H, *J* = 2.6 Hz), 2.26–2.23 (m, 2H), 1.97–1.89 (m, 2H), 1.71–1.65 (m, 1H), 1.58–1.52 (m, 1H), 1.27 (d, 6H, *J* = 6.1 Hz). ^13^C NMR (176 MHz, DMSO-*d*_6_, 300 K): δ (ppm) = 171.6, 168.5, 166.9, 166.1, 164.5, 164.2, 154.1, 142.4, 142.0, 141.6, 138.7, 137.1, 136.3, 132.5, 130.2, 129.1, 128.6, 128.5, 128.4, 128.4, 126.3, 122.8, 120.7, 119.5, 118.8, 118.3, 114.0, 112.4, 112.2, 84.2, 74.9, 71.6, 54.3, 30.7, 25.0, 22.3, 17.6. HRMS (ESI) calcd 821.2935 [M + H^+^], 821.2929 found. HPLC purity 98.7%.

##### *tert*-Butyl 1-[(4-{[4-({4-[(*tert*-Butoxy)carbonyl]phenyl}carbamoyl)-3-(prop-2-en-1-yloxy)-2-(propan-2-yloxy)phenyl]carbamoyl}phenyl)carbamoyl]-2-azabicyclo[2.1.1]hexane-2-carboxylate (**93**)

The aniline **8** (0.18 mol) was coupled with 2-(*tert*-butoxycarbonyl)-2-azabicyclo[2.1.1]hexane-1-carboxylic acid using general procedure 1. Yellow to orange solid, 88 mg (64%). ^1^H NMR (500 MHz, CDCl_3_, 300 K): δ (ppm) = 10.17 (s, 1H), 8.75 (br s, 1H), 8.50 (d, 1H, *J* = 8.9 Hz), 8.07 (d, 1H, *J* = 8.9 Hz), 7.98 (d, 2H, *J* = 8.8 Hz), 7.90 (d, 2H, *J* = 8.8 Hz), 7.76–7.72 (m, 4H), 6.14 (ddt, 1H, *J* = 5.9 Hz, 10.4 Hz, 16.3 Hz), 5.49 (dq, 1H, *J* = 1.4 Hz, 17.1 Hz), 5.41 (dq, 1H, *J* = 1.0 Hz, 10.4 Hz), 4.75 (quint. 1H, *J* = 6.2 Hz), 4.69 (dt, 2H, *J* = 1.2 Hz, 5.9 Hz), 3.58 (br s, 2H), 2.83 (t, 1H, *J* = 3.2 Hz), 2.31–2.28 (m, 2H), 1.73 (dd, 2H, *J* = 1.9 Hz, 4.7 Hz), 1.60 (s, 9H), 1.39 (d, 6H, *J* = 6.2 Hz), 1.36 (s, 9H). ^13^C NMR (126 MHz, CDCl_3_, 300 K): δ (ppm) = 167.1, 165.6, 164.4, 162.3, 158.5, 149.4, 142.3, 141.8, 139.1, 137.8, 132.2, 130.8, 129.6, 128.4, 127.6, 127.4, 121.6, 120.2, 119.2, 119.2, 115.8, 81.7, 80.9, 75.1, 73.5, 54.2, 42.7, 34.8, 28.4, 28.3, 23.0. HPLC purity 95.0%.

##### 4-[4-(4-{2-[4-(4-Cyanobenzamido)benzoyl]-2-azabicyclo[2.1.1]hexane-1-amido}benzamido)-2-hydroxy-3-(propan-2-yloxy)benzamido]benzoic Acid (**31**)

Step 1:83 mg carbamate **93** (0.11 mmol, 1 equiv) was added to a dry flask and further dried under high vacuum. 0.9 mL dry DCM was added under argon atmosphere and the solution was cooled down to 0 °C. 0.5 mL trifluoroacetic acid (6.5 mmol, 59.3 equiv) was added under argon atmosphere. The solution was stirred for 3 h at 0 °C and controlled over LCMS. After completion, 1 mL DCM was added and the solvent was concentrated under reduced pressure. The residue was coevaporated with DCM three times. The crude product was dried under high vacuum.

Step 2:35 mg carboxylic acid **10** (0.13 mmol, 1.2 equiv) and 46 mg HATU (0.12 mmol, 1.1 equiv) were added to a dry vial and dried under high vacuum. 0.8 mL dry DMF and 76 μL dry DIPEA (0.44 mmol, 4 equiv) were added under argon atmosphere and the reaction was stirred for 30 min at 0 °C. The solution was added to the crude amine trifluoroacetate (0.11 mmol, 1 equiv) in a dry vial under argon atmosphere and stirred at 0 °C. The reaction was controlled over LCMS. After full consumption of the amine, 40 μL aniline was added under argon atmosphere and the reaction was stirred for another 30 min at 0 °C. Six mg tetrakis(triphenylphosphine)-palladium(0) (5.2 μmol, 0.05 equiv) and 1.5 mL dry THF were added under argon atmosphere. The reaction was controlled over LCMS. After full deprotection, the solvent was concentrated under reduced pressure. The DMF was coevaporated with *n*-heptane. The crude product was purified by RP-HPLC. Off-white solid, 43.5 mg (49% over 3 steps). ^1^H NMR (500 MHz, DMSO-*d*_6_, 300 K): δ (ppm) = 12.81 (br s, 1H), 12.29 (br s, 1H), 10.70 (br s, 1H), 9.86 (s, 1H), 9.37 (br s, 1H), 8.11 (d, 2H, *J* = 8.5 Hz), 8.04 (d, 2H, *J* = 8.4 Hz), 7.98–7.92 (m, 4H), 7.89 (d, 2H, *J* = 8.6 Hz), 7.87–7.82 (m, 5H), 7.78 (d, 2H, *J* = 8.3 Hz), 7.68 (d, 1H, *J* = 8.8 Hz), 4.55 (hept., 1H, *J* = 6.1 Hz), 3.64 (br s, 2H), 2.82–2.80 (m, 1H), 2.22–2.17 (m, 2H), 1.78 (d, 2H, *J* = 3.4 Hz), 1.26 (d, 6H, *J* = 6.2 Hz). ^13^C NMR (126 MHz, DMSO-*d*_6_, 300 K): δ (ppm) = 171.9, 168.5, 167.1, 167.0, 164.6, 164.3, 154.4, 142.9, 142.2, 141.5, 138.8, 137.1, 136.5, 132.6, 130.3, 130.0, 129.3, 128.7, 128.2, 128.0, 126.2, 122.9, 120.7, 119.6, 119.3, 118.4, 114.1, 112.6, 112.2, 74.8, 72.2, 55.1, 41.4, 34.5, 22.4. HRMS (ESI) calcd 807.2773 [M + H^+^], 807.2776 found. HPLC purity 97.0%.

##### *tert*-Butyl 4-(4-{4-[(2*S*)-2-({[(9*H*-fluoren-9-yl)methoxy]carbonyl}amino)-3-methylbutanamido]benzamido}-2-(prop-2-en-1-yloxy)-3-(propan-2-yloxy)benzamido)benzoate (**95**)

Amine **8** (200 mg, 0.37 mmol) and Fmoc-l-valine (174 mg, 0.51 mmol, 1.40 equiv) were dissolved in EtOAc (800 μL) and pyridine (180 μL, 2.20 mmol, 6.00 equiv) was added. T3P (50% in EtOAc, 900 μL, 1.47 mmol, 4.00 equiv) was added dropwise at 0 °C. The mixture was stirred for 4 h while warming to rt. The mixture was diluted with EtOAc and the organic phase was washed with 1 M HCl solution, sat. NaHCO_3_ solution, brine, dried over Na_2_SO_4_, filtered and concentrated under reduced pressure. The residue was purified by column chromatography (dry load, 40% EtOAc in hexane) to furnish the product (324 mg, quant.) as yellowish amorphous solid, which was directly used in the next step.

##### *tert*-Butyl 4-(4-{4-[(2*S*)-2-{[4-(4-Cyanobenzamido)phenyl]formamido}-3-methylbutanamido]benzamido}-2-(prop-2-en-1-yloxy)-3-(propan-2-yloxy)benzamido)benzoate (**96**)

Step 1: Carbamate **95** (324 mg, 0.37 mmol, 1.00 equiv) was dissolved in MeCN (4 mL) and piperidine (1 mL) was added. The mixture was stirred at rt for 3 h, before it was concentrated under reduced pressure. The residue was coevaporated with MeCN (3×) to furnish the amine, which was used in the next step without further purification. Step 2: Acid **10** (136 mg, 0.51 mmol, 1.40 equiv) and HATU (195 mg, 0.51 mmol, 1.40 equiv) were dissolved in DMF (9 mL) and DIPEA (200 μL, 1.10 mmol, 3.00 equiv) was added. The mixture was stirred for 5 min before it was transferred to a stirred solution of the amine (236 mg, 0.37 mmol, 1.00 equiv) in DMF (5 mL). The resulting mixture was stirred at rt for 23 h. EtOAc was added and the organic phase was washed with 0.1 M HCl solution, brine, dried over Na_2_SO_4_, filtered and concentrated under reduced pressure. The residue was purified by column chromatography (2% MeOH in CH_2_Cl_2_) to furnish the product (168 mg, 0.19 mmol, 51% over three steps) as yellowish amorphous solid. [α]_D_^22^ = +5.5° (c 0.1, MeOH). ^1^H NMR (600 MHz, DMSO-*d*_6_) = δ 10.69 (s, 1H), 10.53 (s, 1H), 10.52 (s, 1H), 9.52 (s, 1H), 8.49–8.47 (d, *J* = 8.1 Hz, 1H), 8.14–8.12 (d, *J* = 8.6 Hz, 2H), 8.05–8.04 (d, *J* = 8.6 Hz, 2H), 7.99–7.96 (m, 4H), 7.90–7.88 (m, 4H), 7.84–7.81 (m, 5H), 7.41–7.40 (d, *J* = 8.5 Hz, 1H), 6.05–5.98 (m, 1H), 5.39–5.35 (dq, *J* = 1.6, 17.2 Hz, 1H), 5.21–5.19 (dq, *J* = 1.6, 10.5 Hz, 1H), 4.61–4.60 (d, *J* = 5.5 Hz, 1H), 4.53–4.47 (sept, *J* = 6.1 Hz, 1H), 4.45–4.42 (t, *J* = 8.3 Hz, 1H), 2.28–2.20 (m, 1H), 1.55 (s, 9H), 1.26–1.25 (d, *J* = 6.1 Hz, 6H), 1.04–0.99 (dd, *J* = 6.7 Hz, 6H) ppm. ^13^C NMR (151 MHz, DMSO-d_6_) = δ 171.2, 166.2, 164.6, 164.5, 164.4, 164.3, 149.5, 143.0, 142.5, 142.1, 141.5, 138.7, 135.7, 133.6, 132.5, 130.1, 129.3, 128.6, 128.5, 128.4, 127.1, 126.0, 123.6, 119.5, 118.9, 118.8, 118.7, 118.3, 117.8, 114.0, 80.3, 76.2, 74.8, 60.3, 30.0, 27.9, 22.3, 19.3, 19.2 ppm. HRMS (ESI^+^) calcd for C_51_H_52_N_6_O_9_Na [M + Na]^+^: 915.3693; found: 915.3715.

##### *tert*-Butyl 4-(4-{4-[(2*S*)-2-{[4-(4-Cyanobenzamido)phenyl]formamido}-3-methylbutanamido]benzamido}-2-hydroxy-3-(propan-2-yloxy)benzamido)benzoate (**97**)

Allyl ether **96** (152 mg, 0.17 mmol, 1.00 equiv) was dissolved in THF (9 mL). Aniline (50.0 μL, 0.56 mmol, 3.30 equiv) and Pd(PPh_3_)_4_ (20.0 mg, 0.02 mmol, 10 mol %) were added and the mixture was stirred at rt for 2 h. The mixture was concentrated under reduced pressure. The residue was purified by column chromatography (dry load, 3% MeOH in CH_2_Cl_2_) to obtain a brown amorphous solid (169 mg), which was stirred in CH_2_Cl_2_. The mixture was filtered and the precipitate was washed with CH_2_Cl_2_ to furnish the product (78.4 mg, 0.09 mmol, 54%) as beige amorphous solid. [α]_D_^22^ = +7.8° (c 0.2, MeOH). ^1^H NMR (600 MHz, DMSO-*d*_6_) = δ 12.29 (s, 1H), 10.69 (s, 1H), 10.61 (s, 1H), 10.54 (s, 1H), 9.39 (s, 1H), 8.49–8.48 (d, *J* = 8.1 Hz, 1H), 8.14–8.12 (d, *J* = 8.5 Hz, 2H), 8.05–8.04 (d, *J* = 8.6 Hz, 2H), 7.97–7.96 (m, 4H), 7.93–7.92 (d, *J* = 8.7 Hz, 2H), 7.89–7.88 (d, *J* = 8.8 Hz, 2H), 7.86–7.82 (m, 5H), 7.71–7.70 (d, *J* = 8.7 Hz, 1H), 4.58–4.52 (sept, *J* = 6.1 Hz, 1H), 4.45–4.42 (t, *J* = 8.3 Hz, 1H), 2.26–2.21 (m, 1H), 1.55 (s, 9H), 1.27–1.26 (dd, *J* = 1.2, 6.1 Hz, 6H), 1.04–0.99 (dd, *J* = 6.7 Hz, 6H) ppm. ^13^C NMR (151 MHz, DMSO-*d*_6_) = δ 171.2, 168.5, 166.2, 164.5, 164.4, 164.2, 154.1, 142.2, 142.0, 141.5, 138.7, 137.0, 136.3, 132.5, 129.9, 129.3, 128.6, 128.5, 128.4, 126.8, 122.8, 120.7, 119.5, 118.8, 118.3, 114.0, 112.4, 112.2, 80.5, 74.8, 60.3, 30.0, 27.8, 22.3, 19.3, 19.2 ppm. HRMS (ESI^+^) calcd for C_48_H_47_N_6_O_9_ [M – H]^−^: 851.3405; found: 851.3419.

##### 4-(4-{4-[(2*S*)-2-{[4-(4-Cyanobenzamido)phenyl]formamido}-3-methylbutanamido]benzamido}-2-hydroxy-3-(propan-2-yloxy)benzamido)benzoic Acid (**32**)

Ester **97** (34.7 mg, 0.04 mmol, 1.00 equiv) was dissolved in precooled TFA (2 mL) at 0 °C. The mixture was stirred for 30 min while warming to rt. Et_2_O was added at 0 °C. The precipitate was filtered off and washed with an excess of Et_2_O to furnish the product (21.4 mg, 0.03 mmol, 66%) as beige amorphous solid. [α]_D_^22^ = +5.7° (c 0.1 MeOH). ^1^H NMR (500 MHz, DMSO-*d*_6_) = δ 12.82 (s, 1H), 12.30 (s, 1H), 10.69 (s, 1H), 10.60 (s, 1H), 10.54 (s, 1H), 9.40 (s, 1H), 8.49 (d, *J* = 8.0 Hz, 1H), 8.14–8.12 (d, *J* = 8.5 Hz, 2H), 8.06–8.04 (d, *J* = 8.4 Hz, 2H), 7.97–7.95 (m, 6H), 7.89–7.82 (m, 7H), 7.72–7.70 (d, *J* = 8.9 Hz, 1H), 4.59–4.51 (sept, *J* = 6.1 Hz, 1H), 4.46–4.42 (t, *J* = 8.4 Hz, 1H), 2.28–2.19 (sept, *J* = 6.7 Hz, 1H), 1.27–1.26 (d, *J* = 6.1 Hz, 6H), 1.04–0.99 (dd, *J* = 6.7, 18.5 Hz, 6H) ppm. ^13^C NMR (126 MHz, DMSO-*d*_6_) = δ 171.2, 168.5, 166.9, 166.2, 164.4, 164.2, 154.1, 142.2, 142.0, 141.5, 138.7, 138.6, 137.0, 136.3, 132.5, 130.2, 129.3, 128.6, 128.5, 128.4, 126.3, 122.8, 120.7, 119.5, 118.8, 118.3, 114.0, 112.4, 112.2, 74.8, 60.3, 30.0, 22.3, 19.3, 19.2 ppm. HRMS (ESI^+^) calcd for C_44_H_39_N_6_O_9_ [M – H]^−^: 795.2779; found: 795.2773.

##### *tert*-Butyl 4-(4-{4-[(2*R*)-2-{[(*tert*-Butoxy)carbonyl]amino}-3-methylbutanamido]benzamido}-2-(prop-2-en-1-yloxy)-3-(propan-2-yloxy)benzamido)benzoate (**98**)

Aniline **8** (200 mg, 0.37 mmol) and Boc-d-Val (112 mg, 0.51 mmol, 1.40 equiv) were dissolved in EtOAc (1 mL) and pyridine (87 μL, 1.10 mmol, 3.00 equiv) was added. T3P (50% in EtOAc, 440 μL, 0.73 mmol, 2.00 equiv) was added at 0 °C and the mixture was stirred at rt for 4 h. EtOAc was added and the organic phase was washed with a 1 M HCl solution, a sat. NaHCO_3_ solution, brine, dried over Na_2_SO_4_, filtered and concentrated under reduced pressure. The residue was purified by column chromatography (1% MeOH in CH_2_Cl_2_) to furnish the product (232 mg, 0.31 mmol, 85%) as yellowish amorphous solid. [α]_D_^22^ = +1.6° (c 0.2, MeOH). ^1^H NMR (600 MHz, DMSO-*d*_6_) = 10.52 (s, 1H), 10.30 (s, 1H), 9.51 (s, 1H), 7.98–7.96 (d, *J* = 8.6 Hz, 2H), 7.90–7.88 (d, *J* = 8.6 Hz, 2H), 7.84–7.82 (m, 3H), 7.78–7.77 (d, *J* = 8.6 Hz, 2H), 7.41–7.40 (d, *J* = 8.5 Hz, 1H), 6.76–6.74 (d, *J* = 8.7 Hz, 1H), 6.05–5.99 (m, 1H), 5.39–5.38 (d, *J* = 17.2 Hz, 1H), 5.21–5.19 (d, *J* = 10.3 Hz, 1H), 4.61–4.60 (d, *J* = 5.4 Hz, 1H), 4.53–4.47 (sept, *J* = 6.2 Hz, 1H), 3.96–3.94 (t, *J* = 7.8 Hz, 1H), 2.02–1.99 (m, 1H), 1.55 (s, 9H), 1.40 (s, 9H), 1.27–1.26 (d, *J* = 6.1 Hz, 6H), 0.92–0.91 (d, *J* = 6.5 Hz, 6H) ppm. ^13^C NMR (151 MHz, DMSO-*d*_6_) = 171.38, 164.6, 164.5, 164.3, 155.7, 149.5, 143.0, 142.5, 142.1, 135.7, 133.6, 130.1, 128.5, 128.3, 127.1, 126.0, 123.6, 118.9, 118.8, 118.7, 117.8, 80.3, 78.1, 76.2, 74.3, 60.8, 30.2, 28.1, 27.9, 22.3, 19.2, 18.5 ppm. HRMS (ESI) calcd for C_41_H_52_N_4_O_9_Na [M + Na]^+^: 767.3632; found: 767.3619.

##### *tert*-Butyl 4-(4-{4-[(2*R*)-2-{[4-(4-Cyanobenzamido)phenyl]formamido}-3-methylbutanamido]benzamido}-2-(prop-2-en-1-yloxy)-3-(propan-2-yloxy)benzamido)benzoate (**99**)

Step 1: Carbamate **98** (220 mg, 0.30 mmol) was dissolved in HCl (4 M in 1,4-dioxane, 7.30 mL, 29.5 mmol, 100 equiv) at 0 °C. The mixture was warmed up to rt and stirred for 15 min. The solution was transferred to a stirred suspension of EtOAc (190 mL) and a sat. NaHCO_3_ solution (190 mL). The aqueous phase was extracted with EtOAc and the combined organic phases were washed with brine, dried over MgSO_4_, filtered and concentrated under reduced pressure. The crude product was used in the next step without further purification. Step 2: Compound **99** (129 mg, 0.14 mmol, 49%) was synthesized from carboxylic acid 10 (94.4 mg, 0.35 mmol) and the amine (190 mg, 0.30 mmol) in accordance to the procedure of compound **96**. [α]_D_^21^ = −4.9° (c 0.2, MeOH).

##### *tert*-Butyl 4-(4-{4-[(2*R*)-2-{[4-(4-Cyanobenzamido)phenyl]formamido}-3-methylbutanamido]benzamido}-2-hydroxy-3-(propan-2-yloxy)benzamido)benzoate (**100**)

Compound **100** (88.3 mg, 0.10 mmol, 75%) was synthesized from allyl ether **99** (124 mg, 0.14 mmol) in accordance to the procedure of compound **97**. [α]_D_^21^ = −3.1° (c 0.1, MeOH).

##### 4-(4-{4-[(2*R*)-2-{[4-(4-Cyanobenzamido)phenyl]formamido}-3-methylbutanamido]benzamido}-2-hydroxy-3-(propan-2-yloxy)benzamido)benzoic Acid (**33**)

**33** (52.1 mg, 0.07 mmol, 65%) was synthesized from ester **100** (85.7 mg, 0.10 mmol) in accordance to the procedure of compound **32**. [α]_D_^21^ = −6.1° (c 0.1 MeOH).

##### 4-(4-{4-[(2*S*)-2-{[4-(4-Cyanobenzamido)phenyl]formamido}-3-(methylamino)propanamido]benzamido}-2-hydroxy-3-(propan-2-yloxy)benzamido)benzoic Acid (**34**)

Amine **8** (0.15 mmol) was coupled with (2*S*)-3-{[(*tert*-butoxy)carbonyl](methyl)amino}-2-({[(9*H*-fluoren-9-yl)methoxy]carbonyl}amino)propanoic acid using general procedure 1, deprotected and coupled with carboxylic acid **10** using general procedure 10 and deprotected by general procedures 6 and 7. Off-white solid, 28 mg (24% over 5 steps). ^1^H NMR (500 MHz, DMSO-*d*_6_, 300 K): δ (ppm) = 10.72 (s, 1H), 9.26 (s, 1H), 8.81 (d, 1H, *J* = 6.2 Hz), 8.13 (d, 2H, *J* = 8.5 Hz), 8.04 (d, 2H, *J* = 8.4 Hz), 7.99 (d, 2H, *J* = 8.8 Hz), 7.96–7.89 (m, 6H), 7.84–7.80 (m, 4H), 7.75 (d, 1H, *J* = 8.8 Hz), 7.54 (d, 1H, *J* = 8.8 Hz), 4.96–4.89 (m, 1H), 4.68 (hept. 1H, *J* = 6.1 Hz), 3.25 (ddd, *J* = 6.7 Hz, 12.5 Hz, 20.8 Hz), 2.55 (s, 3H), 1.25 (d, 6H, *J* = 6.1 Hz). ^13^C NMR (126 MHz, DMSO-*d*_6_, 300 K): δ (ppm) = 169.1, 168.1, 167.3, 166.3, 164.5, 163.9, 142.7, 142.0, 141.8, 138.6, 136.7, 136.2, 132.5, 130.2, 129.0, 128.6, 128.5, 128.2, 126.1, 123.1, 120.0, 119.5, 119.2, 118.3, 114.1, 113.4, 73.7, 52.3, 50.3, 34.3, 22.4. HRMS (ESI) calcd 798.2882 [M + H^+^], 798.2883 found. HPLC purity 98.0%.

##### (2*S*)-2-({[(9*H*-Fluoren-9-yl)methoxy]carbonyl}amino)-3-formamidopropanoic Acid (**104**)

The compound was prepared according to the established literature procedure; see M. Serafini, A. Griglio, S. Viarengo, S. Aprile, T. Pirali, *Org. Biomol. Chem.***2017**, 15, 6604–6612. 100 mg Amine **103** (0.31 mmol, 1 equiv) was dissolved in 0.3 mL formic acid. The flask was cooled down to 0 °C and 0.23 mL acetic anhydride (2.44 mmol, 8 equiv) was added. The flask was sealed and the reaction was stirred overnight. After completion, the 1 mL water was added. The residue was lyophilized to remove the solvent. White solid, 97.8 mg (90%). ^1^H NMR (500 MHz, DMSO-*d*_6_, 300 K): δ (ppm) = 12.85 (br s, 1H), 8.10 (t, 1H, *J* = 5.6 Hz), 8.03 (d, 1H, *J* = 1.1 Hz), 7.90 (d, 2H, *J* = 7.6 Hz), 7.72 (d, 2H, *J* = 7.4 Hz), 7.61 (d, 1H, *J* = 8.3 Hz), 7.42 (t, 2H, *J* = 7.4 Hz), 7.33 (t, 2H, *J* = 7.5 Hz), 4.32–4.28 (m, 2H), 4.23 (t, 1H, *J* = 6.9 Hz), 4.10 (td, 1H, *J* = 5.1 Hz, 8.2 Hz), 3.55 (dt, 1H, *J* = 5.4 Hz, 13.3 Hz). ^13^C NMR (126 MHz, DMSO-*d*_6_, 300 K): δ (ppm) = 171.9, 161.6, 156.0, 143.8, 140.7, 127.7, 127.1, 125.3, 120.1, 65.7, 53.7, 46.6, 38.3. HPLC purity 98.2%.

##### *tert*-Butyl 4-(4-{4-[(2*S*)-2-({[(9*H*-Fluoren-9-yl)methoxy]carbonyl}amino)-3-formamidopropanamido]benzamido}-2-(prop-2-en-1-yloxy)-3-(propan-2-yloxy)benzamido)benzoate (**35**)

The aniline **8** (0.18 mol) was coupled with carboxylic acid **104** using general procedure 1, coupled with **10** using general procedure 10 and deprotected by general procedures 6 and 7. Off-white solid, 16 mg (11% over 4 steps). ^1^H NMR (500 MHz, DMSO-*d*_6_, 300 K): δ (ppm) = 12.77 (br s, 1H), 12.31 (br s, 1H), 10.72 (s, 1H), 10.48 (s, 1H), 9.36 (s, 1H), 8.64 (d, 1H, *J* = 7.2 Hz), 8.30 (dd, 1H, *J* = 5.6 Hz, 6.6 Hz), 8.13 (d, 2H, *J* = 8.5 Hz), 8.10 (d, 1H, *J* = 1.3 Hz), 8.05 (d, 2H, *J* = 8.5 Hz), 7.97–7.93 (m, 6H), 7.91 (d, 2H, *J* = 8.9 Hz), 7.85 (d, 2H, *J* = 8.8 Hz), 7.83–7.79 (m, 3H), 7.63 (d, 1H, *J* = 7.9 Hz), 4.70 (q, 1H, *J* = 6.5 Hz), 4.63–4.55 (m, 1H), 3.64 (t, 2H, *J* = 6.1 Hz), 1.26 (d, 6H, *J* = 6.1 Hz). ^13^C NMR (126 MHz, DMSO-*d*_6_, 300 K): δ (ppm) = 169.3, 168.4, 166.9, 165.9, 164.5, 164.1, 162.1, 142.3, 142.2, 141.7, 138.7, 136.7, 136.5, 132.5, 130.2, 129.0, 128.6, 128.6, 128.4, 128.2, 122.9, 120.5, 119.6, 119.1, 118.3, 114.1, 74.4, 54.9, 38.8, 22.4. HRMS (ESI) calcd 812.2675 [M + H^+^], 812.2675 found. HPLC purity 99.4%.

##### Prop-2-en-1-yl (2*S*)-3-[({[(*tert*-Butoxy)carbonyl]amino}sulfonyl)amino]-2-({[(9*H*-fluoren-9-yl)methoxy]carbonyl}amino)propanoate (**106**)

The compound was prepared according to the established literature procedure; see R.H. Crampton, M. Fox, S. Woodward, *Tetrahedron: Asymmetry***2013**, 24, 599–605. In a dry flask, 50.0 μL *tert*-butanol (0.52 mmol, 1.05 equiv) was dissolved in 0.7 mL dry toluene under argon atmosphere and cooled down to 0 °C. 45.0 μL chlorosulfonyl isocyanate (0.52 mmol, 1.04 equiv) was added dropwise. The mixture was stirred for 45 min at 0 °C. 0.12 mL dry pyridine (1.49 mmol, 3 equiv) was added dropwise under argon atmosphere and a precipitate formed. The supernatant was added to 200 mg amine hydrochloride **105**·HCl (0.5 mmol, 1 equiv) under argon atmosphere at 0 °C. The remaining precipitate was transferred after addition of 1.6 mL dry acetonitrile. The reaction was warmed up to room temperature and stirred overnight. After completion, 0.1 mL methanol was added and the solvent was concentrated under reduced pressure. The crude product was purified by flash chromatography. White solid, 148 mg (55%). ^1^H NMR (500 MHz, DMSO-*d*_6_, 300 K): δ (ppm) = 10.98 (br s, 1H), 7.90 (d, 2H, *J* = 7.5 Hz), 7.70 (d, 2H, *J* = 7.9 Hz), 7.66 (t, 1H, *J* = 6.3 Hz), 7.42 (t, 2H, *J* = 7.4 Hz), 7.34 (t, 2H, J = 7.2 Hz), 5.88 (ddd, 1H, *J* = 5.2 Hz, 10.5 Hz, 22.1 Hz), 5.31 (d, 1H, *J* = 17.3 Hz), 5.19 (d, 1H, *J* = 10.5 Hz), 4.62–4.54 (m, 2H), 4.36–4.22 (m, 4H), 3.31–3.26 (m, 2H), 1.42 (s, 9H). ^13^C NMR (126 MHz, DMSO-*d*_6_, 300 K): δ (ppm) = 169.8, 155.9, 150.6, 143.7, 140.7, 132.3, 127.7, 127.1, 125.2, 120.2, 117.7, 81.5, 65.8, 65.1, 53.9, 46.6, 31.3, 27.7. HPLC purity 96.1%.

##### (2*S*)-3-[({[(*tert*-Butoxy)carbonyl]amino}sulfonyl)amino]-2-({[(9*H*-fluoren-9-yl)methoxy]carbonyl}amino)propanoic Acid (**107**)

144.0 mg ester **106** (0.26 mmol, 1 equiv) was added to a dry vial and further dried under high vacuum. 1.7 mL dry THF and 0.1 mL phenylsilane (0.81 mmol, 3.1 equiv) and 8 mg tetrakis(triphenylphosphine)palladium(0) (7 μmol, 0.026 equiv) were added under argon atmosphere. The reaction was stirred for 3 h at room temperature and controlled over LCMS. After completion, the crude product was directly purified by flash chromatography (DCM/MeOH). Brown solid, 113 mg (84%). ^1^H NMR (500 MHz, DMSO-*d*_6_, 300 K): δ (ppm) = 12.85 (br s, 1H), 10.99 (br s, 1H), 7.90 (d, 2H, *J* = 7.5 Hz), 7.72 (d, 2H, *J* = 7.4 Hz), 7.65–7.60 (m, 1H), 7.42 (t, 2H, *J* = 7.5 Hz), 7.34 (t, 2H, *J* = 7.4 Hz), 4.34–4.12 (m, 4H), 3.29–3.21 (m, 2H), 1.42 (s, 9H). ^13^C NMR (126 MHz, DMSO-*d*_6_, 300 K): δ (ppm) = 171.5, 155.9, 150.7, 143.8, 140.7, 128.8, 127.7, 127.1, 125.2, 120.1, 81.4, 65.8, 53.7, 46.6, 43.9, 27.7. HPLC purity 97.6%.

##### *tert*-Butyl 4-(4-{4-[(2*S*)-3-[({[(*tert*-Butoxy)carbonyl]amino}sulfonyl)amino]-2-({[(9*H*-fluoren-9-yl)methoxy]carbonyl}amino)propanamido]benzamido}-2-(prop-2-en-1-yloxy)-3-(propan-2-yloxy)benzamido)benzoate (**108**)

The aniline **8** (0.16 mol) was coupled with carboxylic acid **107** using general procedure 1. Yellow solid, 97 mg (57%). ^1^H NMR (700 MHz, CDCl_3_, 300 K): δ (ppm) = 10.17 (s, 1H), 8.99 (br s, 1H), 8.73 (s, 1H), 8.48 (d, 1H, *J* = 8.9 Hz), 8.06 (d, 1H, *J* = 8.9 Hz), 7.98 (d, 2H, *J* = 8.6 Hz), 7.87 (d, 2H, *J* = 8.6 Hz), 7.76 (d, 2H, *J* = 7.6 Hz), 7.73 (d, 2H, *J* = 8.7 Hz), 7.68 (d, 2H, *J* = 8.5 Hz), 7.61–7.58 (m, 3H), 7.39 (dd, 2H, *J* = 6.4 Hz, 13.3 Hz), 7.32–7.27 (m, 2H), 6.19 (br s, 1H), 6.14 (ddt, 1H, *J* = 5.9 Hz, 10.5 Hz, 16.4 Hz), 6.05 (br s, 1H), 5.49 (dd, 1H, *J* = 1.2 Hz, 17.1 Hz), 5.40 (d, 1H, *J* = 10.4 Hz), 4.75 (quint., 1H, *J* = 6.1 Hz), 4.69 (d, 2H, *J* = 5.9 Hz), 4.56–4.51 (m, 1H), 4.50–4.44 (m, 2H), 4.23 (t, 1H, *J* = 7.0 Hz), 3.68–3.54 (m, 2H), 1.60 (s, 9H), 1.51 (s, 9H), 1.36 (d, 6H, *J* = 6.1 Hz). ^13^C NMR (176 MHz, CDCl_3_, 300 K): δ (ppm) = 168.5, 165.6, 164.3, 162.8, 157.5, 150.4, 149.4, 143.6, 143.5, 142.3, 141.5, 140.9, 139.1, 137.6, 132.3, 130.8, 130.5, 128.3, 128.1, 127.6, 127.5, 127.3, 125.2, 121.8, 120.3, 120.2, 120.1, 119.2, 115.8, 84.8, 81.0, 75.1, 68.1, 54.7, 47.1, 45.3, 28.4, 28.1, 23.0. HPLC purity 95.3%.

##### 4-(4-{4-[(2*S*)-2-{[4-(4-Cyanobenzamido)phenyl]formamido}-3-(sulfamoylamino)propanamido]benzamido}-2-hydroxy-3-(propan-2-yloxy)benzamido)benzoic Acid (**36**)

The Fmoc protected amino acid **108** (88.0 μmol) was deprotected and coupled with carboxylic acid **10** using general procedure 10. The product was obtained by deprotection with general procedures 5 and 7. Off-white solid, 38.5 mg (51% over 3 steps). ^1^H NMR (700 MHz, DMSO-*d*_6_, 300 K): δ (ppm) = 12.82 (br s, 1H), 12.29 (br s, 1H), 10.71 (s, 1H), 10.62 (br s, 1H), 10.39 (s, 1H), 9.41 (s, 1H), 8.48 (d, 1H, *J* = 7.2 Hz), 8.13 (d, 2H, *J* = 8.4 Hz), 8.05 (d, 2H, *J* = 8.3 Hz), 7.98–7.95 (m, 6H), 7.91 (d, 2H, *J* = 8.8 Hz), 7.87–7.84 (m, 3H), 7.82 (d, 2H, *J* = 8.7 Hz), 7.70 (d, 1H, *J* = 8.8 Hz), 6.86 (t, 1H, *J* = 6.5 Hz), 6.72 (s, 2H), 4.77 (q, 1H, *J* = 6.5 Hz), 4.55 (hept., 1H, *J* = 6.0 Hz), 3.46–3.40 (m, 2H), 1.26 (d, 6H, *J* = 6.1 Hz). ^13^C NMR (176 MHz, DMSO-*d*_6_, 300 K): δ (ppm) = 169.3, 168.5, 166.9, 165.9, 164.5, 164.2, 154.2, 142.3, 142.0, 141.7, 138.7, 137.0, 136.4, 132.5, 130.2, 129.0, 128.6, 128.5, 128.4, 128.3, 126.3, 122.8, 120.7, 119.5, 119.1, 118.3, 114.1, 112.5, 112.2, 74.8, 54.6, 43.9, 22.3. HRMS (ESI) calcd 863.2454 [M + H^+^], 863.2456 found. HPLC purity 99.5%.

##### 2-Amino-3-methyl-3-nitrobutanoic Acid (**112**)

To a solution of KOH (14.1 g, 251 mmol, 2.20 equiv) in H_2_O (280 mL), 2-nitropropane (20.0 mL, 223 mmol, 1.95 equiv)_3_ (25% in H_2_O, 0.93 mol, 150 mL, 8.54 equiv) and a solution of glyoxylic acid monohydrate (10.5 g, 114 mmol, 1.00 equiv) in H_2_O (40.0 mL) were added. The mixture was stirred for 2 h at ambient temperature. Then, the reaction was terminated by adjusting the pH to 0 with conc. HCl whereby a blue coloration of the solution appeared. The aqueous phase was washed with CH_2_Cl_2_ (3×) and the solvent was removed under reduced pressure. The residue was diluted in EtOH, filtered and the filtrate was concentrated to about half under reduced pressure, mixed in equal parts with Et_2_O, filtered again and aniline (10–30 mL) was added to the filtrate until a turbidity of the solution appeared. The turbid solution was stored at 2–8 °C for 16 h. It was then filtered, the solid was washed with EtOH and dried under high vacuum for 16 h. 2-Amino-3-methyl-3-nitrobutanoic acid (10.0 g, 61.8 mmol, 54%) was obtained as a colorless solid. ^1^H NMR (D_2_O, 400 MHz): δ 4.35 (s, 1H), 1.81 (s, 3H), 1.77 (s, 3H) ppm. ^13^C NMR (D_2_O, 100 MHz): δ 170.2, 88.3, 60.8, 24.6, 23.5 ppm.

##### 2-({[(9*H*-Fluoren-9-yl)methoxy]carbonyl}amino)-3-methyl-3-nitrobutanoic Acid (**113**)

Amine **112** (1.03 g, 6.43 mmol) was dissolved in 1,4-dioxane (30 mL) and a 10% Na_2_CO_3_ solution was added at 0 °C, followed by a solution of FmocCl (1.83 g, 7.07 mmol, 1.10 equiv) in 1,4-dioxane (30 mL) *via* a dropping funnel. The mixture was stirred for 18 h while warming to rt. Afterward, the mixture was diluted with H_2_O and Et_2_O. The aqueous phase was washed with Et_2_O (2×), acidified with a 6 M HCl solution and extracted with EtOAc (2×). The combined organic extracts were washed with brine, dried over MgSO_4_, filtered and concentrated under reduced pressure. The residue was coevaporated with PhMe (4×), MeOH (3×) and CH_2_Cl_2_ (4×) to furnish the product (1.40 g, 3.65 mmol, 57%) as colorless solid. ^1^H NMR (400 MHz, DMSO-*d*_6_) = δ 13.40 (s, 1H), 8.12–8.09 (d, *J* = 9.9 Hz, 1H), 7.91–7.89 (d, *J* = 7.6 Hz, 2H), 7.73 (d, *J* = 6.5 Hz, 2H), 7.42 (t, *J* = 7.4 Hz, 2H), 7.32 (dt, *J* = 1.0 Hz, 7.4 Hz, 2H), 4.98 (d, *J* = 9.9 Hz, 1H), 4.40 (dd, *J* = 7.2, 10.4 Hz, 1H), 4.35–4.31 (m, 1H), 4.27–4.23 (m, 1H), 1.58 (s, 3H), 1.50 (s, 3H) ppm. ^13^C NMR (101 MHz, DMSO-*d*_6_) = δ 169.8, 156.6, 143.6, 143.6, 140.7, 140.7, 127.8, 127.8, 127.1, 127.1, 125.3, 125.3, 120.2, 120.2, 88.4, 66.1, 59.2, 46.6, 24.7, 21.1 ppm. HRMS (ESI) calcd for C_20_H_19_N_2_O_6_ [M – H]^−^: 383.1243, found: 383.1244.

##### *tert*-Butyl 4-(4-{4-[2-({[(9*H*-Fluoren-9-yl)methoxy]carbonyl}amino)-3-methyl-3-nitrobutanamido]benzamido}-2-hydroxy-3-(propan-2-yloxy)benzamido)benzoate (**114**)

Amine **8** (100 mg, 0.18 mmol) and the carboxylic acid **113** (98.6 mg, 0.26 mmol, 1.40 equiv) were dissolved in EtOAc (400 μL) and pyridine (45 μL, 0.55 mmol, 3.00 equiv) was added. T3P (50% in EtOAc, 200 μL, 0.33 mmol, 1.80 equiv) was added dropwise at 0 °C. The mixture was stirred at 0 °C for 2 h. H_2_O was added and the aqueous phase was extracted with EtOAc (3×). The combined organic phases were washed with a sat. NaHCO_3_ solution, brine, dried over MgSO_4_, filtered and concentrated under reduced pressure. The residue was purified by column chromatography (dry load, PE/EtOAc = 2:1) to furnish the product (127 mg, 0.14 mmol, 76%) as yellowish solid. ^1^H NMR (500 MHz, DMSO-*d*_6_) = 10.73 (s, 1H), 10.53 (s, 1H), 9.56 (s, 1H), 8.25 (d, *J* = 8.6 Hz, 1H), 7.99 (d, *J* = 8.7 Hz, 2H), 7.90–7.88 (m, 4H), 7.84–7.75 (m, 7H), 7.43–7.39 (m, 3H), 7.34–7.29 (m, 2H), 6.06–5.98 (m, 1H), 5.39–5.35 (dq, *J* = 1.6, 17.2 Hz, 1H), 5.21–5.17 (m, 2H), 4.60 (d, *J* = 5.5 Hz, 2H), 4.50 (sept, *J* = 6.1 Hz, 1H), 4.42–4.36 (m, 1H), 4.29–4.23 (m, 2H), 1.67 (s, 3H), 1.64 (s, 3H), 1.55 (s, 9H), 1.26 (d, *J* = 6.1 Hz, 6H) ppm. ^13^C NMR (126 MHz, DMSO-*d*_6_) = 166.8, 164.6, 164.5, 164.2, 156.2, 149.5, 143.7, 143.7, 143.0, 142.7, 141.4, 140.7, 135.6, 133.7, 130.1, 129.1, 128.5, 127.7, 127.7, 127.2, 127.1, 127.1, 126.0, 125.4, 125.4, 123.6, 120.1, 120.1, 119.4, 119.1, 118.8, 117.8, 89.0, 80.3, 76.3, 74.3, 66.2, 60.5, 46.6, 27.9, 23.2, 22.3 ppm. HRMS (ESI) calcd for C_51_H_53_N_5_O_11_Na [M + Na]^+^: 934.3639; found: 934.3634.

##### *tert*-Butyl 4-{4-[4-(2-Amino-3-methyl-3-nitrobutanamido)benzamido]-2-hydroxy-3-(propan-2-yloxy)benzamido}benzoate (**115**)

The carbamate **114** (400 mg, 0.44 mmol) was dissolved in piperidine (1.3 mL) and MeCN (5.3 mL) and the mixture was stirred at rt for 2 h, before it was concentrated under reduced pressure and coevaporated with MeCN (3×) to furnish the product (quant.) as yellow solid, which was used in the next step without further purification.

##### *tert*-Butyl 4-{4-[4-(2-{[4-(4-Cyanobenzamido)phenyl]formamido}-3-methyl-3-nitrobutanamido)benzamido]-2-(prop-2-en-1-yloxy)-3-(propan-2-yloxy)benzamido}benzoate (**116**)

DIPEA (230 μL, 1.32 mmol, 3.00 equiv) was added dropwise to a stirred solution of HATU (200 mg, 0.53 mmol, 1.20 equiv) and the carboxylic acid **10** (140 mg, 0.53 mmol, 1.20 equiv) in DMF (11 mL). The solution was stirred for 5 min and then transferred to a stirred solution of the amine **115** (303 mg, 0.44 mmol) in DMF (6 mL) at 0 °C. The reaction mixture was stirred at 20 h while warming to rt. The mixture was diluted with EtOAc and washed with a 1 M HCl solution, brine, dried over MgSO_4_ and concentrated under reduced pressure. The crude residue was purified by column chromatography (2% MeOH in CH_2_Cl_2_) to furnish the product (315 mg, 0.34 mmol, 77%) as orange amorphous solid. ^1^H NMR (500 MHz, DMSO-*d*_6_) δ = 10.71 (s, 1H), 10.70 (s, 1H), 10.53 (s, 1H), 9.56 (s, 1H), 8.77 (d, *J* = 9.2 Hz, 1H), 8.13 (d, *J* = 8.5 Hz, 2H), 8.05 (d, *J* = 8.5 Hz, 2H), 7.99 (d, *J* = 8.8 Hz, 2H), 7.96 (d, *J* = 8.8 Hz, 2H), 7.91–7.88 (m, 4H), 7.84–7.79 (m, 5H), 7.41 (d, *J* = 8.4 Hz, 1H), 6.05–5.98 (m, 1H), 5.70 (d, *J* = 9.3 Hz, 1H), 5.37 (dq, *J* = 1.6, 17.2 Hz, 1H), 5.20 (dq, *J* = 1.5, 10.5 Hz, 1H), 4.61 (d, *J* = 5.4 Hz, 2H), 4.49 (sept, *J* = 6.1 Hz, 1H), 1.76 (s, 3H), 1.69 (s, 3H), 1.55 (s, 9H), 1.26 (d, *J* = 6.1 Hz, 6H) ppm. ^13^C NMR (126 MHz, DMSO-*d*_6_) δ = 168.6, 166.8, 166.7, 164.6, 164.5, 164.2, 149.5, 143.0, 142.6, 141.9, 141.5, 138.6, 135.6, 133.7, 132.5, 130.1, 129.1, 128.9, 128.7, 128.6, 128.4, 127.6, 127.2, 126.0, 123.6, 119.9, 119.5, 119.4, 118.8, 117.8, 114.1, 89.0, 80.3, 76.2, 74.3, 58.5, 27.9, 23.2, 22.9, 22.3 ppm. HRMS (ESI) calcd for C_51_H_51_N_7_O_11_Na [M + Na]^+^: 960.3544; found: 960.3557.

##### *tert*-Butyl 4-{4-[4-(2-{[4-(4-Cyanobenzamido)phenyl]formamido}-3-methyl-3-nitrobutanamido)benzamido]-2-hydroxy-3-(propan-2-yloxy)benzamido}benzoate (**117**)

The allyl ether **116** (56.8 mg, 0.06 mmol) was dissolved in THF (2.7 mL). Aniline (18 μL, 0.20 mmol, 3.30 equiv) and Pd(PPh_3_)_4_ (7.0 mg, 6 μmol, 0.10 equiv) were added subsequently and the resulting mixture was stirred at rt for 90 min. The mixture was concentrated under reduced pressure. The crude residue was purified by column chromatography (dry load, 2% MeOH in CH_2_Cl_2_) to furnish the product (57.3 mg, 0.05 mmol, 84%) as yellow solid, which was of 80% purity. The compound was used in the next step without further purification. HRMS (ESI) calcd for C_48_H_46_N_7_O_11_ [M – H]^−^: 896.3255; found: 896.3212.

##### 4-{4-[4-(2-{[4-(4-Cyanobenzamido)phenyl]formamido}-3-methyl-3-nitrobutanamido) benzamido]-2-hydroxy-3-(propan-2-yloxy)benzamido}benzoic Acid (**37**)

The *tert*-butyl ester (19.8 mg, 0.02 mmol) was dissolved in precooled TFA (1.1 mL) at 0 °C with stirring. The solution was warmed up to rt over 30 min. Et_2_O was added at 0 °C. The precipitate was filtered off, washed with excess of Et_2_O and dried *in vacuo* to furnish the product (11.4 mg, 0.01 mmol, 61%) as colorless solid. ^1^H NMR (500 MHz, DMSO-*d*_6_) δ = 12.82 (s, 1H), 12.28 (s, 1H), 10.71 (bs, 2H), 10.60 (s, 1H), 9.44 (s, 1H), 8.77 (d, *J* = 9.3 Hz, 1H), 8.13 (d, *J* = 8.6 Hz, 2H), 8.05 (d, *J* = 8.6 Hz, 2H), 7.98–7.94 (m, 6H), 7.91 (d, *J* = 8.9 Hz, 2H), 7.86–7.84 (m, 3H), 7.81 (d, *J* = 8.8 Hz, 2H), 7.69 (d, *J* = 8.9 Hz, 1H), 5.70 (d, *J* = 9.3 Hz, 1H), 4.53 (sept, *J* = 6.1 Hz, 1H), 1.76 (s, 3H), 1.70 (s, 3H), 1.26 (d, *J* = 6.1 Hz, 6H) ppm. ^13^C NMR (126 MHz, DMSO-*d*_6_) δ = 168.5, 166.9, 166.8, 166.7, 164.5, 164.2, 154.1, 142.0, 141.9, 141.6, 138.6, 137.0, 136.4, 132.5, 130.2, 129.1, 128.9, 128.7, 128.6, 128.3, 126.3, 122.8, 120.7, 119.6, 119.4, 118.3, 114.1, 112.5, 112.3, 89.0, 74.9, 58.5, 23.2, 22.9, 22.3 ppm. HRMS (ESI) calcd for C_44_H_38_N_7_O_11_ [M – H]^−^: 840.2629; found: 840.2632.

##### *tert*-Butyl 4-(4-{4-[(1*S*,2*R*)-1-{[(*tert*-Butoxy)carbonyl]amino}-2-ethenylcyclopropane amido]benzamido}-2-(prop-2-en-1-yloxy)-3-(propan-2-yloxy)benzamido)benzoate (**118**)

After a suspension of the aniline **8** (150 mg, 0.275 mmol, 1.00 equiv) in dry EtOAc (2.00 mL) was cooled down to 0 °C, (1*S*,2*R*)-1-{[(*tert*-butoxy)carbonyl]amino}-2-ethenylcyclopropane-1-carboxylic acid (62 mg, 0.275 mmol, 1.00 equiv) and dry pyridine (75 μL, 0.935 mmol, 3.40 equiv) were added. T3P solution (50% in EtOAc, 327 μL, 0.550 mmol, 2.00 equiv) was added dropwise over 15 min and the resulting solution was stirred for 2.5 h at 0 °C. The reaction mixture was diluted with water (10 mL) and extracted with EtOAc (3 × 10 mL). The combined organic layers were washed with NaHCO_3_ solution (10 mL) and brine (10 mL), dried (Na_2_SO_4_) and concentrated *in vacuo*. The resulting residue was purified by flash chromatography (petroleum ether/EtOAc). Colorless solid, 156 mg (75%). ^1^H NMR (500 MHz, CDCl_3_): δ (ppm) = 1.36–1.39 (m, 6H), 1.51 (s, 10H), 1.60 (s, 9H), 1.91 (s, 1H), 2.17 (q, *J* = 8.6 Hz, 1H), 4.69 (d, *J* = 5.8 Hz, 2H), 4.75 (h, *J* = 6.1 Hz, 1H), 5.12 (dd, *J* = 10.3 Hz, 1.8 Hz, 1H), 5.30 (dd, *J* = 17.1 Hz, 2.0 Hz, 1H), 5.35–5.44 (m, 2H), 5.49 (dd, *J* = 17.1 Hz, 1.4 Hz, 1H), 5.64 (s, 1H), 6.09–6.18 (m, 1H), 7.67 (d, *J* = 8.7 Hz, 2H), 7.73 (d, *J* = 8.7 Hz, 2H), 7.86 (d, *J* = 8.7 Hz, 2H), 7.96–7.99 (m, 2H), 8.06 (d, *J* = 8.9 Hz, 1H), 8.48 (d, *J* = 9.0 Hz, 1H), 8.73 (s, 1H), 9.45 (s, 1H), 10.18 (s, 1H). ^13^C NMR (126 MHz, CDCl_3_): δ (ppm) = 20.0, 22.9–23.0 (m), 28.4, 28.4, 34.0, 43.1, 75.1, 80.9, 81.9, 115.8, 118.0, 119.2, 119.5, 120.2, 121.6, 127.4, 127.6, 128.3, 129.6, 130.8, 132.3, 133.5, 137.8, 139.1, 142.3, 149.4, 157.3, 162.8, 164.4, 165.6, 168.2.

##### (1*S*,2*R*)-1-{[4-({4-[(4-Carboxyphenyl)carbamoyl]-3-(prop-2-en-1-yloxy)-2-(propan-2-yloxy)phenyl}carbamoyl)phenyl]carbamoyl}-2-ethenylcyclopropan-1-aminium Chloride (**119**·HCl)

To the Boc-protected amine **118** (140 mg, 0.185 mmol, 1.00 equiv) was added HCl in dioxane (4.00 M, 1.85 mL, 7.42 mmol, 40.0 equiv) at 0 °C and stirred for 8 h. Afterward, all volatiles were removed, the residue transferred on a fritted funnel, washed with Et_2_O (3 × 1.5 mL) and dried *in vacuo*. Colorless solid, 90 mg (76%).

##### 4-(4-{4-[(1*S*,2*R*)-1-[4-(4-Cyanobenzamido)benzamido]-2-ethenylcyclopropaneamido] benzamido}-2-(prop-2-en-1-yloxy)-3-(propan-2-yloxy)benzamido)benzoic Acid (**120**)

To a solution of the carboxylic acid **10** (47 mg, 0.177 mmol, 1.25 equiv) in dry DMF (518 μL) were added HATU (54 mg, 0.142 mmol, 1.00 equiv) and DIPEA (99 μL, 0.567 mmol, 4.00 equiv) at 0 °C. After 30 min, the amine hydrochloride **119**·HCl (90 mg, 0.142 mmol, 1.00 equiv) dissolved in dry DMF (518 μL) was added and stirring was continued at 0 °C for 30 min before the mixture was stirred overnight at rt. HCl solution (0.5 M, 10 mL) was added to the reaction mixture and extracted with EtOAc (3 × 10 mL). The combined organic layers were washed with HCl solution (0.5 M, 10 mL), water (10 mL) and brine (10 mL), dried over Na_2_SO_4_ and concentrated *in vacuo*. The residue was purified by flash chromatography (CH_2_Cl_2_/MeOH). Colorless solid, 47 mg (39%). ^1^H NMR (700 MHz, DMSO–*d*_6_): δ (ppm) = 1.25 (dd, *J* = 6.1–4.3 Hz, 6H), 1.31–1.35 (m, 1H), 1.91–1.94 (m, 1H), 2.44 (q, *J* = 8.8 Hz, 1H), 4.49 (hept, *J* = 6.2 Hz, 1H), 4.61 (d, *J* = 5.5 Hz, 2H), 5.08–5.11 (m, 1H), 5.20 (dq, *J* = 10.4–1.5 Hz, 1H), 5.31 (d, *J* = 17.1 Hz, 1H), 5.37 (dq, *J* = 17.2–1.7 Hz, 1H), 5.68 (dt, *J* = 17.2–9.7 Hz, 1H), 6.02 (ddt, *J* = 16.1/10.7/5.5 Hz, 1H), 7.40 (d, *J* = 8.4 Hz, 1H), 7.77 (d, *J* = 8.5 Hz, 2H), 7.82 (dd, *J* = 14.1–8.5 Hz, 3H), 7.90 (d, *J* = 8.6 Hz, 2H), 7.93 (d, *J* = 8.6 Hz, 2H), 7.96 (d, *J* = 8.7 Hz, 2H), 7.99 (d, *J* = 8.6 Hz, 2H), 8.03–8.06 (m, 2H), 8.13 (d, *J* = 8.4 Hz, 2H), 9.03 (s, 1H), 9.51 (s, 1H), 10.00 (s, 1H), 10.52 (s, 1H), 10.71 (s, 1H), 12.45 (s, 1H). ^13^C NMR (176 MHz, DMSO–*d*_6_): δ (ppm) = 21.1, 21.4, 22.4, 42.4, 74.3, 76.3, 114.1, 117.1, 117.8, 118.3, 118.9, 119.0, 119.5, 120.7, 123.7, 125.5, 127.2, 128.3, 128.5, 128.7, 128.8, 129.1, 130.4, 132.6, 133.7, 134.7, 135.7, 138.7, 141.8, 142.2, 142.6, 143.1, 149.6, 164.3, 164.5, 164.6, 166.9–167.1 (m), 168.4, 172.1.

##### 4-(4-{4-[(1*S*,2*R*)-1-[4-(4-Cyanobenzamido)benzamido]-2-ethenylcyclopropaneamido] benzamido}-2-hydroxy-3-(propan-2-yloxy)benzamido)benzoic Acid (**38**)

To a solution of the allyl ether **120** (41 mg, 0.048 mmol, 1.00 equiv) in dry THF (365 μL) were added aniline (18 μL, 0.194 mmol, 4.00 equiv) and Pd(PPh_3_)_4_ (2 mg, 0.04 equiv) and the resulting mixture was stirred at rt for 3 h. After completion, HCl solution (0.5 M, 10 mL) was added and extracted with EtOAc (3 × 10 mL). The combined organic layers were washed with HCl solution (0.5 M, 10 mL) and brine (10 mL), dried over Na_2_SO_4_ and concentrated *in vacuo*. The residue was purified by RP-HPLC (H_2_O with 10 mM NH_4_HCO_3_/Acetonitrile) and freeze-dried. Colorless solid, 21 mg (54%). ^1^H NMR (700 MHz, DMSO–*d*_6_): δ (ppm) = 1.24–1.26 (m, 6H), 1.31–1.35 (m, 1H), 1.92 (t, 1H), 2.44 (q, *J* = 8.7 Hz, 1H), 4.54 (hept, *J* = 6.1 Hz, 1H), 5.10 (d, *J* = 12.1 Hz, 1H), 5.31 (d, *J* = 17.1 Hz, 1H), 5.67 (dt, *J* = 17.1/9.7 Hz, 1H), 7.68 (d, *J* = 8.8 Hz, 1H), 7.78 (d, *J* = 8.6 Hz, 2H), 7.82–7.87 (m, 3H), 7.90 (d, *J* = 8.6 Hz, 2H), 7.93 (d, *J* = 8.8 Hz, 2H), 7.95–7.97 (m, 2H), 7.99 (d, *J* = 8.6 Hz, 2H), 8.03–8.06 (m, 2H), 8.11–8.15 (m, 2H), 9.03 (s, 1H), 9.39 (s, 1H), 10.01 (s, 1H), 10.67 (s, 1H), 10.71 (s, 1H), 12.28 (s, 1H), 12.80 (s, 1H). ^13^C NMR (176 MHz, DMSO–*d*_6_): δ (ppm) = 21.4, 22.3, 32.2, 42.4, 74.8, 111.9, 112.5, 114.1, 117.2, 118.3, 119.5, 119.6, 120.7, 122.9, 126.3, 128.2, 128.5, 128.7, 129.1, 130.2, 132.6, 134.7, 136.4, 137.0, 138.7, 141.8, 142.1, 142.3, 154.2, 164.2, 164.5, 166.9, 167.0, 168.5, 168.5. HPLC purity 99.0%.

##### *tert*-Butyl 4-(4-{4-[(1*R*,2*S*)-1-{[(*tert*-Butoxy)carbonyl]amino}-2-ethenylcyclopropane amido]benzamido}-2-(prop-2-en-1-yloxy)-3-(propan-2-loxy)benzamido)benzoate (**121**)

After a suspension of the aniline **8** (150 mg, 0.275 mmol, 1.00 equiv) in dry EtOAc (2.00 mL) was cooled down to 0 °C, (1*R*,2*S*)-1-{[(*tert*-butoxy)carbonyl]amino}-2-ethenylcyclopropane-1-carboxylic acid (62 mg, 0.275 mmol, 1.00 equiv) and dry pyridine (75 μL, 0.935 mmol, 3.40 equiv) were added. T3P solution (50% in EtOAc, 327 μL, 0.550 mmol, 2.00 equiv) was added dropwise over 15 min and the resulting solution was stirred for 2.5 h at 0 °C. The reaction mixture was diluted with water (10 mL) and extracted with EtOAc (3 × 10 mL). The combined organic layers were washed with NaHCO_3_ solution (10 mL) and brine (10 mL), dried (Na_2_SO_4_) and concentrated *in vacuo*. The resulting residue was purified by flash chromatography (petroleum ether/EtOAc). Colorless solid, 71 mg (34%).

##### (1*R*,2*S*)-1-{[4-({4-[(4-Carboxyphenyl)carbamoyl]-3-(prop-2-en-1-yloxy)-2-(propan-2-yloxy)phenyl}carbamoyl)phenyl]carbamoyl}-2-ethenylcyclopropan-1-aminium Chloride (**122**·HCl)

To the Boc-protected amine (68 mg, 0.090 mmol, 1.00 equiv) was added HCl in dioxane (4.00 M, 901 μL, 3.60 mmol, 40.0 equiv) at 0 °C and stirred for 8 h. Afterward, all volatiles were removed, the residue transferred on a fritted funnel, washed with Et_2_O (3 × 1.5 mL) and dried *in vacuo*. Colorless solid, 50 mg.

##### 4-(4-{4-[(1*R*,2*S*)-1-[4-(4-Cyanobenzamido)benzamido]-2-ethenylcyclopropaneamido]benzamido}-2-(prop-2-en-1-yloxy)-3-(propan-2-yloxy)benzamido)benzoic Acid (**123**)

To a solution of the carboxylic acid **10** (58 mg, 0.216 mmol, 1.25 equiv) in dry DMF (300 μL) were added HATU (66 mg, 0.173 mmol, 1.00 equiv) and DIPEA (121 μL, 0.693 mmol, 4.00 equiv) at 0 °C. After 30 min, the amine hydrochloride **122**·HCl (110 mg, 0.173 mmol, 1.00 equiv) dissolved in dry DMF (650 μL) was added and stirring was continued at 0 °C for 30 min before the mixture was stirred overnight at rt. HCl solution (0.5 M, 10 mL) was added to the reaction mixture and extracted with EtOAc (3 × 10 mL). The combined organic layers were washed with HCl solution (0.5 M, 10 mL), water (10 mL) and brine (10 mL), dried over Na_2_SO_4_ and concentrated *in vacuo*. The residue was purified by flash chromatography (CH_2_Cl_2_/MeOH). Colorless solid, 61 mg (41%). ^1^H NMR (500 MHz, DMSO–*d*_6_): δ (ppm) = 1.25 (dd, *J* = 6.1–3.1 Hz, 6H), 1.31–1.35 (m, 1H), 1.93 (dd, *J* = 7.7–5.1 Hz, 1H), 2.45 (q, *J* = 8.7 Hz, 1H), 4.48 (p, *J* = 6.2 Hz, 1H), 4.59–4.63 (m, 2H), 5.06–5.12 (m, 1H), 5.20 (dq, *J* = 10.5–1.4 Hz, 1H), 5.28–5.40 (m, 2H), 5.68 (ddd, *J* = 17.1/10.5/9.1 Hz, 1H), 5.97–6.07 (m, 1H), 7.40 (d, *J* = 8.5 Hz, 1H), 7.76–7.84 (m, 5H), 7.88–8.00 (m, 8H), 8.04–8.06 (m, 2H), 8.11–8.15 (m, 2H), 9.03 (s, 1H), 9.52 (s, 1H), 10.00 (s, 1H), 10.53 (s, 1H), 10.71 (s, 1H), 12.72 (s, 1H). ^13^C NMR (126 MHz, DMSO–*d*_6_): δ (ppm) = 21.4, 22.2–22.4 (m), 32.2, 42.4, 74.3, 76.3, 114.1, 117.1, 117.8, 118.3, 118.9, 119.0, 119.4–119.6 (m), 120.7, 123.6, 125.5, 127.2, 128.3, 128.5, 128.7, 128.9, 129.1, 130.4, 132.6, 133.7, 134.7, 135.6, 138.6, 141.8, 142.1, 142.6, 143.0, 151.1, 164.3, 164.5, 164.6, 166.9, 167.0, 168.4. LC-MS: *m*/*z* = 847.5 (calcd 847.31 for C_48_H_43_N_6_O_9_^+^ [M + H^+^]).

##### 4-(4-{4-[(1*R*,2*S*)-1-[4-(4-Cyanobenzamido)benzamido]-2-ethenylcyclopropaneamido]benzamido}-2-hydroxy-3-(propan-2-yloxy)benzamido)benzoic Acid (**39**)

To a solution of the allyl ether **123** (48 mg, 0.057 mmol, 1.00 equiv) in dry THF (428 μL) were added aniline (21 μL, 0.227 mmol, 4.00 equiv) and Pd(PPh_3_)_4_ (3 mg, 0.04 equiv) and the resulting mixture was stirred at rt for 3 h. After completion, HCl solution (0.5 M, 10 mL) was added and extracted with EtOAc (3 × 10 mL). The combined organic layers were washed with HCl solution (0.5 M, 10 mL) and brine (10 mL), dried over Na_2_SO_4_ and concentrated *in vacuo*. The residue was purified by RP-HPLC (H_2_O with 10 mM NH_4_HCO_3_/Acetonitrile) and freeze-dried. Colorless solid, 24 mg (51%). ^1^H NMR (700 MHz, DMSO–*d*_6_): δ (ppm) = 1.25–1.27 (m, 6H), 1.34 (dt, *J* = 9.4/4.2 Hz, 1H), 1.91–1.94 (m, 1H), 2.44 (q, *J* = 8.7 Hz, 1H), 4.54 (hept, *J* = 6.1 Hz, 1H), 5.08–5.11 (m, 1H), 5.31 (dd, *J* = 17.1–1.8 Hz, 1H), 5.64–5.71 (m, 1H), 7.69 (d, *J* = 8.9 Hz, 1H), 7.77–7.80 (m, 2H), 7.83–7.87 (m, 3H), 7.89–7.92 (m, 2H), 7.92–7.95 (m, 2H), 7.96–7.98 (m, 2H), 7.98–8.01 (m, 2H), 8.04–8.06 (m, 2H), 8.12–8.15 (m, 2H), 9.04 (s, 1H), 9.39 (s, 1H), 10.02 (s, 1H), 10.64 (s, 1H), 10.71 (s, 1H), 12.29 (s, 1H), 12.82 (s, 1H). ^13^C NMR (176 MHz, DMSO–*d*_6_): δ (ppm) = 21.4, 22.3–22.3 (m), 32.2, 42.4, 74.8, 112.1, 112.5, 114.1, 117.1, 118.3, 119.5, 119.6, 120.7, 122.9, 126.3, 128.2, 128.5, 128.6, 129.1, 130.2, 132.5, 134.7, 136.3, 137.0, 138.6, 141.8, 142.0, 142.3, 154.2, 164.2, 164.5, 166.9, 167.0, 168.4, 168.5. HPLC purity 99.0%.

##### *tert*-Butyl 4-(4-{4-[(2*S*)-3-(*tert*-Butoxy)-2-({[(9*H*-fluoren-9-yl)methoxy]carbonyl}amino)propanamido]benzamido}-2-(prop-2-en-1-yloxy)-3-(propan-2-yloxy)benzamido)benzoate (**124**)

Under Ar atmosphere, aniline **8** (100 mg, 1.0 equiv) and (2*S*)-3-(*tert*-butoxy)-2-({[(9*H*-fluoren-9-yl)methoxy]carbonyl}amino)propanoic acid (77 mg, 1.1 equiv) were dissolved in dry EtOAc (1.6 mL). Then dry pyridine (77 μL, 5.0 equiv) and T3P (50% sol. in EtOAc, 0.22 mL, 2.0 equiv) were added and the reaction was stirred at rt while being screened by LCMS. After 2 h, T3P (50% sol. in EtOAc, 0.11 mL, 1.0 equiv) was added again and after 3 h the mixture was partitioned between EtOAc (30 mL) and aq HCl solution (0.1 M, 30 mL). The aqueous phase was extracted with EtOAc (2 × 15 mL). The combined organic phases were dried over Na_2_SO_4_ and dried *in vacuo*. The residue was purified by flash chromatography (solid loading, 80 × theor. prod mass, Cyclohexane/EtOAc, 70:30). Beige sticky foam, 148 mg (89%). ^1^H NMR (500 MHz, CDCl_3_): δ = 10.19 (s, 1H), 9.01 (s, 1H), 8.75 (s, 1H), 8.50 (d, *J* = 8.9, 1H), 8.07 (d, *J* = 8.9, 1H), 7.98 (d, *J* = 8.9, 2H), 7.91 (d, *J* = 8.7, 2H), 7.78 (d, *J* = 7.9, 2H), 7.74 (d, *J* = 8.9, 2H), 7.69 (d, *J* = 8.9, 2H), 7.62 (d, *J* = 7.3, 2H), 7.41 (t, *J* = 7.5, 2H), 7.37–7.27 (m, 2H), 6.14 (ddt, *J* = 17.1, 10.4, 5.8, 1H), 5.85–5.77 (m, 1H), 5.50 (dq, *J* = 17.1, 1.5, 1H), 5.41 (dq, *J* = 10.4, 1.1, 1H), 4.80–4.72 (m, 1H), 4.70 (dt, *J* = 6.0, 1.4, 2H), 4.47 (d, *J* = 7.2, 2H), 4.40 (s, 1H), 4.26 (t, *J* = 6.9, 1H), 3.99–3.88 (m, 1H), 3.48 (t, *J* = 8.8, 1H), 1.60 (s, 9H), 1.39 (d, *J* = 6.3, 6H), 1.28 (d, *J* = 17.7, 9H). ^13^C NMR (126 MHz, CDCl_3_): δ = 169.0, 165.6, 164.3, 162.7, 149.4, 143.8, 143.8, 142.3, 141.5, 141.2, 139.1, 137.7, 132.3, 130.8, 130.1, 128.4, 128.0, 127.6, 127.4, 127.2, 125.2, 121.7, 120.2, 120.2, 119.6, 119.1, 115.8, 80.9, 75.3, 75.1, 67.4, 61.8, 55.0, 47.3, 28.4, 27.7, 23.0. HRMS (ESI) calcd [M + H]^+^: 911.4226; found: 911.4226.

##### *tert*-Butyl 4-(4-{4-[(2*S*)-3-(*tert*-butoxy)-2-{[4-(4-cyanobenzamido)phenyl]formamido}propanamido]benzamido}-2-hydroxy-3-(propan-2-yloxy)benzamido)benzoate (**125**)

Carbamate **124** (57 mg, 1.0 equiv) was dissolved in ACN (1.0 mL) and HNEt_2_ (0.10 mL, 15 equiv) was added. The reaction was stirred at rt while being screened by LCMS. After 1 h, the solvent was removed under reduced pressure and by coevaporation with ACN (2×). The crude product was used without further purification. Under Ar atmosphere, the crude carboxylic acid **10** (18.3 mg, 1.1 equiv) and HATU (26 mg, 1.1 equiv) were added to the amine and the mixture was dissolved in dry DMF (0.65 mL). DIPEA (32 μL, 3.0 equiv) was added and the reaction was stirred at rt for 2 h 10 min. The reaction solution was directly used in the next step. Dry THF (0.65 mL), aniline (17 μL, 3.0 equiv) and Pd(PPh_3_)_4_ (spatula tip) were added to the reaction solution. The reaction was then stirred at rt while being screened by LCMS. After 2 h, the mixture was partitioned between EtOAc (20 mL)/aq HCl solution (0.1 M, 20 mL) and the aqueous phase was extracted with EtOAc (2 × 15 mL). The combined organic layers were dried over Na_2_SO_4_ and all volatiles were removed under reduced pressure. The residue was purified by flash chromatography (solid loading, 100 × theor. prod mass, cyclohexane/acetone, 75:25 → 70:30 → 60:40). Colorless solid, crude 51 mg.

##### 4-(4-{4-[(2*S*)-3-(*tert*-Butoxy)-2-{[4-(4-cyanobenzamido)phenyl]formamido}propanamido]benzamido}-2-hydroxy-3-(propan-2-yloxy)benzamido)benzoic Acid (**40**)

Under Ar atmosphere, to a solution of *tert*-Butyl ester **125** (51 mg, 1.0 equiv) in dry CH_2_Cl_2_ (0.85 mL) were added anisole (12.4 μL, 2.0 equiv) and TFA (0.22 mL, 50 equiv). After 2 h, the flask was flushed with Ar to concentrate the solution by evaporating the CH_2_Cl_2_. After 3.25 h, TFA (0.22 mL, 50 equiv) was added again and after 4.5 h the reaction mixture was stirred at 40 °C for 15 min. All volatiles were removed under reduced pressure, by coevaporation with ACN and by lyophilization overnight. The material was purified by RP-HPLC (H_2_O with 10 mM NH_4_HCO_3_/Acetonitrile) and freeze-dried. Colorless solid, 22 mg (45% over 4 steps). ^1^H NMR (500 MHz, DMSO): δ = 12.84 (s, 1H), 12.30 (s, 1H), 10.72 (s, 1H), 10.66 (br s, 1H), 10.46 (s, 1H), 9.41 (s, 1H), 8.43 (d, *J* = 7.3, 1H), 8.13 (d, *J* = 8.5, 2H), 8.05 (d, *J* = 8.5, 2H), 7.99–7.94 (m, 6H), 7.90 (d, *J* = 9.0, 2H), 7.88–7.81 (m, 5H), 7.69 (d, *J* = 8.9, 1H), 5.14 (t, *J* = 5.7, 1H), 4.69 (q, *J* = 6.0, 1H), 4.54 (hept, *J* = 6.3, 1H), 3.89–3.79 (m, 2H), 1.26 (d, *J* = 6.1, 6H). ^13^C NMR (126 MHz, DMSO): δ = 169.9, 168.5, 166.9, 166.0, 164.5, 164.2, 154.2, 142.4, 142.1, 141.6, 138.7, 137.0, 136.4, 132.6, 130.2, 129.2, 128.7, 128.4, 128.4, 128.3, 126.2, 122.9, 120.7, 119.5, 118.8, 118.3, 114.1, 112.5, 112.1, 74.8, 61.5, 57.1, 22.3. HRMS (ESI) calcd [M + H]^+^: 785.2566; found: 785.2564. HPLC purity 98.2%.

##### *tert*-Butyl 4-(4-{4-[(2*S*,3*S*)-3-(*tert*-Butoxy)-2-({[(9*H*-fluoren-9-yl)methoxy]carbonyl} amino)butanamido]benzamido}-2-(prop-2-en-1-yloxy)-3-(propan-2-yloxy)benzamido)benzoate (**126**)

The amine **8** (0.18 mol) was coupled with (2*S*,3*S*)-3-(*tert*-butoxy)-2-({[(9*H*-fluoren-9-yl) methoxy]carbonyl}amino)butanoic acid using general procedure 1. Yellowish solid, 124.9 mg (74%). ^1^H NMR (700 MHz, CDCl_3_, 300 K): δ (ppm) = 10.17 (s, 1H), 8.74 (s, 1H), 8.50 (d, 1H, *J* = 8.9 Hz), 8.27 (br s, 1H), 8.07 (d, 1H, *J* = 8.9 Hz), 7.98 (d, 2H, *J* = 8.7 Hz), 7.90 (d, 2H, *J* = 8.7 Hz), 7.77 (dd, 2H, *J* = 2.7 Hz, 7.5 Hz), 7.73 (d, 2H, *J* = 8.8 Hz), 7.70 (d, 2H, *J* = 8.7 Hz), 7.61–7.58 (m, 2H), 7.40 (td, 2H, *J* = 2.7 Hz, 7.4 Hz), 7.30 (t, 2H, *J* = 7.3 Hz), 6.14 (ddt, 1H, J = 5.9 Hz, 10.4 Hz, 16.3 Hz), 5.49 (ddd, 1H, *J* = 1.4 Hz, 2.7 Hz, 17.1 Hz), 5.41 (ddd, 1H, *J* = 1.0 Hz, 2.1 Hz, 10.4 Hz), 4.76 (hept., 1H, *J* = 6.1 Hz), 4.69 (d, 2H, *J* = 5.9 Hz), 4.52 (dd, 1H, *J* = 7.0 Hz, 10.6 Hz), 4.46–4.42 (m, 1H), 4.24 (t, 1H, *J* = 6.8 Hz), 3.97–3.91 (m, 1H), 1.60 (s, 9H), 1.39 (dd, 6H, *J* = 1.6 Hz, 6.2 Hz), 1.19 (s, 9H). ^13^C NMR (176 MHz, CDCl_3_, 300 K): δ (ppm) = 165.6, 164.3, 162.8, 149.4, 143.9, 143.8, 142.3, 141.5, 141.1, 139.1, 137.7, 132.3, 130.8, 130.1, 128.4, 127.9, 127.6, 127.4, 127.3, 125.1, 121.7, 120.2, 119.6, 119.2, 115.8, 80.9, 75.4, 75.1, 47.3, 28.6, 28.4, 23.0. HPLC purity 96.8%.

##### 4-(4-{4-[(2*S*,3*S*)-2-{[4-(4-Cyanobenzamido)phenyl]formamido}-3-hydroxybutanamido]benzamido}-2-hydroxy-3-(propan-2-yloxy)benzamido)benzoic Acid (**41**)

Step 1: The amino acid derivative **126** (129.3 μmol) was deprotected using general procedure 2. The crude product was used without further purification. Step 2: The crude amine (129.3 μmol) was coupled with the carboxylic acid **10** using general procedure 4. Steps 3 and 4: The product was obtained by deprotection with general procedures 6 and 7. White solid, 39 mg (38% over 4 steps). ^1^H NMR (700 MHz, DMSO-*d*_6_, 300 K): δ (ppm) = 12.82 (br s, 1H), 12.30 (s, 1H), 10.69 (s, 1H), 10.61 (br s, 1H), 10.40 (br s, 1H), 9.38 (s, 1H), 8.45 (d, 1H, *J* = 8.2 Hz), 8.13 (d, 2H, *J* = 8.5 Hz), 8.05 (d, 2H, *J* = 8.5 Hz), 7.98–7.94 (m, 6H), 7.89 (d, 2H, *J* = 8.8 Hz), 7.87–7.84 (m, 5H), 7.71 (d, 1H, *J* = 8.8 Hz), 5.16 (d, 1H, *J* = 5.2 Hz), 4.57–4.50 (m, 2H), 4.14–4.09 (m, 1H), 1.27 (dd, 6H, *J* = 0.9 Hz, 6.1 Hz), 1.23 (d, 3H, *J* = 6.2 Hz). ^13^C NMR (176 MHz, DMSO-*d*_6_, 300 K): δ (ppm) = 170.5, 168.5, 166.9, 165.9, 164.4, 164.2, 154.1, 142.5, 142.0, 141.6, 138.7, 137.1, 136.3, 132.5, 130.2, 129.2, 128.6, 128.4, 128.3, 128.2, 126.3, 122.8, 120.7, 119.5, 118.8, 118.3, 114.0, 112.4, 112.1, 74.9, 66.5, 60.7, 22.3, 21.0. HPLC purity 99.8%.

##### *tert*-Butyl 4-(4-{4-[(2*S*,3*R*)-3-(*tert*-Butoxy)-2-({[(9*H*-fluoren-9-yl)methoxy]carbonyl} amino)butanamido]benzamido}-2-(prop-2-en-1-yloxy)-3-(propan-2-yloxy)benzamido)benzoate (**128**)

The aniline **8** (0.37 mol) was coupled with (2*S*,3*R*)-3-(*tert*-Butoxy)-2-({[(9*H*-fluoren-9-yl)methoxy]carbonyl}amino)butanoic acid using general procedure 1. Yellowish solid, 312.5 mg (92%). ^1^H NMR (700 MHz, CDCl_3_, 300 K): δ (ppm) = 10.18 (s, 1H), 9.48 (br s, 1H), 8.76 (br s, 1H), 8.52 (d, 1H, *J* = 8.9 Hz), 8.09 (d, 1H, *J* = 8.9 Hz), 8.00 (d, 2H, *J* = 8.7 Hz), 7.92 (d, 2H, *J* = 8.7 Hz), 7.79 (d, 2H, *J* = 7.6 Hz), 7.75 (d, 2H, *J* = 8.7 Hz), 7.68 (d, 2H, *J* = 8.7 Hz), 7.64 (d, 2H, *J* = 7.3 Hz), 7.43 (t, 2H, *J* = 7.2 Hz), 7.34 (t, 2H, *J* = 7.8 Hz), 6.15 (ddt, 1H, *J* = 5.9 Hz, 10.4 Hz, 16.3 Hz), 6.04 (d, 1H, *J* = 5.0 Hz), 5.51 (dq, 1H, *J* = 1.4 Hz, 17.1 Hz), 5.42 (dq, 1H, *J* = 1.1 Hz, 10.4 Hz), 4.77 (hept., 1H, *J* = 6.2 Hz), 4.71 (dt, 2H, *J* = 1.2 Hz, 5.9 Hz), 4.46 (d, 2H, *J* = 7.0 Hz), 4.38 (t, 1H, *J* = 4.3 Hz), 4.32–4.29 (m, 1H), 4.27 (t, 1H, *J* = 7.1 Hz), 1.61 (s, 9H), 1.42–1.39 (m, 15H), 1.12 (d, 6H, *J* = 6.4 Hz). ^13^C NMR (176 MHz, CDCl_3_, 300 K): δ (ppm) = 168.2, 165.6, 164.3, 162.8, 156.2, 149.4, 144.0, 143.8, 142.3, 141.5, 141.3, 139.1, 137.7, 132.3, 130.8, 130.0, 128.4, 127.9, 127.7, 127.4, 127.2, 125.3, 121.7, 120.2, 120.2, 119.6, 119.2, 115.8, 80.9, 76.9, 75.1, 67.1, 59.3, 47.3, 28.6, 28.4, 23.0. HPLC purity 95.3%.

##### 4-(4-{4-[(2*S*,3*R*)-2-{[4-(4-Cyanobenzamido)phenyl]formamido}-3-hydroxybutanamido]benzamido}-2-hydroxy-3-(propan-2-yloxy)benzamido)benzoic Acid (**42**)

Step 1: The amino acid derivative **128** (99.6 μmol) was deprotected using general procedure 2. The crude product was used without further purification. Step 2: The crude amine (167.8 μmol) was coupled with the carboxylic **10** acid using general procedure 4. Step 3 and 4: The product was obtained by deprotection with general procedures 6 and 7. White to off-white solid, 49 mg (37% over 4 steps). ^1^H NMR (700 MHz, DMSO-*d*_6_, 300 K): δ (ppm) = 12.78 (br s, 1H), 12.31 (br s, 1H), 10.72 (s, 1H), 10.40 (s, 1H), 9.36 (br s, 1H), 8.16–8.13 (m, 3H), 8.05 (d, 2H, *J* = 8.4 Hz), 7.99–7.94 (m, 6H), 7.92 (d, 2H, *J* = 8.8 Hz), 7.85 (d, 2H, *J* = 8.7 Hz), 7.83–7.80 (m, 3H), 7.66 (d, 1H, *J* = 8.0 Hz), 5.08 (d, 1H, *J* = 6.5 Hz), 4.61–4.56 (m, 2H), 4.23–4.18 (m, 1H) 1.26 (dd, 6H, *J* = 1.8 Hz, 6.1 Hz), 1.20 (d, 3H, *J* = 6.3 Hz). ^13^C NMR (176 MHz, DMSO-*d*_6_, 300 K): δ (ppm) = 169.9, 168.4, 166.9, 166.2, 164.1, 142.3, 141.7, 138.7, 136.8, 136.4, 132.5, 130.2, 129.2, 128.6, 128.5, 128.3, 126.1, 122.9, 120.5, 119.6, 118.9, 118.3, 114.1, 112.7, 74.5, 66.8, 60.6, 22.3, 20.4. HPLC purity 97.2%.

##### Methyl (2*R*)-2-{[(*tert*-Butoxy)carbonyl]amino}-3-hydroxypropanoate (**131**)

d-Serine (500 mg, 4.76 mmol) was suspended in MeOH (10 mL) and SOCl_2_ (2.1 mL, 28.5 mmol, 6.00 equiv) was added dropwise at 0 °C. The solution was stirred at ambient temperature for 19 h. The solvent was removed under reduced pressure and coevaporated with Et_2_O (3×). The residue was dissolved in CH_2_Cl_2_ (20 mL) and cooled to 0 °C. Then Et_3_N (1.80 mL, 12.8 mmol, 2.70 equiv) and Boc_2_O (1.10 g, 5.23 mmol, 1.10 equiv) were added carefully and the reaction mixture was allowed to warm to rt. The mixture was stirred for 22 h before the solvent was removed under reduced pressure. The residue was purified by column chromatography (MeOH in CH_2_Cl_2_ = 2, 5, 10%) to furnish the product (945 mg, 4.31 mmol, 91%) as a yellow oil. The analytical data are consistent with those reported in the literature (F. W. Foss, A. H. Snyder, M. D. Davis, M. Rouse, M. D. Okusa, K. R. Lynch, T. L. Macdonald, *Bioorg. Med. Chem.***2007**, 15, 663–677). ^1^H NMR (400 MHz, CDCl_3_) = δ 5.43 (s, 1H), 4.39 (m, 1H), 3.99–3.88 (m, 2H), 3.79 (s, 3H), 2.24 (s, 1H), 1.46 (s, 9H) ppm.

##### *tert*-Butyl *N*-(1,3-Dihydroxy-3-methylbutan-2-yl)carbamate (**132**)

Ester **131** (940 mg, 4.30 mmol) was suspended in Et_2_O (23 mL). MeMgBr (3 M in Et_2_O, 8.60 mL, 25.7 mmol, 6.00 equiv) was added at −78 °C. The emulsion was allowed to warm to rt and stirred at rt for 3 h. The reaction mixture was cooled to 0 °C and a sat. NH_4_Cl solution was added. The aqueous phase was extracted with EtOAc (3×). The combined organic phases were washed with brine, dried over MgSO_4_, filtered and concentrated under reduced pressure to furnish the alcohol (866 mg, 3.95 mmol, 92%) as yellow oil. The analytical data are consistent with those reported in the literature (J. E. Dettwiler, W. D. Lubell, *J. Org. Chem.***2003**, 68, 177–179). ^1^H NMR (400 MHz, CD_3_OD) = δ 3.82–3.79 (dd, *J* = 4.1, 11.2 Hz, 1H), 3.62–3.57 (m, 1H), 3.51–3.48 (m, 1H), 1.45 (s, 9H), 1.23 (s, 3H), 1.15 (s, 3H) ppm.

##### 2-{[(*tert*-Butoxy)carbonyl]amino}-3-hydroxy-3-methylbutanoic Acid (**133**)

Diol **132** (860 mg, 3.92 mmol) was dissolved in MeCN (15 mL). Phosphate buffer (pH 7, 14 mL) and TEMPO (61.3 mg, 0.39 mmol, 0.10 equiv) were added. The solution was warmed to 35 °C and NaClO_2_ (2 M in H_2_O, 4.00 mL, 7.84 mmol, 2.00 equiv) and NaOCl (0.04 M in H_2_O, 2.00 mL, 0.08 mmol, 2 mol %) were added simultaneously over 2 h. The mixture was stirred at 35 °C for 24 h. Citric acid (10%) was added until pH 2. The aqueous phase was extracted with EtOAc (3×) and the combined organic phases were concentrated under reduced pressure. The residue was dissolved in a sat. NaHCO_3_ solution (80 mL). The aqueous phase was washed with EtOAc (2×) and afterward treated with a 1 M H_3_PO_4_ solution (100 mL) until pH 2 was reached. The acidic phase was extracted with EtOAc (3×). The combined organic phases were washed with brine, dried over MgSO_4_, filtered and concentrated under reduced pressure to furnish the carboxylic acid (719 mg, 3.08 mmol, 79%) as a colorless amorphous solid. The analytical data are consistent with those reported in the literature (J. E. Dettwiler, W. D. Lubell, *J. Org. Chem.***2003**, 68, 177–179). ^1^H NMR (400 MHz, CD_3_OD) = δ 4.08 (m, 1H), 1.45 (s, 9H), 1.29 (s, 3H), 1.25 (s, 3H) ppm.

##### *tert*-Butyl 4-(4-{4-[(2*S*)-2-{[(*tert*-Butoxy)carbonyl]amino}-3-hydroxy-3-methylbutanamido]benzamido}-2-(prop-2-en-1-yloxy)-3-(propan-2-yloxy)benzamido)benzoate (**134**)

Amine **8** (200 mg, 0.37 mmol) and carboxylic acid **133** (120 mg, 0.51 mmol, 1.40 equiv) were dissolved in EtOAc (800 μL) and pyridine (89 μL, 1.10 mmol, 3.00 equiv) was added. T3P (50% in EtOAc, 400 μL, 0.66 mmol, 1.80 equiv) was added at 0 °C and the mixture was stirred for 22 h while warming to rt. H_2_O was added and the aqueous phase was extracted with EtOAc (3×). The combined organic phases were washed with a sat. NaHCO_3_ solution, brine, dried over MgSO_4_, filtered and concentrated under reduced pressure. The residue was purified by column chromatography (MeOH in CH_2_Cl_2_ = 0, 1%) to furnish the product (189 mg, 0.25 mmol, 68%) as yellow amorphous solid. [α]_D_^22^ = −2.7° (c 0.2, MeOH). ^1^H NMR (500 MHz, DMSO-*d*_6_) = 10.53 (s, 1H), 10.10 (s, 1H), 9.51 (s, 1H), 7.98–7.96 (d, *J* = 8.8 Hz, 2H), 7.90–7.88 (d, *J* = 8.8 Hz, 2H), 7.84–7.81 (m, 3H), 7.79–7.77 (d, *J* = 8.7 Hz, 2H), 7.41–7.40 (d, *J* = 8.4 Hz, 1H), 6.66–6.64 (d, *J* = 9.0 Hz, 1H), 6.06–5.98 (m, 1H), 5.39–5.35 (dq, *J* = 1.7, 17.1 Hz, 1H), 5.22–5.19 (dq, *J* = 1.7, 10.5 Hz, 1H), 4.87 (s, 1H), 4.61–4.60 (d, *J* = 5.5 Hz, 1H), 4.53–4.46 (sept, *J* = 6.1 Hz, 1H), 4.12–4.08 (m, 1H), 1.55 (s, 9H), 1.40 (s, 9H), 1.26–1.25 (d, *J* = 6.1 Hz, 6H), 1.21 (s, 3H), 1.16 (s, 3H) ppm. ^13^C NMR (126 MHz, DMSO-*d*_6_) = 169.8, 164.6, 164.6, 164.3, 155.4, 149.5, 143.0, 142.5, 142.1, 135.7, 133.7, 130.1, 128.4, 128.3, 127.1, 126.0, 123.6, 118.9, 118.9, 118.8, 117.8, 80.4, 78.4, 76.3, 74.3, 70.8, 63.2, 28.2, 27.9, 27.4, 26.4, 22.3 ppm. HRMS (ESI) calcd for C_41_H_52_N_4_O_10_Na [M + Na]^+^: 783.3581; found: 783.3585.

##### *tert*-Butyl 4-(4-{4-[(2*S*)-2-{[4-(4-Cyanobenzamido)phenyl]formamido}-3-hydroxy-3-methylbutanamido]benzamido}-2-(prop-2-en-1-yloxy)-3-(propan-2-yloxy)benzamido)benzoate (**135**)

Step 1: Carbamate **134** (184 mg, 0.24 mmol) was dissolved in HCl (4 M in 1,4-dioxane, 6.00 mL, 24.2 mmol, 100 equiv) at 0 °C. The mixture was warmed up to rt and stirred for 15 min. The solution was transferred to a stirred suspension of EtOAc (160 mL) and a sat. NaHCO_3_ solution (160 mL). The aqueous phase was extracted with EtOAc and the combined organic phases were washed with brine, dried over MgSO_4_, filtered and concentrated under reduced pressure. The crude product was used in the next step without further purification. Step 2: DIPEA (62 μL, 0.36 mmol, 3.00 equiv) was added dropwise to a stirred solution of HATU (54.1 mg, 0.14 mmol, 1.20 equiv) and carboxylic acid **10** (37.9 mg, 0.14 mmol, 1.20 equiv) in DMF (3.0 mL). The solution was stirred for 5 min and was then transferred to a stirred solution of the amine (78.3 mg, 0.12 mmol) in DMF (1.7 mL). The reaction mixture was stirred at rt for 21 h. The mixture was diluted with EtOAc and washed with a 1 M HCl solution, brine, dried over MgSO_4_, filtered and concentrated under reduced pressure. The crude product was purified by column chromatography (MeOH in CH_2_Cl_2_ = 1, 2, 3%) to furnish the product (50.3 mg, 0.06 mmol, 47%) as colorless amorphous solid. [α]_D_^23^ = +6.0° (c 0.1, MeOH). ^1^H NMR (500 MHz, DMSO-*d*_6_) = 10.71 (s, 1H), 10.53 (s, 1H), 10.28 (s, 1H), 9.52 (s, 1H), 8.14–8.12 (d, *J* = 8.8 Hz, 2H), 8.08–8.06 (d, *J* = 8.9 Hz, 1H), 8.06–8.04 (d, *J* = 8.6 Hz, 2H), 7.99–7.97 (d, *J* = 8.8 Hz, 2H), 7.96–7.95 (d, *J* = 8.9 Hz, 2H), 7.92–7.88 (m, 4H), 7.83–7.80 (m, 5H), 7.41–7.40 (d, *J* = 8.5 Hz, 1H), 6.05–5.97 (m, 1H), 5.39–5.35 (dq, *J* = 1.7, 17.2 Hz, 1H), 5.21–5.18 (dq, *J* = 1.7, 10.5 Hz, 1H), 5.07 (s, 1H), 4.67–4.66 (d, *J* = 8.7 Hz, 1H), 4.61–4.60 (d, *J* = 5.5 Hz, 2H), 4.53–4.46 (sept, *J* = 6.1 Hz, 1H), 1.54 (s, 9H), 1.31 (s, 3H), 1.27 (s, 3H), 1.26–1.25 (d, *J* = 6.1 Hz, 6H) ppm. ^13^C NMR (126 MHz, DMSO-*d*_6_) = 169.4, 166.1, 164.6, 164.6, 164.5, 164.3, 149.6, 143.0, 142.6, 142.1, 141.7, 138.7, 135.7, 133.7, 132.6, 130.1, 129.3, 128.7, 128.5, 128.4, 127.1, 126.1, 123.7, 119.6, 119.0, 118.9, 118.3, 117.8, 114.1, 80.4, 76.3, 74.3, 70.9, 62.3, 27.9, 27.4, 27.2, 22.4 ppm. HRMS (ESI) calcd for C_51_H_52_N_6_O_10_Na [M + Na]^+^: 931.3643; found: 931.3638.

##### *tert*-Butyl 4-(4-{4-[(2*S*)-2-{[4-(4-Cyanobenzamido)phenyl]formamido}-3-hydroxy-3-methylbutanamido]benzamido}-2-hydroxy-3-(propan-2-yloxy)benzamido)benzoate (**136**)

Allyl ether **135** (47.4 mg, 0.05 mmol) was dissolved in THF (2.6 mL). Aniline (16 μL, 0.17 mmol, 3.30 equiv) and Pd(PPh_3_)_4_ (6.0 mg, 5 μmol, 0.10 equiv) were added subsequently and the resulting mixture was stirred at rt for 2 h. The mixture was concentrated under reduced pressure. The crude product was purified by column chromatography (MeOH in CH_2_Cl_2_ = 1, 2, 3%) to furnish the product (37.7 mg, 0.04 mmol, 83%) as yellow amorphous solid. [α]_D_^23^ = +4.9° (c 0.1, MeOH). ^1^H NMR (500 MHz, DMSO-*d*_6_) = 12.28 (s, 1H), 10.71 (s, 1H), 10.61 (s, 1H), 10.30 (s, 1H), 9.40 (s, 1H), 8.14–8.13 (d, *J* = 8.6 Hz, 2H), 8.08–8.04 (m, 3H), 7.97–7.90 (m, 8H), 7.86–7.81 (m, 5H), 7.71–7.69 (d, *J* = 8.9 Hz, 1H), 5.05 (s, 1H), 4.69–4.67 (d, *J* = 8.7 Hz, 1H), 4.58–4.51 (sept, *J* = 6.1 Hz, 1H), 1.55 (s, 9H), 1.31 (s, 3H), 1.27–1.26 (s, 9H) ppm. ^13^C NMR (126 MHz, DMSO-*d*_6_) = 169.4, 168.5, 166.1, 164.5, 164.5, 164.2, 154.1, 142.2, 142.0, 141.7, 138.7, 137.0, 136.3, 132.5, 129.9, 129.3, 128.6, 128.4, 128.4, 126.8, 122.8, 120.7, 119.6, 118.9, 118.3, 114.1, 112.4, 80.5, 74.9, 70.9, 62.3, 27.8, 27.3, 27.1, 22.3 ppm. HRMS (ESI) calcd for C_48_H_47_N_6_O_10_Na [M – H]^−^: 867.3354; found: 867.3352.

##### 4-(4-{4-[(2*S*)-2-{[4-(4-Cyanobenzamido)phenyl]formamido}-3-hydroxy-3-methylbutanamido]benzamido}-2-hydroxy-3-(propan-2-yloxy)benzamido)benzoic Acid (**43**)

Ester **136** (35.8 mg, 0.04 mmol) was dissolved in precooled TFA (2 mL) at 0 °C with stirring. The solution was warmed up to rt over 30 min. Et_2_O was added at 0 °C. The precipitate was filtered off, washed with an excess of Et_2_O and dried *in vacuo* to furnish the product (13.0 mg, 0.02 mmol, 39%) as beige amorphous solid. [α]_D_^23^ = +9.5° (c 0.1, MeOH). ^1^H NMR (500 MHz, DMSO-*d*_6_) = 12.82 (s, 1H), 12.29 (s, 1H), 10.71 (s, 1H), 10.60 (s, 1H), 10.30 (s, 1H), 9.40 (s, 1H), 8.14–8.13 (d, *J* = 8.5 Hz, 2H), 8.08–8.04 (m, 3H), 7.98–7.95 (m, 5H), 7.92–7.90 (d, *J* = 8.9 Hz, 2H), 7.86–7.81 (m, 5H), 7.72–7.70 (d, *J* = 9.0 Hz, 1H), 5.05 (s, 1H), 4.69–4.67 (d, *J* = 8.7 Hz, 1H), 4.58–4.51 (sept, *J* = 6.2 Hz, 1H), 1.31 (s, 3H), 1.27–1.26 (s, 9H) ppm. ^13^C NMR (126 MHz, DMSO-*d*_6_) = 169.4, 168.5, 166.9, 166.1, 164.5, 164.2, 154.1, 142.2, 142.0, 141.7, 138.7, 137.0, 136.3, 132.5, 130.2, 129.3, 128.6, 128.4, 128.4, 126.3, 122.8, 120.7, 119.6, 118.9, 118.3, 114.1, 112.4, 112.2, 74.9, 70.9, 62.3, 27.3, 27.1, 22.3 ppm. HRMS (ESI) calcd for C_44_H_39_N_6_O_10_ [M – H]^−^: 811.2728; found: 811.2729.

##### (2*S*,3*S*)-2-{[(*tert*-Butoxy)carbonyl]amino}-3-hydroxybutanoic Acid (**138**)

A mixture of NaHCO_3_ (543 mg, 6.46 mmol, 1.50 equiv) and Boc_2_O (1.43 g, 6.55 mmol, 1.60 equiv) in MeOH (8.5 mL) was added to a solution of l-allothreonine (**137**) (500 mg, 4.20 mmol) in H_2_O (8.5 mL). The mixture was stirred at rt for 18 h and afterward acidified with a 0.5 M HCl solution. The aqueous phase was extracted with EtOAc (3×). The combined organic phases were dried over MgSO_4_, filtered and concentrated under reduced pressure to furnish the carbamate (963 mg), which was used in the next step without further purification.

##### Methyl (2*S*,3*S*)-2-{[(*tert*-Butoxy)carbonyl]amino}-3-methoxybutanoate (**139**)

Amino acid derivative **138** (920 mg, 4.20 mmol) was dissolved in MeCN (4.6 mL) and Ag_2_O (4.86 g, 21.0 mmol, 5.00 equiv) was added. MeI (4.20 mL, 67.2 mmol, 16.00 equiv) was added at 0 °C and the resulting mixture was stirred at rt for 48 h. The mixture was filtered through Celite and the plug was washed with EtOAc. The filtrate was concentrated under reduced pressure and the residue was purified by column chromatography (dry load, PE/EtOAc = 8:1) to furnish product **295** (486 mg, 1.97 mmol, 47% over two steps) as colorless oil. The analytical data are consistent with those reported in the literature (G. B. Martinez, A. Griffin, P. Charifson, K. Reddy, K. M. Kahlig, B. Marron, WO2020227101 (A1), **2020**). ^1^H NMR (400 MHz, CDCl_3_) = δ 5.28–5.27 (d, *J* = 7.2 Hz, 1H), 4.44–4.41 (dd, *J* = 3.6, 4.9 Hz, 1H), 3.76 (s, 3H), 3.64–3.62 (m, 1H), 3.36 (s, 3H), 1.44 (s, 9H), 1.21–1.19 (d, *J* = 6.4 Hz, 1H) ppm.

##### (2*S*,3*S*)-2-{[(*tert*-Butoxy)carbonyl]amino}-3-methoxybutanoic Acid (**140**)

Ester **139** (479 mg, 1.94 mmol) was dissolved in THF (3.6 mL) and H_2_O (1.8 mL). LiOH·H_2_O (488 mg, 11.6 mmol, 6.00 equiv) was added and the mixture was stirred at rt for 3 h. THF was removed under reduced pressure and the residue was acidified with a 2 M HCl solution. The aqueous phase was extracted with EtOAc (3×). The combined organic phases were dried over Na_2_SO_4_, filtered and concentrated under reduced pressure to furnish the acid (490 mg, quant.) as yellow oil, which was used in the next step without further purification. The analytical data are consistent with those reported in the literature (G. B. Martinez, A. Griffin, P. Charifson, K. Reddy, K. M. Kahlig, B. Marron, WO2020227101 (A1), **2020**). ^1^H NMR (400 MHz, CDCl_3_) = δ 8.34 (bs, 1H), 5.31–5.29 (d, *J* = 7.5 Hz, 1H), 4.44–4.43 (m, 1H), 3.69 (m, 1H), 3.38 (s, 3H), 1.45 (s, 9H), 1.26–1.24 (d, *J* = 6.3 Hz, 3H) ppm.

##### *tert*-Butyl 4-(4-{4-[(2*S*,3*S*)-2-{[(*tert*-Butoxy)carbonyl]amino}-3-methoxybutanamido]benzamido}-2-(prop-2-en-1-yloxy)-3-(propan-2-yloxy)benzamido)benzoate (**141**)

Amine **8** (100 mg, 0.18 mmol), carboxylic acid **140** (72.7 mg, 0.31 mmol, 1.70 equiv) and EEDQ (72.5 mg, 0.29 mmol, 1.60 equiv) were dissolved in precooled CHCl_3_ (1 mL) at 0 °C. The mixture was stirred at 18 h while warming to rt. The mixture was concentrated under reduced pressure and the residue was purified by column chromatography (1. dry load, 20% Et_2_O in CH_2_Cl_2_, 2. dry load, 1% MeOH in CH_2_Cl_2_) to furnish the product (74.3 mg, 0.10 mmol, 53%) as yellow amorphous solid, which contained small impurities. The compound was used in the next step without further purification. ^1^H NMR (500 MHz, DMSO-*d*_6_) = δ 10.53 (s, 1H), 10.33–10.25 (m, 1H), 9.51–9.49 (m, 1H), 7.98–7.94 (m, 2H), 7.90–7.88 (m, 2H), 7.83–7.79 (m, 5H), 7.42–7.40 (d, *J* = 8.4 Hz, 1H), 7.08–7.07 (d, *J* = 8.6 Hz, 1H), 6.07–5.98 (m, 1H), 5.39–5.36 (dd, *J* = 1.6, 17.2 Hz, 1H), 5.21–5.19 (dd, *J* = 1.5, 10.5 Hz, 1H), 4.61–4.60 (d, *J* = 5.4 Hz, 1H), 4.54–4.46 (sept, *J* = 6.1 Hz, 1H), 4.23–4.20 (t, *J* = 8.1 Hz, 1H), 3.59–3.57 (m, 1H), 3.23 (s, 3H), 1.55 (s, 9H), 1.39 (s, 9H), 1.27–1.26 (d, *J* = 6.1 Hz, 6H), 1.13–1.12 (d, *J* = 5.9 Hz, 3H) ppm. HRMS (ESI) calcd for C_41_H_52_N_4_O_10_Na [M + Na]^+^: 783.3581; found: 783.3583.

##### *tert*-Butyl 4-(4-{4-[(2*S*,3*S*)-2-{[4-(4-Cyanobenzamido)phenyl]formamido}-3-methoxybutanamido]benzamido}-2-(prop-2-en-1-yloxy)-3-(propan-2-yloxy)benzamido)benzoate (**142**)

Step 1: Carbamate **141** (70.9 mg, 0.09 mmol) was dissolved in HCl (4 M in 1,4-dioxane, 2.3 mL, 9.32 mmol, 100 equiv) at 0 °C. The mixture was warmed up to rt and stirred for 15 min. The solution was transferred to a stirred suspension of EtOAc (60 mL) and a sat. NaHCO_3_ solution (60 mL). The aqueous phase was extracted with EtOAc and the combined organic phases were washed with brine, dried over MgSO_4_, filtered and concentrated under reduced pressure. The crude product was used in the next step without further purification. Step 2: DIPEA (49 μL, 0.28 mmol, 3.00 equiv) was added dropwise to a stirred solution of HATU (42.5 mg, 0.11 mmol, 1.20 equiv) and carboxylic acid **10** (29.8 mg, 0.11 mmol, 1.20 equiv) in DMF (2.3 mL). The solution was stirred for 5 min and was then transferred to a stirred solution of the amine (61.6 mg, 0.09 mmol) in DMF (1.3 mL). The reaction mixture was stirred at rt for 16 h. The mixture was diluted with EtOAc and washed with a 1 M HCl solution, brine, dried over MgSO_4_, filtered and concentrated under reduced pressure. The crude product was purified by column chromatography (dry load, 2% MeOH in CH_2_Cl_2_) to furnish the product (31.4 mg, 0.04 mmol, 37%) as colorless amorphous solid. [α]_D_^23^ = +3.3° (c 0.1, MeOH). ^1^H NMR (600 MHz, DMSO-*d*_6_) = 10.69 (s, 1H), 10.52 (s, 1H), 10.50 (s, 1H), 9.51 (s, 1H), 8.53–8.52 (d, *J* = 8.3 Hz, 1H), 8.13–8.12 (d, *J* = 8.4 Hz, 2H), 8.05–8.04 (d, *J* = 8.4 Hz, 2H), 7.99–7.95 (m, 4H), 7.90–7.88 (m, 4H), 7.84–7.82 (m, 4H), 7.41–7.40 (d, *J* = 8.4 Hz, 1H), 6.05–5.98 (m, 1H), 5.39–5.36 (dd, *J* = 1.6, 17.2 Hz, 1H), 5.21–5.19 (dd, *J* = 1.3, 10.5 Hz, 1H), 4.61–4.60 (d, *J* = 5.3 Hz, 2H), 4.74–4.72 (t, *J* = 8.3 Hz, 1H), 4.53–4.48 (sept, *J* = 6.1 Hz, 1H), 3.85–3.81 (m, 1H), 3.29 (s, 3H), 1.55 (s, 9H), 1.27–1.25 (d, *J* = 6.1 Hz, 6H), 1.23–1.22 (d, *J* = 6.0 Hz, 3H) ppm. ^13^C NMR (151 MHz, DMSO-*d*_6_) = 170.1, 166.0, 164.6, 164.5, 164.4, 164.3, 149.5, 143.0, 142.5, 142.3, 141.6, 138.7, 135.6, 133.6, 132.5, 130.1, 129.1, 128.6, 128.5, 128.4, 128.4, 127.1, 126.0, 123.6, 119.5, 118.9, 118.8, 118.8, 118.3, 117.8, 114.0, 80.3, 76.3, 76.2, 74.3, 58.5, 56.5, 27.9, 22.3, 16.2 ppm. HRMS (ESI) calcd for C_51_H_52_N_6_O_10_Na [M + Na]^+^: 931.3643; found: 931.3643.

##### *tert*-Butyl 4-(4-{4-[(2*S*,3*S*)-2-{[4-(4-Cyanobenzamido)phenyl]formamido}-3-methoxybutanamido]benzamido}-2-hydroxy-3-(propan-2-yloxy)benzamido)benzoate (**143**)

Allyl ether **142** (29.0 mg, 0.03 mmol) was dissolved in THF (1.6 mL). Aniline (10 μL, 0.11 mmol, 3.30 equiv) and Pd(PPh_3_)_4_ (3.7 mg, 0.003 mmol, 0.10 equiv) were added subsequently and the resulting mixture was stirred at rt for 90 min. The mixture was concentrated under reduced pressure. The crude product was purified by column chromatography (dry load, MeOH in CH_2_Cl_2_ = 2, 3%) to furnish product **149** (19.7 mg, 0.02 mmol, 71%) as beige amorphous solid. [α]_D_^25^ = +2.3° (c 0.1, MeOH). ^1^H NMR (500 MHz, DMSO-*d*_6_) = 12.29 (s, 1H), 10.69 (s, 1H), 10.61 (bs, 1H), 10.52 (s, 1H), 9.39 (s, 1H), 8.54–8.52 (d, *J* = 8.3 Hz, 1H), 8.14–8.12 (d, *J* = 8.6 Hz, 2H), 8.05–8.04 (d, *J* = 8.6 Hz, 2H), 7.96–7.92 (m, 6H), 7.89–7.84 (m, 7H), 7.72–7.70 (d, *J* = 8.9 Hz, 1H), 4.74–4.71 (t, *J* = 8.3 Hz, 1H), 4.58–4.51 (sept, *J* = 6.1 Hz, 1H), 3.85–3.80 (m, 1H), 3.29 (s, 3H), 1.55 (s, 9H), 1.27–1.26 (d, *J* = 6.1 Hz, 6H), 1.24–1.22 (d, *J* = 6.0 Hz, 3H) ppm. ^13^C NMR (126 MHz, DMSO-*d*_6_) = 170.1, 168.5, 166.0, 164.5, 164.4, 164.2, 142.4, 142.0, 141.6, 138.7, 137.0, 136.3, 132.5, 129.9, 129.1, 128.6, 128.5, 128.4, 126.8, 122.8, 120.7, 119.5, 118.8, 118.3, 114.0, 112.4, 112.1, 80.5, 76.3, 74.8, 58.5, 56.5, 27.8, 22.3, 16.2 ppm. In the ^13^C NMR spectrum is one signal around 154 ppm missing due to the minimal analytical amount. HRMS (ESI) calcd for C_48_H_48_N_6_O_10_Na [M + Na]^+^: 891.3330; found: 891.3321.

##### 4-(4-{4-[(2*S*,3*S*)-2-{[4-(4-Cyanobenzamido)phenyl]formamido}-3-methoxybutanamido]benzamido}-2-hydroxy-3-(propan-2-yloxy)benzamido)benzoic Acid (44)

*tert*-Butyl ester **143** (17.6 mg, 0.02 mmol) was dissolved in precooled TFA (1.2 mL) at 0 °C with stirring. The solution was warmed up to rt over 30 min. Et_2_O was added at 0 °C. The precipitate was filtered off, washed with an excess of Et_2_O and dried *in vacuo* to furnish the acid (10.1 mg, 0.01 mmol, 61%) as gray amorphous solid. [α]_D_^25^ = +4.2° (c 0.1, MeOH). ^1^H NMR (500 MHz, DMSO-*d*_6_) = 12.82 (bs, 1H), 12.30 (s, 1H), 10.69 (s, 1H), 10.60 (s, 1H), 10.52 (s, 1H), 9.40 (s, 1H), 8.54–8.52 (d, *J* = 8.4 Hz, 1H), 8.14–8.12 (d, *J* = 8.5 Hz, 2H), 8.05–8.04 (d, *J* = 8.5 Hz, 2H), 7.98–7.95 (m, 6H), 7.89–7.84 (m, 7H), 7.72–7.70 (d, *J* = 8.9 Hz, 1H), 4.74–4.71 (t, *J* = 8.3 Hz, 1H), 4.58–4.51 (sept, *J* = 6.1 Hz, 1H), 3.85–3.80 (m, 1H), 3.29 (s, 3H), 1.27–1.26 (d, *J* = 6.1 Hz, 6H), 1.24–1.22 (d, *J* = 6.1 Hz, 3H) ppm. ^13^C NMR (126 MHz, DMSO-*d*_6_) = 170.1, 168.5, 166.9, 166.0, 164.4, 164.2, 154.1, 142.4, 142.0, 141.6, 138.7, 137.1, 136.3, 132.5, 130.2, 129.1, 128.6, 128.5, 128.4, 128.4, 126.3, 122.8, 120.7, 119.5, 118.8, 118.3, 114.0, 112.4, 112.2, 76.3, 74.9, 58.5, 56.5, 22.3, 16.2 ppm. HRMS (ESI) calcd for C_44_H_39_N_6_O_10_ [M – H]^−^: 811.2728; found: 811.2733.

##### (2*S*)-2-{[(*tert*-Butoxy)carbonyl]amino}-3-methoxy-3-methylbutanoic Acid (**144**)

Tertiary alcohol **133** (300 mg, 1.29 mmol) in THF (2.0 mL) was added to NaH (60% in mineral oil, 154 mg, 3.86 mmol, 3.00 equiv) in THF (2.4 mL) at 0 °C. The mixture was stirred at rt for 1 h. MeI (96 μL, 1.54 mmol, 1.20 equiv) was added and the mixture was stirred at rt for 19 h. H_2_O was added and the aqueous phase was extracted with Et_2_O (3×). The aqueous phase was acidified with 6 M HCl until pH 2 and extracted with EtOAc (4×). The combined organic phases were washed with brine, dried over MgSO_4_, filtered and concentrated under reduced pressure. The crude product was purified by column chromatography (MeOH in CH_2_Cl_2_ = 0, 2%) to furnish product (162 mg, 0.65 mmol, 51%) as yellow gum. [α]_D_^21^ = +5.8° (c 1.3, MeOH). ^1^H NMR (500 MHz, CD_3_OD) = δ 4.20 (s, 1H), 3.23 (s, 3H), 1.45 (s, 9H), 1.28 (s, 3H), 1.26 (s, 3H) ppm. ^13^C NMR (126 MHz, CD_3_OD) = δ 174.1, 157.9, 80.8, 77.1, 61.7, 50.0, 28.7, 22.7 (d, *J* = 8.0 Hz) ppm. HRMS (ESI^+^) calcd for C_11_H_20_NO_5_ [M – H]^−^: 246.1341; found: 246.1351.

##### *tert*-Butyl 4-(4-{4-[(2*S*)-2-{[(*tert*-Butoxy)carbonyl]amino}-3-methoxy-3-methylbutanamido]benzamido}-2-(prop-2-en-1-yloxy)-3-(propan-2-yloxy)benzamido)benzoate (**145**)

Amine **8** (100 mg, 0.18 mmol) and carboxylic acid **144** (63.5 mg, 0.26 mmol, 1.40 equiv) were dissolved in EtOAc (400 μL) and pyridine (44 μL, 0.55 mmol, 3.00 equiv) was added. T3P (50% in EtOAc, 200 μL, 0.33 mmol, 1.80 equiv) was added at 0 °C and the mixture was stirred at 0 °C for 3 h. H_2_O was added and the aqueous phase was extracted with EtOAc (3×). The combined organic phases were washed with a sat. NaHCO_3_ solution, brine, dried over MgSO_4_, filtered and concentrated under reduced pressure. The residue was purified by column chromatography (dry load, PE/EtOAc = 2:1) to furnish the product (70.2 mg, 0.09 mmol, 49%) as brown amorphous solid. [α]_D_^22^ = −15.2° (c 0.1, MeOH). ^1^H NMR (500 MHz, DMSO-*d*_6_) = 10.53 (s, 1H), 10.16 (s, 1H), 9.51 (s, 1H), 7.98–7.97 (d, *J* = 8.7 Hz, 2H), 7.90–7.88 (d, *J* = 8.7 Hz, 2H), 7.84–7.81 (m, 3H), 7.79–7.78 (d, *J* = 8.7 Hz, 2H), 7.41–7.40 (d, *J* = 8.4 Hz, 1H), 6.76–6.74 (d, *J* = 8.7 Hz, 1H), 6.05–5.99 (m, 1H), 5.39–5.38 (dd, *J* = 1.6, 17.2 Hz, 1H), 5.21–5.19 (dd, *J* = 1.4, 10.5 Hz, 1H), 4.61–4.60 (d, *J* = 5.4 Hz, 1H), 4.53–4.47 (sept, *J* = 6.2 Hz, 1H), 4.31–4.30 (d, *J* = 9.0 Hz, 1H), 3.17 (s, 3H), 1.55 (s, 9H), 1.40 (s, 9H), 1.26–1.25 (d, *J* = 6.1 Hz, 6H), 1.19 (s, 6H) ppm. ^13^C NMR (126 MHz, DMSO-*d*_6_) = 169.4, 164.6, 164.5, 164.3, 155.4, 149.5, 143.0, 142.5, 142.0, 135.6, 133.6, 130.1, 128.5, 128.4, 127.1, 126.0, 123.6, 118.9, 118.8, 118.8, 117.8, 80.5, 78.5, 76.2, 76.0, 74.3, 59.7, 49.2, 28.1, 27.9, 22.3, 22.1 ppm. HRMS (ESI) calcd for C_42_H_54_N_4_O_10_Na [M + Na]^+^: 797.3738; found: 797.3726.

##### *tert*-Butyl 4-(4-{4-[(2*S*)-2-{[4-(4-Cyanobenzamido)phenyl]formamido}-3-methoxy-3-methylbutanamido]benzamido}-2-(prop-2-en-1-yloxy)-3-(propan-2-yloxy)benzamido)benzoate (**146**)

Step 1: Carbamate **145** (65.0 mg, 0.08 mmol) was dissolved in HCl (4 M in 1,4-dioxane, 2.10 mL, 9.32 mmol, 100 equiv) at 0 °C. The mixture was warmed up to rt and stirred for 15 min. The solution was transferred to a stirred suspension of EtOAc (60 mL) and a sat. NaHCO_3_ solution (60 mL). The aqueous phase was extracted with EtOAc and the combined organic phases were washed with brine, dried over MgSO_4_, filtered and concentrated under reduced pressure. The crude product was used in the next step without further purification. Step 2: DIPEA (44 μL, 0.25 mmol, 3.00 equiv) was added dropwise to a stirred solution of HATU (38.3 mg, 0.10 mmol, 1.20 equiv) and carboxylic acid **10** (26.8 mg, 0.10 mmol, 1.20 equiv) in DMF (2.1 mL). The solution was stirred for 5 min and was then transferred to a stirred solution of the amine (56.6 mg, 0.08 mmol) in DMF (1.2 mL). The reaction mixture was stirred at rt for 21 h. The mixture was diluted with EtOAc and washed with a 1 M HCl solution, brine, dried over MgSO_4_, filtered and concentrated under reduced pressure. The crude product was purified by column chromatography (MeOH in CH_2_Cl_2_ = 1, 2, 3%) to furnish the product (40.3 mg, 0.04 mmol, 52%) as colorless amorphous solid. [α]_D_^22^ = +5.4° (c 0.1, MeOH). ^1^H NMR (500 MHz, DMSO-*d*_6_) = 10.71 (s, 1H), 10.53 (s, 1H), 10.33 (s, 1H), 9.52 (bs, 1H), 8.14–8.12 (d, *J* = 8.6 Hz, 2H), 8.07–8.04 (m, 3H), 7.99–7.97 (d, *J* = 8.8 Hz, 2H), 7.96–7.94 (d, *J* = 8.9 Hz, 2H), 7.91–7.88 (m, 3H), 7.84–7.80 (m, 5H), 7.41–7.40 (d, *J* = 8.5 Hz, 1H), 6.06–5.98 (m, 1H), 5.39–5.35 (dq, *J* = 1.7, 17.2 Hz, 1H), 5.22–5.19 (dq, *J* = 1.7, 10.5 Hz, 1H), 4.89–4.87 (d, *J* = 8.7 Hz, 1H), 4.61–4.60 (d, *J* = 5.5 Hz, 1H), 4.53–4.46 (sept, *J* = 6.1 Hz, 1H), 3.23 (s, 3H), 1.55 (s, 9H), 1.31 (s, 6H), 1.26–1.25 (d, *J* = 6.1 Hz, 6H) ppm. ^13^C NMR (126 MHz, DMSO-*d*_6_) = 169.1, 166.2, 164.6, 164.5, 164.4, 164.3, 149.5, 143.0, 142.5, 142.0, 141.7, 138.7, 135.6, 133.7, 132.5, 130.4, 130.1, 129.2, 128.6, 128.6, 128.5, 127.1, 126.0, 123.6, 119.5, 119.0, 118.9, 118.8, 118.3, 117.8, 114.0, 80.5, 76.2, 76.1, 74.3, 60.4, 49.3, 27.9, 22.3, 21.7 ppm. HRMS (ESI) calcd for C_52_H_54_N_6_O_10_Na [M + Na]^+^: 945.3799; found: 945.3806.

##### *tert*-Butyl 4-(4-{4-[(2*S*)-2-{[4-(4-Cyanobenzamido)phenyl]formamido}-3-methoxy-3-methylbutanamido]benzamido}-2-hydroxy-3-(propan-2-yloxy)benzamido)benzoate (**147**)

Allyl ether **146** (38.5 mg, 0.04 mmol) was dissolved in THF (2 mL). Aniline (13 μL, 0.14 mmol, 3.30 equiv) and Pd(PPh_3_)_4_ (4.8 mg, 4 μmol, 0.10 equiv) were added subsequently and the resulting mixture was stirred at rt for 2 h. The mixture was concentrated under reduced pressure. The crude product was purified by column chromatography (MeOH in CH_2_Cl_2_ = 1, 2, 3%) to furnish the product (27.0 mg, 0.03 mmol, 73%) as colorless amorphous solid. [α]_D_^22^ = −3.8° (c 0.1, MeOH).

^1^H NMR (600 MHz, DMSO-*d*_6_) = 12.29 (s, 1H), 10.70 (s, 1H), 10.60 (bs, 1H), 10.35 (s, 1H), 9.39 (bs, 1H), 8.14–8.13 (d, *J* = 8.6 Hz, 2H), 8.07–8.04 (m, 3H), 7.96–7.89 (m, 8H), 7.86–7.81 (m, 5H), 4.89–4.88 (d, *J* = 8.9 Hz, 1H), 4.55 (bs, 1H), 3.23 (s, 3H), 1.55 (s, 9H), 1.31 (s, 6H), 1.27–1.26 (d, *J* = 6.1 Hz, 6H) ppm. ^13^C NMR (151 MHz, DMSO-*d*_6_) = 169.1, 168.4, 166.2, 164.6, 164.4, 164.1, 142.1, 141.7, 138.7, 136.3, 132.5, 131.5, 131.4, 129.9, 129.2, 128.8, 128.7, 128.6, 128.5, 128.4, 122.9, 120.6, 119.5, 118.9, 118.3, 114.0, 80.5, 76.1, 74.9, 60.4, 49.3, 27.8, 22.3, 21.7 ppm. HRMS (ESI) calcd for C_49_H_50_N_6_O_10_Na [M + Na]^+^: 905.3486; found: 905.3470.

##### 4-(4-{4-[(2*S*)-2-{[4-(4-Cyanobenzamido)phenyl]formamido}-3-methoxy-3-methylbutanamido]benzamido}-2-hydroxy-3-(propan-2-yloxy)benzamido)benzoic Acid (**45**)

Ester **147** (25.0 mg, 0.03 mmol) was dissolved in precooled TFA (1.5 mL) at 0 °C with stirring. The solution was warmed up to rt over 30 min. Et_2_O was added at 0 °C. The precipitate was filtered off, washed with an excess of Et_2_O and dried *in vacuo* to furnish the product (5.3 mg, 0.01 mmol, 23%) as beige amorphous solid. [α]_D_^22^ = +0.8° (c 0.1, MeOH). ^1^H NMR (500 MHz, DMSO-*d*_6_) = 12.82 (s, 1H), 12.29 (s, 1H), 10.71 (s, 1H), 10.60 (s, 1H), 10.35 (s, 1H), 9.40 (s, 1H), 8.14–8.12 (d, *J* = 8.5 Hz, 2H), 8.07–8.04 (m, 3H), 7.98–7.94 (m, 6H), 7.91–7.89 (d, *J* = 8.8 Hz, 2H), 7.86–7.81 (m, 5H), 7.71–7.70 (d, *J* = 8.9 Hz, 1H), 4.89–4.87 (d, *J* = 8.9 Hz, 1H), 4.58–4.51 (sept, *J* = 6.1 Hz, 1H), 3.23 (s, 3H), 1.31 (s, 6H), 1.27–1.26 (d, *J* = 6.1 Hz, 6H) ppm. ^13^C NMR (126 MHz, DMSO-*d*_6_) = 169.1, 168.5, 166.9, 166.2, 164.4, 164.2, 154.1, 142.1, 142.0, 141.7, 138.7, 137.0, 136.3, 132.5, 130.2, 129.2, 128.6, 128.6, 128.5, 126.3, 122.8, 120.7, 119.5, 118.9, 118.3, 114.0, 112.4, 112.2, 76.1, 74.9, 60.4, 49.3, 22.3, 21.7 ppm. HRMS (ESI) calcd for C_45_H_41_N_6_O_10_ [M – H]^−^: 825.2884; found: 825.2889.

### Determination of Thermodynamic (Equilibrium) Solubility

The selected compound is suspended in phosphate buffer (50 mM, pH 7.4) at a target concentration of 1 mg/mL. After overnight stirring at room temperature, protected from light, suspensions are filtered through a 0.45 μm PTFE membrane. An aliquot of the resulting supernatant is quantified using UPLC UV method against a reference solution obtained by preparation of a DMSO stock solution. The solubility value is expressed as μL.

### Determination of Log *D*_7.4_ Values

Log *D* values at pH 7.4 were determined by a standardized HPLC method as essentially derived from Genieser et al.^25^ The calculation of the log *D* value for measured compounds is performed by comparison of the retention times with standard compounds of known distribution coefficients between 1-octanol and water at pH 7.4.

### Determination of Minimal Inhibitory Concentrations (MIC)

#### Method 1

All microorganisms were handled according to standard procedures. The microorganisms were obtained from the German Collection of Microorganisms and Cell Cultures (*Deutsche Sammlung für Mikroorganismen and Zellkulturen*, DSMZ), the American Type Culture Collection (ATCC) or were part of the internal strain collection. The cystobactamids were prepared as DMSO stock solutions with a concentration of 5 mg/mL. The Minimum inhibitory concentrations (MIC) were determined by standard procedures.^8^ Single colonies of the bacterial strains were suspended in Müller-Hinton broth and grown overnight at appropriate temperature. On the following day, the bacterial count was adjusted by dilution to achieve a final inoculum of approximately 5 × 10^5^ – 1 × 10^6^ CFU/mL in cation-adjusted Müller-Hinton broth. Serial dilutions of the tested cystobactamids and reference antibiotics (0.03–64 μg/mL) were prepared in sterile 96-well plates and the bacterial suspension was added. The microorganisms were grown overnight at appropriate temperature under shaking conditions. The growth inhibition was assessed visually. The determined MIC value represented the lowest concentration of antibiotic at which there was no visible growth. This determination method was applied for the testing of the compounds in Table 3.

#### Method 2

All microorganisms were handled according to standard procedures. The microorganisms were obtained from the German Collection of Microorganisms and Cell Cultures (*Deutsche Sammlung für Mikroorganismen and Zellkulturen*, DSMZ), the American Type Culture Collection (ATCC), Evotec or were part of the internal strain collection. The cystobactamids were prepared as DMSO stock solutions with a concentration of 5 mg/mL. The Minimum inhibitory concentrations (MIC) were determined by standard procedures.^26^ The bacterial cultures were streaked out on Müller-Hinton or blood agar, as appropriate, and grown overnight at appropriate temperature. The following day, three to four isolated colonies were collected with a sterile cotton swab and resuspended in saline solution to obtain turbidity equal to McFarland Standard 0.5. Serial dilutions of the tested cystobactamids and reference antibiotics (0.03–64 μg/mL) were prepared in cation-adjusted Müller-Hinton broth or TSB, as appropriate, in sterile 96-well plates and the bacterial suspension was added. The growth inhibition was assessed after 16–20 h or after satisfactory growth of the controls was observed at appropriate temperature under static conditions. The determined MIC value represented the lowest concentration of antibiotic at which there was no visible growth. This determination method was applied for the testing of the compounds in Tables 1 and 2 as well as Tables S1–S4.

### Determination of the Time-Kill Curve

Test articles were prepared in sterile dimethyl sulfoxide (DMSO) and used at final concentrations of 1, 2, 4, or 8× the minimum inhibitory concentration (MIC), with a final DMSO concentration of 1% (v/v). Killing kinetics assays were performed in triplicate on separate days, using separate preparations of growth medium and bacterial inocula. Starter cultures of each bacterial strain were inoculated by suspending a single colony from a Mueller-Hinton agar plate in 10 mL cation-adjusted Mueller-Hinton broth (caMHB) and incubating at 37 °C with shaking at 300 rpm. Following 18 h’ incubation, starter cultures were diluted into fresh prewarmed caMHB containing the appropriate concentration of CN-CC-861 (**13**) (or 1% DMSO for the vehicle-treated control) to achieve a target bacterial density of approximately 5 × 10^5^ CFU/mL at the start of the assay. A sample (200 μL) was removed immediately from each tube for quantification of the total viable bacterial count, which was achieved by serial 10-fold dilution in sterile phosphate-buffered saline (PBS), followed by plating onto cystine–lactose–electrolyte-deficient (CLED) agar. Subsequently, at 1-, 2-, 4-, 6-, 8- and 24 h postinoculation, additional samples were taken for bacterial quantification as described above. Agar plates were incubated at 37 °C in air overnight prior to enumeration of colonies for each condition. The limit of detection (LoD) for the assay was 50 CFU/mL.

### DNA Supercoiling Assay

The supercoiling assay for the determination if IC_50_ values on *E. coli* gyrase was carried out in two steps. The test compounds were diluted to a 0.75 mM for the assay. The method is described in the following.

#### DNA Relaxation

In two separate reactions 25 μL (1 μg/μL) purified circular pUC19 plasmid was mixed with 13.5 μL H_2_O, 0.5 μL Topoisomerase I (6.5 U) and 50 μL topoisomerase buffer (250 mM Tris [pH 7.5], 250 mM KCl, 50 mM MgCl_2_, 2.5 mM DTT, 0.5 mM EDTA and 150 μg/mL BSA). The mixture was incubated for 90 min at 37 °C. Both reactions were combined, and the relaxed plasmid DNA was purified by spin-columns according to the vendor’s manual. The concentration was determined by measurement of the optical density at 600 nm and adjusted to 50 ng/μL.

#### DNA Supercoiling Assay

The 0.75 mM test compound solution was diluted to obtain final concentrations of 25, 8.33, 2.78, 0.93, 0.31, 0.1, and 0.03 μM. For a singular determination 7.9 μL H_2_O, 3 μL DNA gyrase buffer (Inspiralis) and 0.1 μL *E. coli* gyrase (5 U/μL; Inspiralis) were added to a 0.2 mL test tube and a control tube. One μL of the compound dilution series was added to the test tube. Both tubes were vortexed. One μL relaxed plasmid DNA (50 ng) was diluted with 2 μL water, added to each tube and vortexed. The mixtures were incubated for 30 min at 37 °C and the reaction stopped by increased temperature at 60 °C for 10 min.

#### Agarose Gel Electrophoresis

Three μL agarose gel loading buffer was added to each sample and the control. Fifteen μL of the sample was loaded to the 0.8% agarose gel. The gel was run at 25 min at 100 V and subsequently stained with ethidium bromide (1 μg/mL) for 5 min. The ethidium bromide fluorescence was documented under a UV lamp. The gel was divided into lanes. The intensity of the staining was assessed by Image Lab 5.0 (BioRad). By densitometric analysis, each lane was analyzed for bands corresponding to the coiled pUC19 DNA. The intensity of the bands was determined by relative intensity to the untreated control. A graph was generated with the concentration of the test sample at the *X*-axis and the relative intensity of the pUC19 DNA band at the *Y*-axis. The IC_50_ values were calculated by nonlinear regression by graph pad prism.^27^

### *E. coli* Gyrase Supercoiling Inhibition Assay and Topoisomerase IV Relaxation Assay

Both assay kits were purchased from Inspiralis (Norwich, U.K.). The assays were performed according to the manufacturer’s instructions, with one modification. Prior to addition of either relaxed or supercoiled pBR322 plasmid, the enzyme was preincubated with the serial dilutions of investigated compound. In short, one unit of enzyme was pre incubated with the compound at room temperature for 15 min, final concentration of DMSO did not exceed 1%. After 15 min, 0.5 μg of either relaxed or supercoiled plasmid was added and the reaction was incubated for 30 min at 37 °C. Next, the reaction was stopped by addition of chloroform/isoamyl alcohol (v/v, 24.1) and GSTEB buffer. Samples were briefly vortexed and centrifuged for 1 min at 15,000 rpm. Upper aqueous phase was loaded onto a 1% TAE gel and ran at 90 V until the dye traveled 6–7 cm from the loading pocket to ensure sufficient separation of relaxed and supercoiled topoisomers. Next, the gels were stained in 1 μg/mL ethidium bromide bath for 15 min and destained in water for 5 min. In the final step, the gels were visualized with a gel documentation system. Gel band intensities were determined using ImageJ and normalized to the control. IC_50_ values were determined using nonlinear regression in graph pad prism.^27^

### Determination of Cytotoxicity against HepG2 and CHO Cell Lines

The cytotoxicity assay was conducted as described previously.^28^ In brief, the epithelial cell line HepG2 (ATCC HB-8065TM) was cultivated in Dulbecco’s modified Eagle’s medium (DMEM) with 10% heat-inactivated fetal calf serum (FCS) at 37 °C and 5% CO_2_. Similarly, CHO cells (ATCC CCL-61) were cultivated in Gibco Ham’s F-12K (Kaighn’s) medium with 10% heat-inactivated fetal calf serum (FCS) at 37 °C and 5% CO_2_. Cells were tested for mycoplasma contamination *via* electron microscopy before use. HepG2 cells as well as CHO-cells were seeded into a 96-well plate (Nunc, Roskilde, Denmark) and grown to 75% confluency. CN-CC-861 (**13**) was tested in concentrations ranging from 0.01 to 300 μM for 24 h with a residual DMSO assay concentration of 1%. All assays were conducted in triplicates.

### Determination of Plasma Stability

The plasma stability assay was conducted as described previously^28^ with procaine, procainamide and propoxycaine as controls. Plasma stability was determined for CN-CC-861 (**13**), **16**, **24**, **26**, **27**, **38**, **39**, **41** and **42**. All samples were analyzed *via* HPLC-MS/MS.

### Determination of Plasma Protein Binding

The plasma protein binding assay was conducted as described previously^29^ with naproxen as control. Plasma protein binding was determined for CN-CC-861 (**13**), **16**, **24**, **26**, **27**, **38**, **39**, **41** and **42**. All samples were analyzed *via* HPLC-MS/MS.

### HPLC-MS/MS Analysis of Cystobactamids

Samples were analyzed using an Agilent 1290 Infinity II HPLC system coupled to an AB Sciex QTrap 6500plus mass spectrometer. LC conditions were as follows: column: Agilent Zorbax Eclipse Plus C18, 50 mm × 2.1 mm, 1.8 μm; temperature: 30 °C; injection volume: 1 μL per sample; flow rate: 700 μL min^–1^. Samples were run under acidic conditions. Solvents: A: water +0.1% formic acid; solvent B: 95% acetonitrile/5% H_2_O + 0.1% formic acid. The following gradient was applied: 99% A at 0 min, 99% A until 0.1 min, 99–50% A from 0.1 to 3.5 min, 50–0% A from 3.5 min until 3.8 min, 0–99% A from 3.8 min until 4.7 min. The mass spectrometer was run in positive and negative mode with multiple reaction monitoring (MRM). Mass transitions for controls and compounds are depicted in SI Table 7. Samples were analyzed using MultiQuant 3.0 software (AB Sciex).

The HPLC traces were determined and analyzed using an Agilent 1100 HPLC system. LC conditions were as follows: column: Phenomenex Aeris PEPTIDE XB-C18, 50 mm × 2.1 mm, 3.6 μm; temperature: 25 °C; injection volume: 10 μL of a 1 mg/mL stock solution in DMSO; flow rate: 700 μL min^–1^. Samples were run under acidic conditions. Solvents A: water +0.1% trifluoroacetic acid; solvent B acetonitrile +0.1% trifluoroacetic acid. The following gradient was applied: 95% A at 0 min, 95% A until 3.0 min, 95–0% A from 3.0 to 23.0 min, 0% A from 23 to 28 min, 0–95% A from 28 to 28.1 min, 95% A from 28.1 to 33 min. The UV detection and quantification was carried out at 254 nm.

### *In Vivo* Efficacy Study of CN-CC-861 (**13**) in an *E. coli* Thigh Infection Model

The inoculum of the *E. coli* ATCC 25922 was diluted from a frozen stock to 1.4 × 10^6^ cfu/mL. *N* = 5 animals per group were used. *N* = 4 animals were used for the pretreatment group. Mice were rendered neutropenic by administration of 150 and 100 mg/kg cyclophosphamide intraperitoneally on day −4 and −1, respectively. On the day of infection (day 0), mice received 50 μL of the inoculum with *E. coli* into each lateral thigh under isoflurane aesthesia. While still under anesthesia, mice were administered a dose of Buprenorphine (0.03 mg/kg) subcutaneously for pain relief. Treatment started 1 h post infection. As vehicle hydroxyl-propyl-β cyclodextrin in Tris-buffer 1% at pH 9.0 (20:80 (w/v)) was used. For dissolution of cystobactamids vehicle was added to the respective amount of cystobactamid and sonicated for 10 min at 35 kHz. Then, the solution was stirred overnight with high speed at ambient temperature and protected from light. CN-DM-861 and CN-CC-861 were both dosed q6h starting from 1 h post infection. CN-DM-861 was dosed at 20, 40, and 50 mg/kg/day, whereas CN-CC-861 was dosed at 20, 40, and 80 mg/kg/day. Twenty-five hours after infection, mice were euthanized, blood was removed from the heart, thighs were aseptically removed. Twenty-five hours after infection the clinical score of every individual animal was assessed. When animals reached the humane end point, they were euthanized earlier and blood was removed from the heart and thighs were aseptically removed. Whole blood was collected into Eppendorf tubes coated with 0.5 M EDTA. Organs were homogenized in PBS. Thighs were plated onto agar plates in duplicates in serial dilutions and incubated at 37 °C for 24 h. The Evotec guidelines for animal testing are aligned with the 3Rs (Replacement, Reduction and Refinement) principles of Russel and Burch. These principles work toward good laboratory animal welfare and the principle of 3Rs is a holistic and integral part of R&D processes at Evotec. The responsible use of animals remains essential in research. Animals remain a small but an integral part of a comprehensive research that includes alternative methods and clinical research. Evotec animal facilities are AAALAC accredited or in the process of first accreditation and undergo regular visits by AAALAC. When animal experimentation is necessary, great care is taken to choose the most appropriate animal species for the research and optimize the study design to ensure that the results will be as meaningful as possible. All studies are designed to gain the maximum information from the fewest number of animals and the lowest burden possible. Each proposed use of animals is reviewed and approved by our veterinarians and scientists with an emphasis on eliminating or minimizing any potential pain or distress which may be experienced by the animals. Our standards of animal care and welfare meet or exceed those required by applicable local, national, or international laws and regulations. All Evotec personnel involved in the care, welfare and use of animals are trained to ensure that they are competent, aware of ethical issues and demonstrate respect and humane treatment toward the animals in their care within the procedures required to complete the proposed work.

## Supporting Information Available

MIC tables including **13**, **21**-**27**, **32**, **33**, **37**–**43**; antibiotic activities of CN-DM-861 (**4**), CN-CC-861 (**13**) and ciprofloxacin (CIP) against susceptible and multiresistant bacteria; docking study with Albi-1, **17** and **18**; mass transitions of the internal standard (caffeine), controls and cystobactamids; synthetic schemes; ^1^H and ^13^C NMR spectra; HPLC chromatograms for CN-CC 861 (**13**) and exemplified for **17**, **21**, **38** and **42** (PDF)
Molecular formula strings (CSV)

## Abbreviations Used

cfu: colony-forming unit
CIP: ciprofloxacin
Cryo-EM: cryogenic electron microscopy
EEDQ: ethyl 2-ethoxyquinoline-1(2H)-carboxylate
FoR: frequency of resistance
LPS: lipopolysaccharide
PABA: *para*-aminobenzoic acid
T3P: propanephosphonic acid anhydride
TI IV: topoisomerase IV
WHO: World Health Organization
