# Supplementary material for: Optimization of the Central α-Amino Acid in Cystobactamids to the Broad-Spectrum, Resistance-Breaking Antibiotic CN-CC-861
Source: J Med Chem. 2024 Sep 20;67(19):17162–90. doi: 10.1021/acs.jmedchem.4c00927 (PMC11472329; doi:10.1021/acs.jmedchem.4c00927)
Supplement: Supplementary file 1 — jm4c00927_si_001.pdf [file jm4c00927_si_001.pdf]

Supplementary Information

Optimization of the central α-amino acid in cystobactamids to the broad-spectrum, resistance-breaking antibiotic CN-CC-861

Daniel Kohnhäuser[1], Tim Seedorf[2], Katarina Cirnski[3,4], Dominik Heimann[1], Janetta Coetzee[3,4], Sylvie Sordello[5], Jana Richter[1], Moritz Stappert[1], Jean-Francois Sabuco[5], David Corbett[5], Eric Bacqué[5], Katharina Rox[1,4], Jennifer Herrmann[3,4], Aurelie Vassort[5], Rolf Müller[3,4], Andreas Kirschning[2,6], Mark Brönstrup[1,2,4,]*

Author Address

[1] Department of Chemical Biology, Helmholtz Centre for Infection Research, Inhoffenstraße 7, 38124 Braunschweig, Germany.

[2] Institute of Organic Chemistry and Biomolecular Drug Research Centre (BMWZ), Leibniz University Hannover, Schneiderberg 1B, 30167, Hannover, Germany.

[3] Microbial Natural Products, Helmholtz Institute for Pharmaceutical Research Saarland (HIPS), Helmholtz Centre for Infection Research (HZI), Department of Pharmacy at Saarland University, Campus Building E8.1, 66123 Saarbrücken (Germany).

[4] German Center for Infection Research (DZIF), Site Hannover-Braunschweig, Inhoffenstraße 7, 38124 Braunschweig, Germany.

[5] Evotec ID, 1541 Avenue Marcel Merieux, 69289 Marcy l'Etoile, France.

[6] Uppsala Biomedical Center (BMC), Uppsala University, Husargatan 3, 752 37 Uppsala, Sweden

Corresponding Author

Mark Brönstrup, e*mail: mark.broenstrup@helmholtz-hzi.de*

Table of content

SI Tables 2

Docking Study 12

Synthesis of Cystobactamide Derivatives 15

NMR Spectra of Final Compounds 36

HPLC chromatograms 68

References 70

SI Tables

Table S1. MIC values of selected compounds on an extended panel of bacteria compared to CN-DM-861 and ciprofloxacin (CIP) determined by standard procedures[5].

	MIC (µg/mL)						
	**CN-DM-861**	**CIP**	**23**	**24**	**27**	**32**	**33**
Gram-negative							
A. baumannii DSM 30008	0.5	0.2-0.32	0.25	0.06	0.25	0.4	2
A. baumannii ATCC BAA-1710	64	> 6.4	0.25-0.5	0.06	1	0.05	2
A. baumannii CIP-105742	0.06	≤ 0.03-0.25	0.06	0.016	0.5	0.05	0.25
A. baumannii CIP-107292	64	64	0.5-4	4	4	2	> 64
A. baumannii R835	2-64	32	4	2	> 8	6.4	> 64
A. baumannii NCTC 13301	> 64	32	0.8	0.5	4	0.06-0.125	> 64
A. baumannii ACC00445	>32	n.d.	4	1	n.d.	n.d.	n.d.
E. aerogenes CIP-106754	> 64	> 6.4	> 64	1	> 6.4	2-4	> 64
K. pneumoniae CIP-104298	4	0.025	> 64	> 64	32	> 6.4	1
K. pneumoniae KP10581 (waaC::Tn30)	0.125-2	> 6.4	> 64	0.5	> 6.4	0.1-0.2	> 64
K. pneumoniae R-1525 (QnrA1)	> 64	> 64	> 64	> 64	> 6.4	> 64	> 64
K. pneumoniae DSM 30104	0.25-64	0.01-0.1	> 64	n.d.	1	n.d.	n.d.
P. aeruginosa PAO1	n.d.	n.d.	n.d.	8	n.d.	> 64	> 64
P. aeruginosa CRPA/4MRGN (clin HAP/VAP, pneumo isolate BAL #2182MHH, 2021)	n.d.	n.d.	> 64	> 64	> 64	> 64	> 64
E. cloacae ATCC BAA-2468	1	> 6.4	4	4	6.4	2	0.25
P. vulgaris DSM 2140	0.25-0.5	≤ 0.06	1	n.d.	4	n.d.	n.d.
S. marcescens DSM 30121	64	0.2	> 64	n.d.	2	n.d.	n.d.
Gram-positive							
E. faecium DSM 17050	n.d.	0.05-4	≤ 0.03	32	> 6.4	0.01	> 64
S. aureus NRS384	n.d.	2	0.5	0.06	n.d.	n.d.	n.d.
S. aureus MRSA (clin HAP/VAP, pneumo isolate BAL #2524MHH, 2022)	n.d.	n.d.	n.d.	≤ 0.03	n.d.	≤ 0.03	> 64

n.d.: not determined.

Table S2. MIC values of selected compounds on an extended panel of bacteria compared to CN-DM-861 and ciprofloxacin (CIP) determined by standard procedures[5].

	MIC (µg/mL)				
	**CN-DM-861**	**CIP**	**37**	**38**	**39**
Gram-negative					
A. baumannii DSM 30008	0.5	0.2-0.32	0.03	0.016-0.03	0.125-0.25
A. baumannii ATCC BAA-1710	64	> 6.4	0.25-0.5	0.06-0.125	0.5-1
A. baumannii CIP-105742	0.06	≤ 0.03-0.25	≤ 0.0038	0.016	0.06
A. baumannii CIP-107292	64	64	0.5	> 8	2-4
A. baumannii R835	2-64	32	0.5	4	2-4
A. baumannii ACC00535	> 32	n.d.	n.d.	n.d.	0.5
A. baumannii NCTC 13301	> 64	32	1.25	0.06	0.5
E. aerogenes CIP-106754	> 64	> 6.4	2	1	> 64
E. cloacae ATCC BAA-2468	1	> 6.4	2	0.25	> 64
K. pneumoniae CIP-104298	4	0.025	≤ 0.3	1	> 64
K. pneumoniae KP10581 (waaC::Tn30)	0.125-2	> 6.4	0.25	≤ 0.03	> 64
K. pneumoniae R-1525 (QnrA1)	> 64	> 64	1.25	4	> 64
Gram-positive					
E. faecium DSM 17050	n.d.	0.05-4	≤ 0.06	0.125	> 64
S. pneumoniae DSM 11865	n.d.	> 6.4	≤ 0.3	0.5	> 64

n.d.: not determined.

Table S3. MIC values of selected compounds on an extended panel of bacteria compared to CN-DM-861 and ciprofloxacin (CIP) determined by standard procedures[5].

	MIC (µg/mL)					
	**CN-DM-861**	**CIP**	**40**	**41**	**42**	**43**
Gram-negative						
A. baumannii DSM 30008	0.5	0.2-0.32	n.d.	0.06-0.125	≤ 0.0038	n.d.
A. baumannii ATCC BAA-1710	64	> 6.4	0.25	1	0.25	0.05
A. baumannii CIP-105742	0.06	≤ 0.03-0.25	> 64	0.0075-0.015	≤ 0.0038	n.d.
A. baumannii CIP-107292	64	64	8	8	1	2-4
A. baumannii R835	2-64	32	1	8	2-4	n.d.
A. baumannii ACC00535	> 32	n.d.	n.d.	0.25	0.5	n.d.
A. baumannii NCTC 13301	> 64	32	4	0.25	0.5	0.125-0.25
E. aerogenes CIP-106754	n.d.	> 6.4	0.125	1	8	1
E. cloacae ATCC BAA-2468	1	> 6.4	0.01	≤ 0.03	0.25	n.d.
K. pneumoniae CIP-104298	4	0.025	0.25	0.5	0.5	n.d.
K. pneumoniae KP10581 (waaC::Tn30)	0.125-2	> 6.4	0.5	≤ 0.03	≤ 0.03	0.025
K. pneumoniae R-1525 (QnrA1)	> 64	> 64	0.5	2	1	32-64
P. aeruginosa PAO1	n.d.	n.d.	> 64	n.d.	n.d.	4
P. aeruginosa CRPA/4MRGN (clin HAP/VAP, pneumo isolate BAL #2182MHH, 2021)	n.d.	n.d.	> 64	> 6.4	3.2	32
Gram-positive						
E. faecium DSM 17050	n.d.	0.05-4	0.04	≤ 0.03	≤ 0.03	0.0025
S. aureus MRSA (clin HAP/VAP, pneumo isolate BAL #2524MHH, 2022)	n.d.	n.d.	0.05	n.d.	n.d.	≤ 0.03
S. pneumoniae DSM 11865 (PRSP)	n.d.	> 6.4	0.004	1	> 64	n.d.

n.d.: not determined.

Table S4. MIC values of selected compounds on an extended panel of bacteria compared to CN-DM-861 and ciprofloxacin (CIP) by standard procedures[5].

	CN-DM-861	CIP	CN-CC-861 (13)	21	26	22	25
Gram-negative							
A. baumannii ATCC BAA-1710	64	> 6.4	0.06 – 0.25	≤ 0.03	1	0.125	> 64
A. baumannii CIP-105742	0.06	≤ 0.03-0.25	0.02	≤ 0.03	≤ 0.03	≤ 0.03	> 64
A. baumannii CIP-107292	64	64	0.5 – 1	2	2	2	> 64
A. baumannii R835	2-64	32	1	0.5	2	1	> 64
A. baumannii NCTC 13301	> 64	32	2	0.25	n.d.	0.5	> 64
E. aerogenes CIP-106754	n.d.	> 6.4	0.125 – 4	4	> 64	2	2
E. cloacae ATCC BAA-2468	1	> 6.4	0.06 – 0.5	0.5	0.5	0.5	2
K. pneumoniae CIP-104298	4	0.025	8	> 64	0.25	> 64	> 64
K. pneumoniae KP10581 (waaC::Tn30)	0.125-2	> 6.4	0.06 – 0.25	> 64	0.25	> 64	> 64
K. pneumoniae R-1525 (QnrA1)	> 64	> 64	16 - > 64	> 64	0.25	> 64	0.5
P. aeruginosa CRPA/4MRGN (clin HAP/VAP, pneumo isolate BAL #2182MHH, 2021)	n.d.	n.d.	> 64	32	> 64	> 64	≤ 0.03
Gram-positive							
E. faecium DSM 17050	n.d.	0.05-4	≤ 0.03	≤ 0.03	≤ 0.03	≤ 0.03	≤ 0.03

n.d.: not determined.

Table S5. Antibiotic activities of CN-DM-861, CN-CC-861 and ciprofloxacin (CIP) against susceptible and multiresistant bacteria.

Genus	Species	Strain	MIC (µg/mL)			Resistance phenotype	Resistant against:	Susceptible against:
			CN-DM-861	CN-CC-861	CIP			
Enterococcus	*E. faecium*	EF66	32	≤ 0.03	> 6.4	VRE	Ampicillin, Cefuroxim, Imipenem, Vancomycin	Gentamicin (high-level), Linezolid, Tigecyclin
Staphylococcus	*S. aureus*	Sa6	1	≤ 0.03	0.2	MSSA	Penicillin G	Cefazolin, Clindamycin, Co-trimoxazol, Daptomycin, Doxycyclin, Erythromycin, Fosfomycin, Fusidinsäure, Linezolid, Gentamicin, Moxifloxacin, Tigecyclin, Vancomycin
	S. aureus	Sa20	1	≤ 0.03	0.4	MSSA	Penicillin G	Clindamycin, Co-trimoxazol, Daptomycin, Erythromycin, Flucloxacillin, Fosfomycin, Fusidinsäure, Linezolid, Gentamicin, Tigecyclin, Vancomycin
	S. aureus	Sa26	0.25	≤ 0.03	0.2	MSSA	Penicillin G	Clindamycin, Co-trimoxazol, Daptomycin, Erythromycin, Fosfomycin, Fusidinsäure, Linezolid, Gentamicin, Oxacillin, Tetracyclin, Tigecyclin, Vancomycin
	S. aureus	Sa37	8	0.125	> 6.4	MRSA	Cefazolin, Ciprofloxacin, Clindamycin, Erythromycin, Flucloxacillin, Moxifloxacin, Penicillin G	Co-trimoxazol, Daptomycin, Doxycyclin, Fosfomycin, Fusidinsäure, Gentamicin, Linezolid, Tigecyclin, Vancomycin
	S. aureus	Sa40	32	0.125	> 6.4	MRSA	Cefuroxim, Ciprofloxacin, Clindamycin, Erythromycin, Moxifloxacin, Oxacillin, Penicillin G	Co-trimoxazol, Daptomycin, Fosfomycin, Fusidinsäure, Gentamicin, Linezolid, Tetracyclin, Tigecyclin, Vancomycin
	S. aureus	Sa38	4	0.125	> 6.4	MSSA	Ciprofloxacin, Moxifloxacin, Penicillin G	Cefazolin, Clindamycin, Co-trimoxazol, Daptomycin, Doxycyclin, Erythromycin, Flucloxacillin, Fosfomycin, Fusidinsäure, Linezolid,

								Gentamicin, Tigecyclin, Vancomycin
	S. aureus	Sa45	> 64	0.5	> 6.4	MRSA	Cefuroxim, Ciprofloxacin, Clindamycin, Erythromycin, Moxifloxacin, Oxacillin, Penicillin G	Linezolid, Tigecyclin, Vancomycin
	S. aureus	Sa46	32	0.5	6.4	MRSA	Cefuroxim, Ciprofloxacin, Co-Trimoxacol, Clindamycin, Gentamicin, Moxifloxacin, Oxacillin, Penicillin G	Clindamycin, Daptomycin, Erythromycin, Fosfomycin, Fusidinsäure, Linezolid, Tetracyclin, Tigecyclin, Vancomycin
	S. aureus	Sa68	1	≤ 0.03	0.4	MSSA	-	Cefuroxim, Clindamycin, Co-trimoxazol, Daptomycin, Erythromycin, Fosfomycin, Fusidinsäure, Linezolid, Gentamicin, Moxifloxacin, Oxacillin, Tetracyclin, Tigecyclin, Vancomycin
	S. aureus	Sa69	> 64	0.25	> 6.4	MRSA	Cefuroxim, Ciprofloxacin, Clindamycin, Erythromycin, Moxifloxacin, Oxacillin, Penicillin G	Co-trimoxazol, Daptomycin, Fosfomycin, Fusidinsäure, Linezolid, Gentamicin, Tetracyclin, Tigecyclin, Vancomycin
Median MIC			4-8	0.125	6.4-> 6.4			
Klebsiella	*K. oxytoca*	K6	0.25	≤ 0.03	0.006		Ampicillin, Ampicillin/Sulbactam[1], Cefuroxim[1]	Cefotaxim, Cefpodoxim, Ceftazidim, Ciprofloxacin, Co-trimoxazol, Ertapenem, Gentamicin, Imipenem, Meropenem, Moxifloxacin, Piperacillin, Piperacillin/Tazobactam, Tigecyclin
	K. oxytoca	K24	2	0.25	0.0125		Ampicillin, Ampicillin/Sulbactam, Cefuroxim[1],	Cefotaxim, Cefpodoxim, Ceftazidim, Ciprofloxacin, Co-trimoxazol, Ertapenem, Gentamicin, Imipenem, Meropenem, Moxifloxacin, Piperacillin, Piperacillin/Tazobactam, Tigecyclin
	K. oxytoca	K26	0.5	0.125	3.2	3MRGN	Ampicillin, Ampicillin/Sulbactam, Cefotaxim, Cefpodoxim,	Ertapenem, Gentamicin, Imipenem, Meropenem

							Ceftazidim, Cefuroxim, Ciprofloxacin, Co-trimoxazol, Moxifloxacin, Piperacillin, Piperacillin/Tazobactam	
	K. oxytoca	K27	0.25	0.125	0.6	3MRGN	Ampicillin, Ampicillin/Sulbactam, Cefalexin, Cefpodoxim, Ceftazidim, Cefuroxim, Ciprofloxacin, Co-Trimoxacol, Levofloxacin, Piperacillin, Trimethoprim	Fosfomycin, Gentamicin, Imipenem, Meropenem
	K. oxytoca	K33	0.5	0.25	0.0125	4MRGN	Ampicillin, Ampicillin/Sulbactam, Cefotaxim, Cefpodoxim, Ceftazidim, Cefuroxim, Ertapenem, Fosfomycin, Imipenem[1], Meropenem[1], Piperacillin, Piperacillin/Tazobactam	Amikacin, Aztreonam, Ceftazidim/Avibactam, Ciprofloxacin, Co-trimoxazol, Gentamicin, Moxifloxacin, Tobramycin, Tigecyclin
	K. pneumoniae	K46	2	8	0.05		Ampicillin, Piperacillin	Amikacin, Ampicillin/Sulbactam, Aztreonam, Cefepim, Cefotaxim, Ceftazidim, Ciprofloxacin, Co-trimoxazol, Fosfomycin, Gentamicin, Meropenem, Moxifloxacin, Piperacillin/Tazobactam, Polymyxin B/Colistin, Tigecyclin, Tobramycin, Trimethoprim
	K. pneumoniae	K47	1	32	> 6.4	4MRGN	Ampicillin, Ampicillin/Sulbactam, Cefalexin, Cefpodoxim, Ceftazidim, Cefuroxim, Ciprofloxacin, Co-Trimoxacol, Ertapenem, Fosfomycin, Gentamicin, Imipenem[1], Levofloxacin, Moxifloxacin, Piperacillin, Trimethoprim, Tobramycin	Amikacin, Polymyxin B/Colistin, Tigecyclin
	K. pneumoniae	K61	4	> 64	> 6.4	3MRGN	Ampicillin, Ampicillin/Sulbactam, Cefalexin, Cefpodoxim, Ceftazidim, Cefuroxim, Ciprofloxacin, Co-Trimoxacol, Gentamicin, Levofloxacin, Piperacillin, Trimethoprim	Fosfomycin, Imipenem, Meropenem
	K. pneumoniae	K62	8	8	> 6.4	4MRGN	Ampicillin, Amoxicillin/Clavulansäure,	Amikacin, Aztreonam, Fosfomycin, Tigecyclin

							Cefalexin, Cefotaxim, Cefpodoxim, Ceftazidim/Avibactam, Ceftazidim, Cefuroxim, Cefuroxim/Cefuroxim-Axetil, Cefepim, Ciprofloxacin, Co-Trimoxacol, Ertapenem, Imipenem[1], Levofloxacin, Meropenem[1], Moxifloxacin, Piperacillin, Piperazillin/Tazobactam, Trimethoprim	
	K. pneumoniae	K63	0.5	8	3.2	2MRGN	Ampicillin, Amoxicillin/Clavulansäure, Cefotaxim, Ceftazidim, Cefuroxim, Cefuroxim/Cefuroxim-Axetil, Cefepim, Ciprofloxacin, Co-Trimoxacol, Levofloxacin, Trimethoprim	Ertapenem, Fosfomycin, Meropenem, Piperacillin/Tazobactam
Median MIC			0.5-1	0.25-8	0.6-3.2			
Pseudomonas	*P. aeruginosa*	Pa33	> 64	> 64	0.4	4MRGN	Aztreonam, Cefepim, Ceftazidim, Ciprofloxacin, Imipenem, Meropenem, Piperacillin, Piperacillin/Tazobactam	Amikacin, Gentamicin, Polymyxin B/Colistin, Tobramycin
	P. aeruginosa	Pa34	16	1	3.2	4MRGN	Aztreonam, Cefepim, Ceftazidim, Ciprofloxacin, Gentamicin, Imipenem, Levofloxacin, Meropenem, Piperacillin	Ceftazidim/Avibactam, Ceftolozan/Tazobactam, Polymyxin B/Colistin, Tobramycin
	P. aeruginosa	Pa57	> 64	8	> 6.4	4MRGN	Amikazin, Aztreonam, Cefepim, Ceftazidim, Ceftazidim/Avibactam, Ceftolozan/Tazobactam, Ciprofloxacin, Fosfomycin, Gentamicin, Imipenem, Meropenem, Piperacillin, Piperacillin/Tazobactam, Tobramycin	Colistin
	P. aeruginosa	Pa59	8	2	0.2	3MRGN	Aztreonam, Cefepim, Ceftazidim, Imipenem, Meropenem, Piperacillin, Piperacillin/Tazobactam	Amikacin, Ciprofloxacin, Gentamicin, Tobramycin
	P. aeruginosa	Pa60	> 64	8	0.4	3MRGN	Aztreonam, Cefepim, Ceftazidim, Imipenem, Meropenem,	Amikacin, Ciprofloxacin, Gentamicin, Tobramycin

							Piperacillin, Piperacillin/Tazobactam	
	P. aeruginosa	Pa61	> 64	> 64	0.4	3MRGN	Aztreonam, Cefepim, Ceftazidim, Imipenem, Meropenem, Piperacillin, Piperacillin/Tazobactam	Amikacin, Ciprofloxacin, Gentamicin, Tobramycin
	P. aeruginosa	Pa62	> 64	8	3.2	3MRGN	Aztreonam, Cefepim, Ceftazidim, Ciprofloxacin, Piperacillin	Amikacin, Gentamicin, Imipenem, Meropenem, Tobramycin
	P. aeruginosa	Pa63	> 64	8	> 6.4	4MRGN	Aztreonam[1], Cefepim, Ceftazidim, Ciprofloxacin, Imipenem, Meropenem[1], Piperacillin, Piperacillin/Tazobactam	Amikacin, Gentamicin, PolymyxinB/Colistin, Tobramycin
	P. aeruginosa	Pa91	> 64	8	0.8	4MRGN	Aztreonam, Cefepim, Ceftazidim, Ciprofloxacin, Imipenem, Levofloxacin, Meropenem, Piperacillin	Gentamicin, Polymyxin B/Colistin, Tobramycin
	P. aeruginosa	Pa92	32	8	3.2	3MRGN	Cefepim, Ceftazidim, Ciprofloxacin, Levofloxacin, Piperacillin	Gentamicin, Imipenem, Meropenem, Tobramycin
	P. aeruginosa	Pa94	2	0.5	> 6.4	4MRGN	Amikacin, Cefepim, Ceftazidim, Ciprofloxacin, Gentamicin, Imipenem, Levofloxacin, Meropenem, Piperacillin/Tazobactam, Tobramycin	-
Median MIC			> 64	8	3.2			
Acinetobacter	*A. baumannii*	ABC3	4	≤ 0.03	0.1		-	Ciprofloxacin, Co-trimoxazol, Gentamicin, Imipenem, Meropenem
	A. baumannii complex	ABC7	4	0.125	> 6.4	3MRGN	Ciprofloxacin, Gentamicin, Meropenem	Amikacin, Co-Trimoxazol, Imipenem, PolymyxinB/Colistin, Tobramycin
	A. johnsonii	ABC12	≤ 0.03	≤ 0.03	0.05		-	Amikacin, Ciprofloxacin, Co-Trimoxazol, Gentamicin, Imipenem, Meropenem, Tobramycin
	A. lwoffii	ABC14	0.06	≤ 0.03	0.025		-	Ciprofloxacin, Gentamicin, Imipenem, Meropenem, Tobramycin

	A. ursingii	ABC22	> 64	4	0.1		-	Ciprofloxacin, Gentamicin, Imipenem, Meropenem, Tobramycin
	A. baumannii complex	ABC26	4	0.125	0.4	3MRGN	Ciprofloxacin[1]	Gentamicin, Imipenem, Meropenem
	A. baumannii complex	ABC28	1	0.06	0.2	3MRGN	Ciprofloxacin[1], Levofloxacin[1]	Co-Trimoxazol, Gentamicin, Imipenem, Meropenem
	A. baumannii complex	ABC29	16	0.5	0.4	3MRGN	Ciprofloxacin[1], Levofloxacin[1]	Co-trimoxazol, Gentamicin, Imipenem, Meropenem
Median MIC			4	0.06-0.125	0.1-0.2			

[1]intermediate resistant. 2/3/4 MRGN: Multidrug-resistant Gram-negative bacteria with resistance against 2, 3 or 4 of the 4 antibiotic groups acylureidopenicillins, third-generation cephalosporins, carbapenems or fluoroquinolones; example 3MRGN: Resistance against 3 of the 4 antibiotic groups. VRE: Vancomycin-resistant *Enterococcus*. MSSA: Methicillin-susceptible *Staphylococcus aureus*. MRSA: Methicillin-resistant *Staphylococcus aureus*.

Docking Study

Schrödinger Release 2023-3 (Glide, Schrödinger, LLC, New York, NY, 2023) was used for the docking study. Ligands were prepared using the default settings of LigPrep (Force field: OPLS4; Target pH: 7.00 ± 2.00 using Epik Classic). The Cryo-EM structure was prepared with the standard settings of the Protein Preparation Workflow, which, among other things, involves the assignment of bond orders and the correct protonation state for pH 7.4, the optimization of the H-bond network, a minimization of the protein and the removal of irrelevant water molecules. The Receptor Grid Generator was used to prepare the protein grid necessary for Glide docking. Albi-1 was used to determine the size of the enclosing box, since cystobactamids have a comparable size. Docking of the ligands was performed using Glide. The following settings were used: Precision: XP (extra precision); Ligand sampling: flexible; Intramolecular hydrogen bonds were rewarded. Since a pose determined for the double-deprotonated Albi-1 was closest to the natural pose found in the cryo-EM structure, the cystobactamids were also docked in the double-deprotonated form.

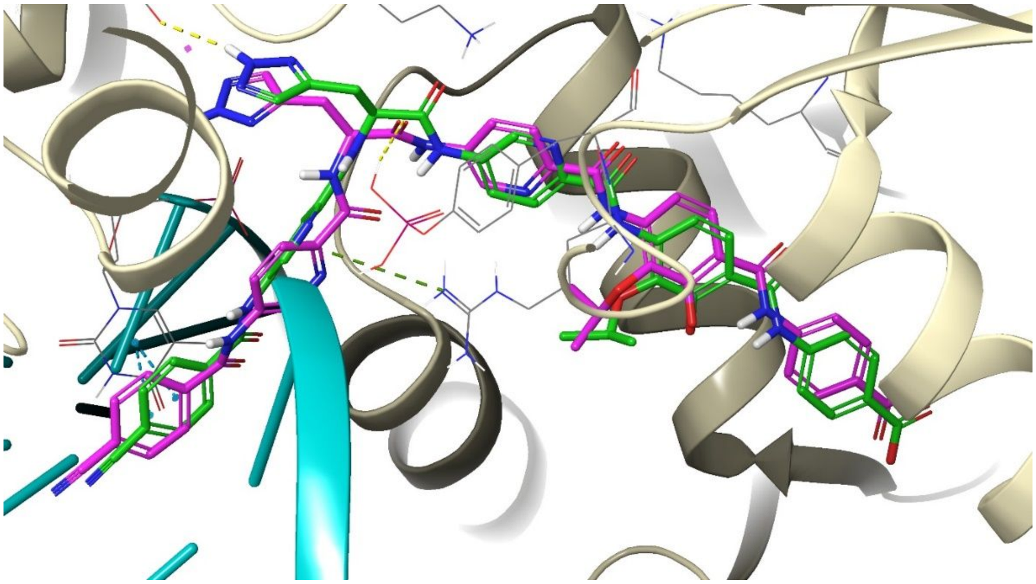

Figure S1. Cryo-EM structure of Albi-1 (green ligand) in *E. coli* gyrase holocomplex with 217 bp DNA (PDB: 7Z9K).[1] Double deprotonated Albi-1 (magenta ligand) docked into the protein-DNA complex with Glide.[2]

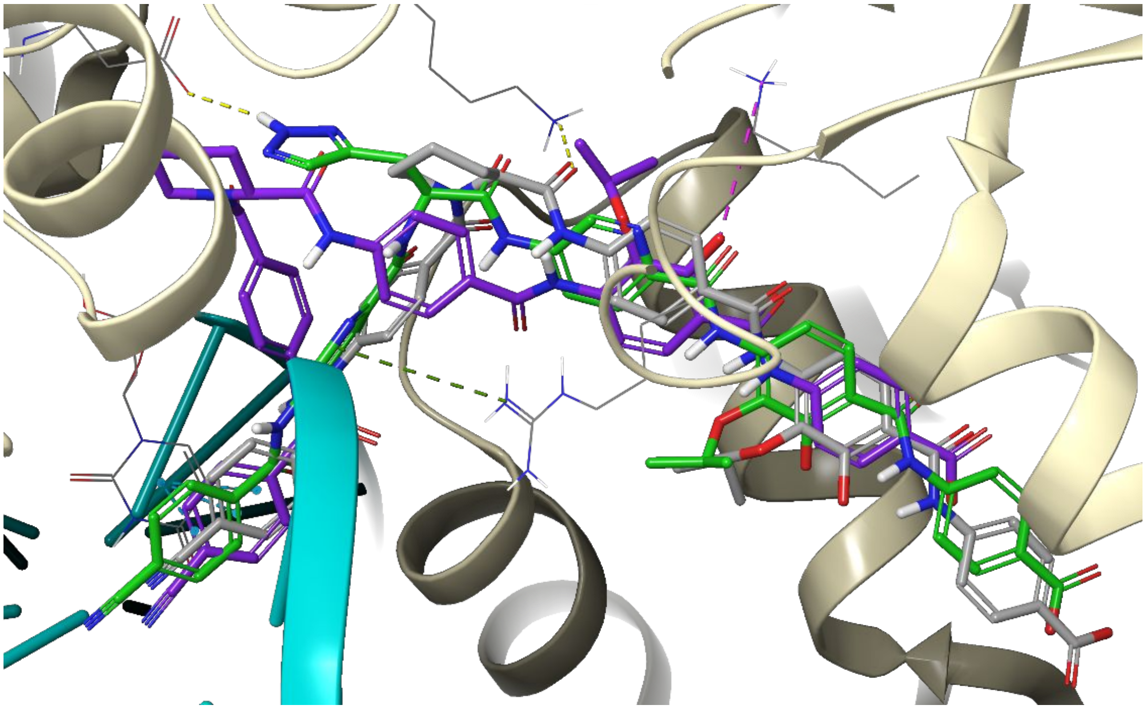

Figure S2. Cryo-EM structure of Albi-1 (green ligand) in *E. coli* gyrase (beige) holocomplex with 217 bp DNA (cyan) (PDB: 7Z9K).[1] **17** (grey ligand) and **18** (purple ligand) were modeled into the protein-DNA complex with Glide.[2]

Table S6. Docking scores of the obtained poses for the double deprotonated **17** and **18**.

Compound	Docking Score
**17**	-7.25
**17**	-6.03
**17**	-4.97
**17**	-4.06
**17**	-3.17
**18**	-1.66
**17**	-0.96
**18**	-0.68
**17**	-0.32
**18**	-0.27
**18**	1.54
**18**	3.58

Table S7: Mass transitions of the internal standard (caffeine), controls and cystobactamids.

	Q1 mass	Q3 mass	DP [V]	CE [V]	CXP [V]
**caffeine**	195.024	138.0	130	25	14
		110.0	130	25	14
**naproxen**	231.106	185.1	80	19	10
		170.2	80	33	12
**procaine**	235.744	163.0	80	21	18
		120.0	80	39	12
**procainamide**	236.773	100.0	80	21	12
		120.0	80	31	14
**propoxycaine**	294.738	100.1	80	17	12
		178.1	80	21	20
**verapamil**	454.688	165.0	1	35	28
		303.1	1	35	18
**CN-CC-861**	791.115	432.0	-240	-52	-21
		748.1	-240	-38	-39
**16**	846.140	765.0	-165	-38	-45
		432.0	-165	-60	-47
**24**	807.307	431.9	-215	-56	-41
		764.1	-215	-38	-13
		294.9	-215	-74	-19
**26**	782.098	432.0	-165	-54	-37
		739.2	-165	-44	-17
**38**	805.135	432.0	-170	-54	-19
		762.2	-170	-40	-37
**39**	805.129	432.0	-300	-54	-19
		762.1	-300	-38	-35
**41**	799.206	469.0	141	25	30
		450.1	141	19	22
**42**	799.204	469.0	156	23	22
		313.1	156	25	28
	821.171	684.1	251	43	44
		641.0	251	57	52

DP: declustering potential; CE: collision energy; CXP: collision cell exit potential.

Synthesis of Cystobactamide Derivatives

tert-Butyl 4-[4-(4-aminobenzamido)-2-(prop-2-en-1-yloxy)-3-(propan-2-yloxy)benzamido]benzoate (8)

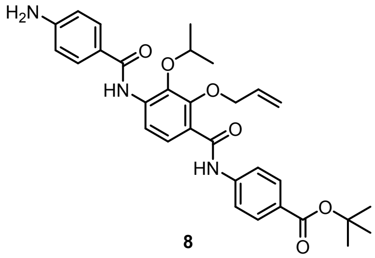

The compound was prepared according to the established literature procedure; see M. Moeller, M. D. Norris, T. Planke, K. Cirnski, J. Herrmann, R. Müller, A. Kirschning, *Organic Letters* **2019**, *21*, 8369-8372.

4-(4-Cyanobenzamido)benzoic acid (10)

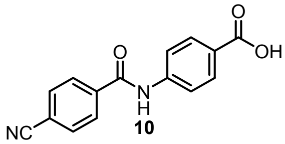

The compound was prepared according to the established literature procedure; see Dong, Y. *et al.*; *Bioorg. Med. Chem. Lett.* **2014**, 24, 3, 944-948.

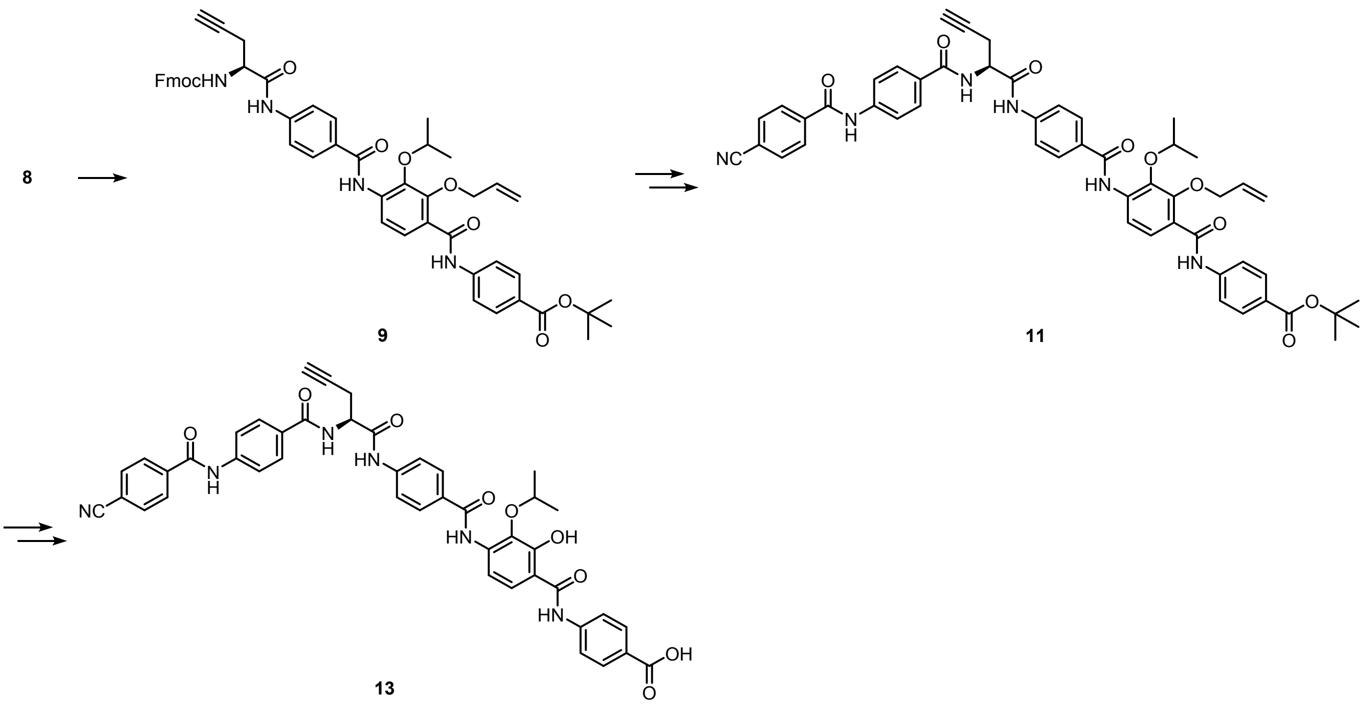

Scheme S1. Synthesis of compound **13** (CN-CC-861).

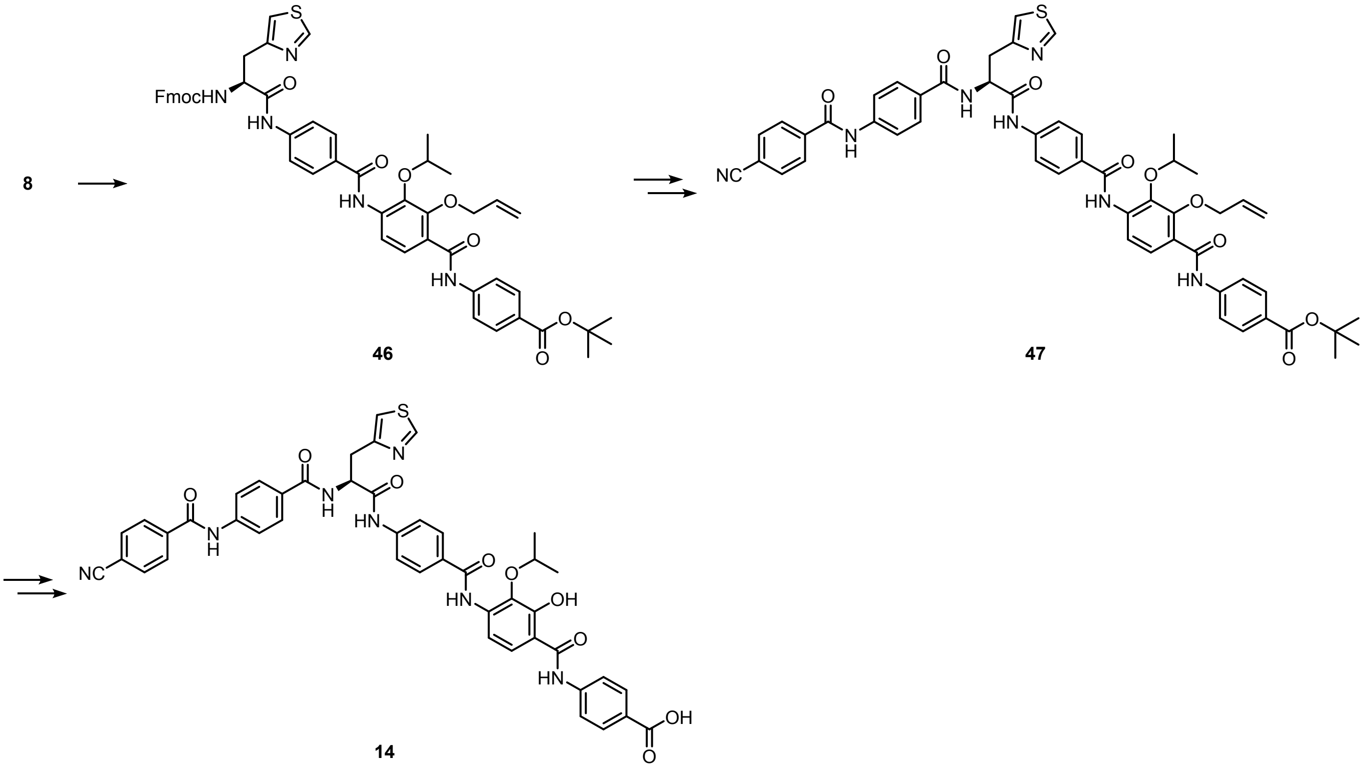

Scheme S2. Synthesis of compound **14**.

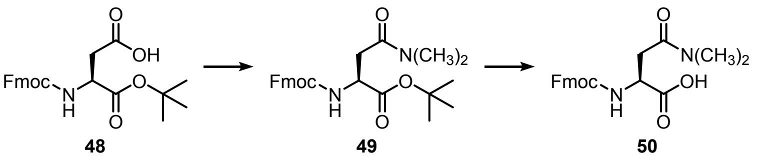

Scheme S3. Synthesis of compound **50**.

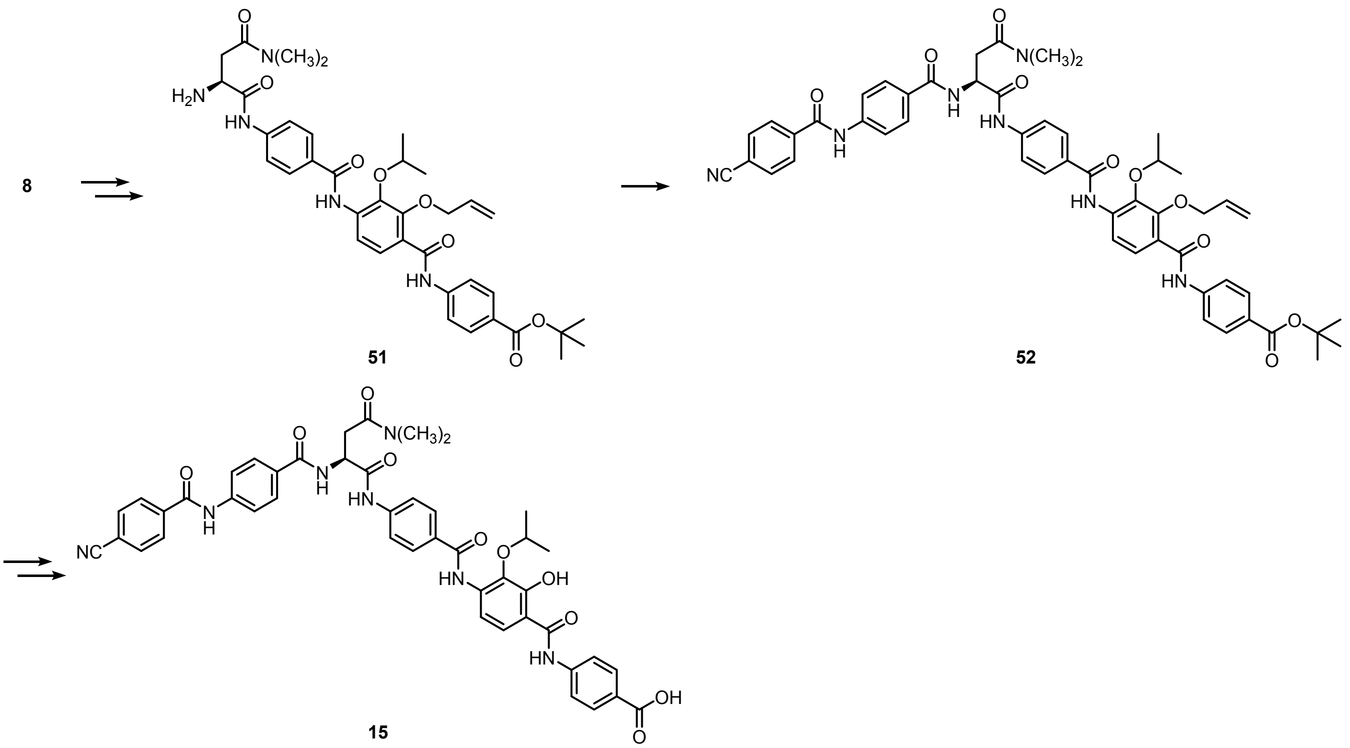

Scheme S4. Synthesis of compound **15**.

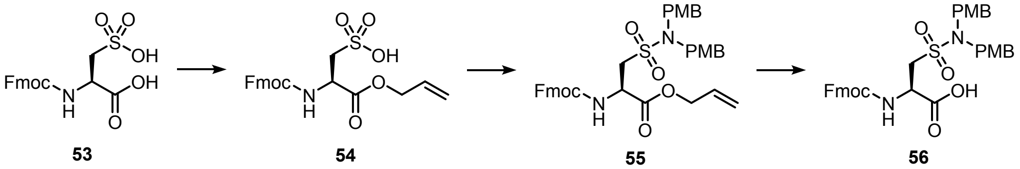

Scheme S5. Synthesis of compound **56**.

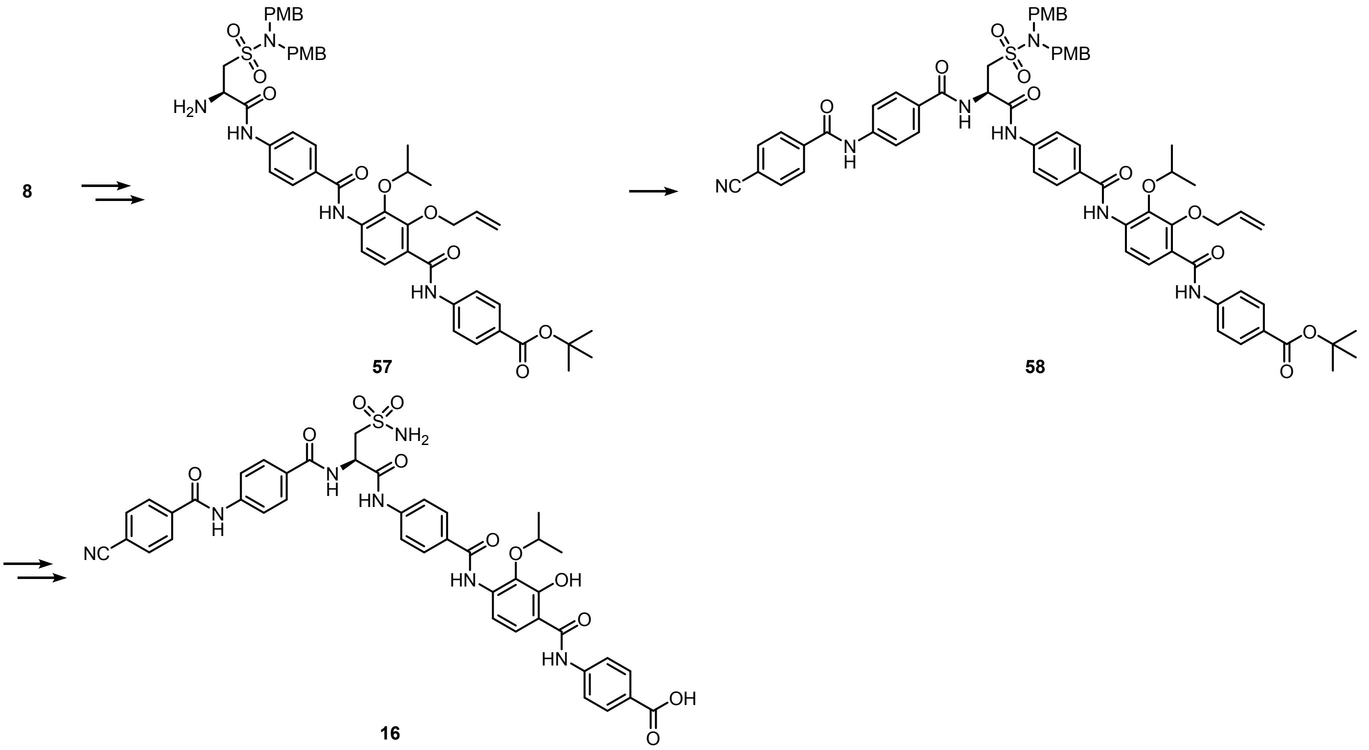

Scheme S6. Synthesis of compound **16**.

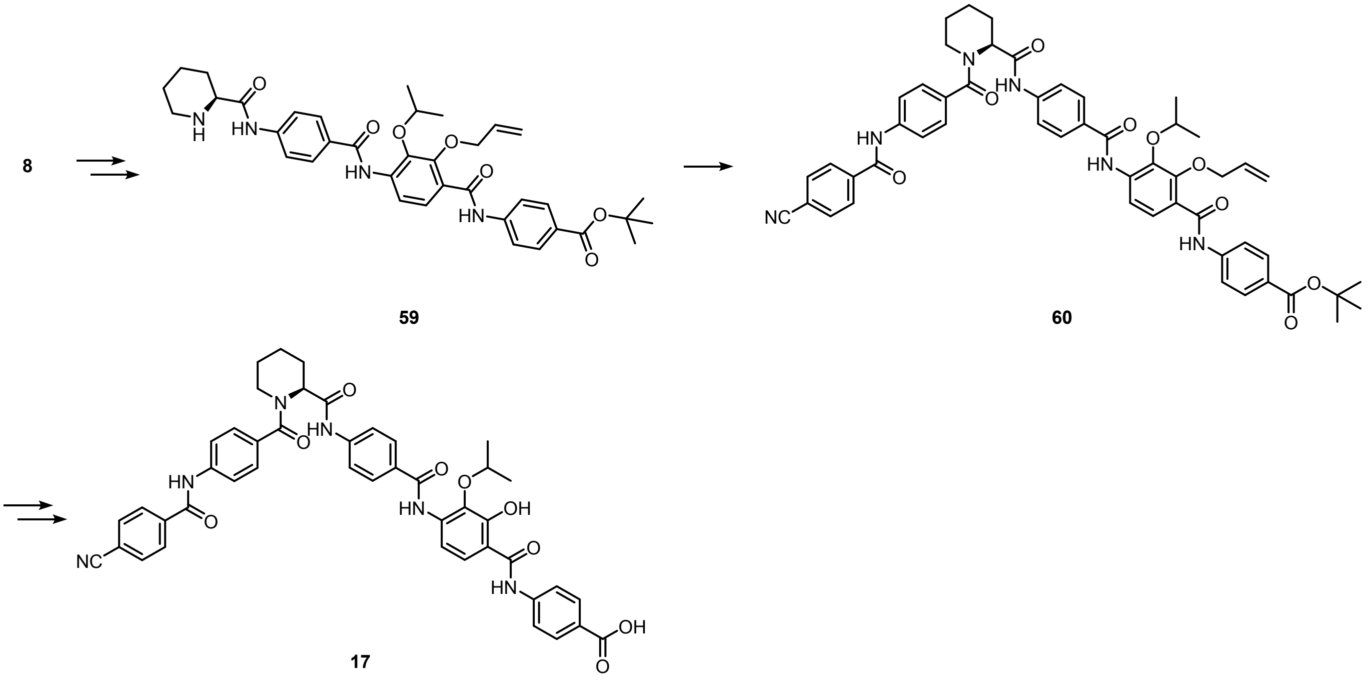

Scheme S7. Synthesis of compound **17**.

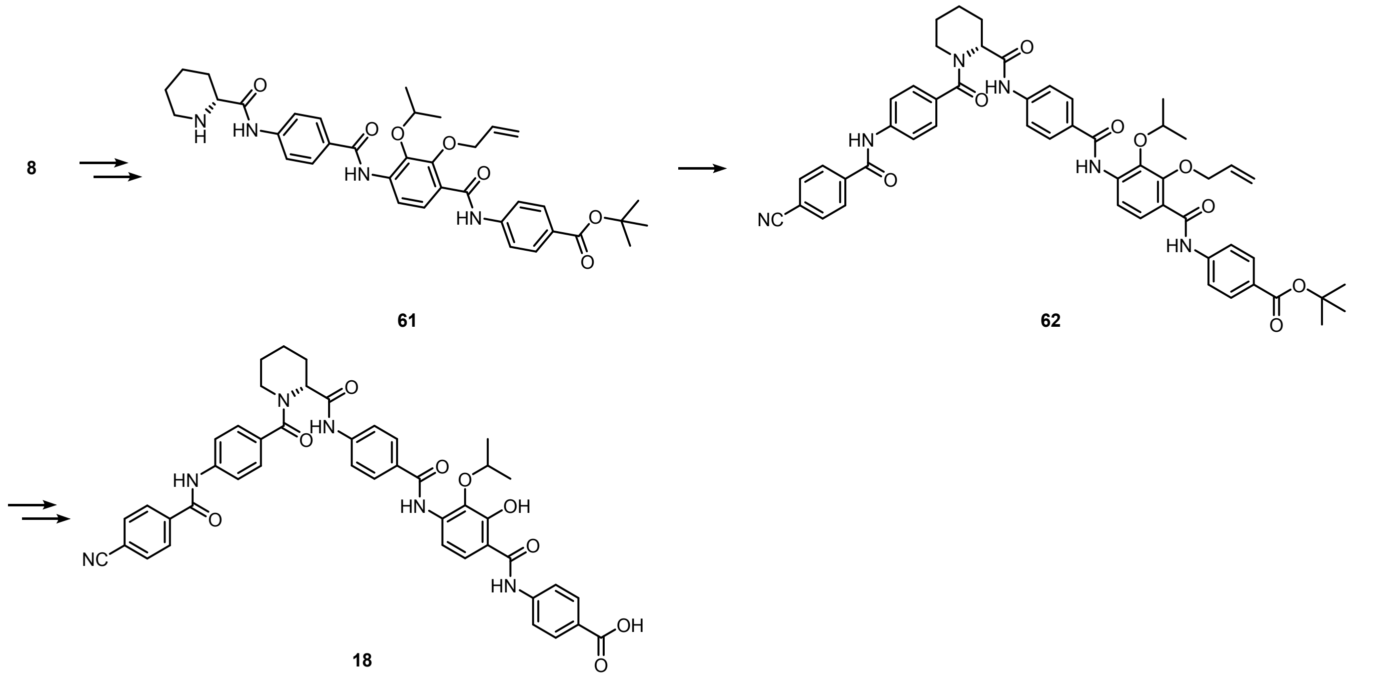

Scheme S8. Synthesis of compound **18**.

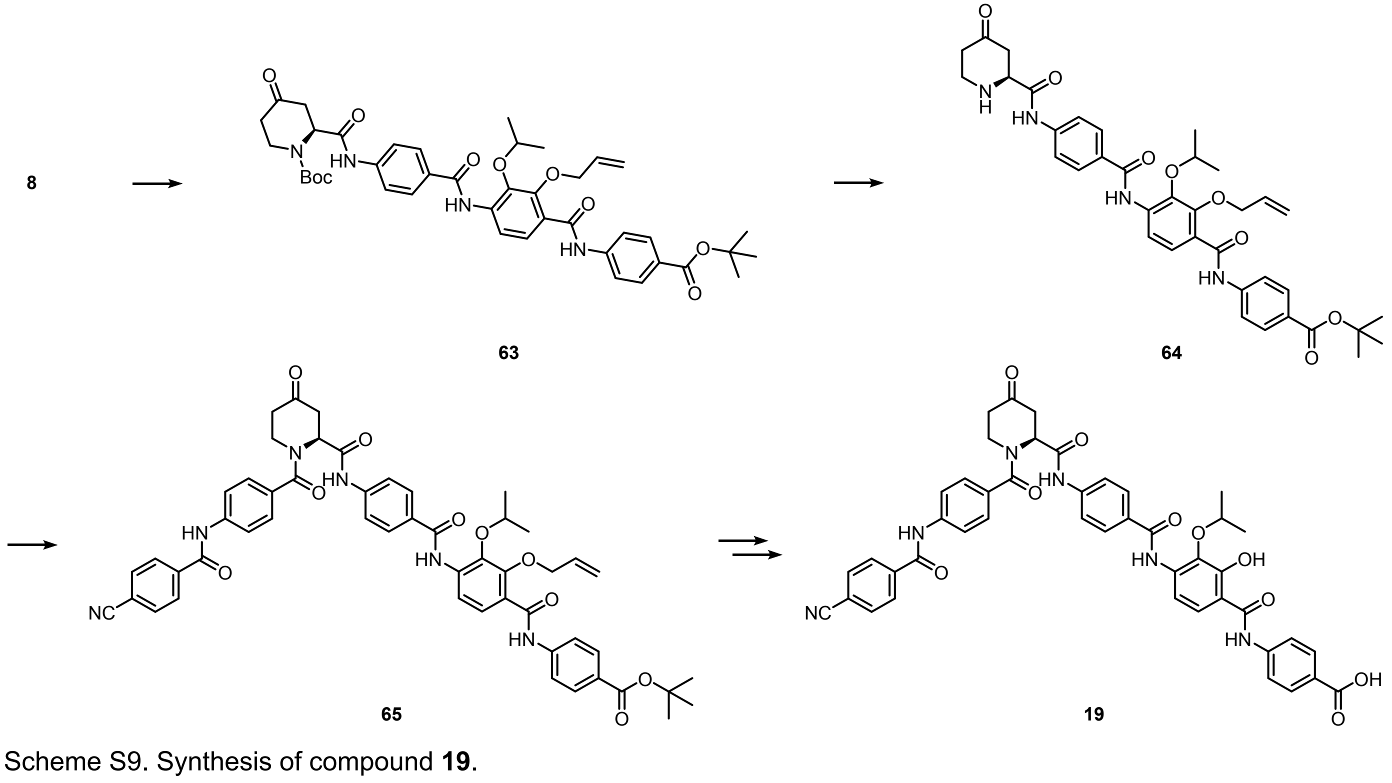

Scheme S9. Synthesis of compound **19**.

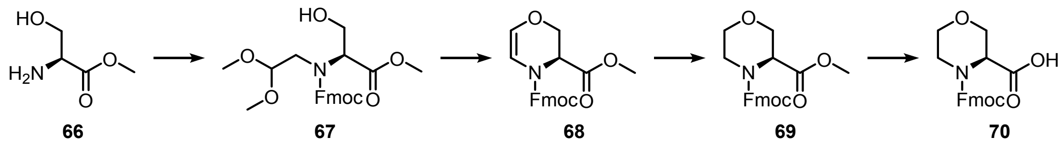

Scheme S10. Synthesis of compound **70**.

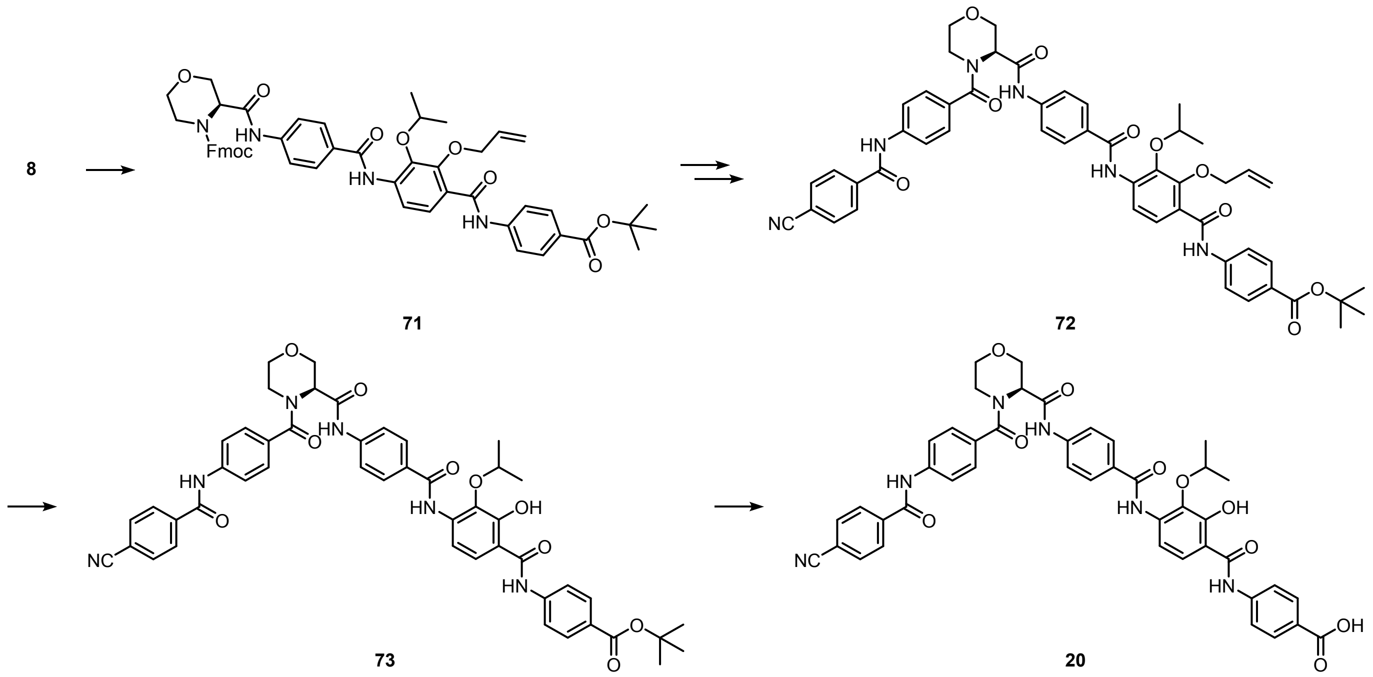

Scheme S11. Synthesis of compound **20**.

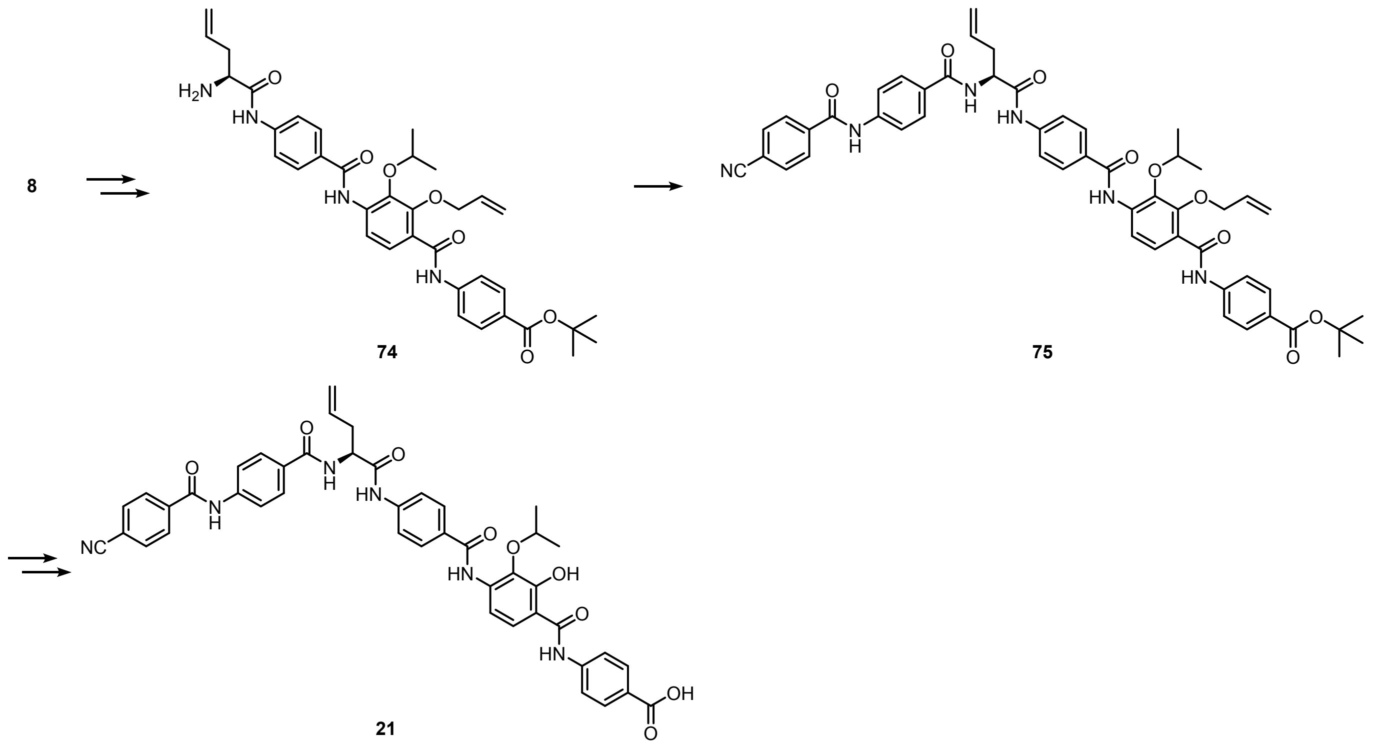

Scheme S12. Synthesis of compound **21**.

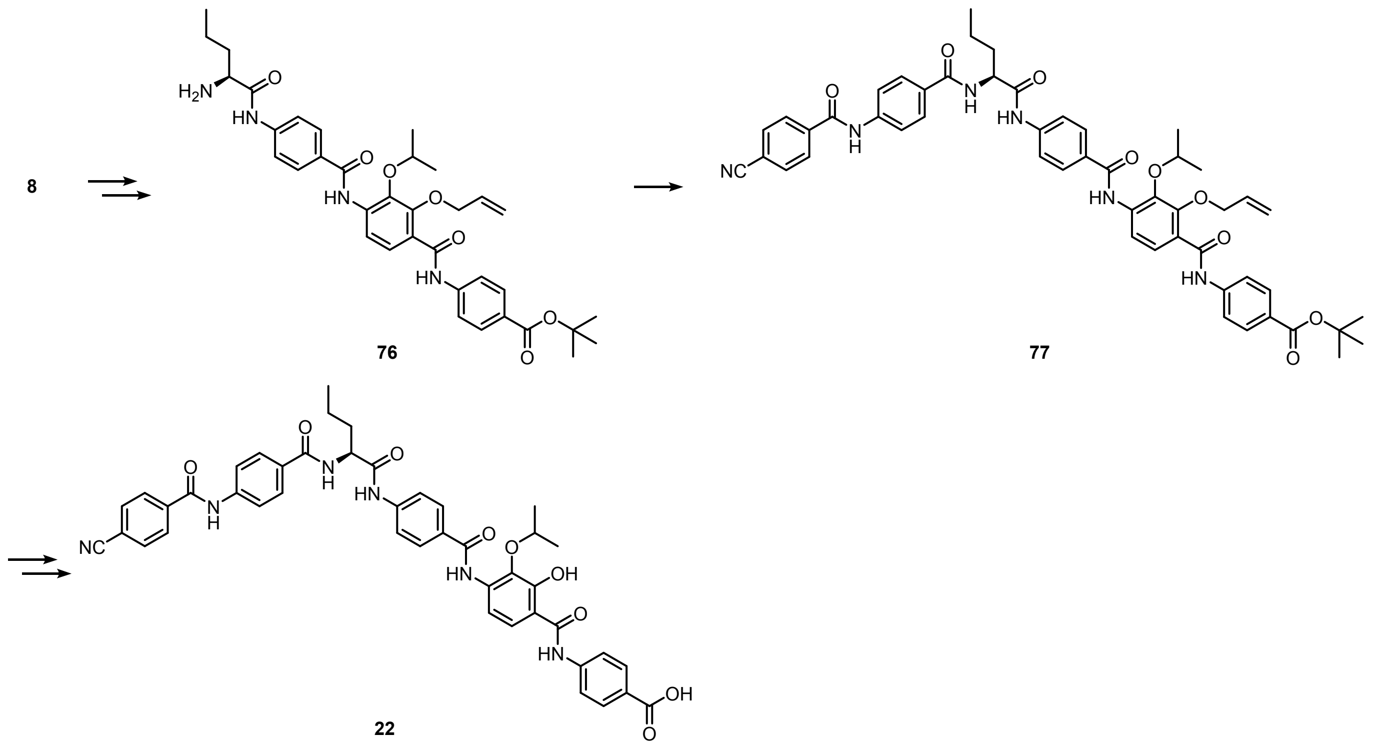

Scheme S13. Synthesis of compound **22**.

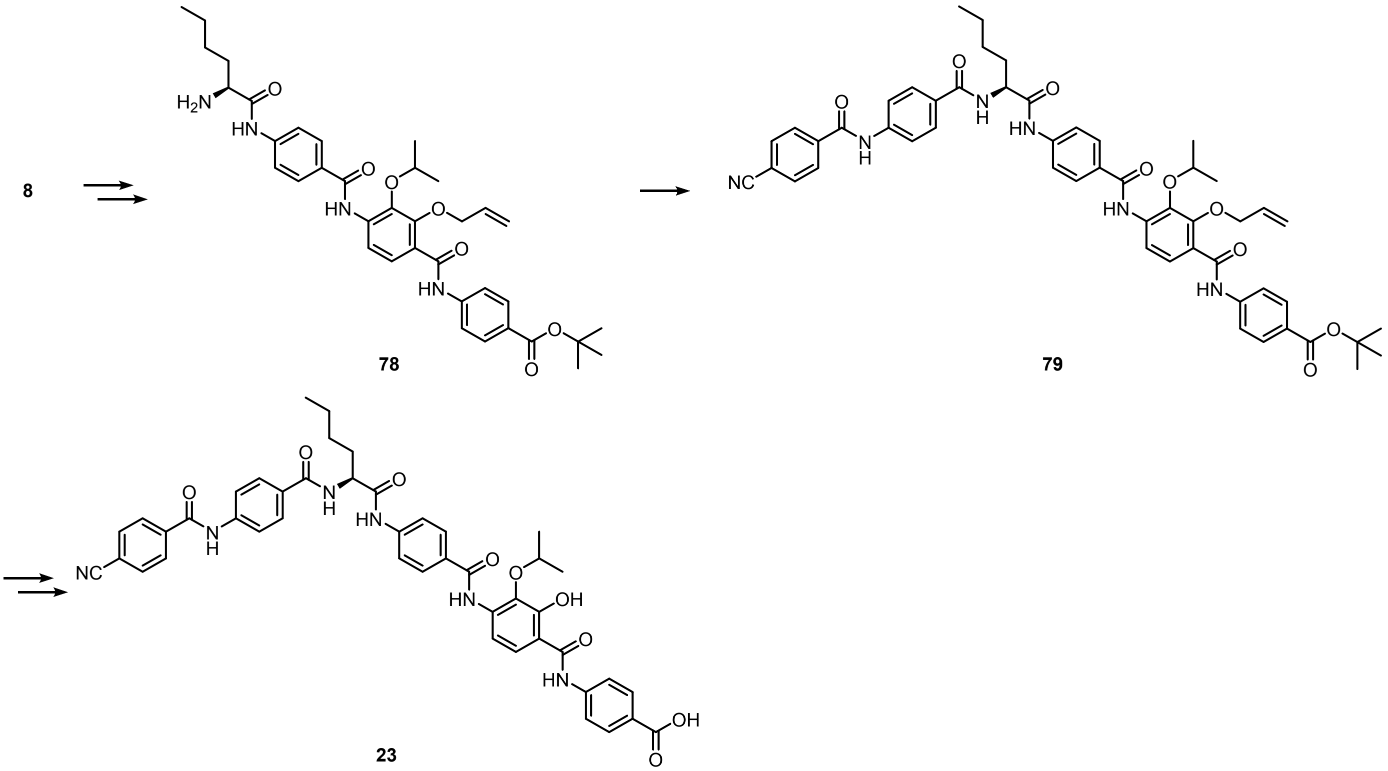

Scheme S14. Synthesis of compound **23**.

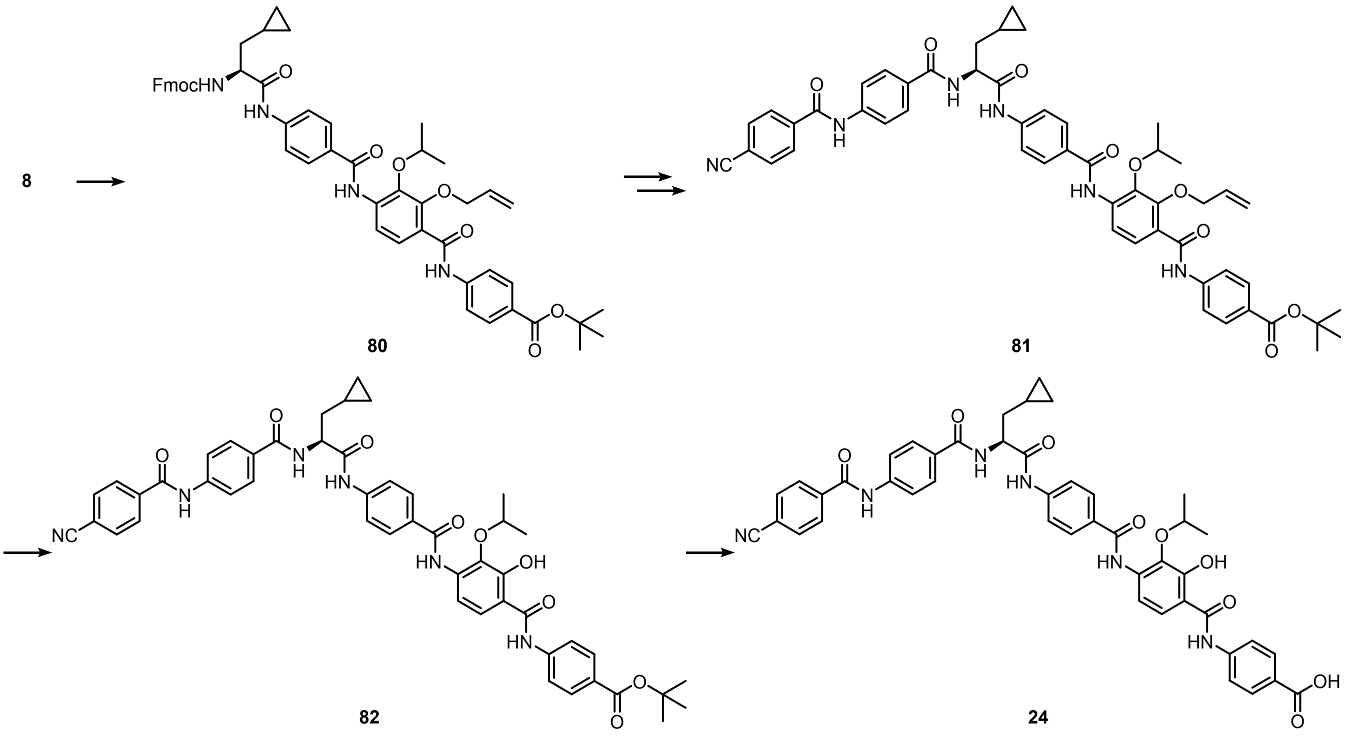

Scheme S15. Synthesis of compound **24**.

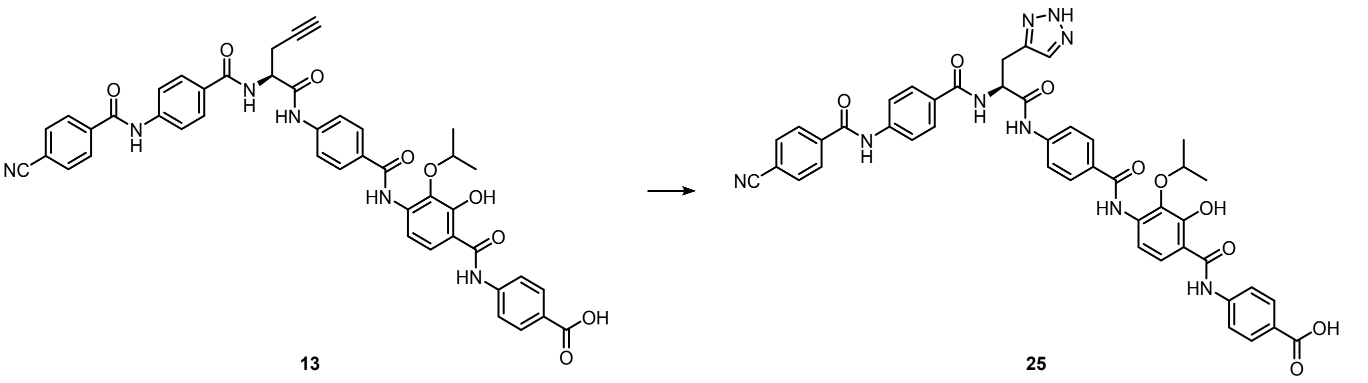

Scheme S16. Synthesis of compound **25**.

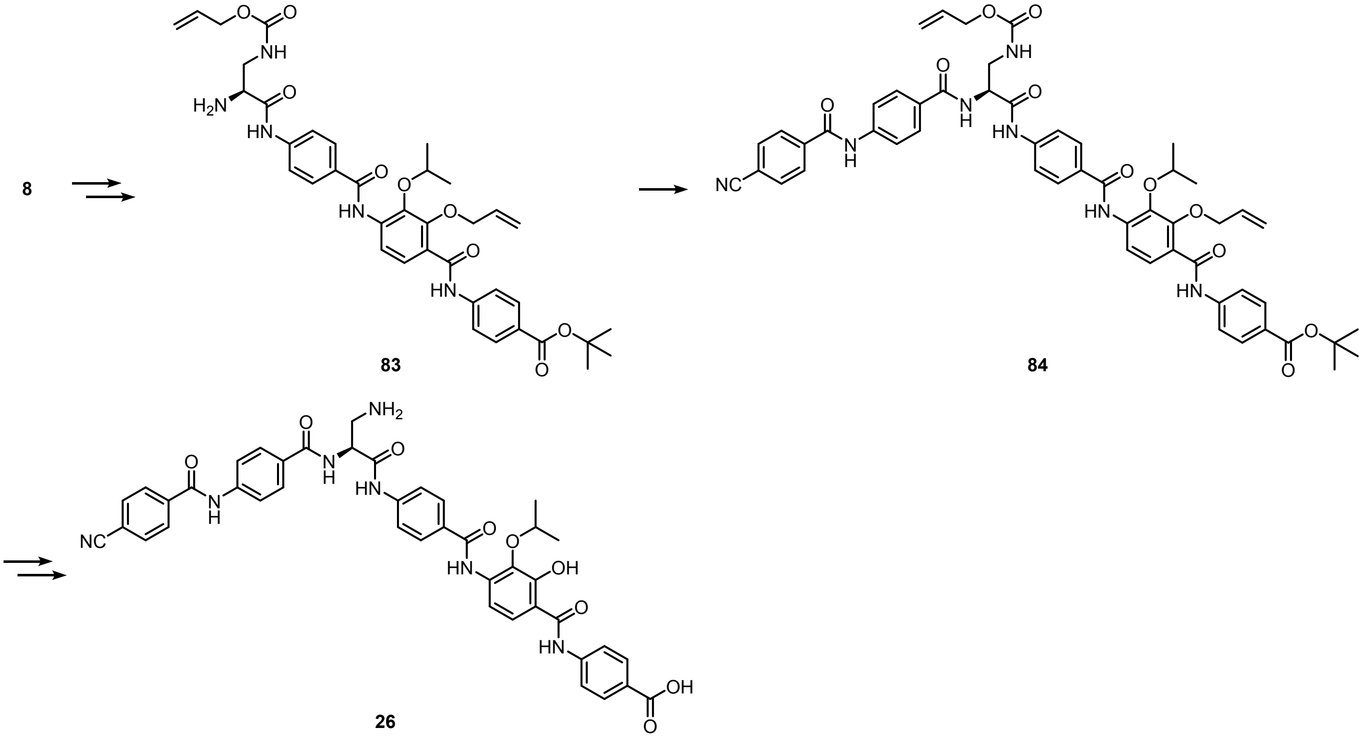

Scheme S17. Synthesis of compound **26**.

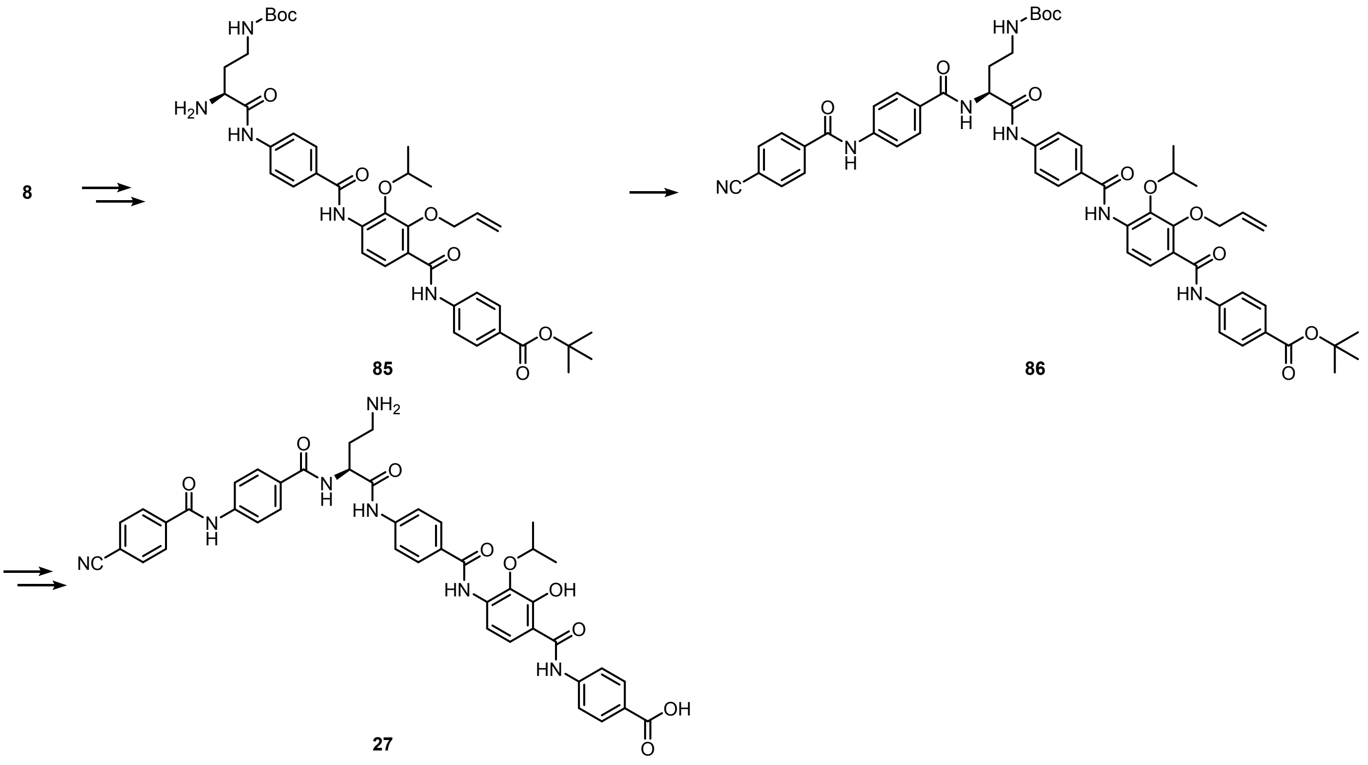

Scheme S18. Synthesis of compound **27**.

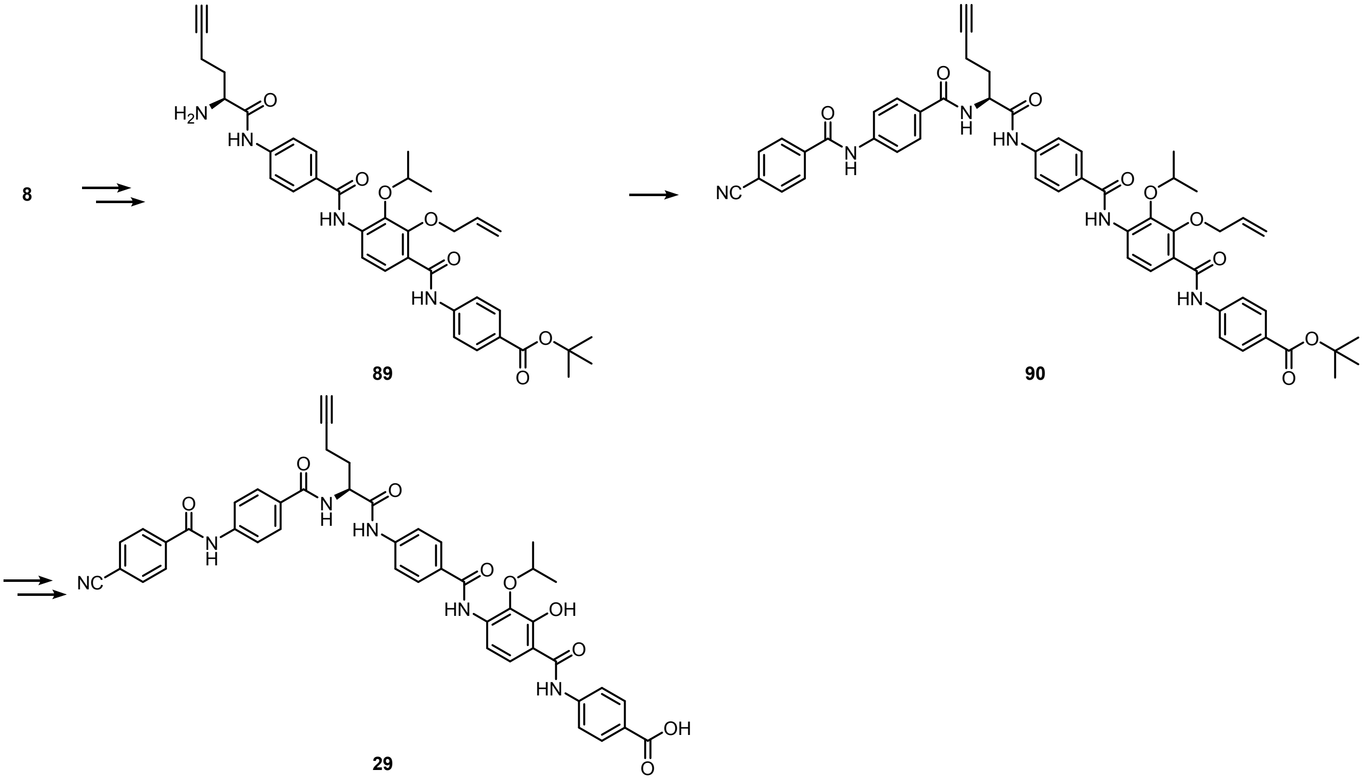

Scheme S19. Synthesis of compound **29**.

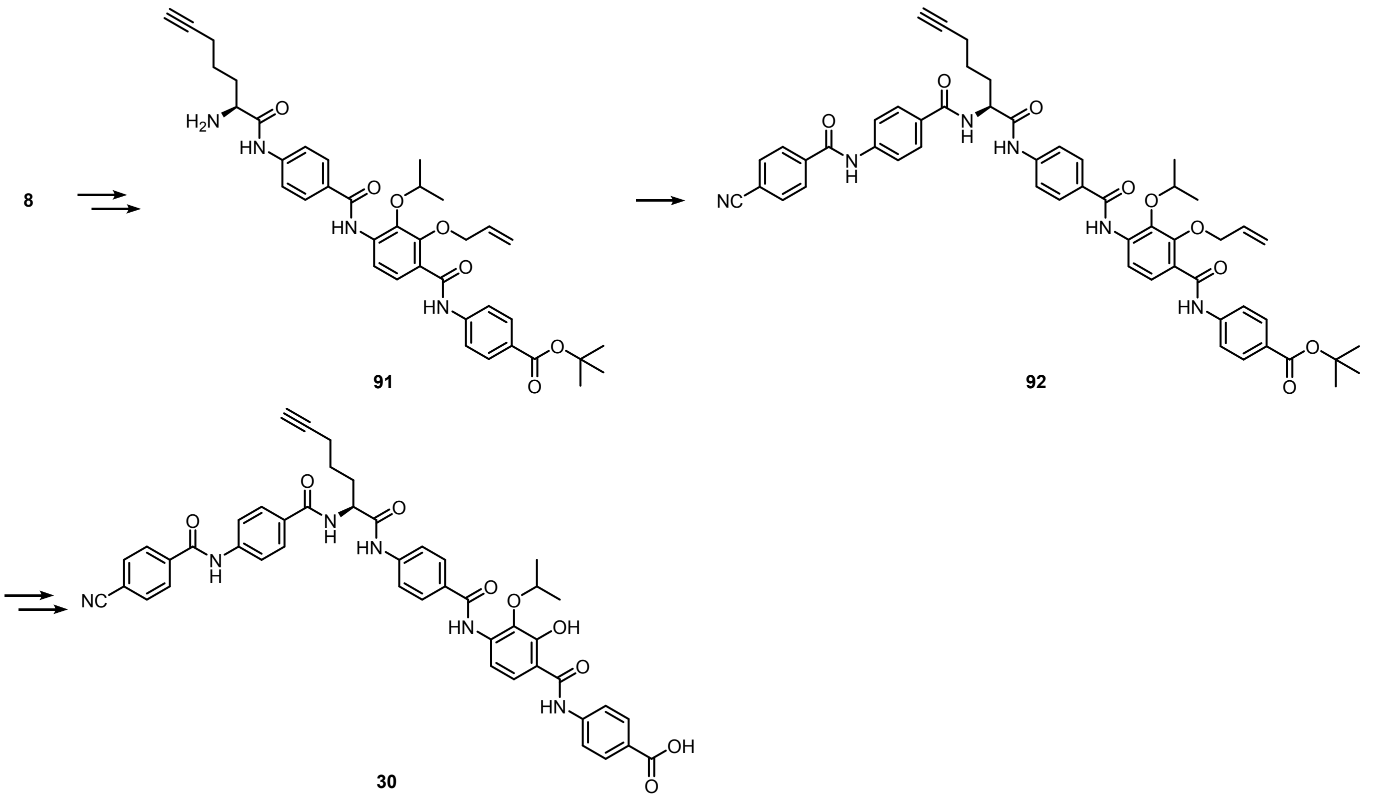

Scheme S20. Synthesis of compound **30**.

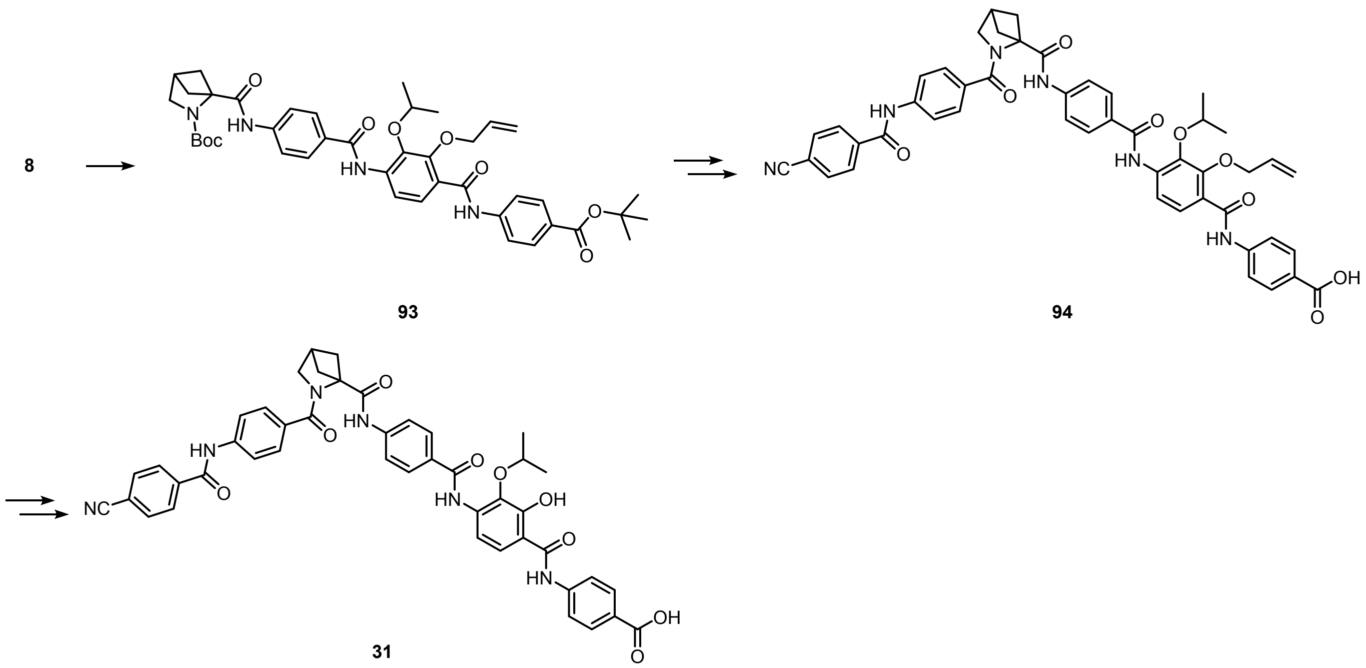

Scheme S21. Synthesis of compound **31**.

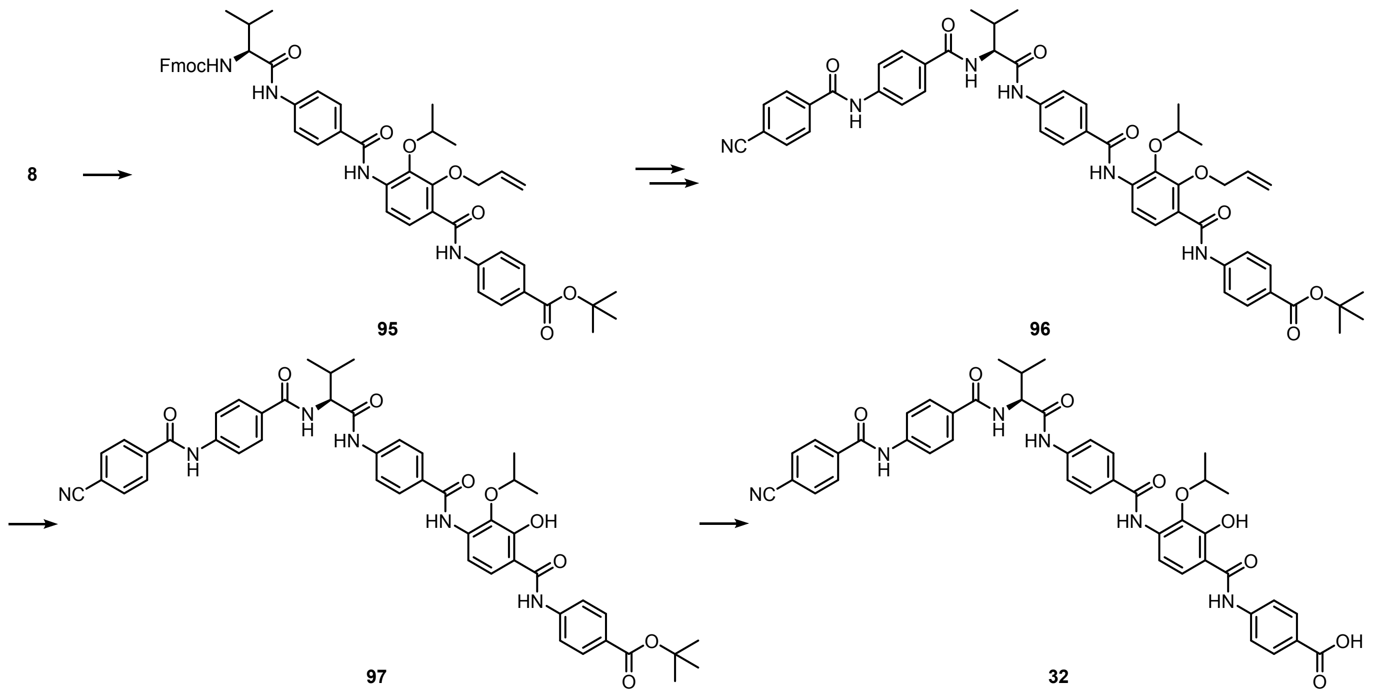

Scheme S22. Synthesis of compound **32**.

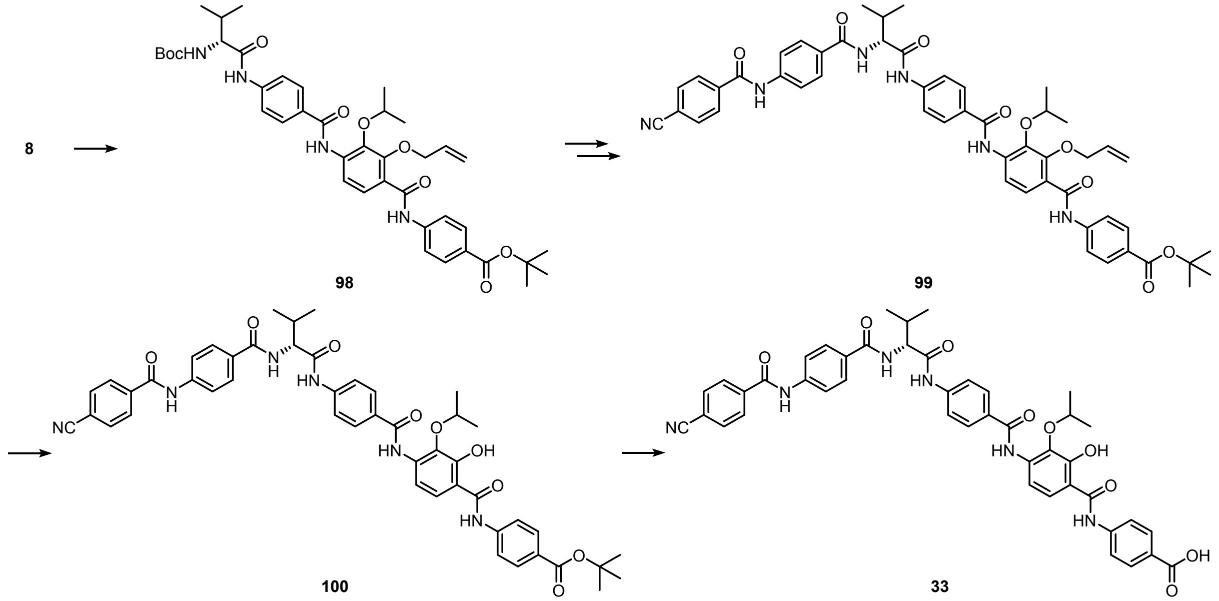

Scheme S23. Synthesis of compound **33**.

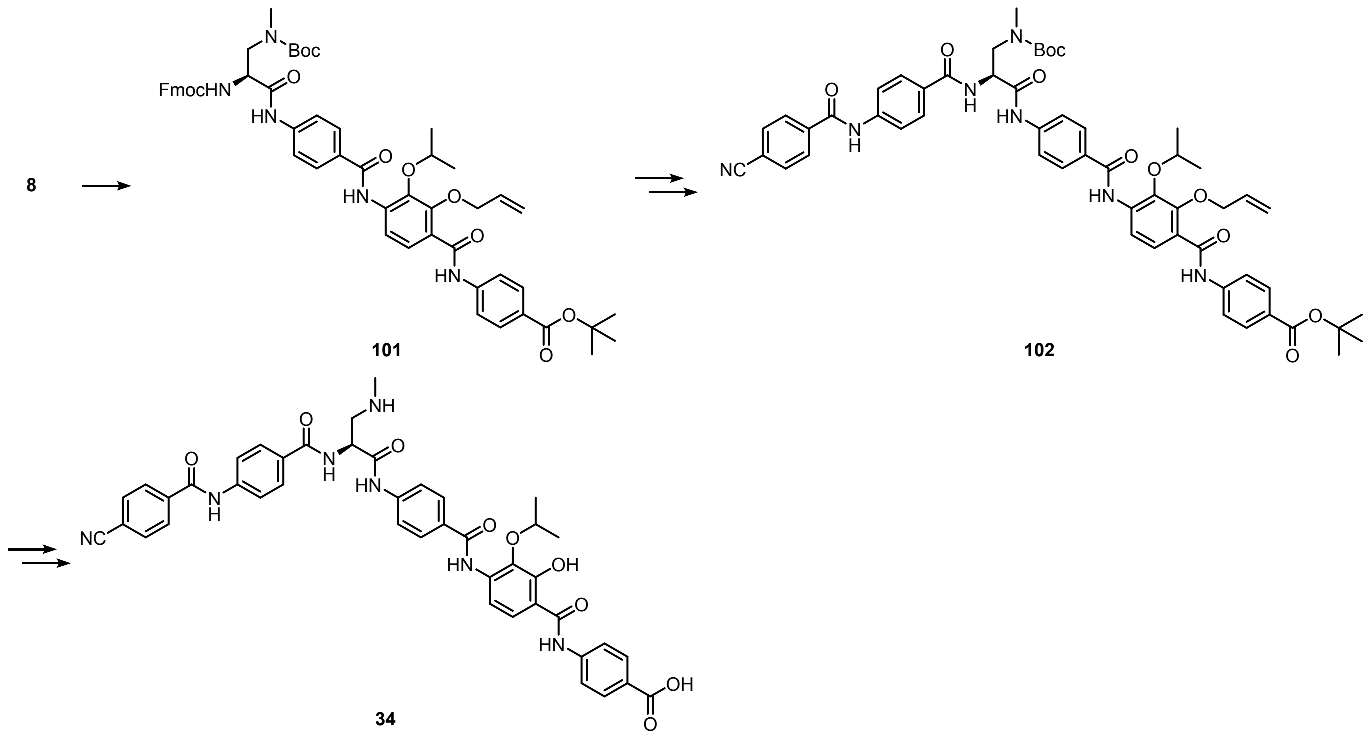

Scheme S24. Synthesis of compound **34**.

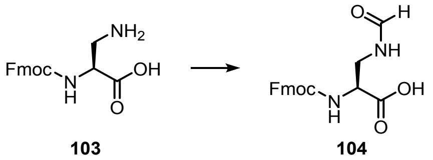

Scheme S25. Synthesis of compound **104**.

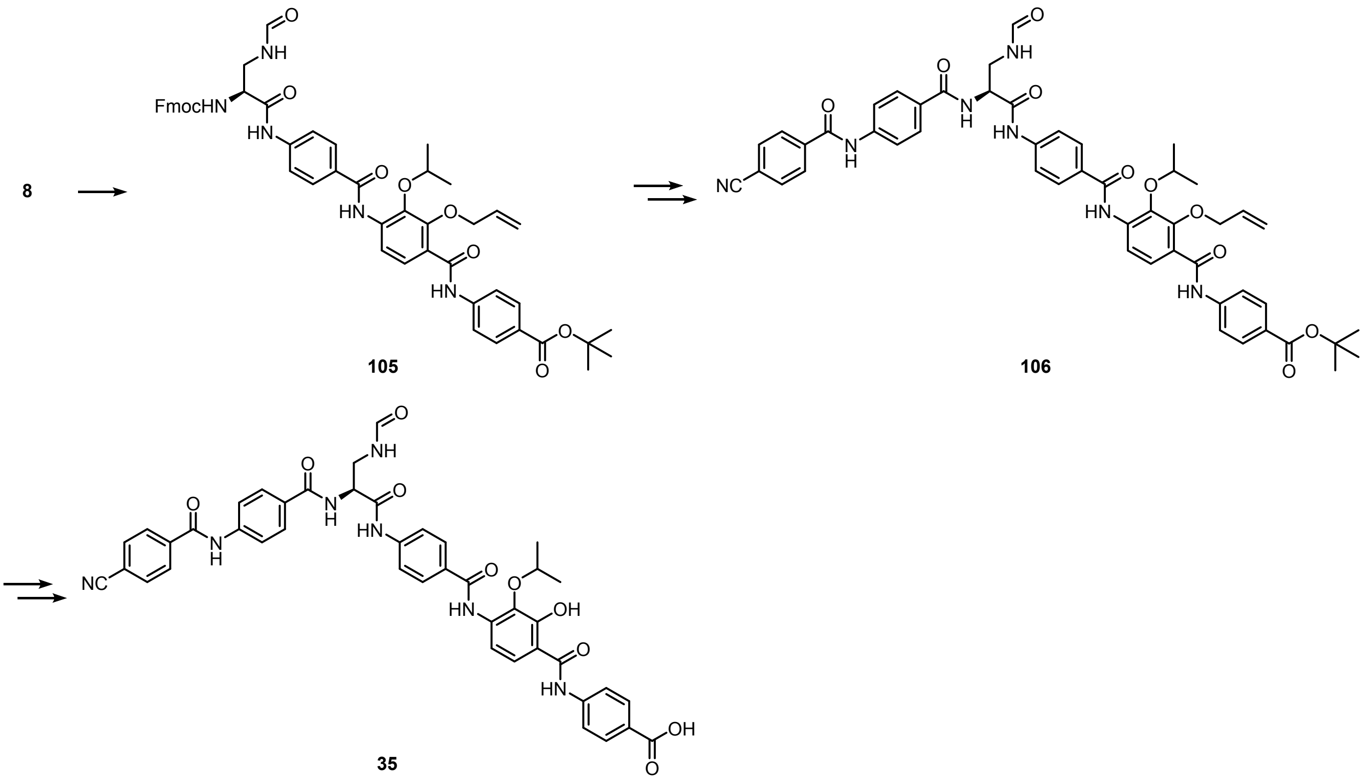

Scheme S26. Synthesis of compound **35**.

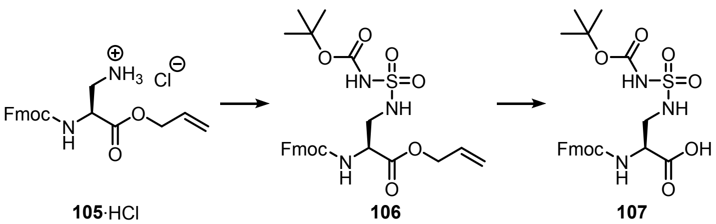

Scheme S27. Synthesis of compound **107**.

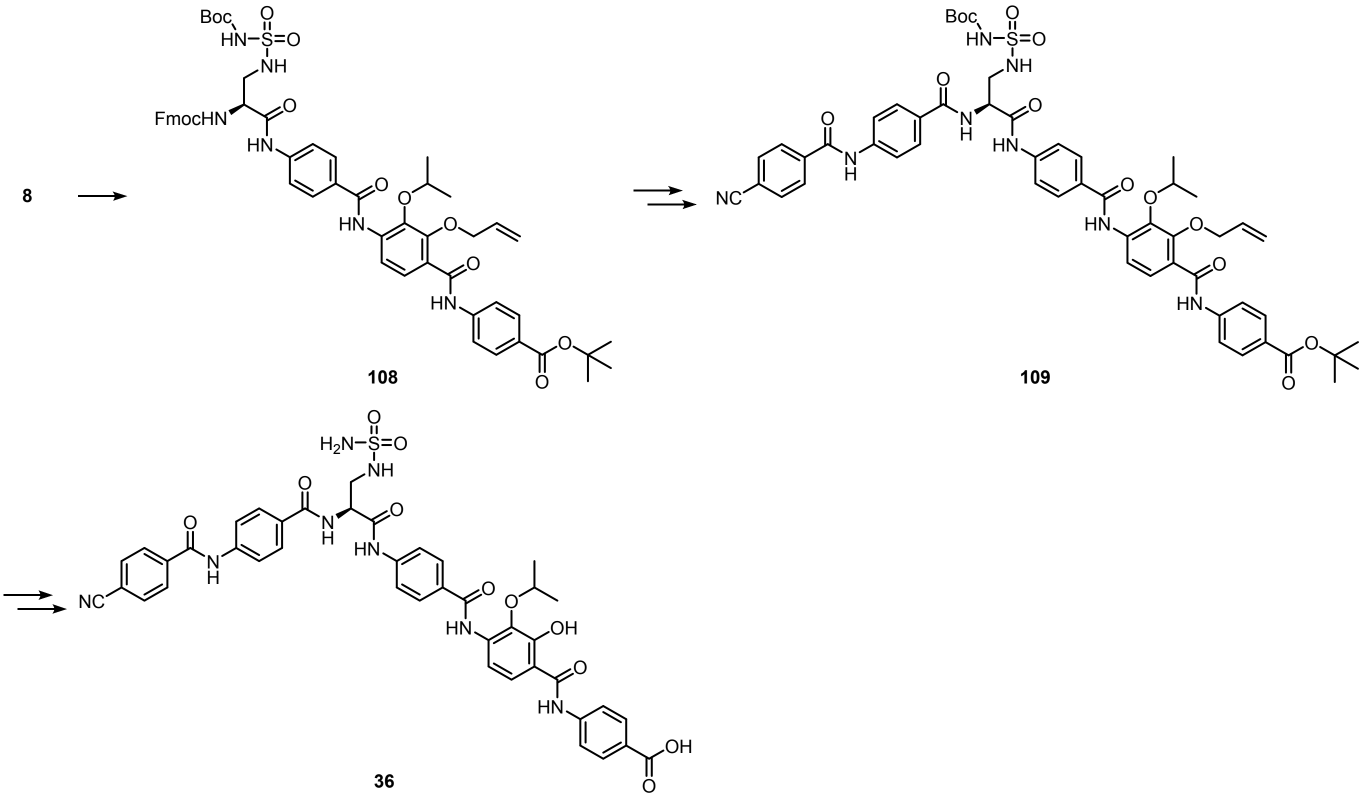

Scheme S28. Synthesis of compound **36**.

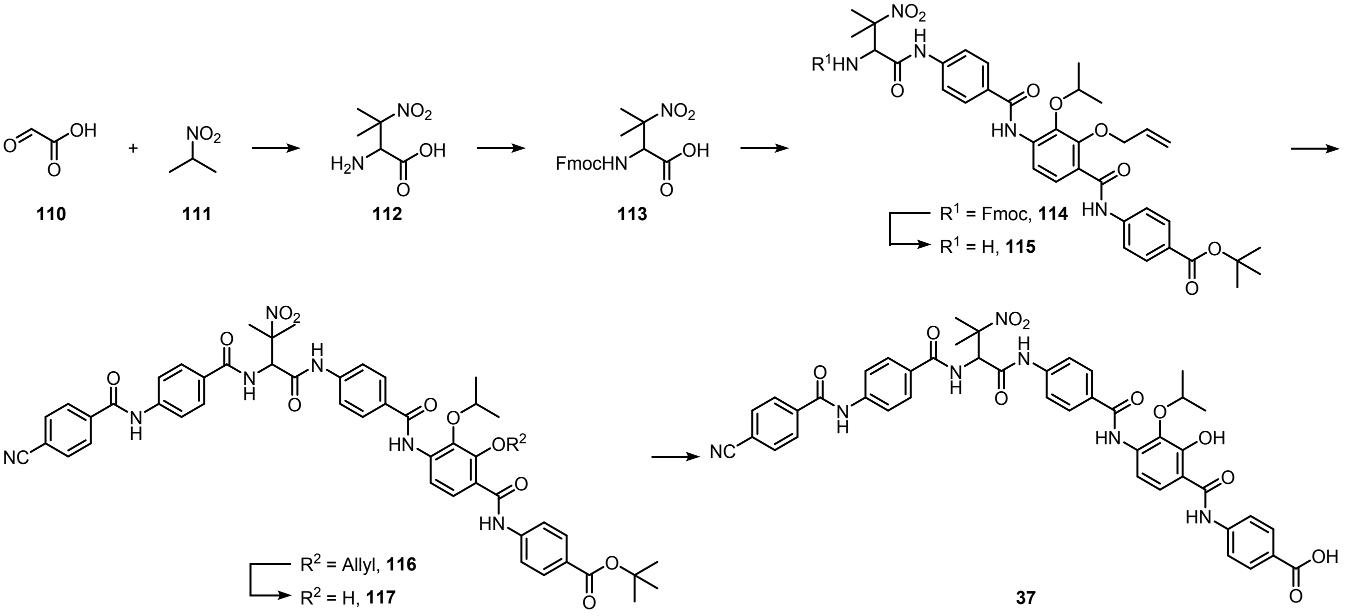

Scheme S29. Synthesis of compound **37**.

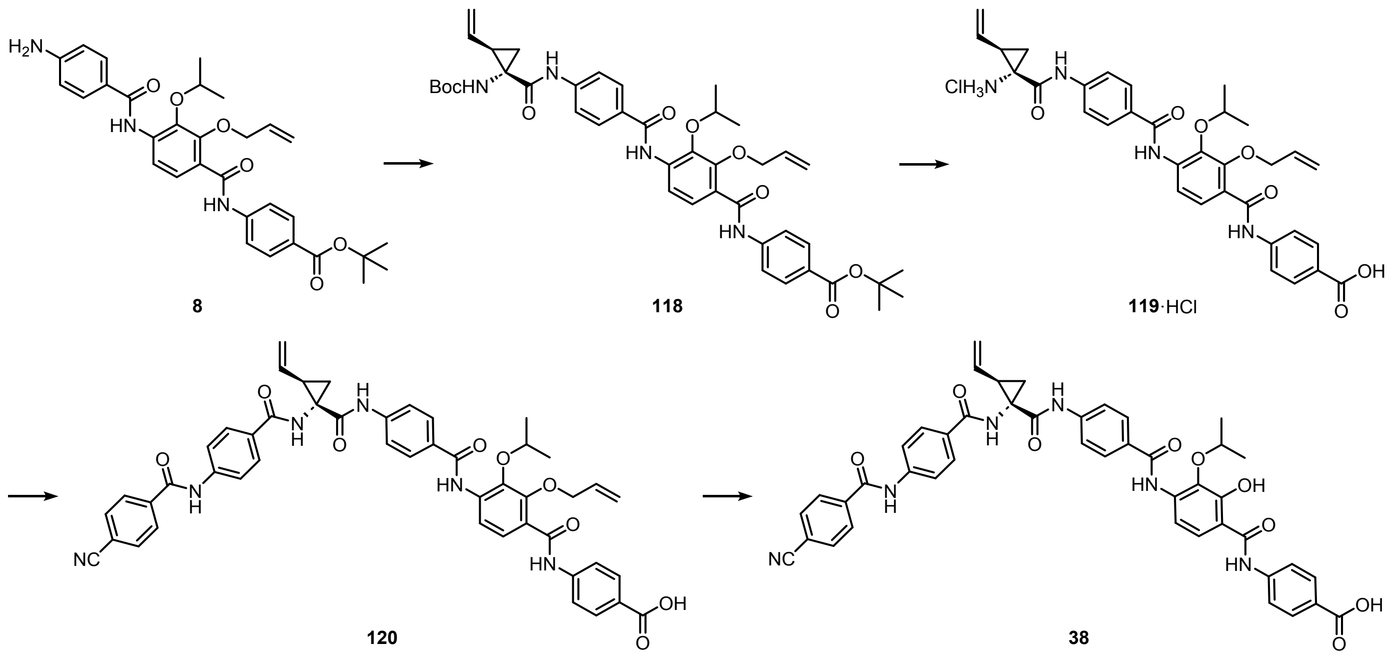

Scheme S30. Synthesis of compound **38**.

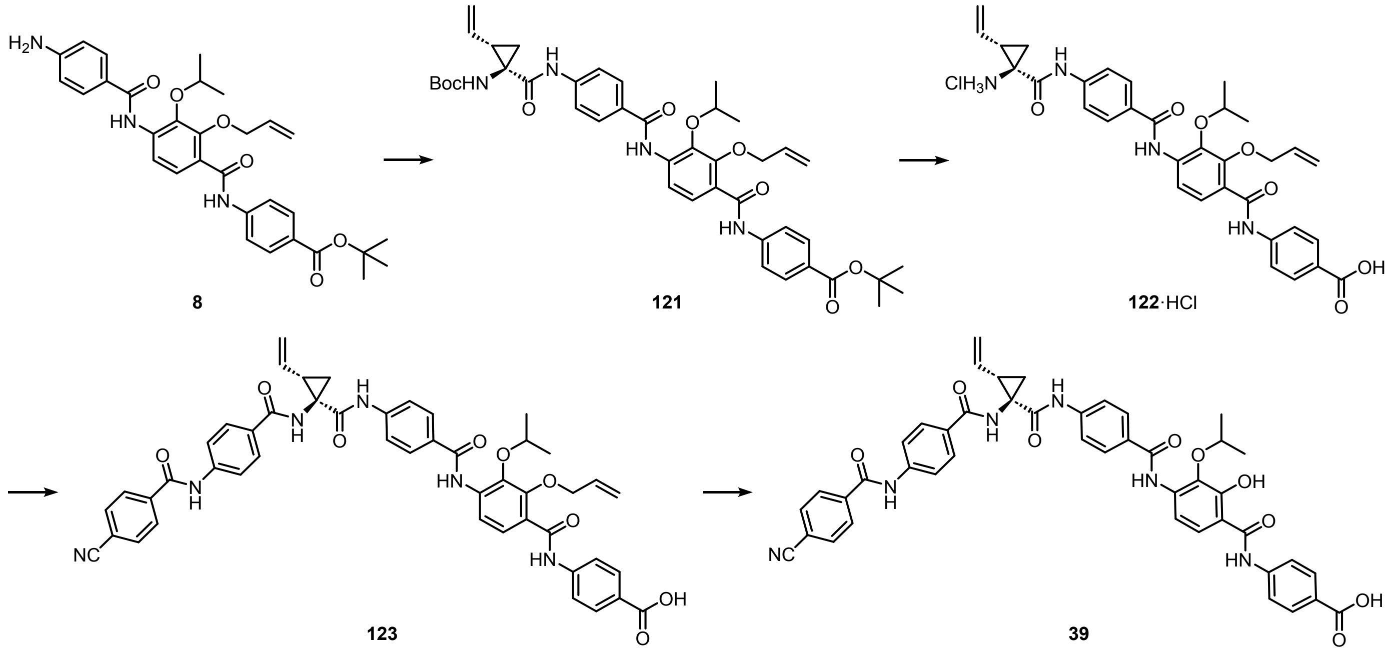

Scheme S31. Synthesis of compound **39**.

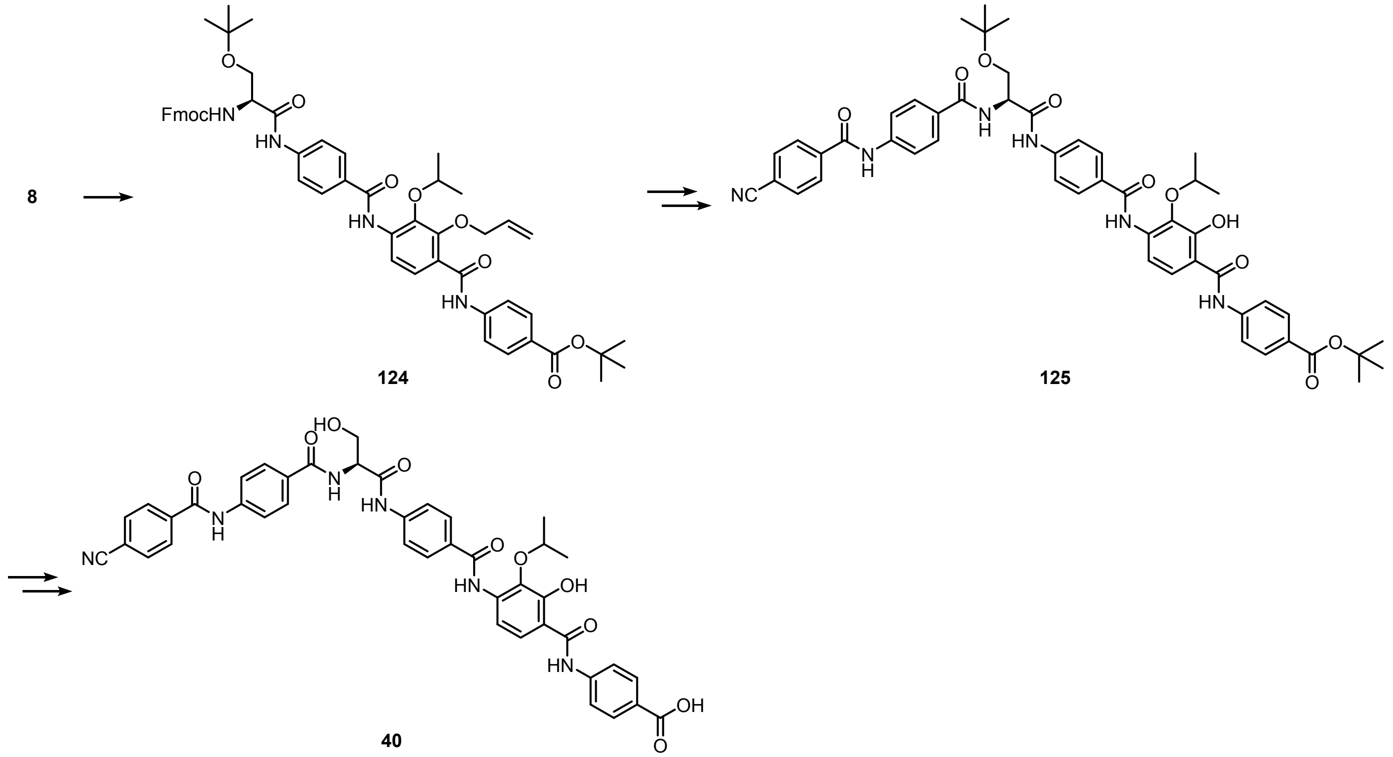

Scheme S32. Synthesis of compound **40**.

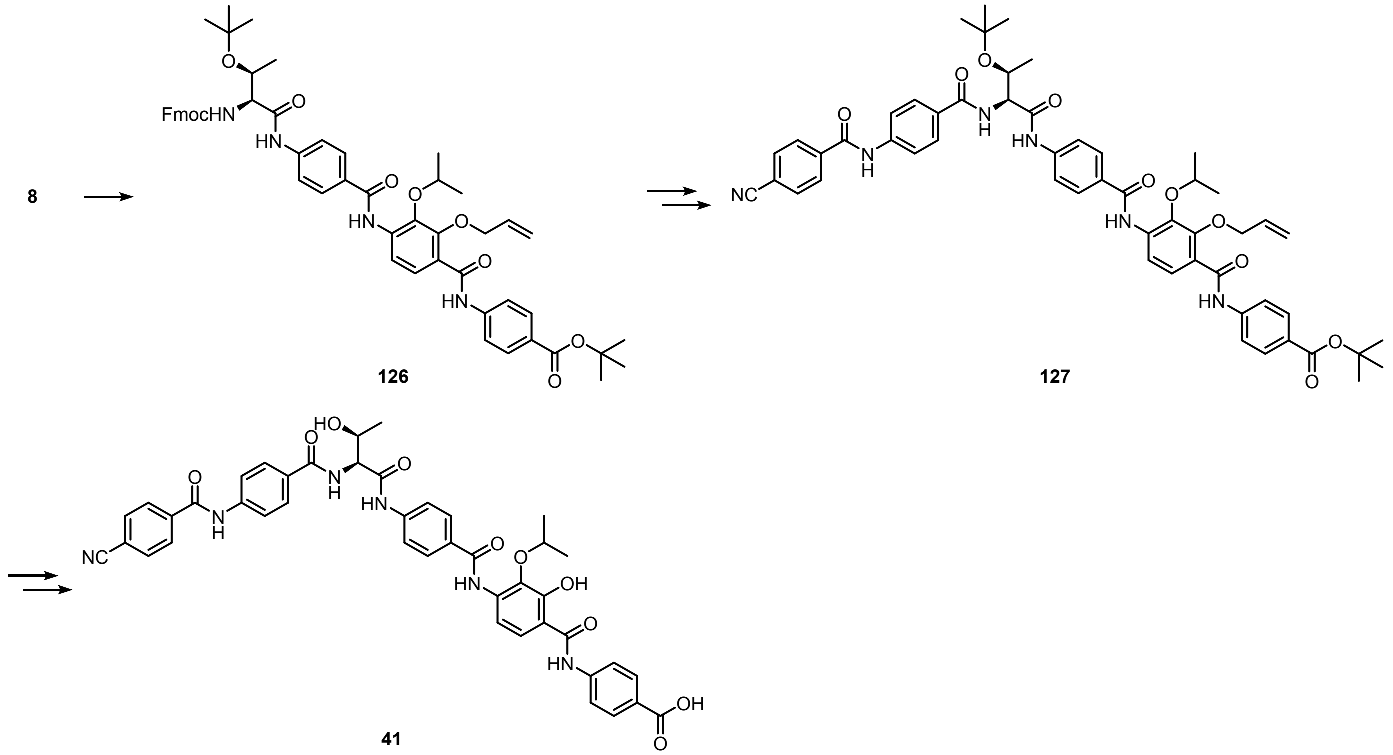

Scheme S33. Synthesis of compound **41**.

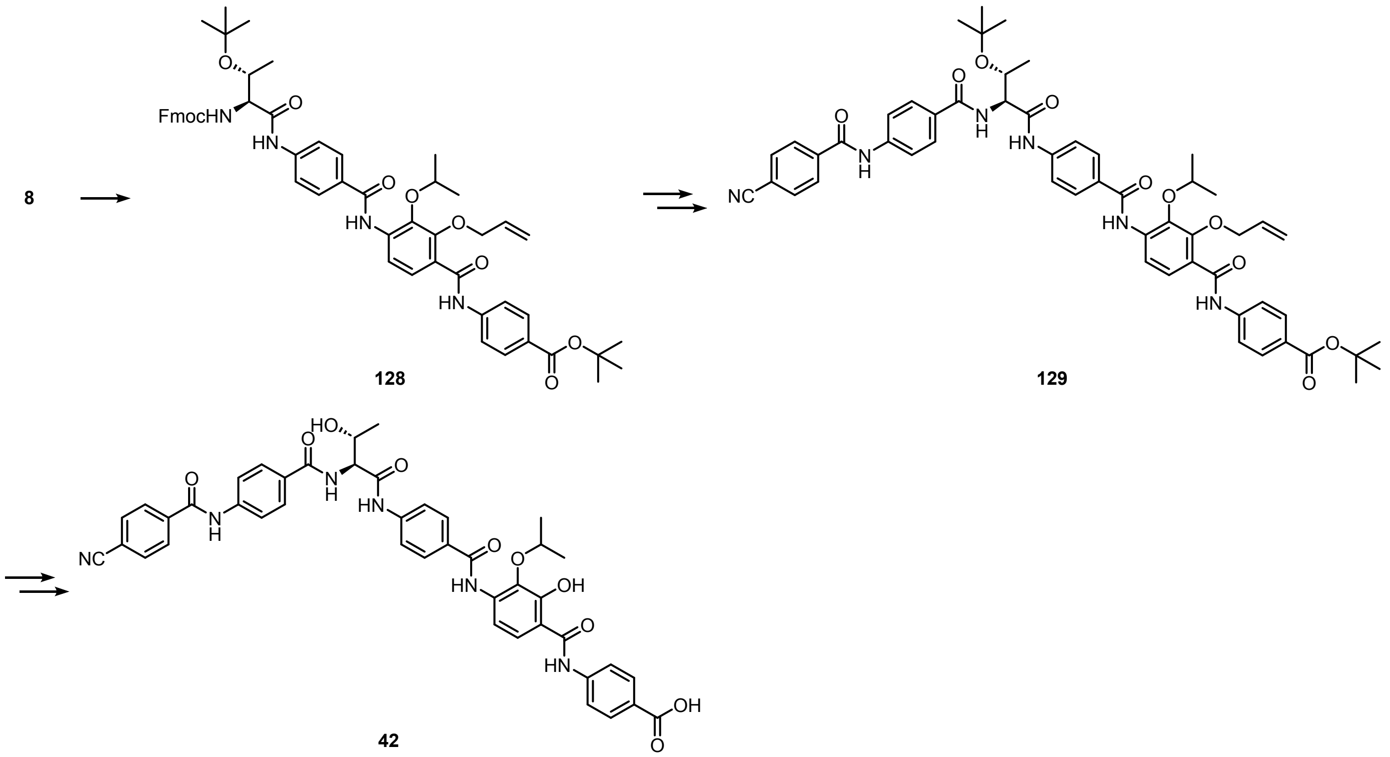

Scheme S34. Synthesis of compound **42**.

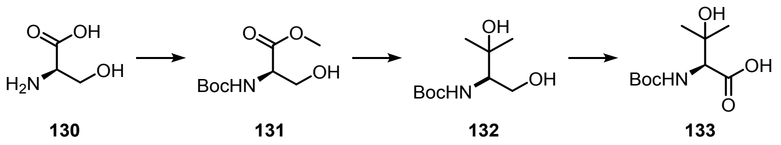

Scheme S35. Synthesis of compound **133**.

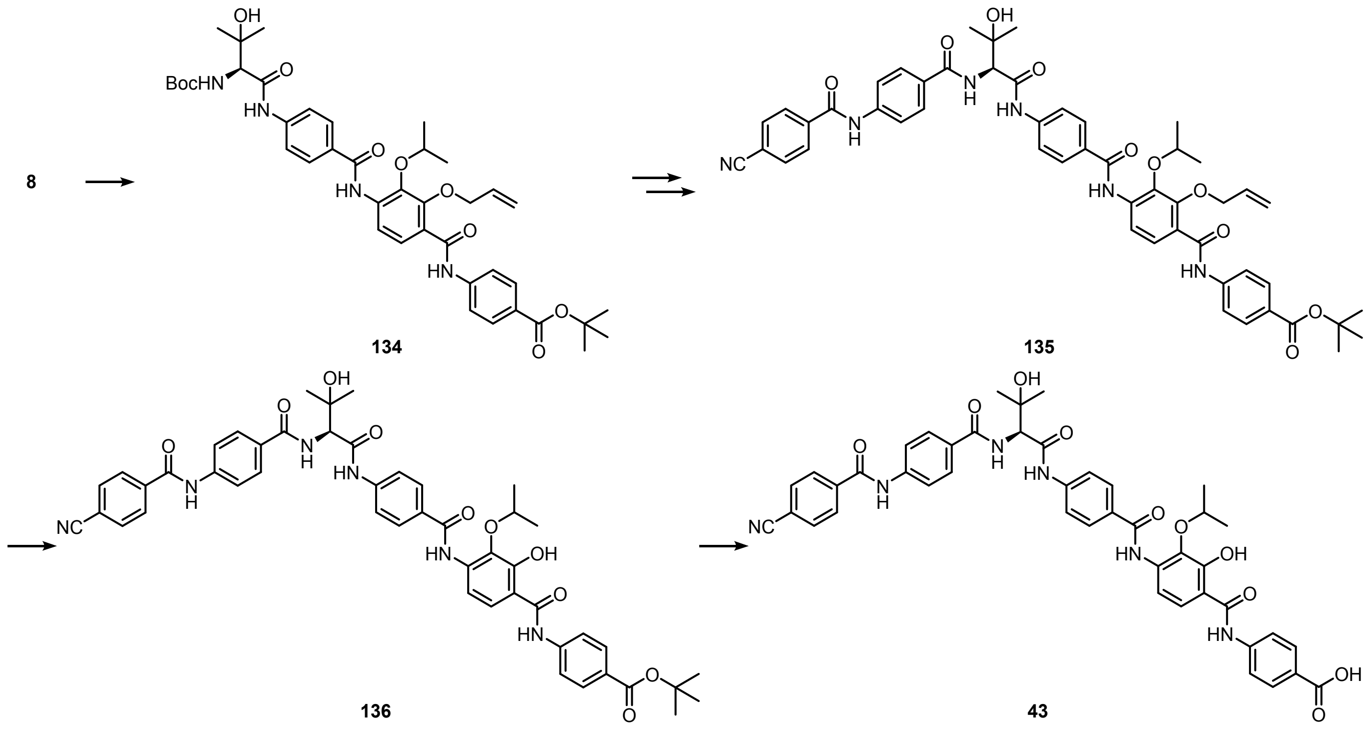

Scheme S36. Synthesis of compound **43**.

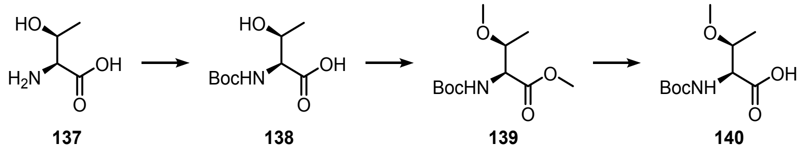

Scheme S37. Synthesis of compound **140**.

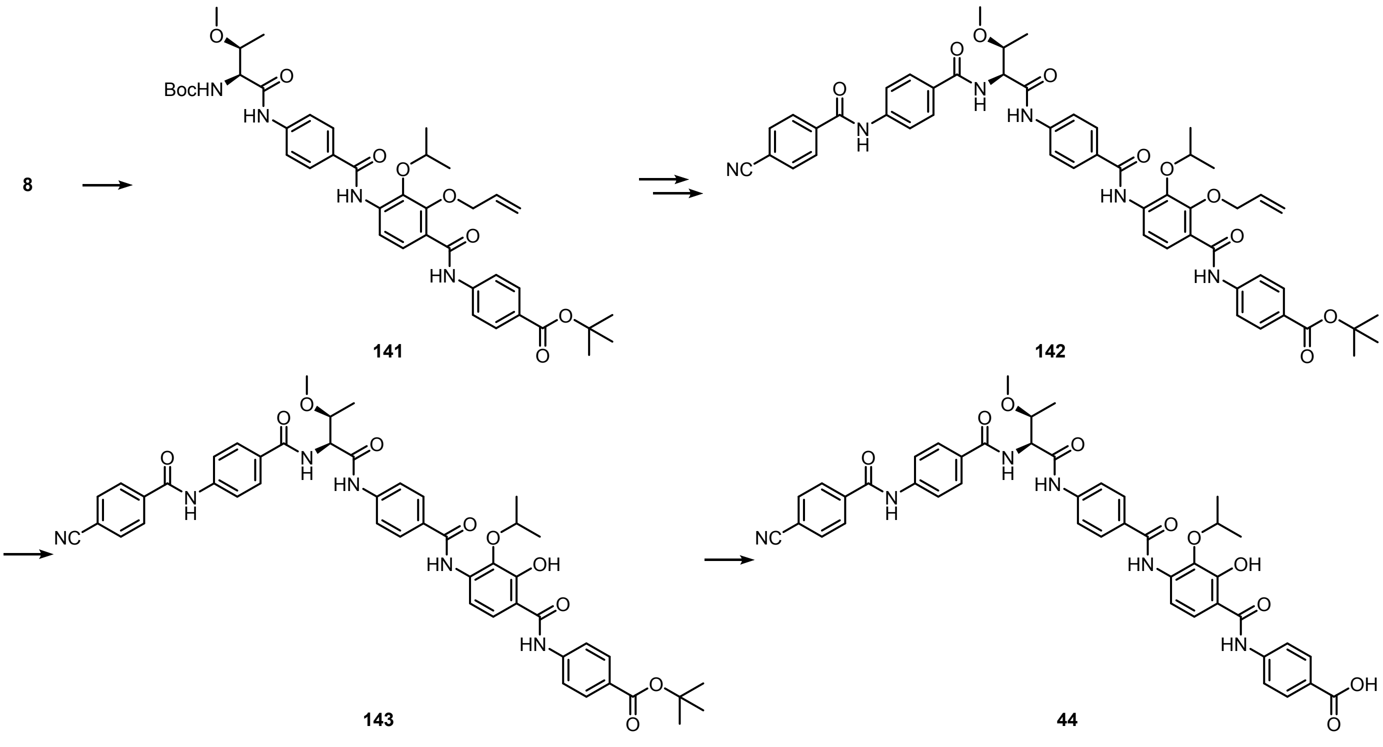

Scheme S38. Synthesis of compound **44**.

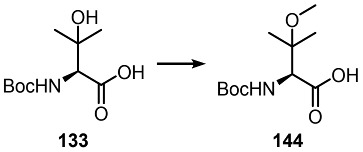

Scheme S39. Synthesis of compound **144**.

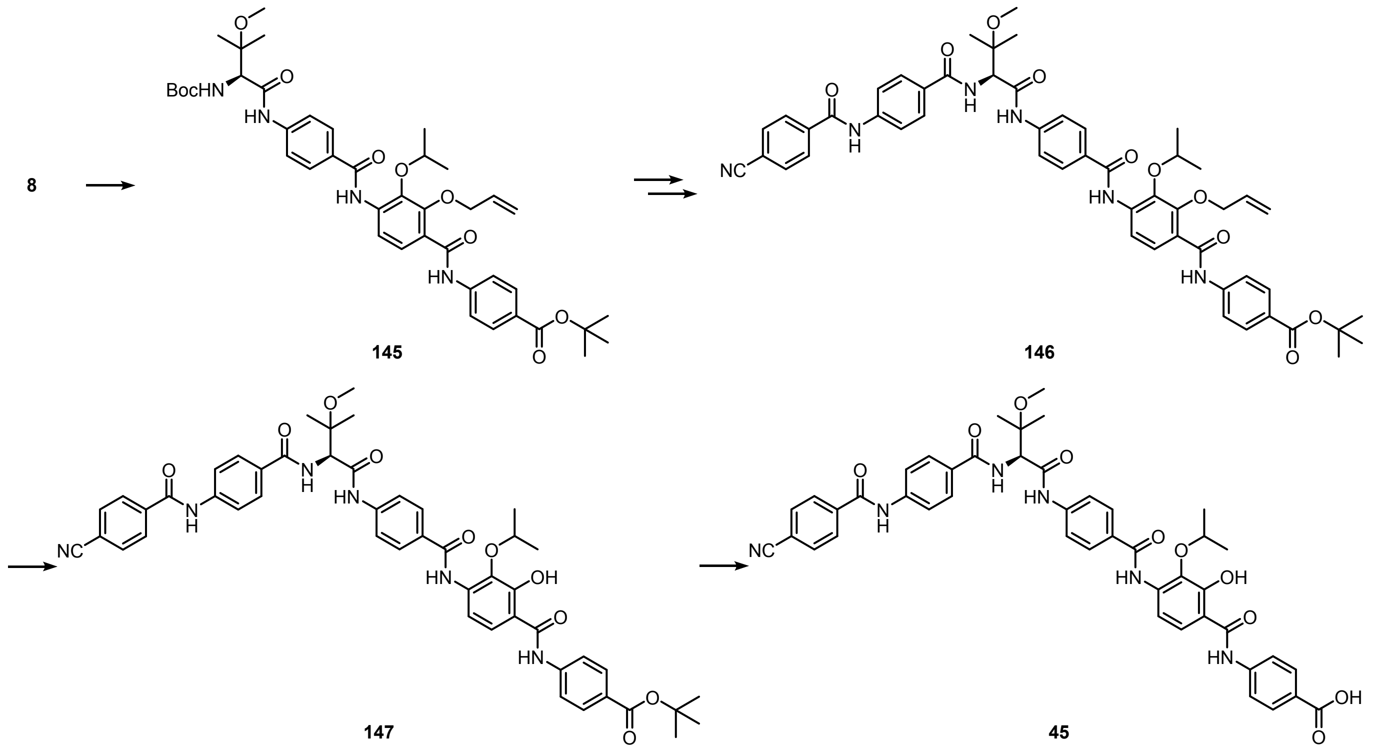

Scheme S40. Synthesis of compound **45**.

NMR Spectra of Final Compounds

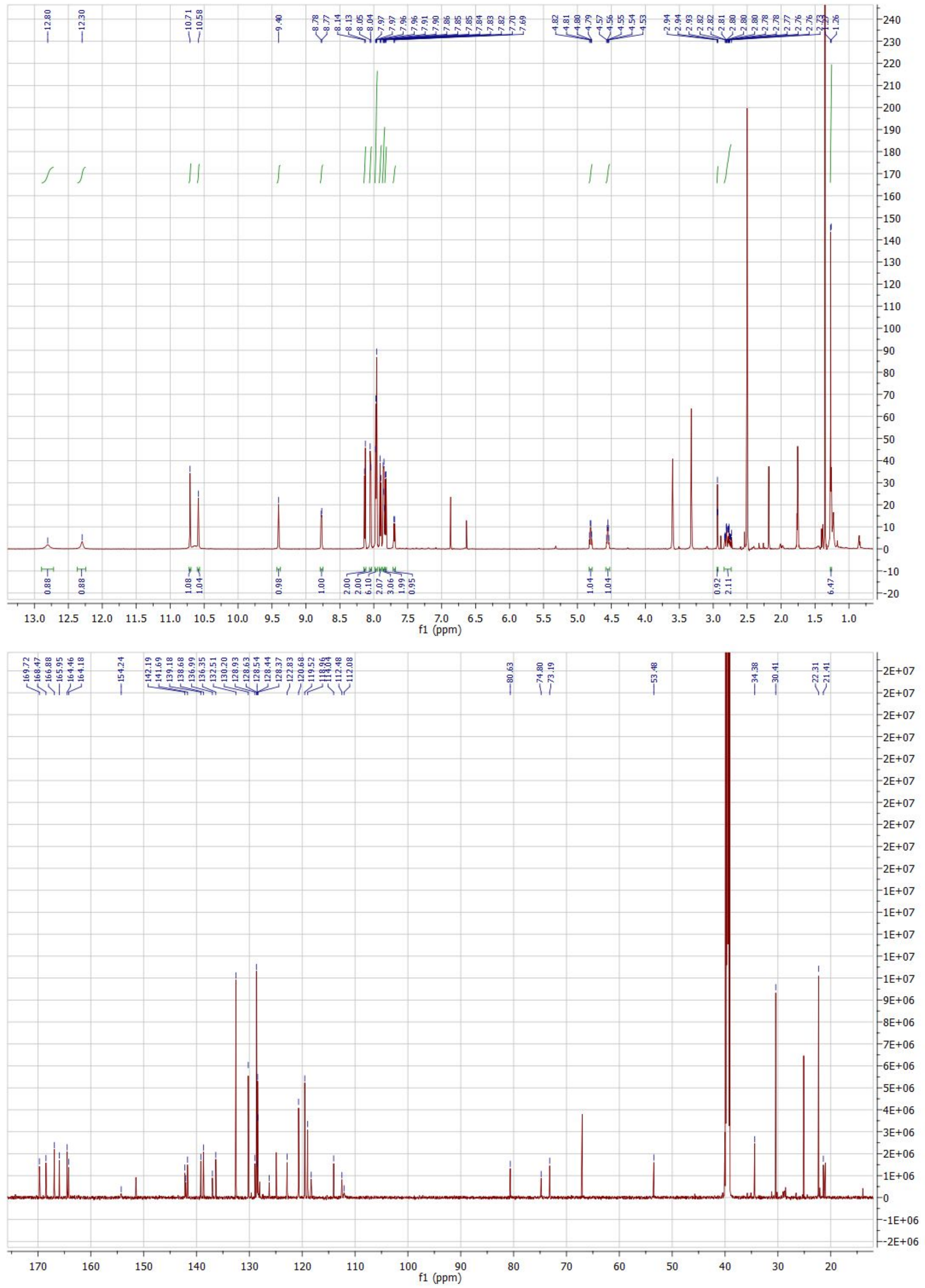

Figure S3. ^{1}H- (top) and ^{13}C-NMR (bottom) spectrum of compound **13** (**CN-CC-861**).

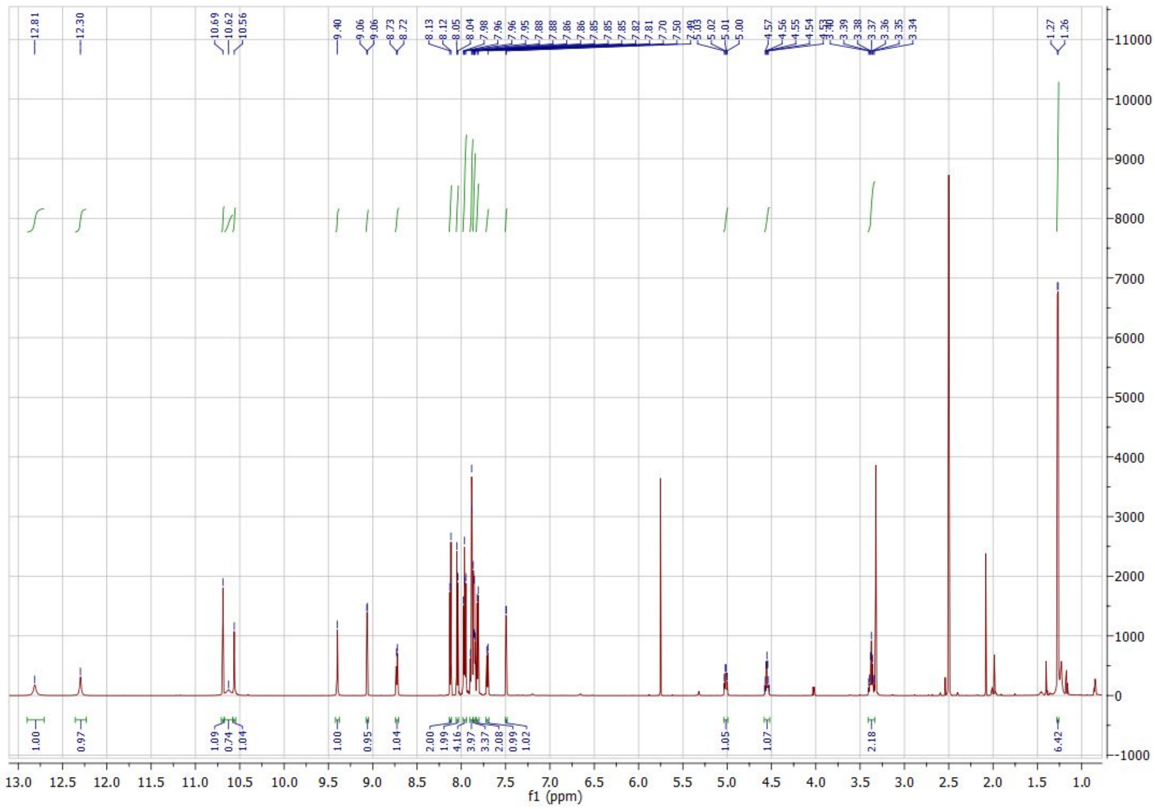

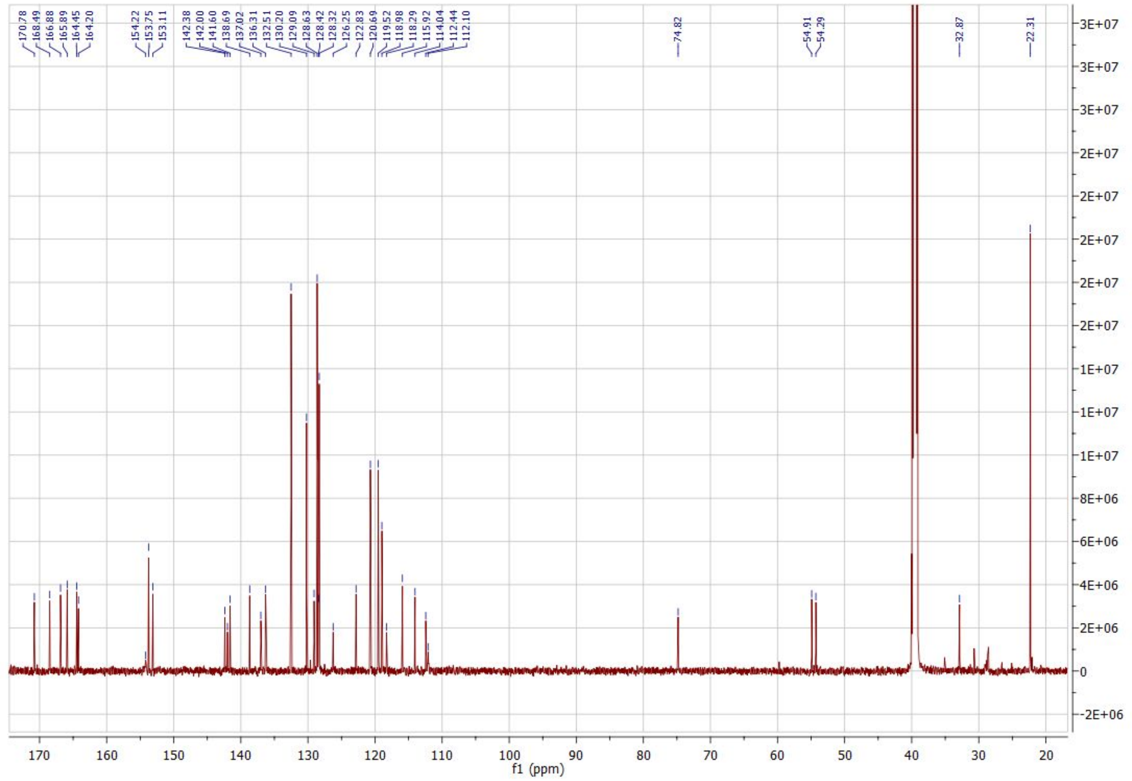

Figure S4. ^{1}H- (top) and ^{13}C-NMR (bottom) spectrum of compound **14**.

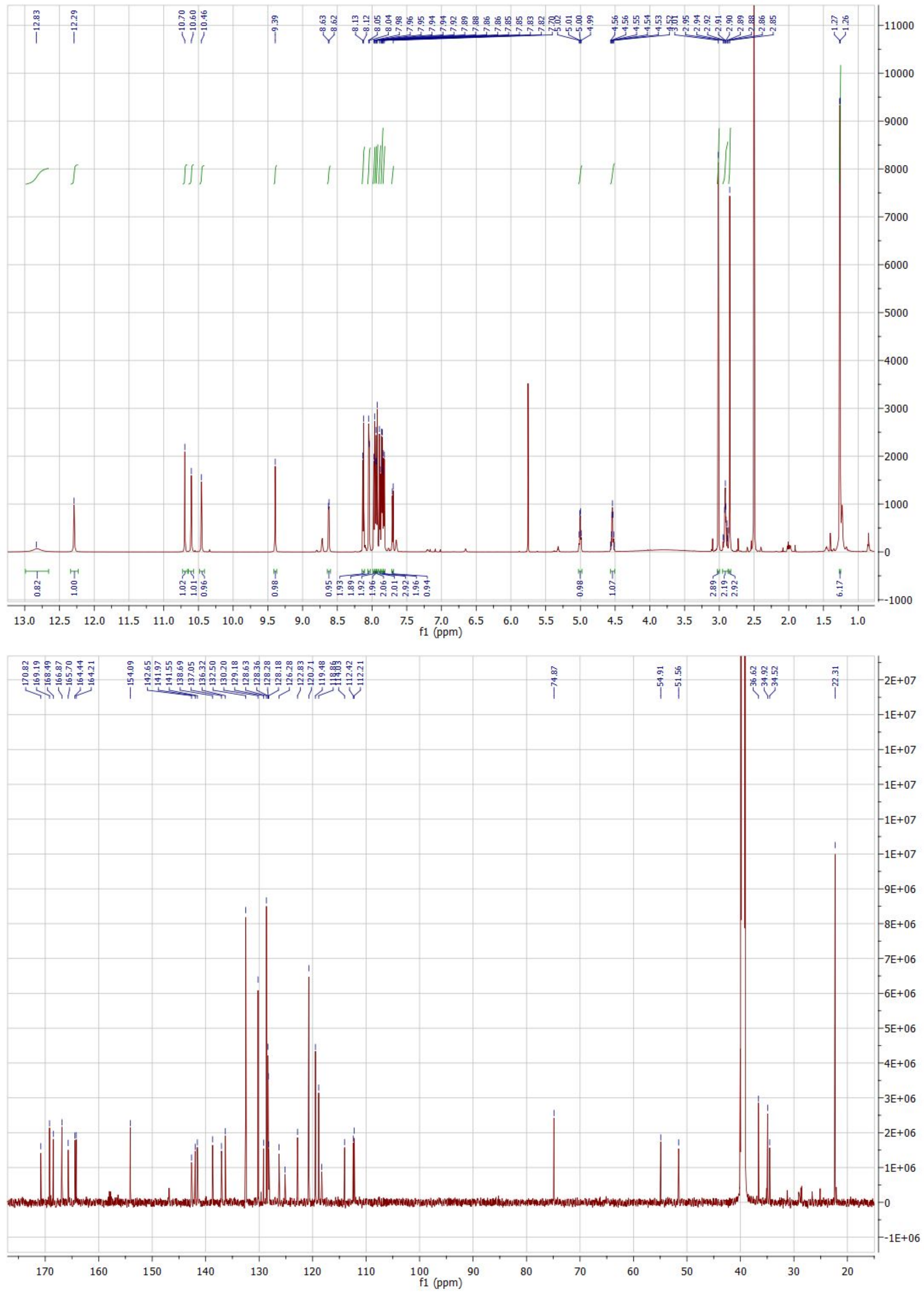

Figure S5. ^{1}H- (top) and ^{13}C-NMR (bottom) spectrum of compound **15**.

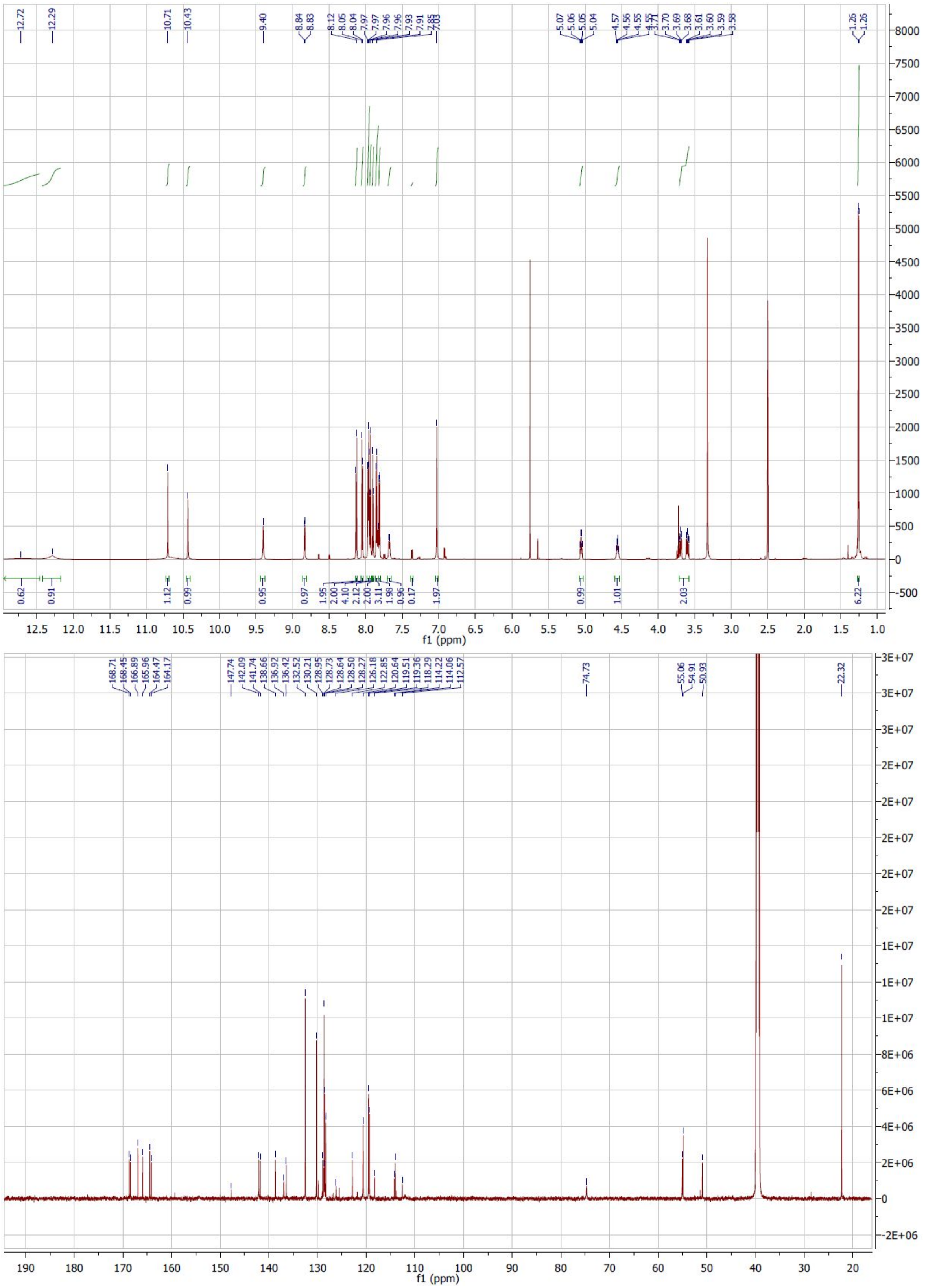

Figure S6. ^{1}H- (top) and ^{13}C-NMR (bottom) spectrum of compound **16**.

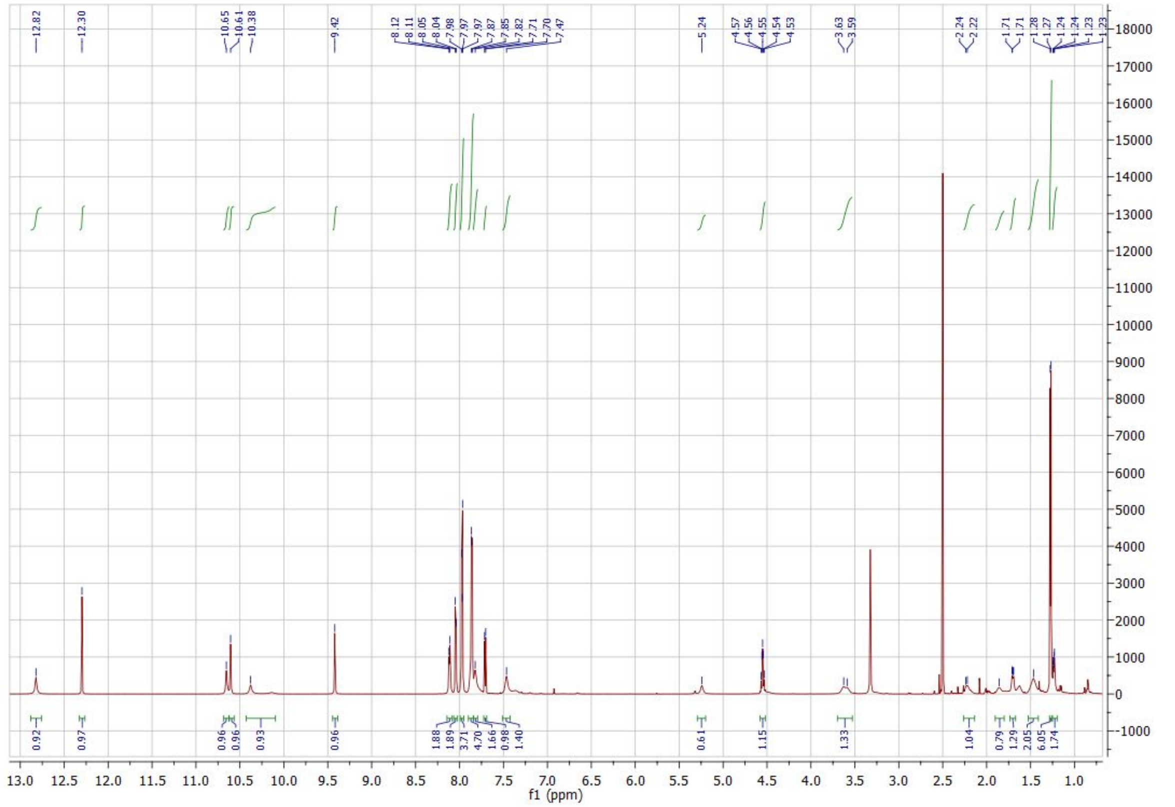

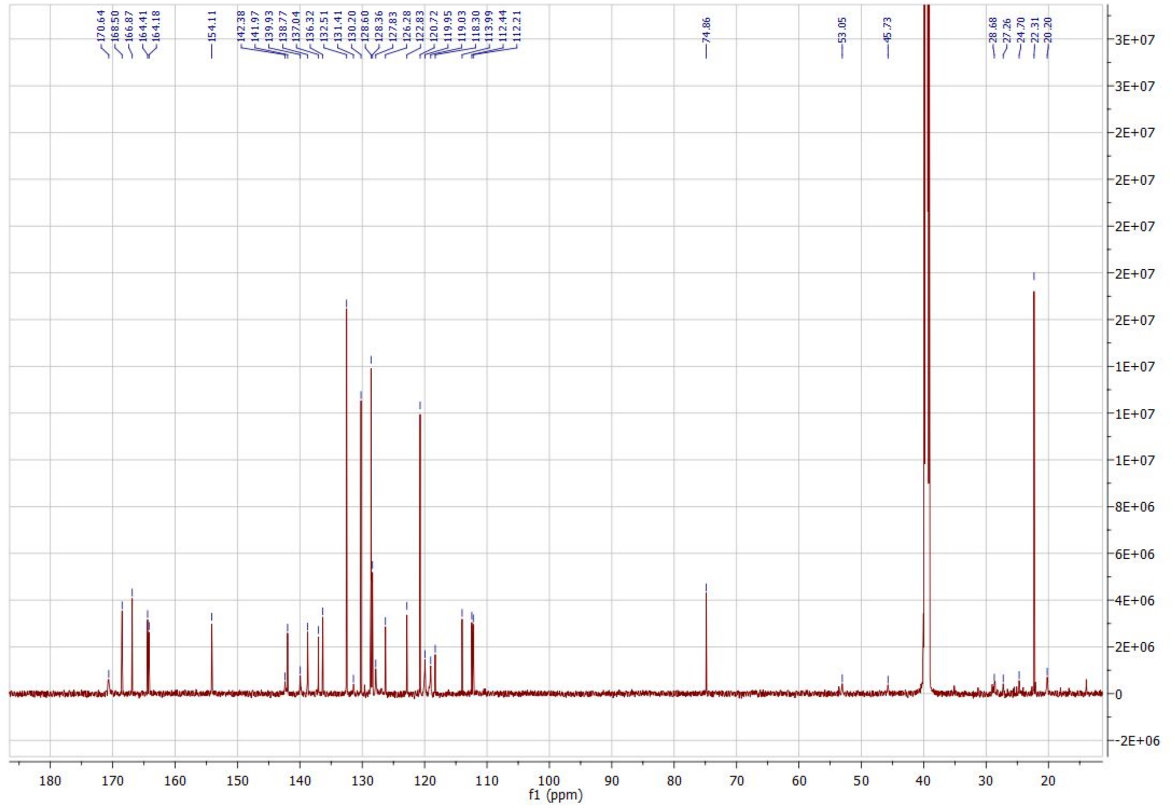

Figure S7. ^{1}H- (top) and ^{13}C-NMR (bottom) spectrum of compound **17**.

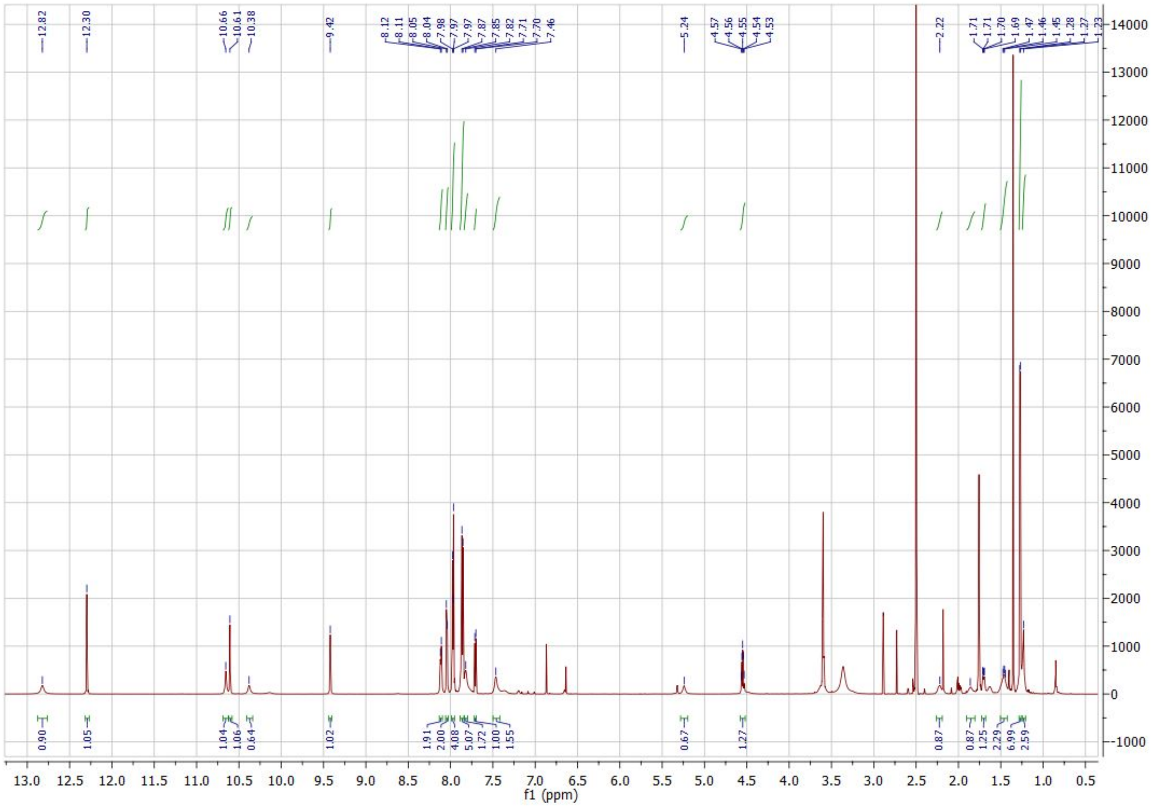

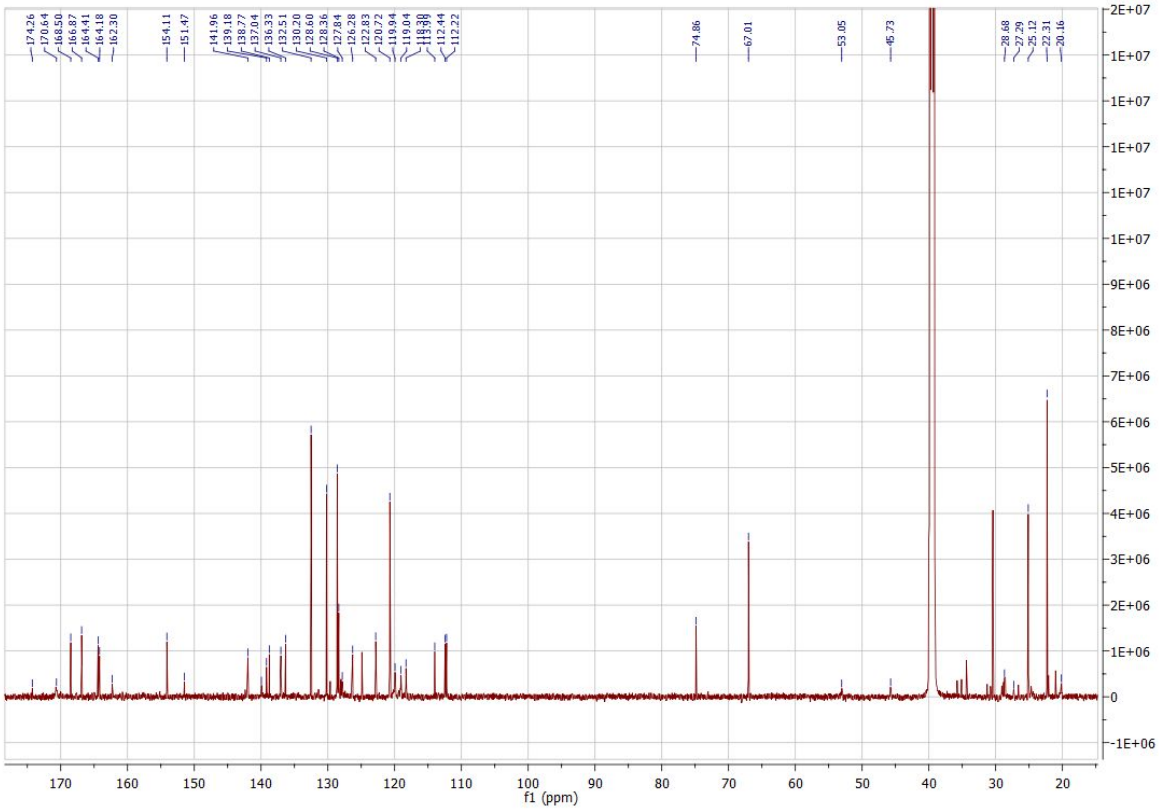

Figure S8. ^{1}H- (top) and ^{13}C-NMR (bottom) spectrum of compound **18**.

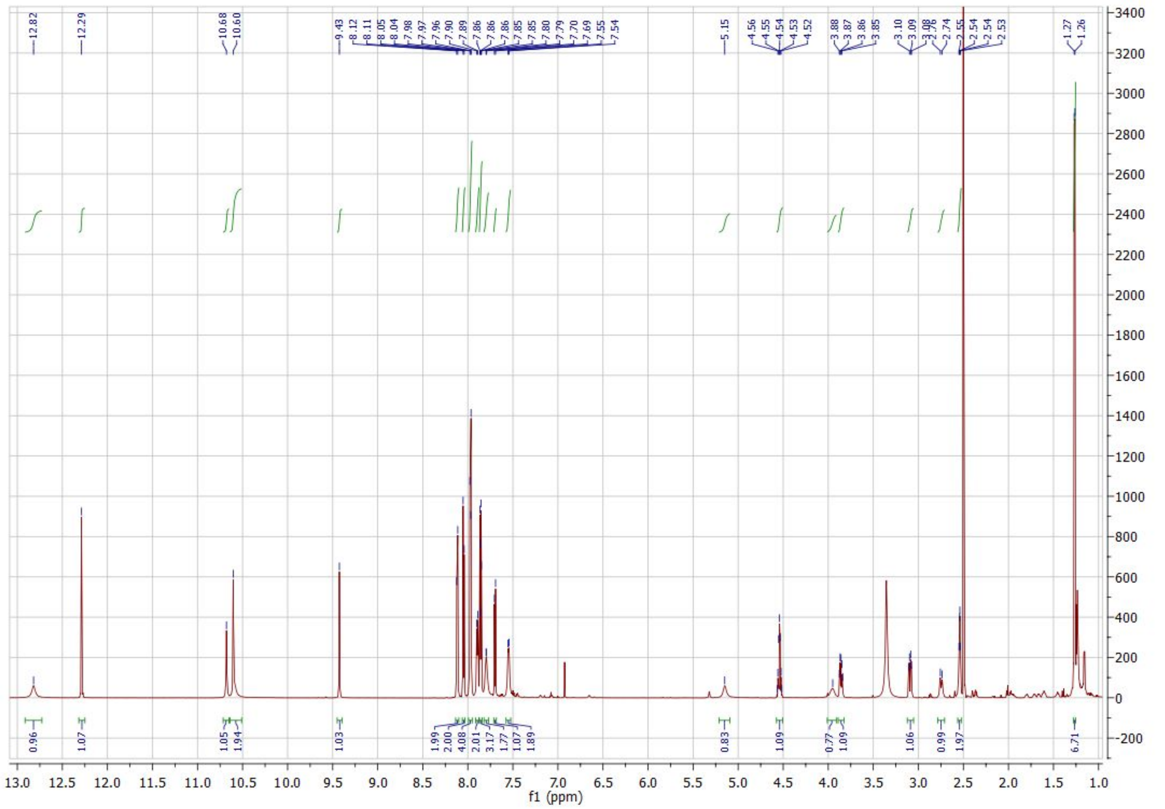

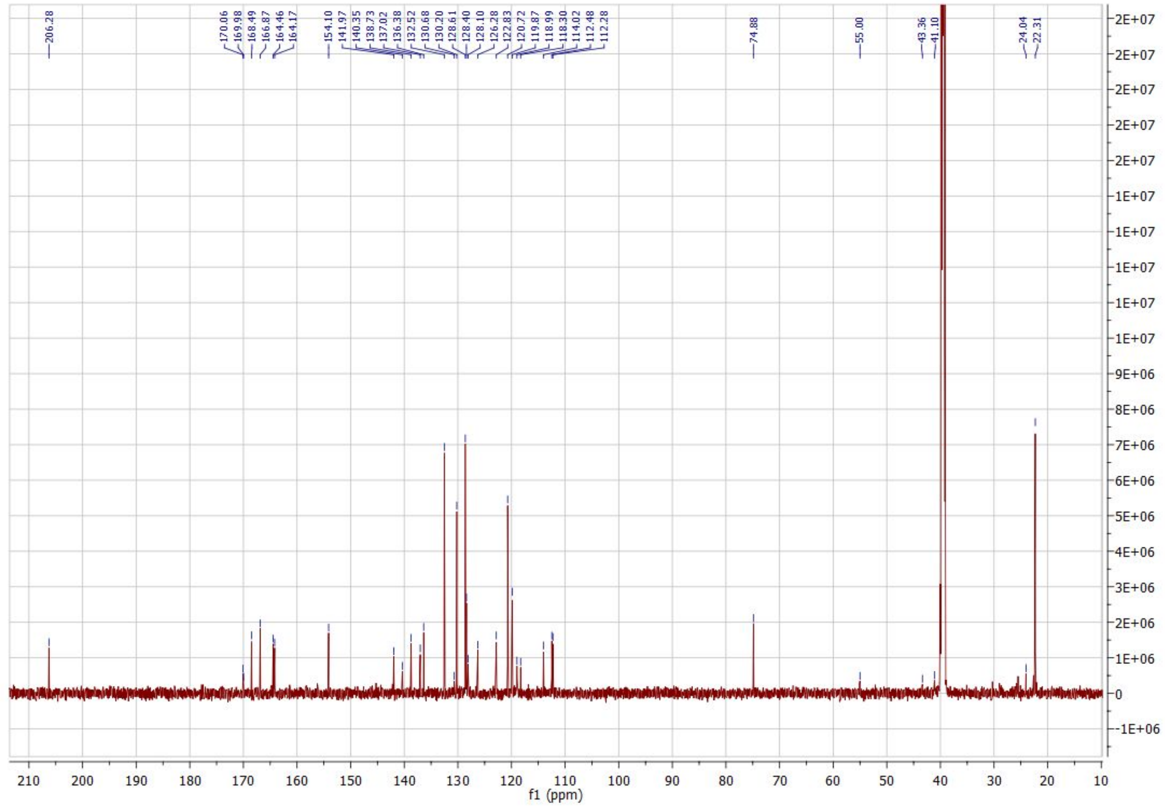

Figure S9. ^{1}H- (top) and ^{13}C-NMR (bottom) spectrum of compound **19**.

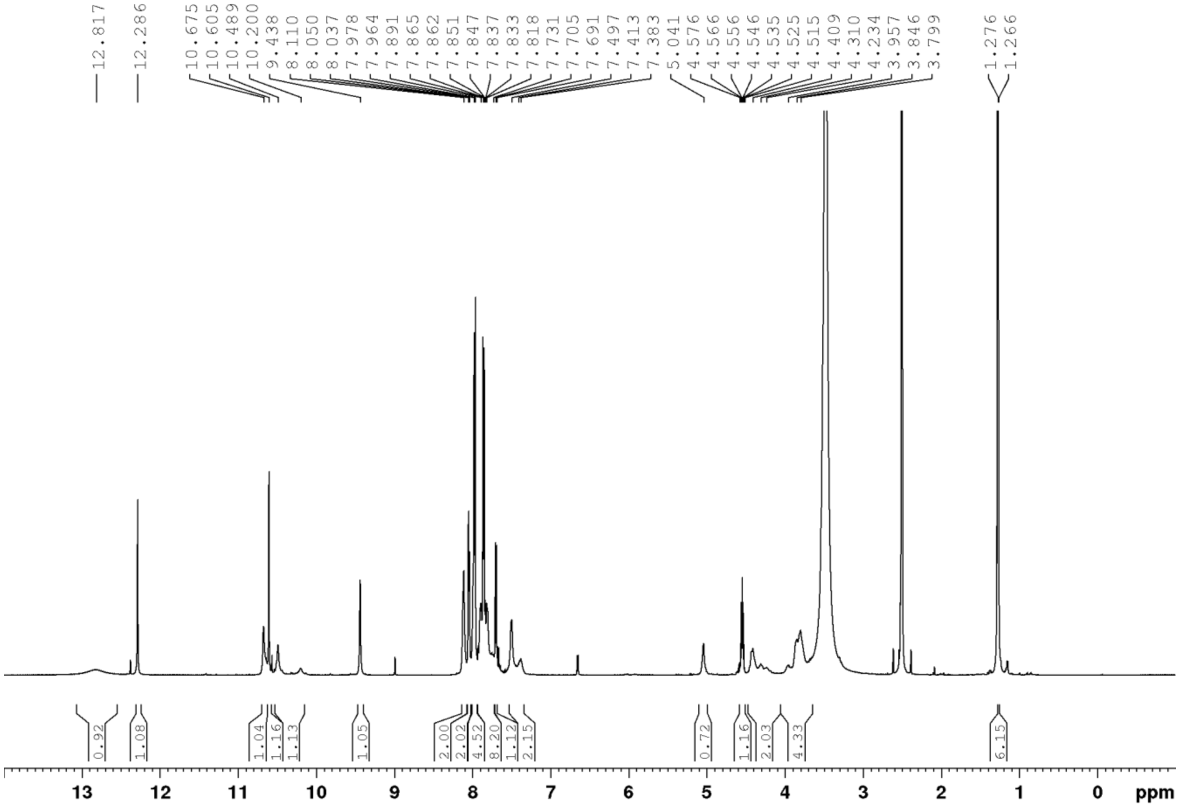

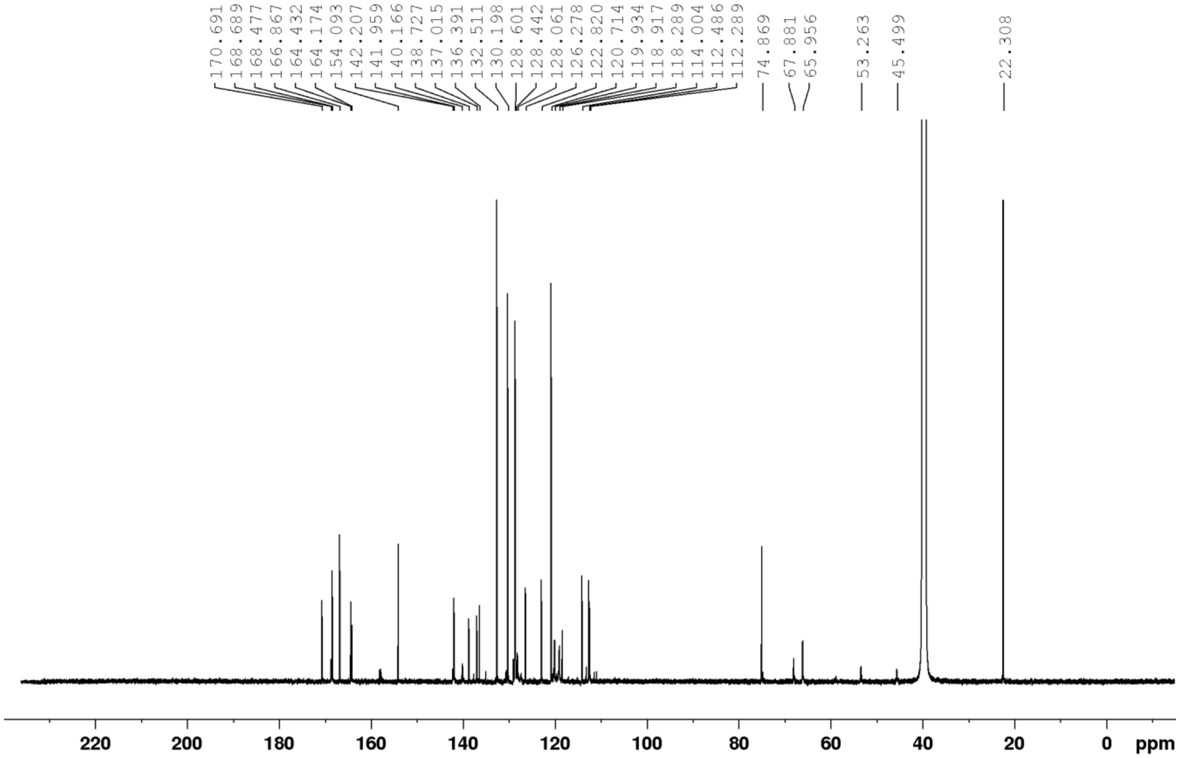

Figure S10. ^{1}H- (top) and ^{13}C-NMR (bottom) spectrum of compound **20**.

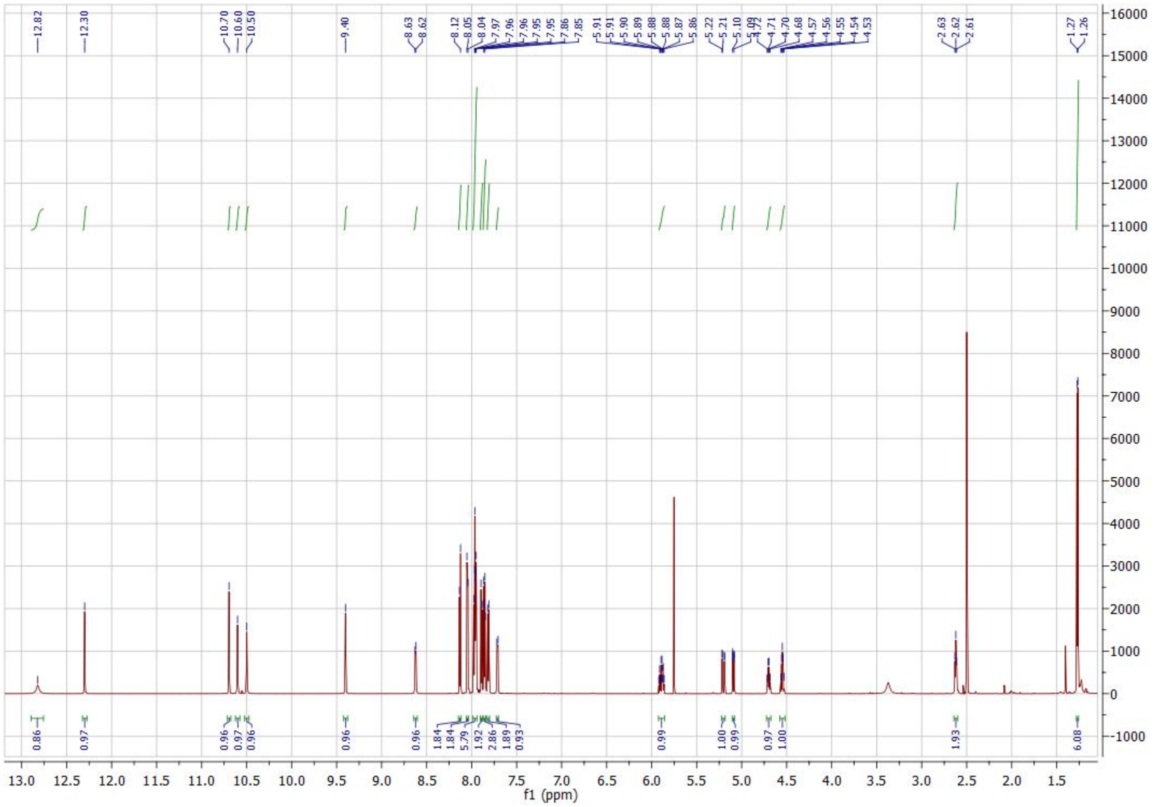

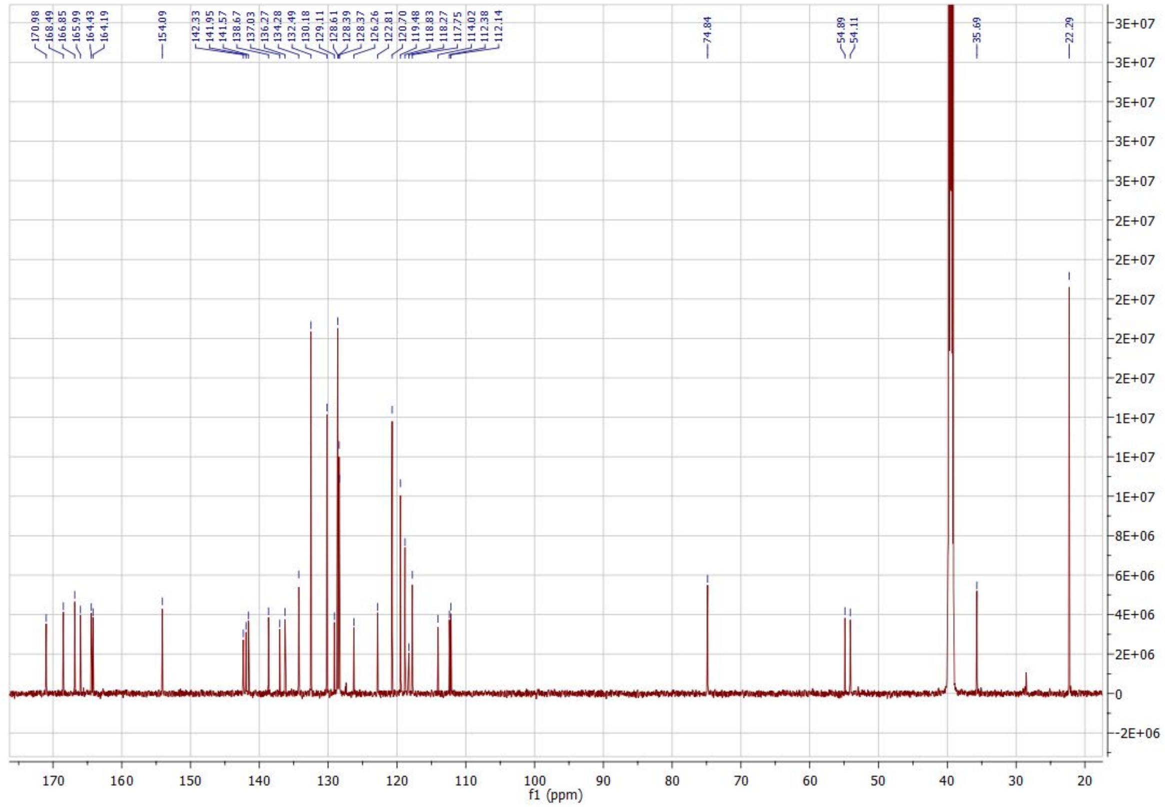

Figure S2. ^{1}H- (top) and ^{13}C-NMR (bottom) spectrum of compound **21**.

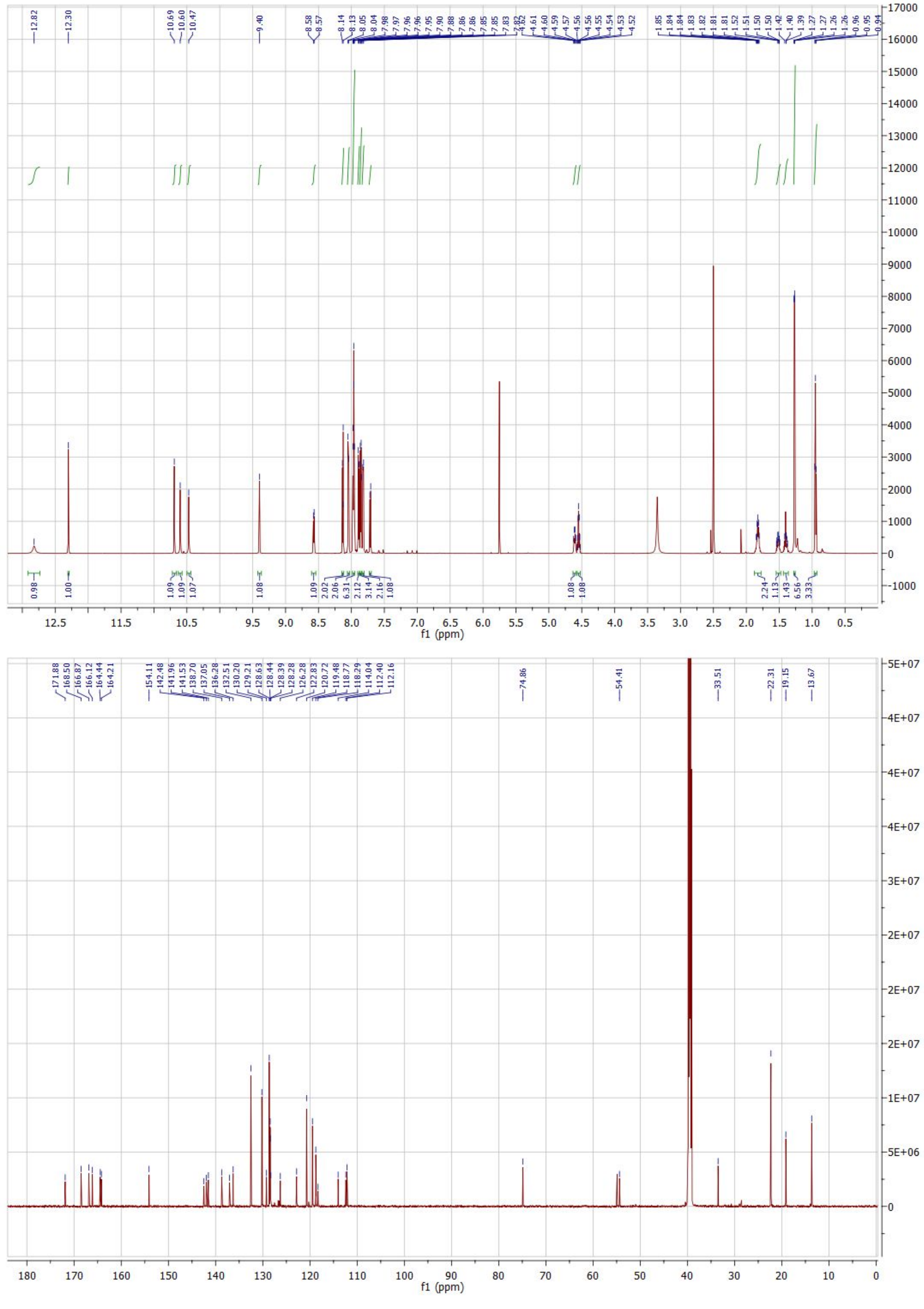

Figure S3. ^{1}H- (top) and ^{13}C-NMR (bottom) spectrum of compound **22**.

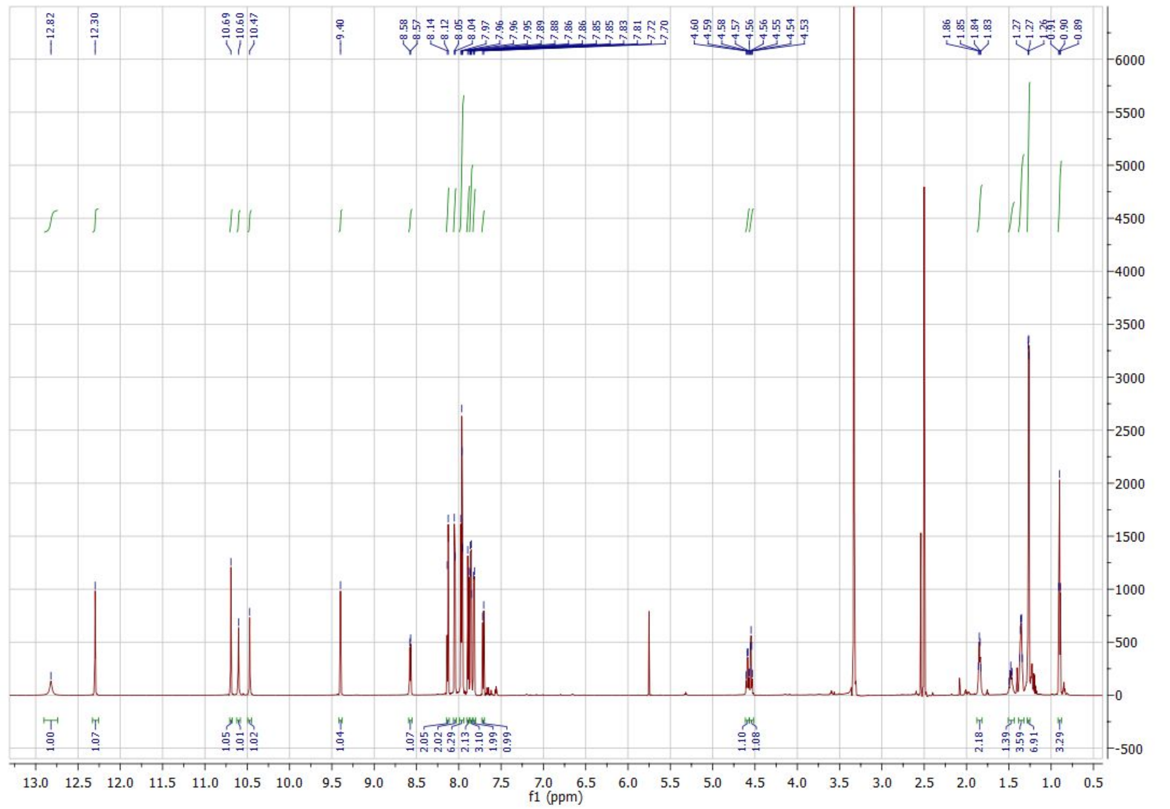

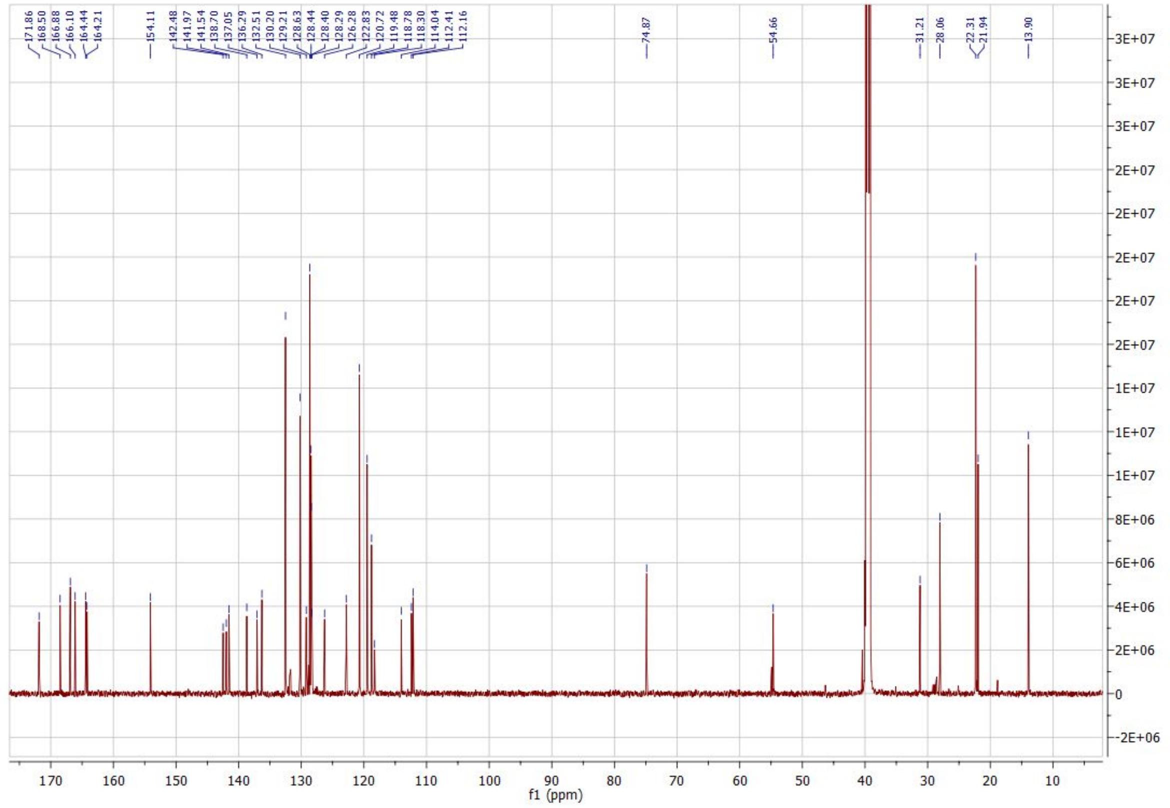

Figure S4. ^{1}H- (top) and ^{13}C-NMR (bottom) spectrum of compound **23**.

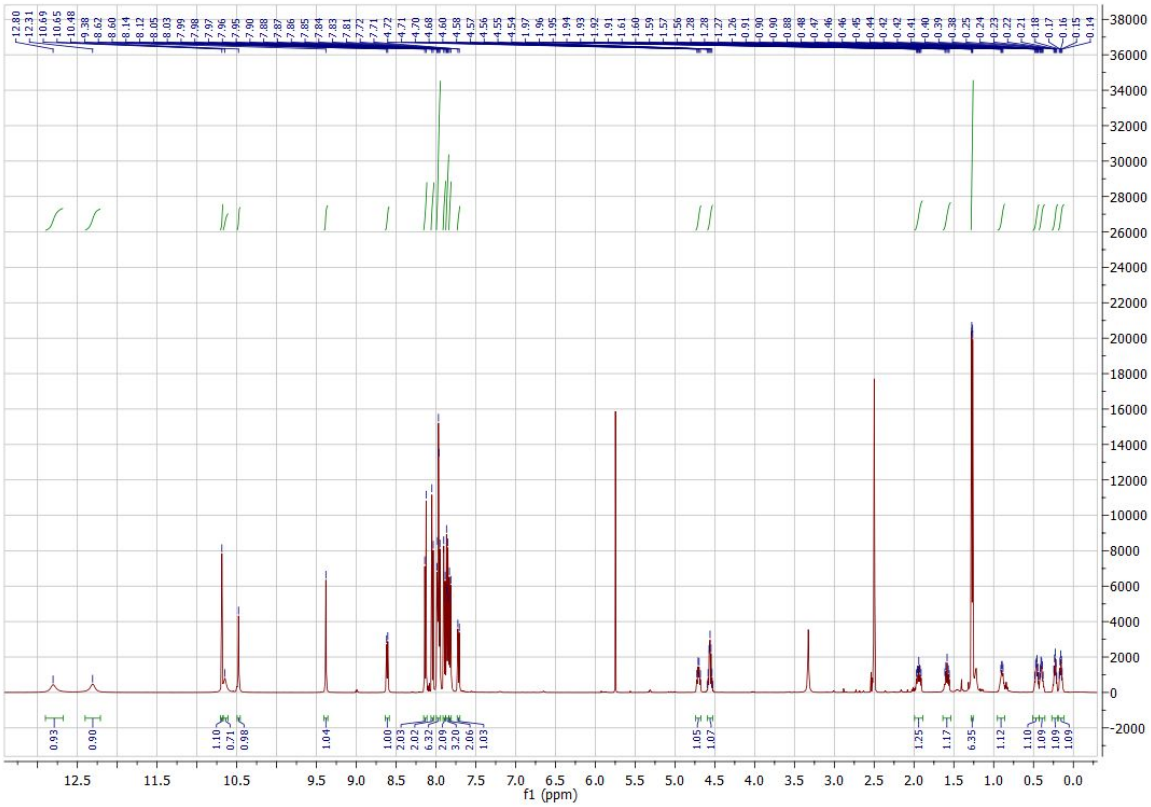

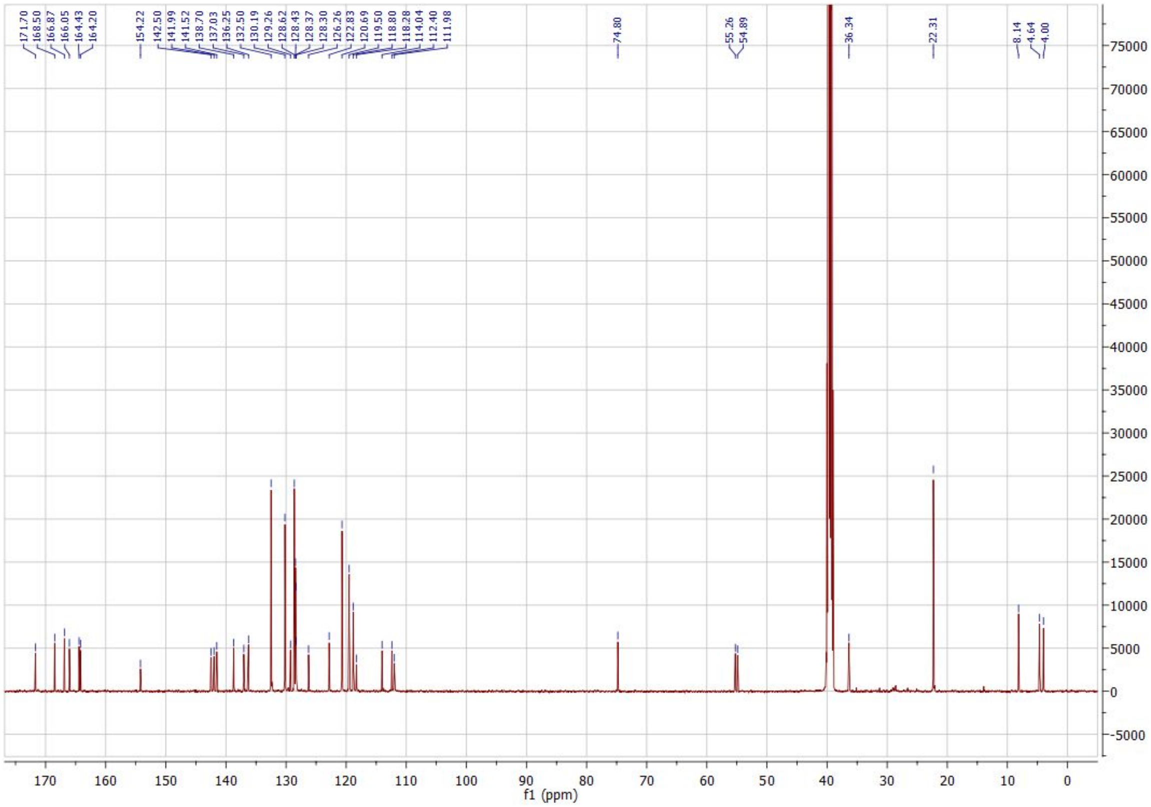

Figure S5. ^{1}H- (top) and ^{13}C-NMR (bottom) spectrum of compound **24**.

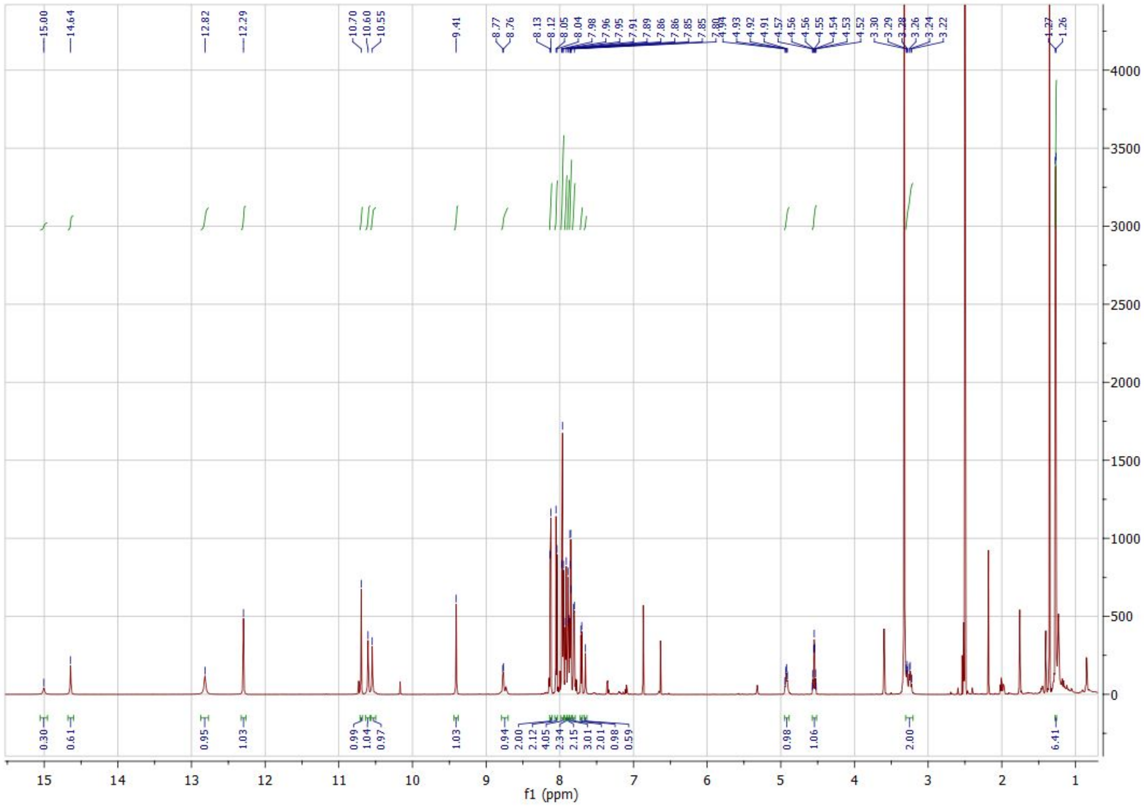

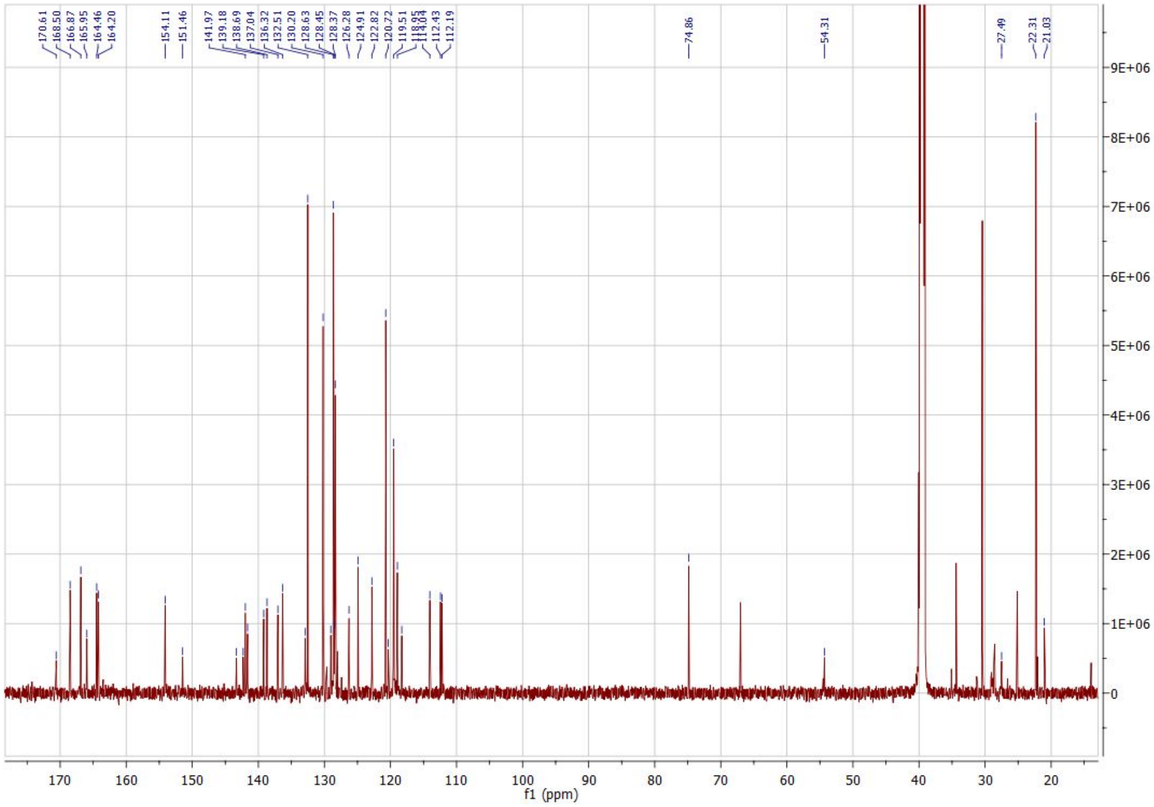

Figure S6. ^{1}H- (top) and ^{13}C-NMR (bottom) spectrum of compound **25**.

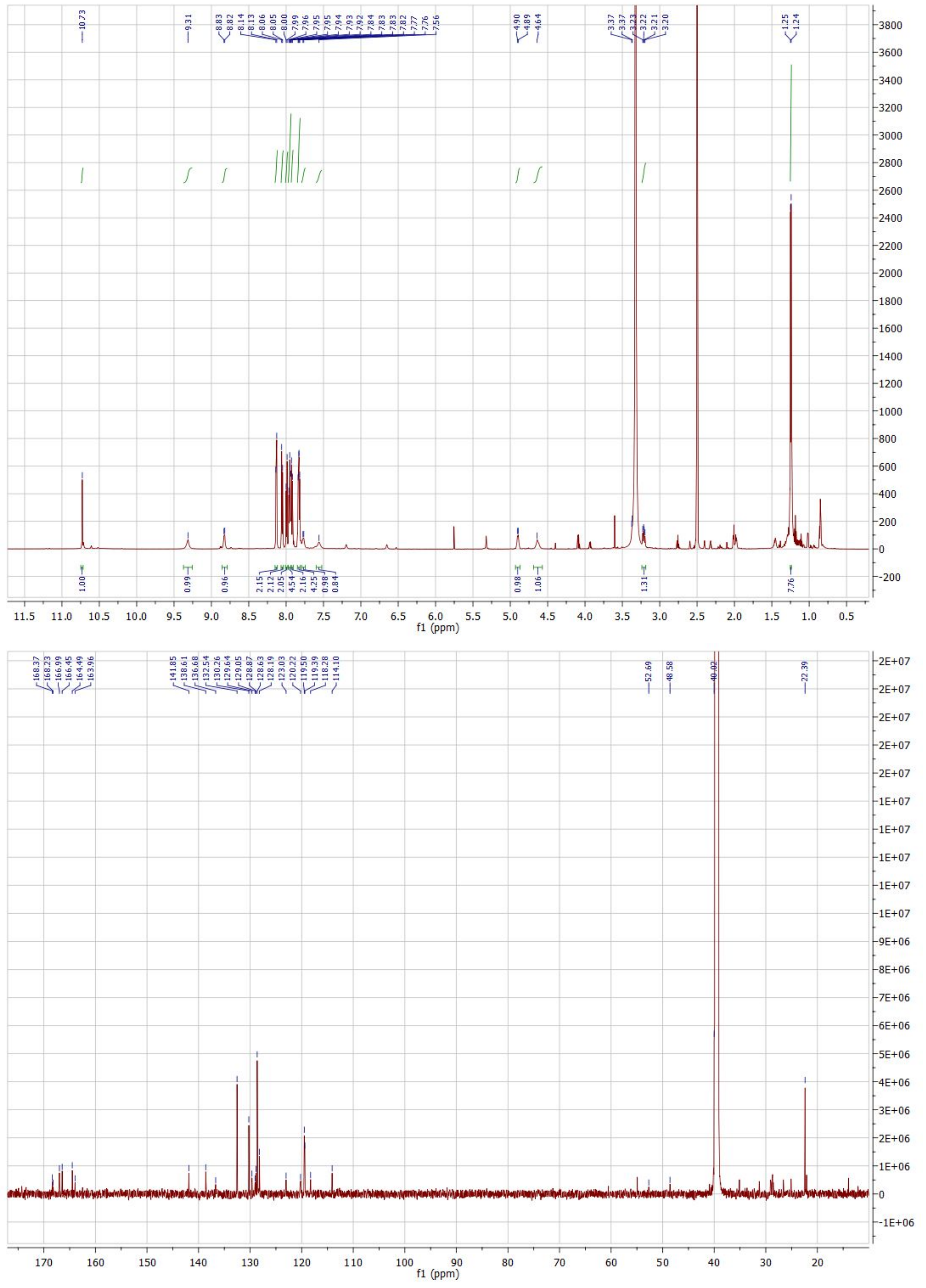

Figure S7. ^{1}H- (top) and ^{13}C-NMR (bottom) spectrum of compound **26**.

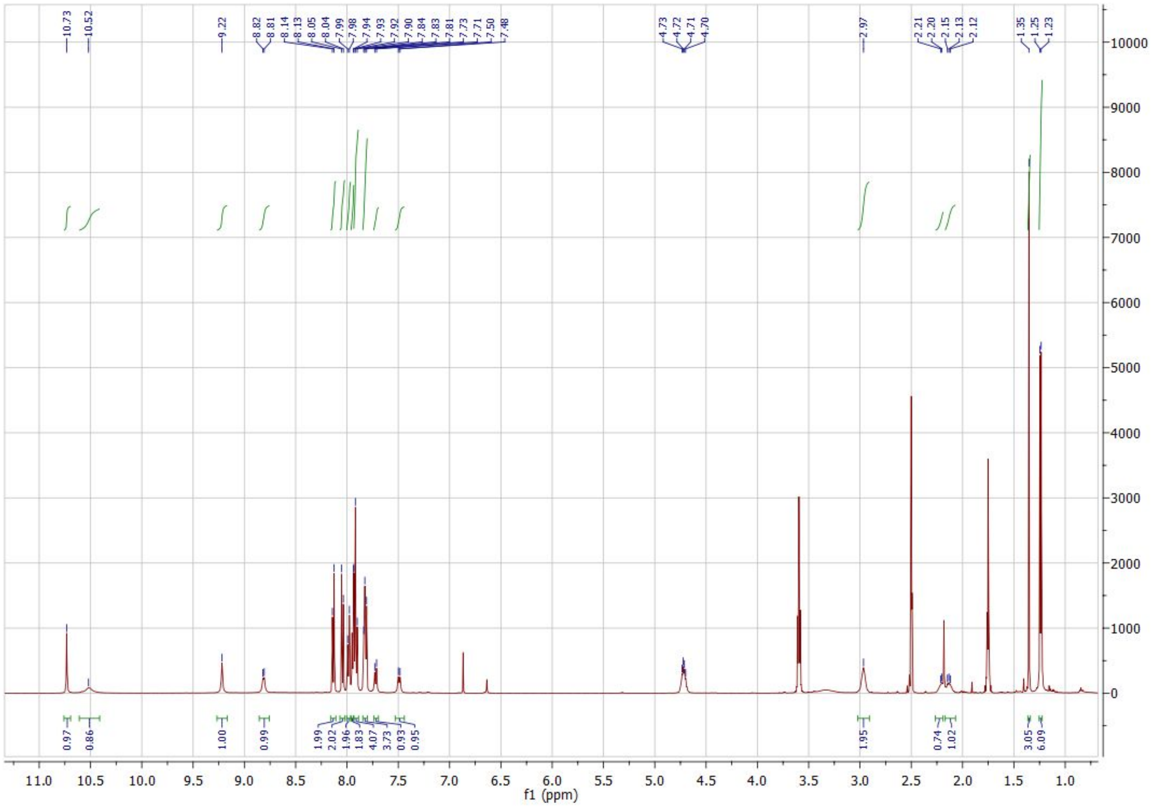

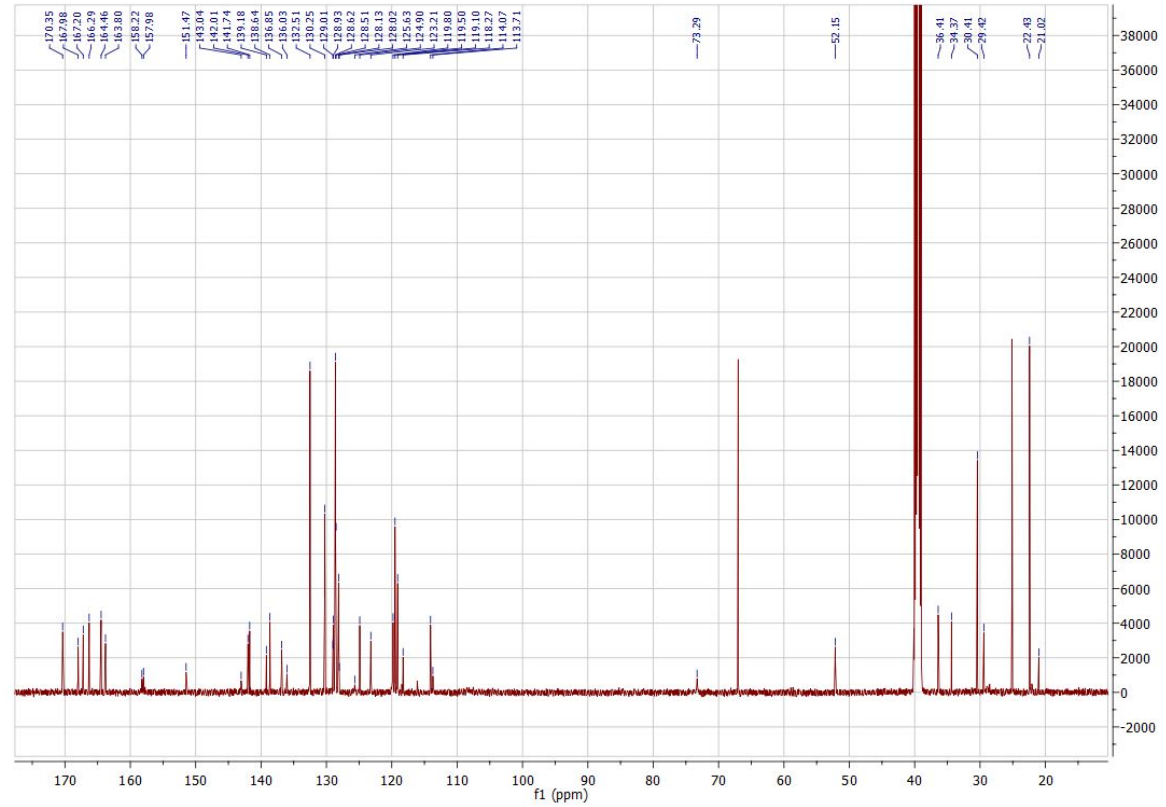

Figure S8. ¹H- (top) and ¹³C-NMR (bottom) spectrum of compound **27**.

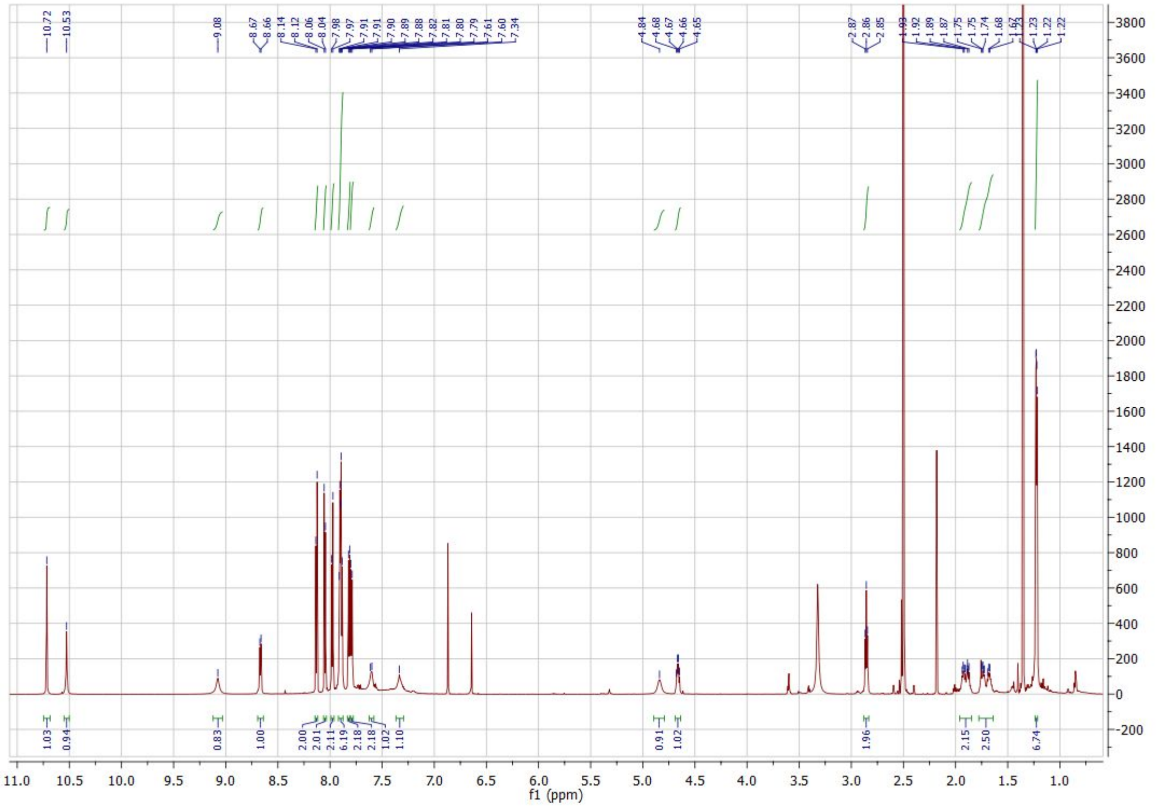

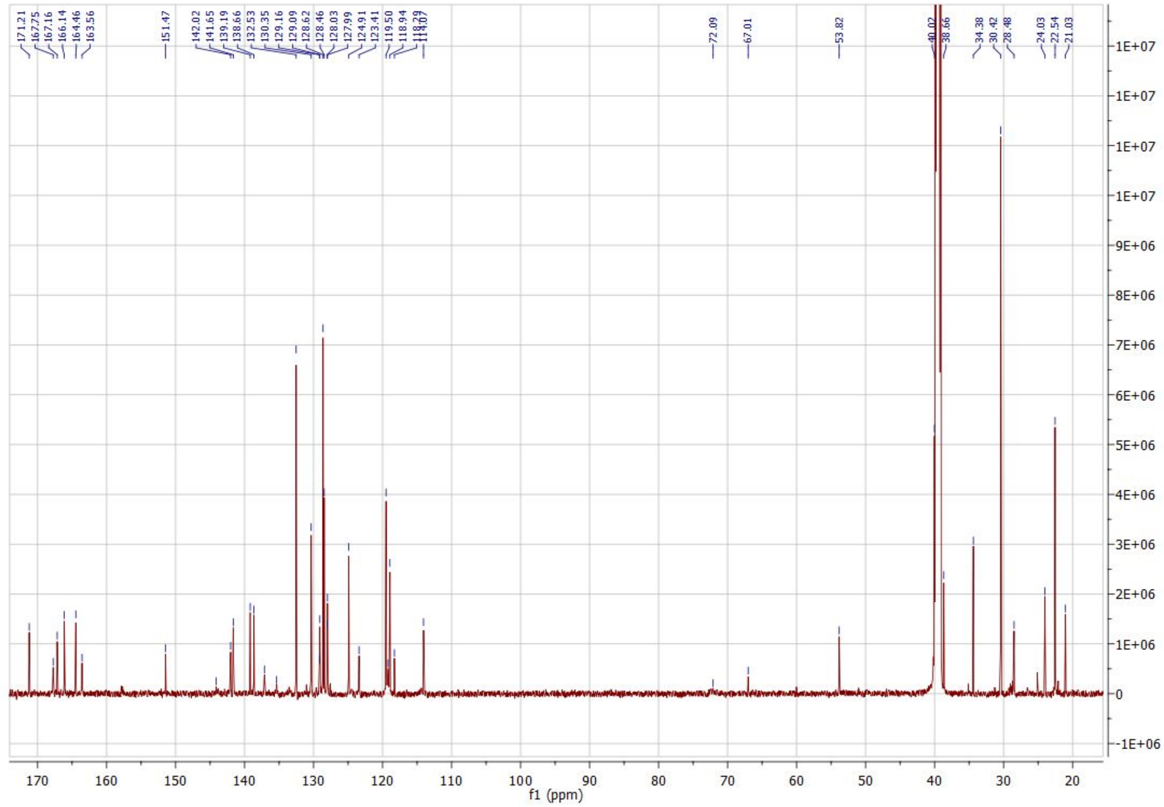

Figure S9. ^{1}H- (top) and ^{13}C-NMR (bottom) spectrum of compound **28**.

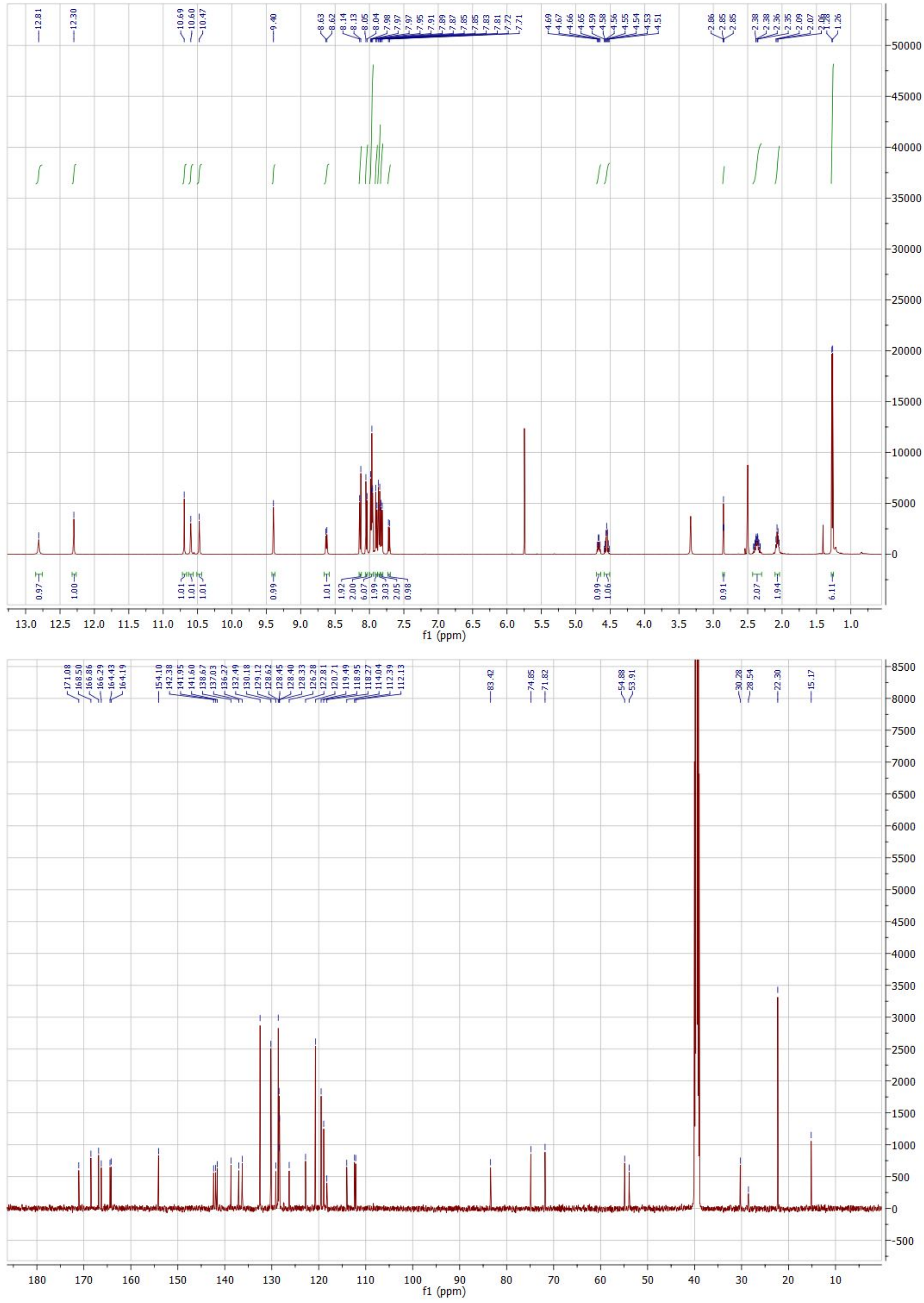

Figure S10. ^{1}H- (top) and ^{13}C-NMR (bottom) spectrum of compound **29**.

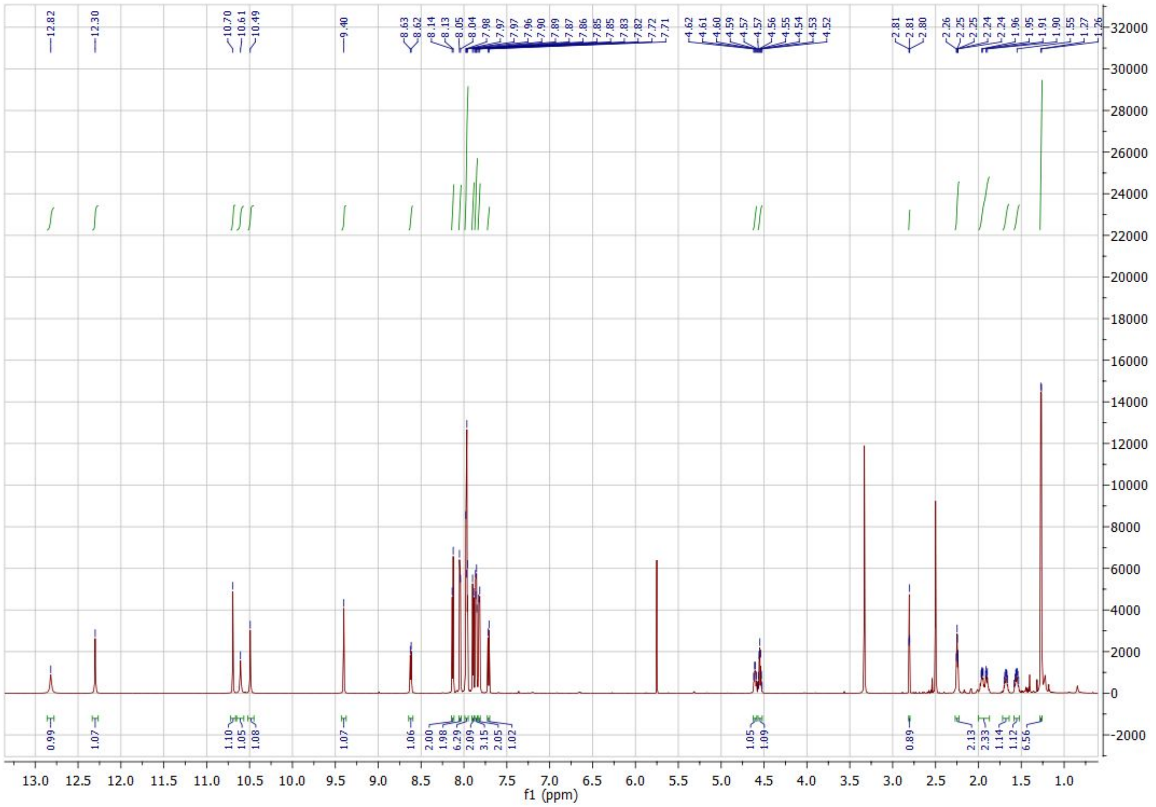

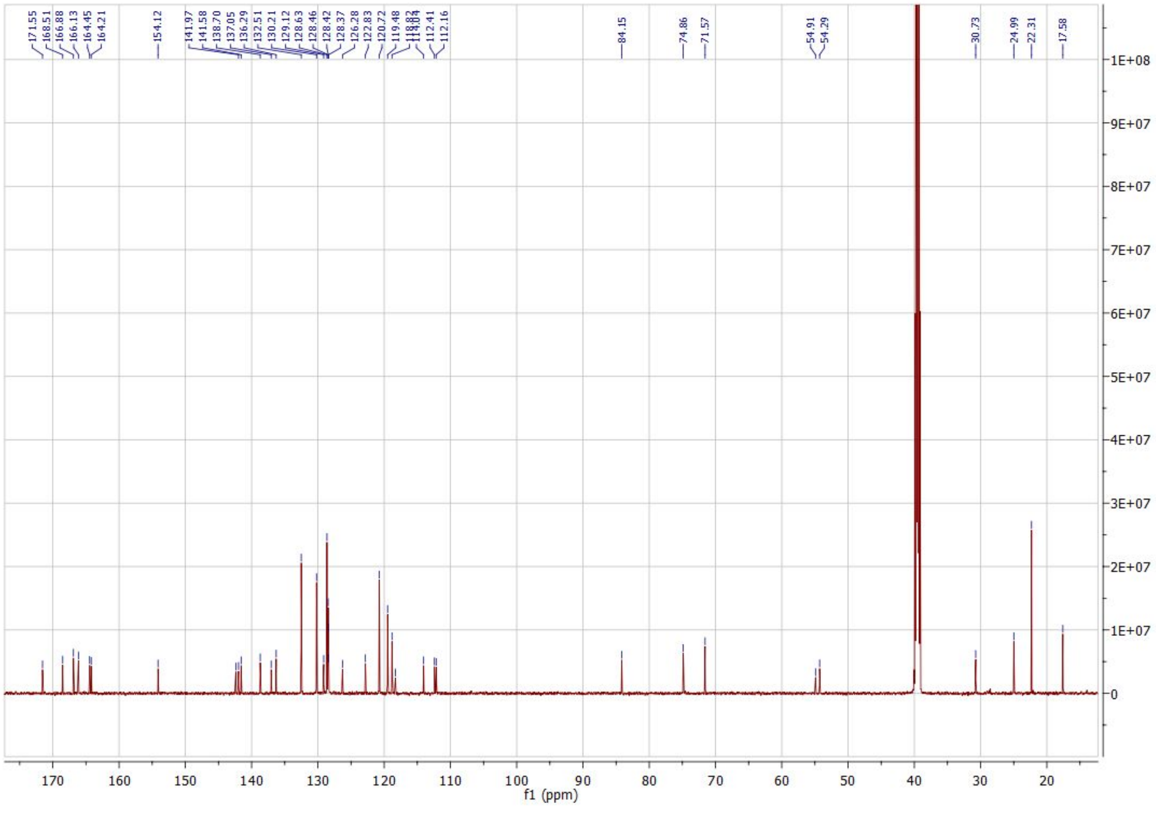

Figure S11. ^{1}H- (top) and ^{13}C-NMR (bottom) spectrum of compound **30**.

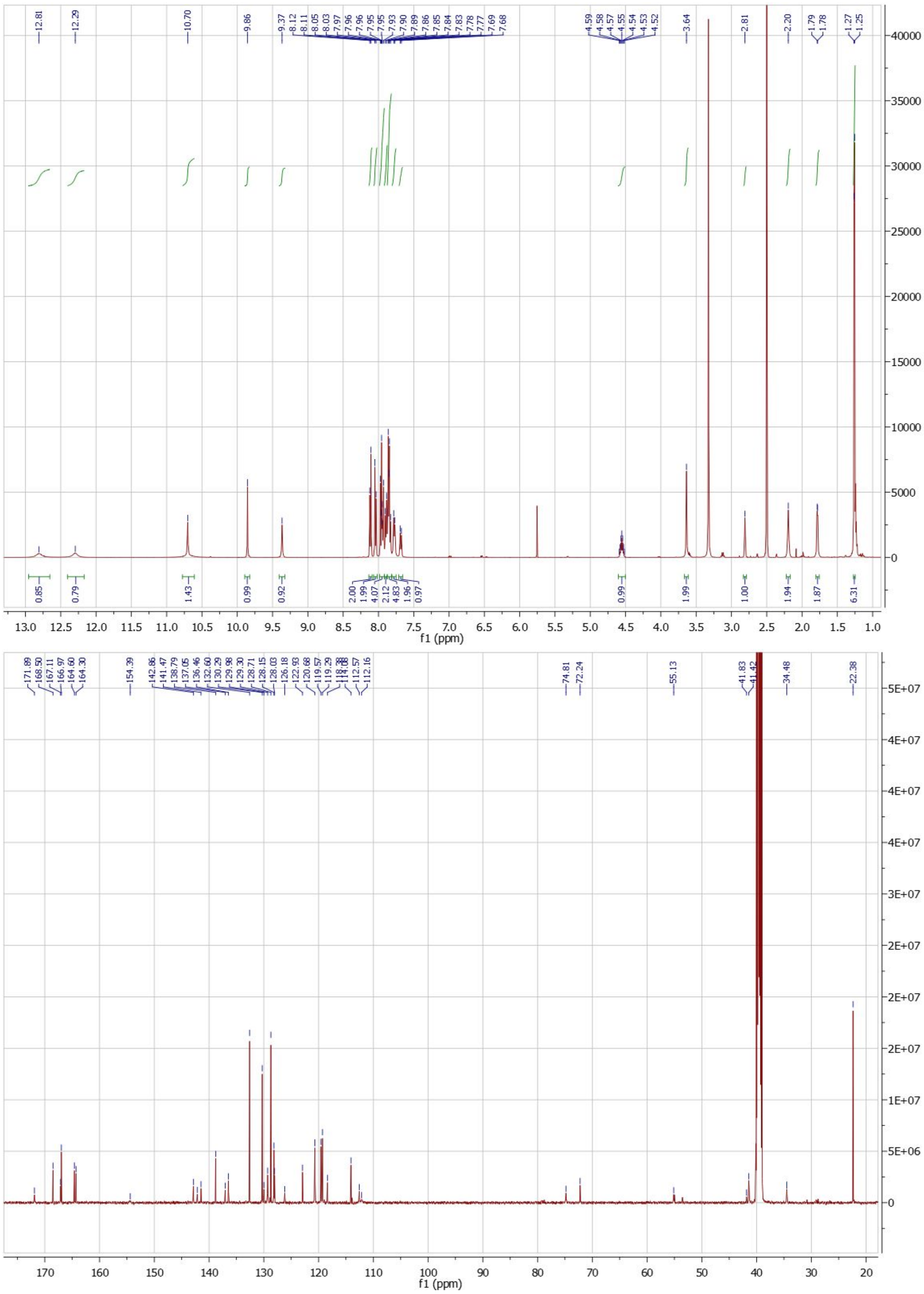

Figure S12. ^{1}H- (top) and ^{13}C-NMR (bottom) spectrum of compound **31**.

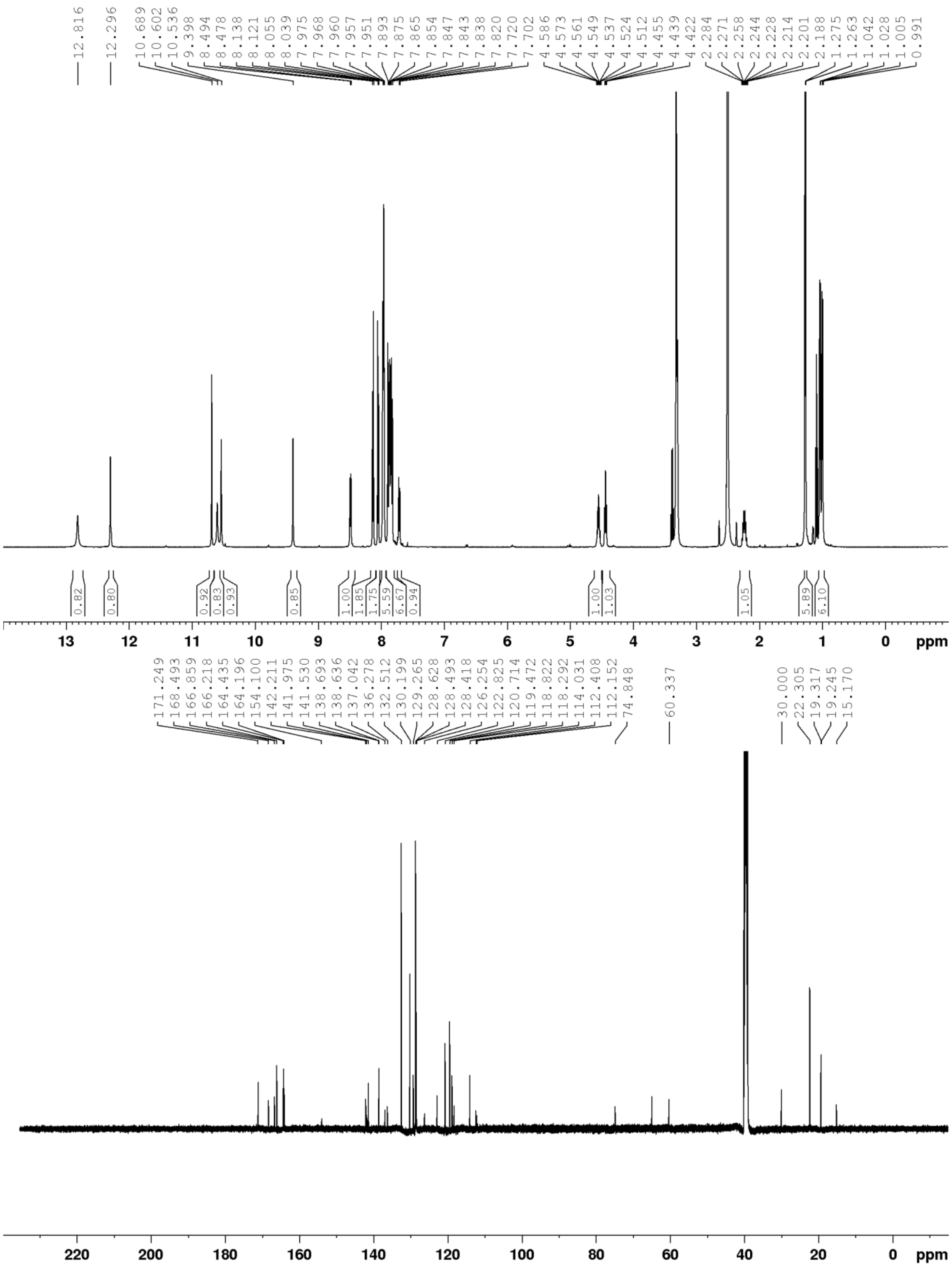

Figure S13. ^{1}H- (top) and ^{13}C-NMR (bottom) spectrum of compound **32**.

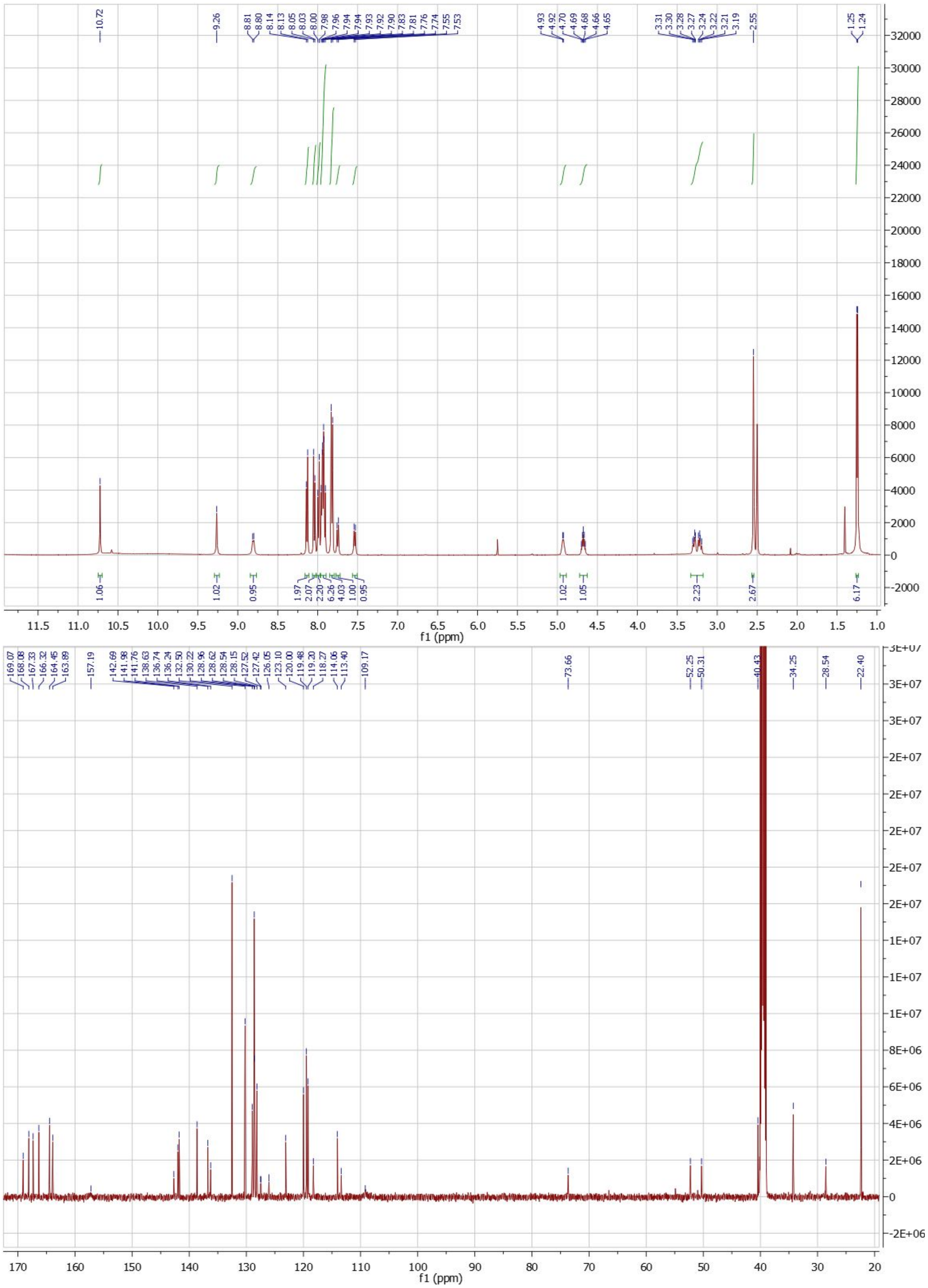

Figure S14. ^{1}H- (top) and ^{13}C-NMR (bottom) spectrum of compound **34**.

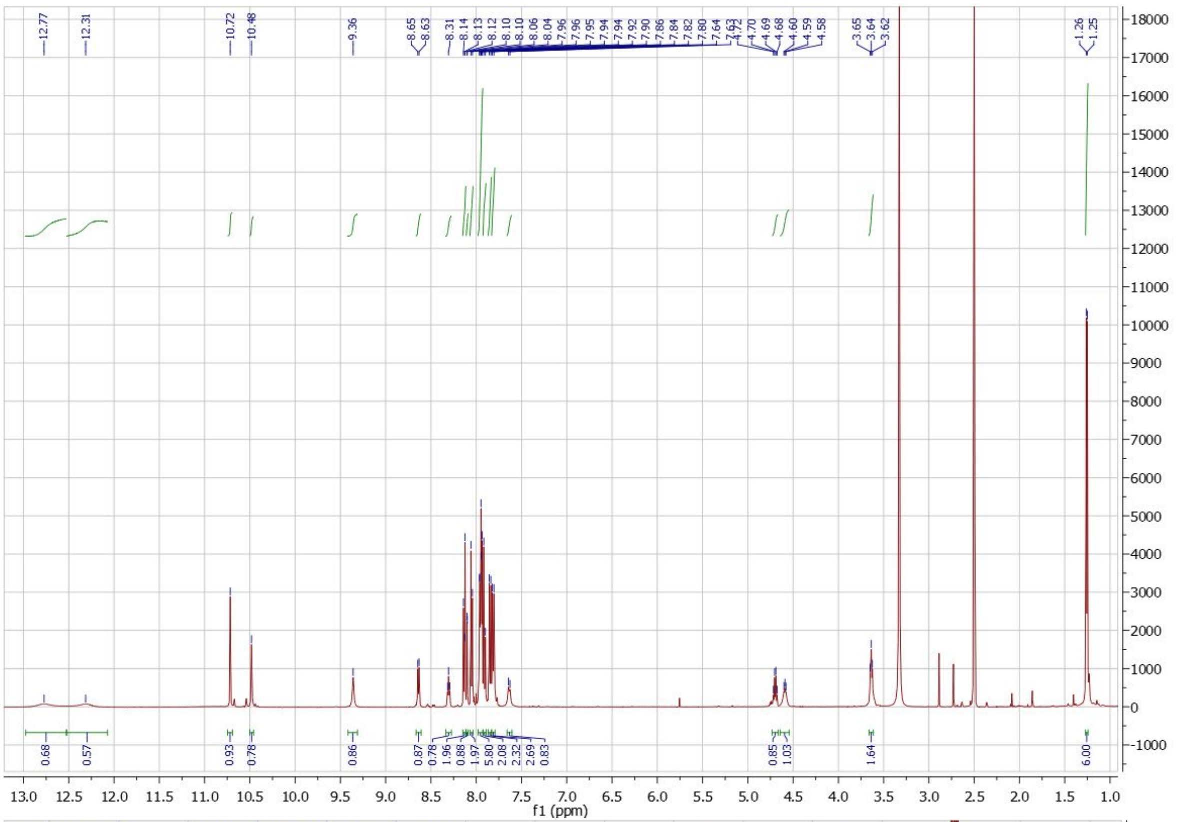

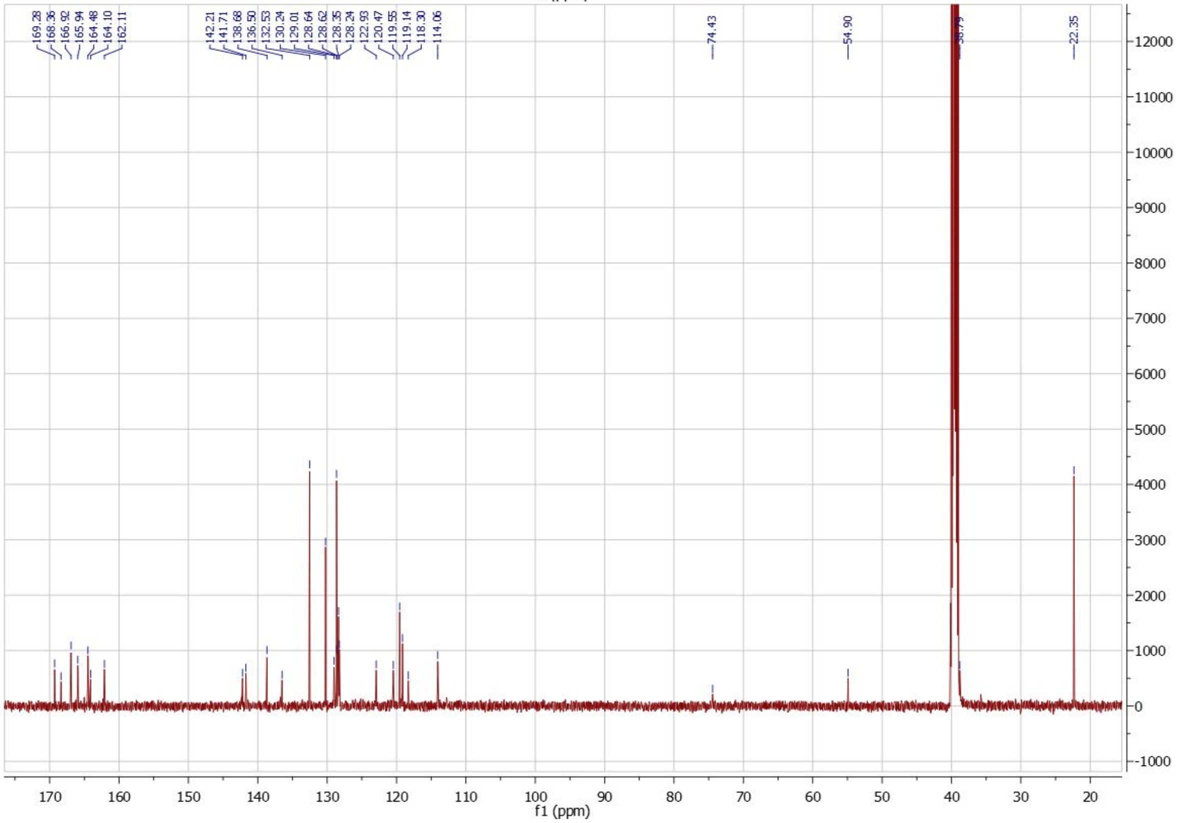

Figure S15. ^{1}H- (top) and ^{13}C-NMR (bottom) spectrum of compound **35**.

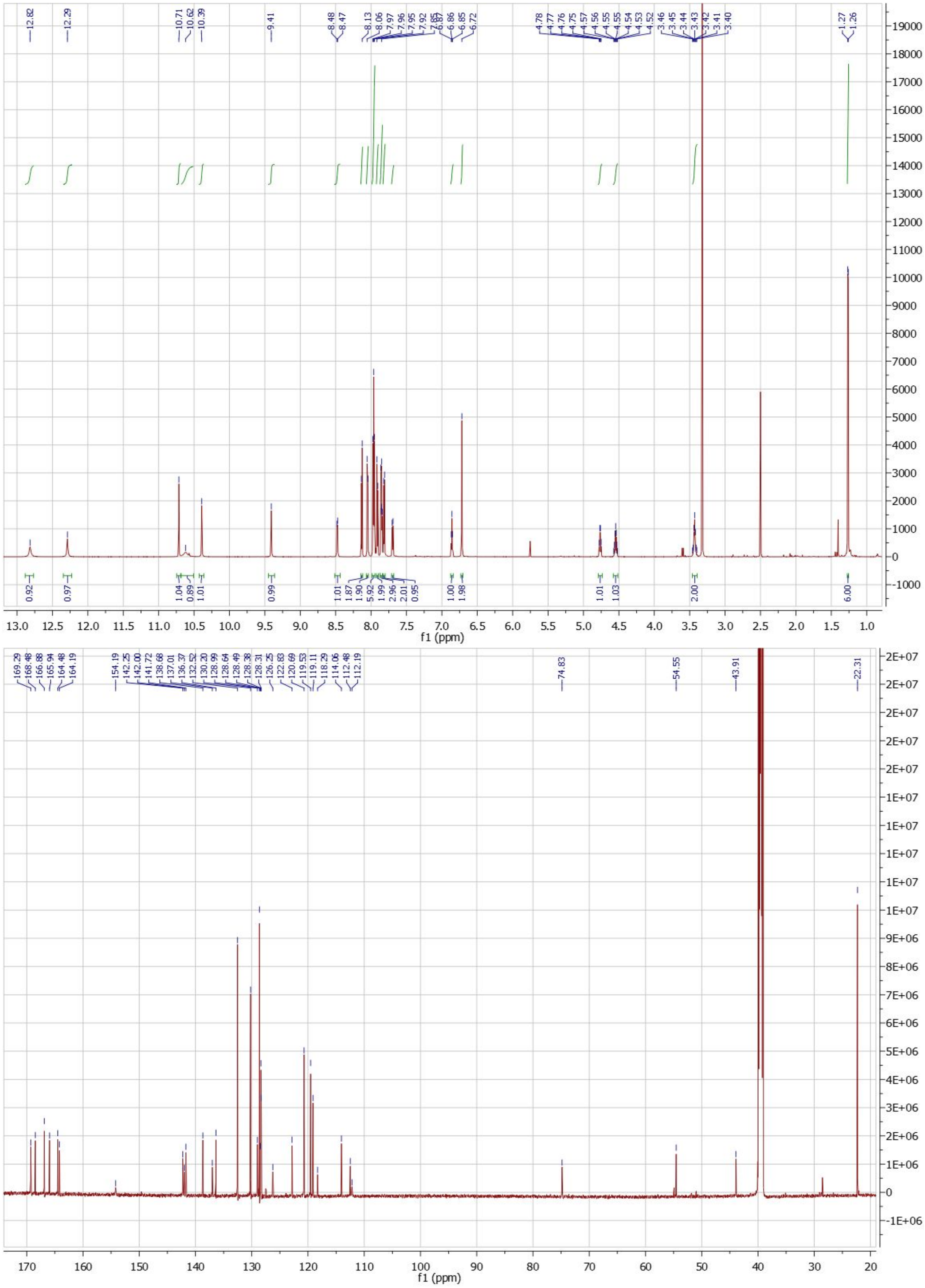

Figure S16. ^{1}H- (top) and ^{13}C-NMR (bottom) spectrum of compound **36**.

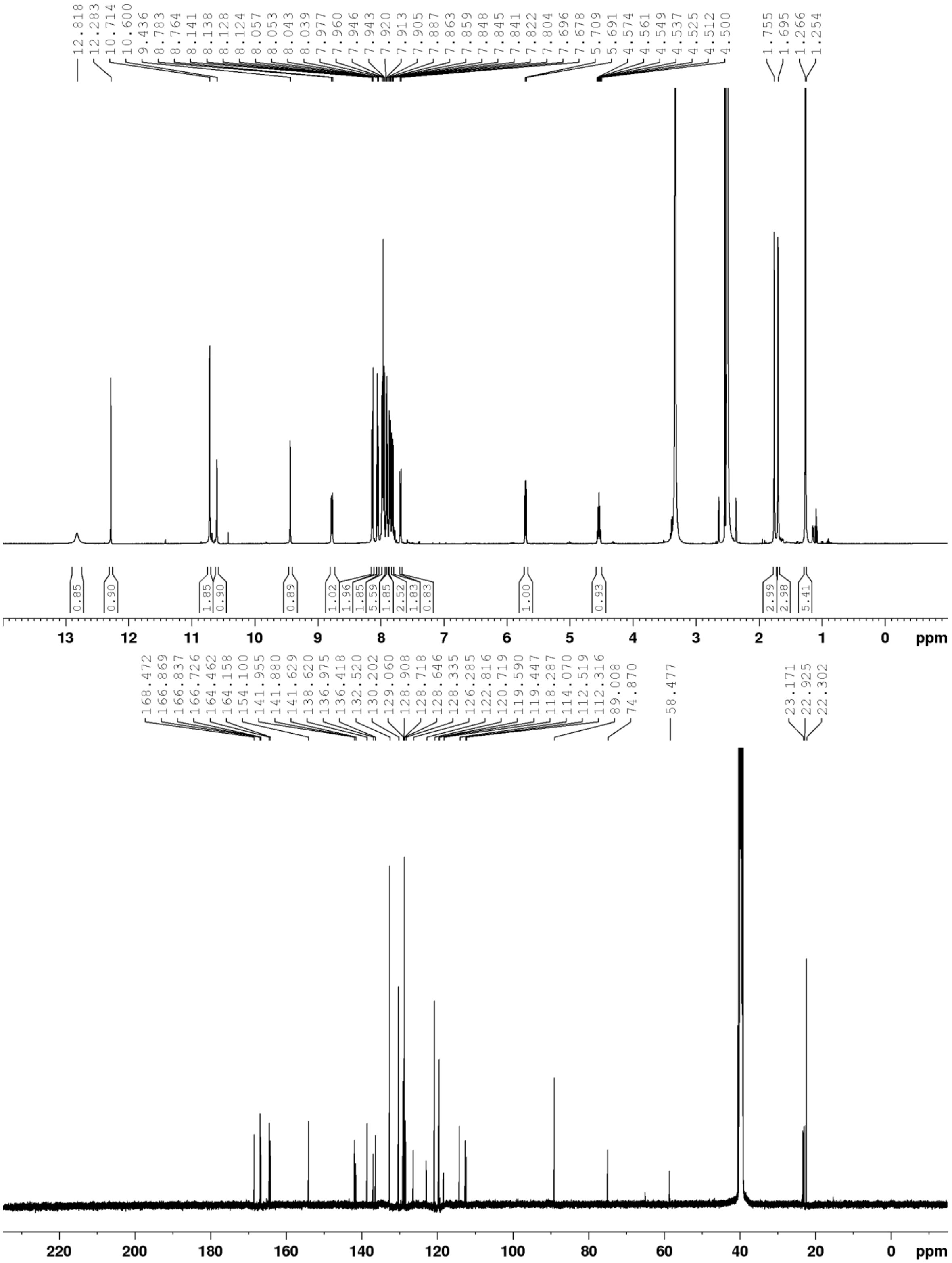

Figure S17. ^{1}H- (top) and ^{13}C-NMR (bottom) spectrum of compound **37**.

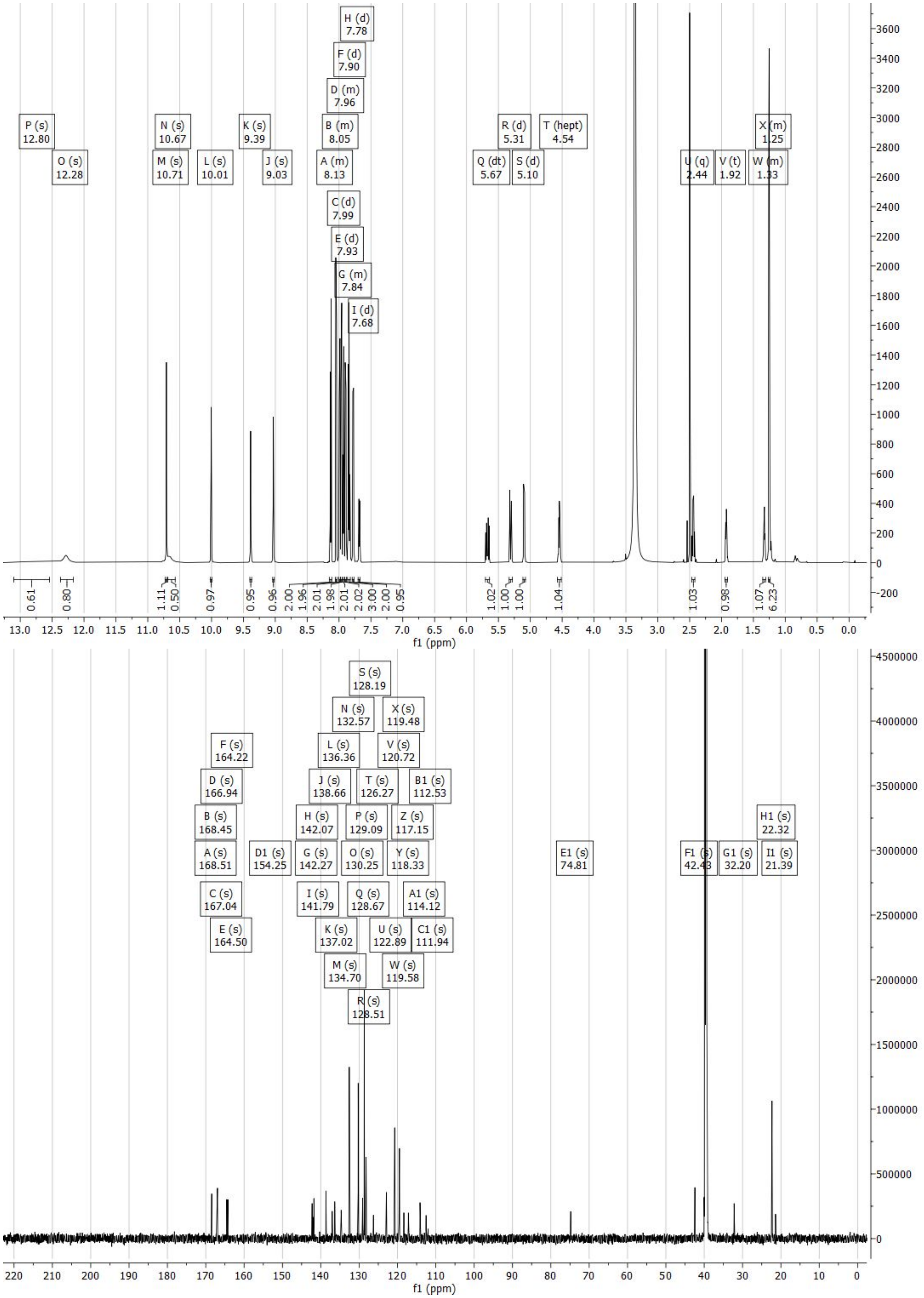

Figure S18. ^{1}H- (top) and ^{13}C-NMR (bottom) spectrum of compound **38**.

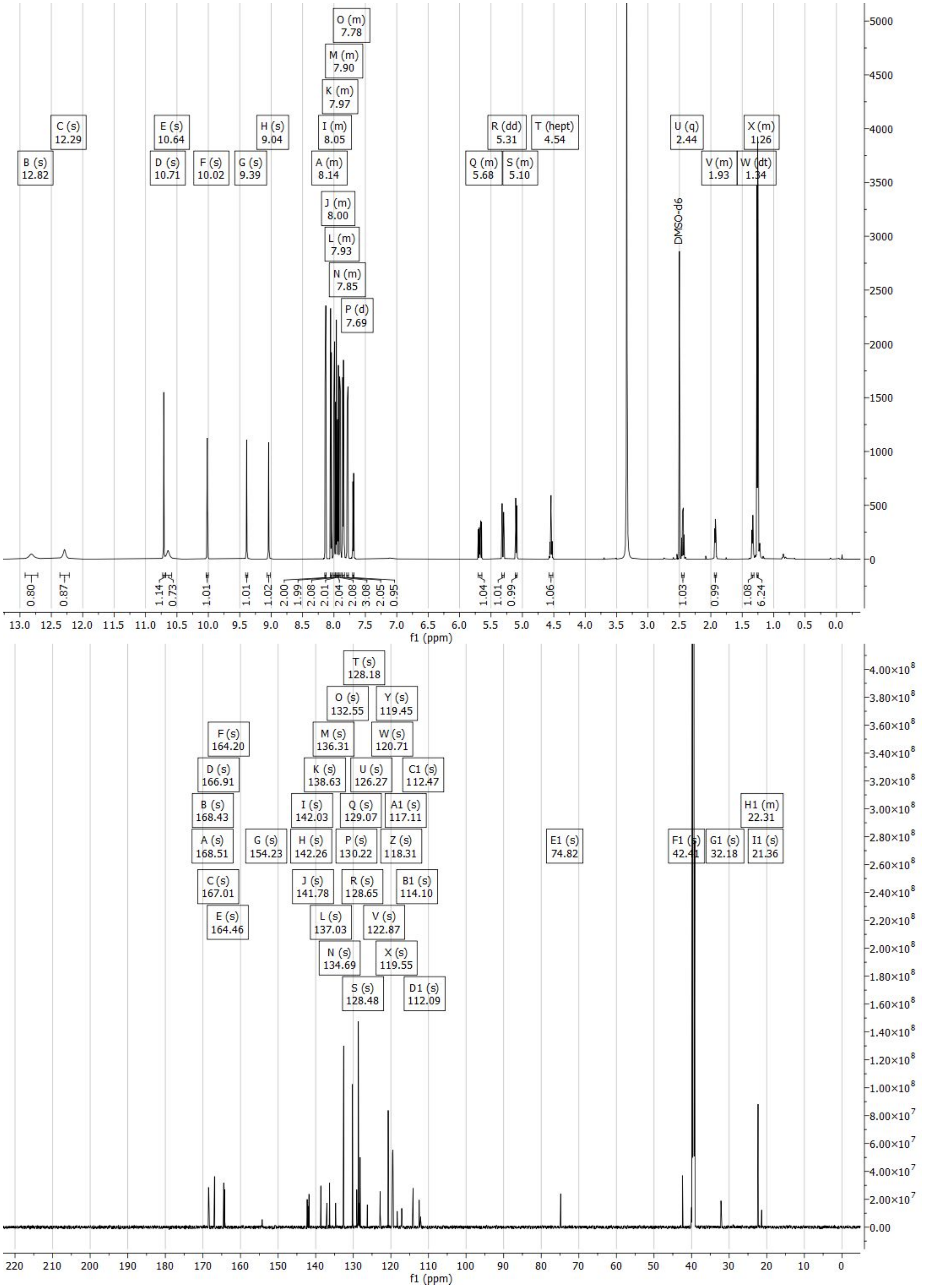

Figure S19. ^{1}H- (top) and ^{13}C-NMR (bottom) spectrum of compound **39**.

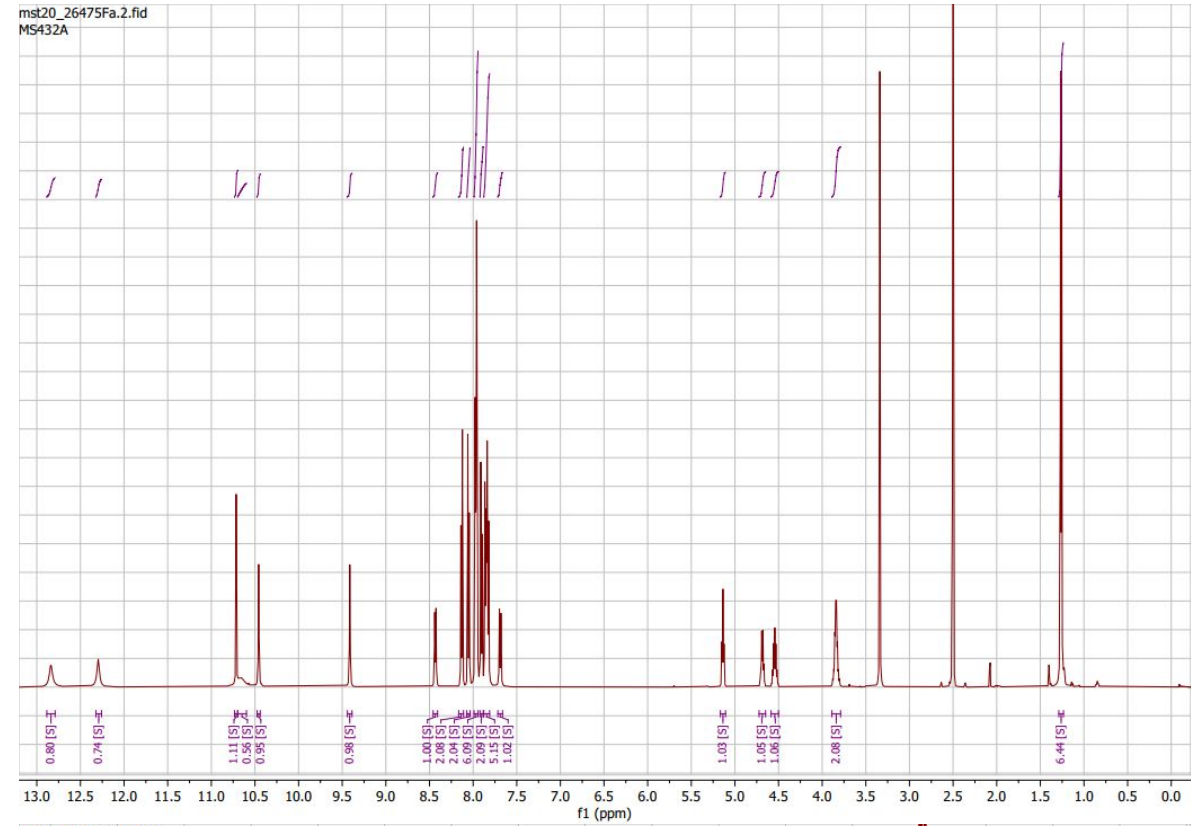

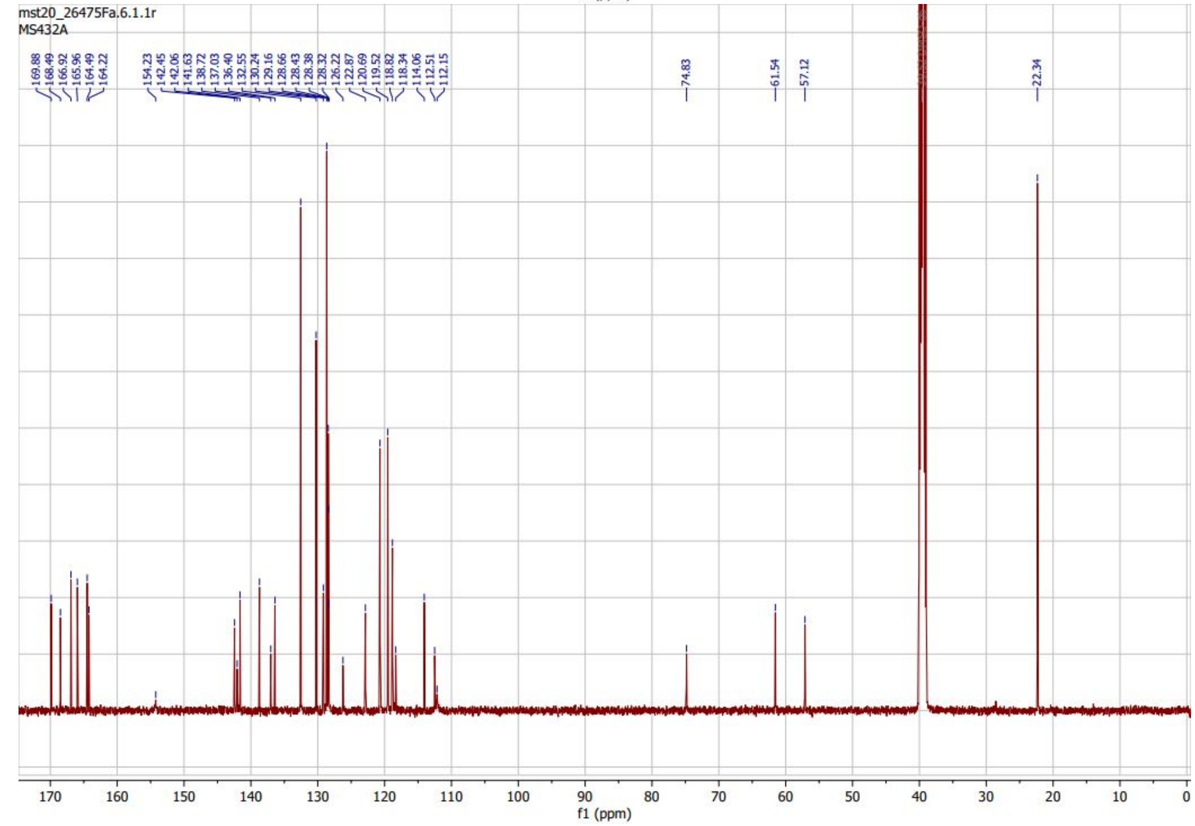

Figure S20. ^{1}H- (top) and ^{13}C-NMR (bottom) spectrum of compound **40**.

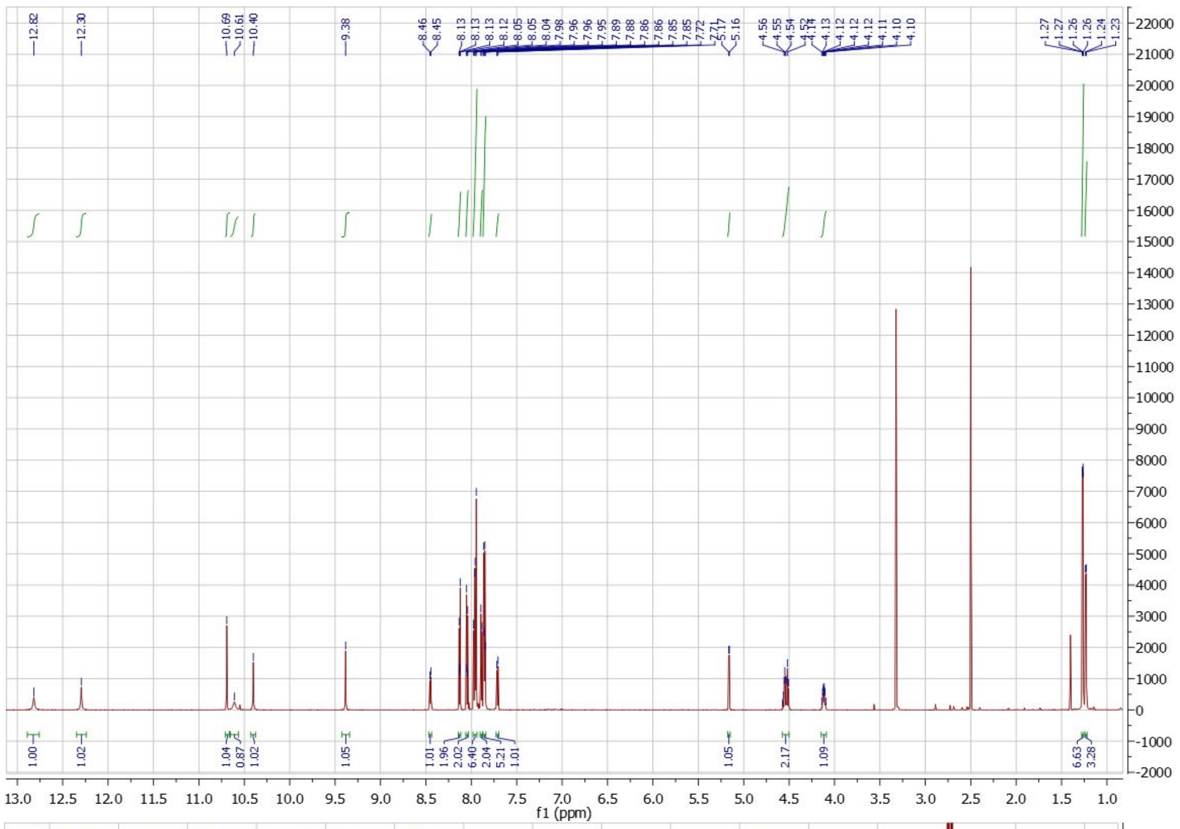

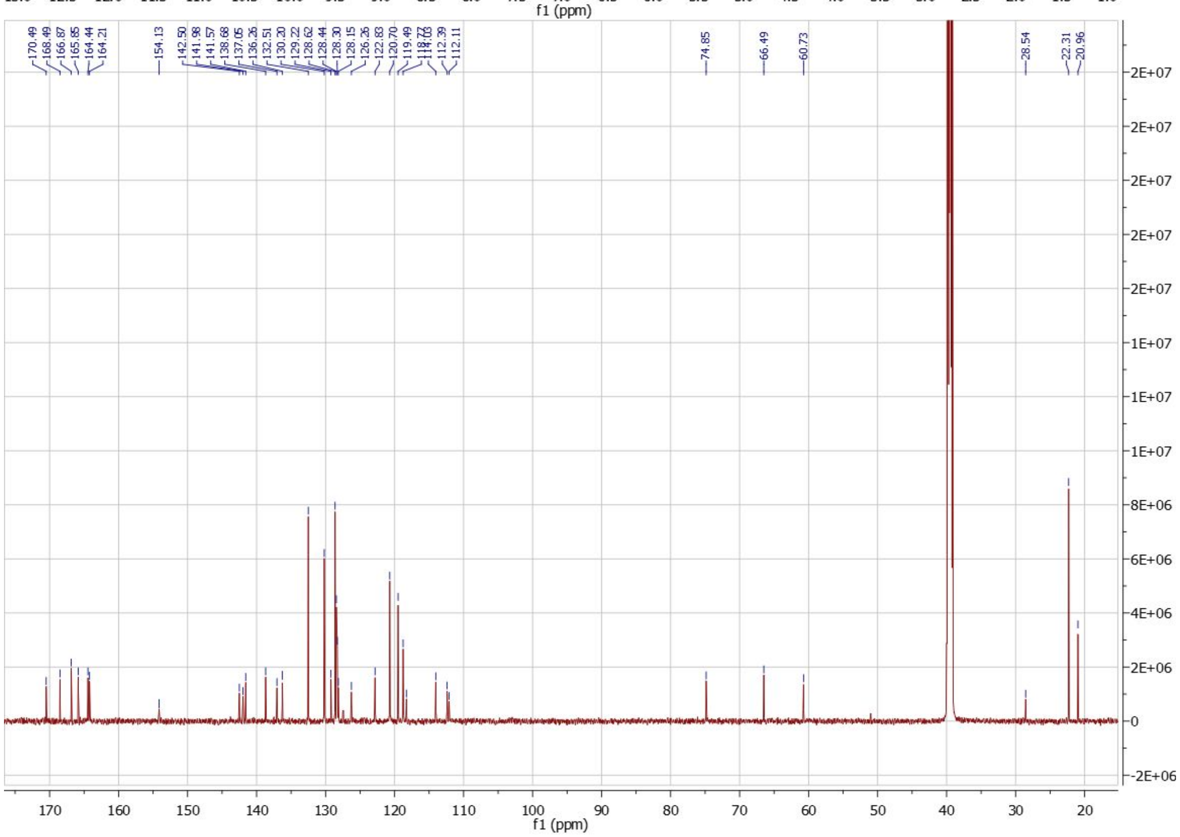

Figure S21. ^{1}H- (top) and ^{13}C-NMR (bottom) spectrum of compound **41**.

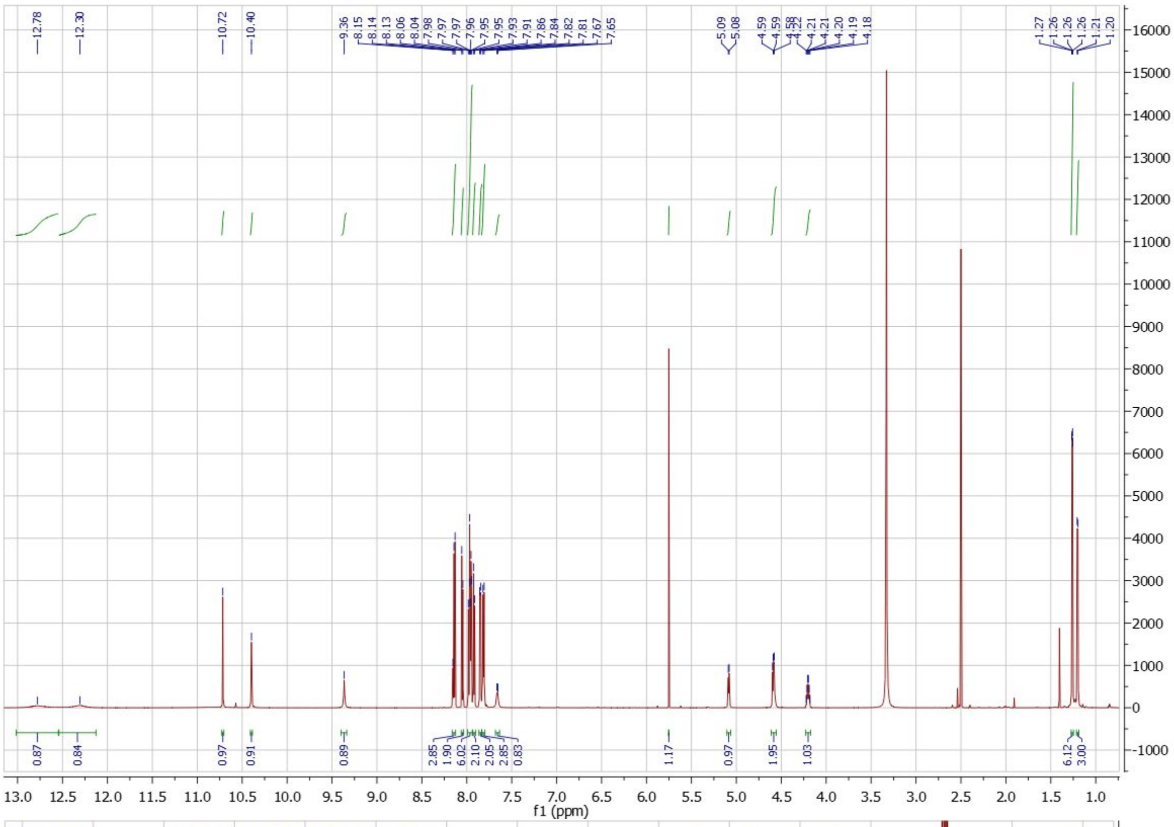

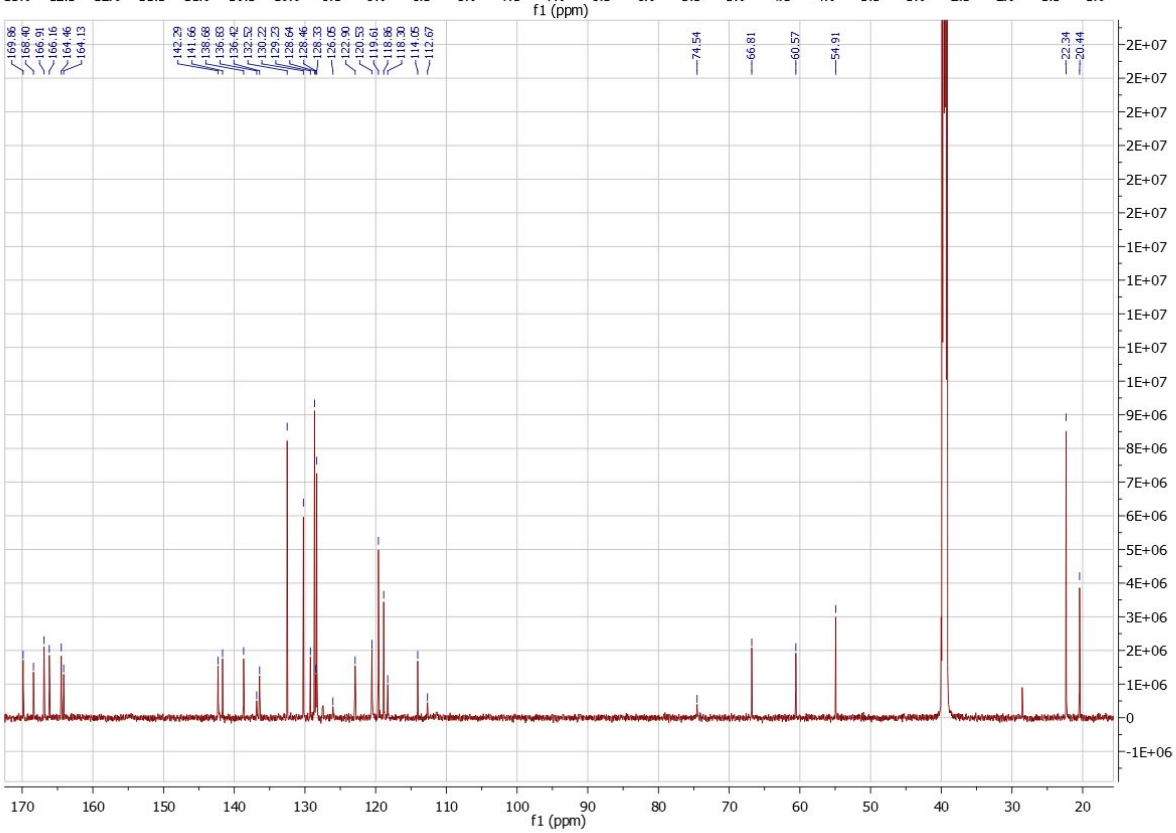

Figure S22. ^{1}H- (top) and ^{13}C-NMR (bottom) spectrum of compound **42**.

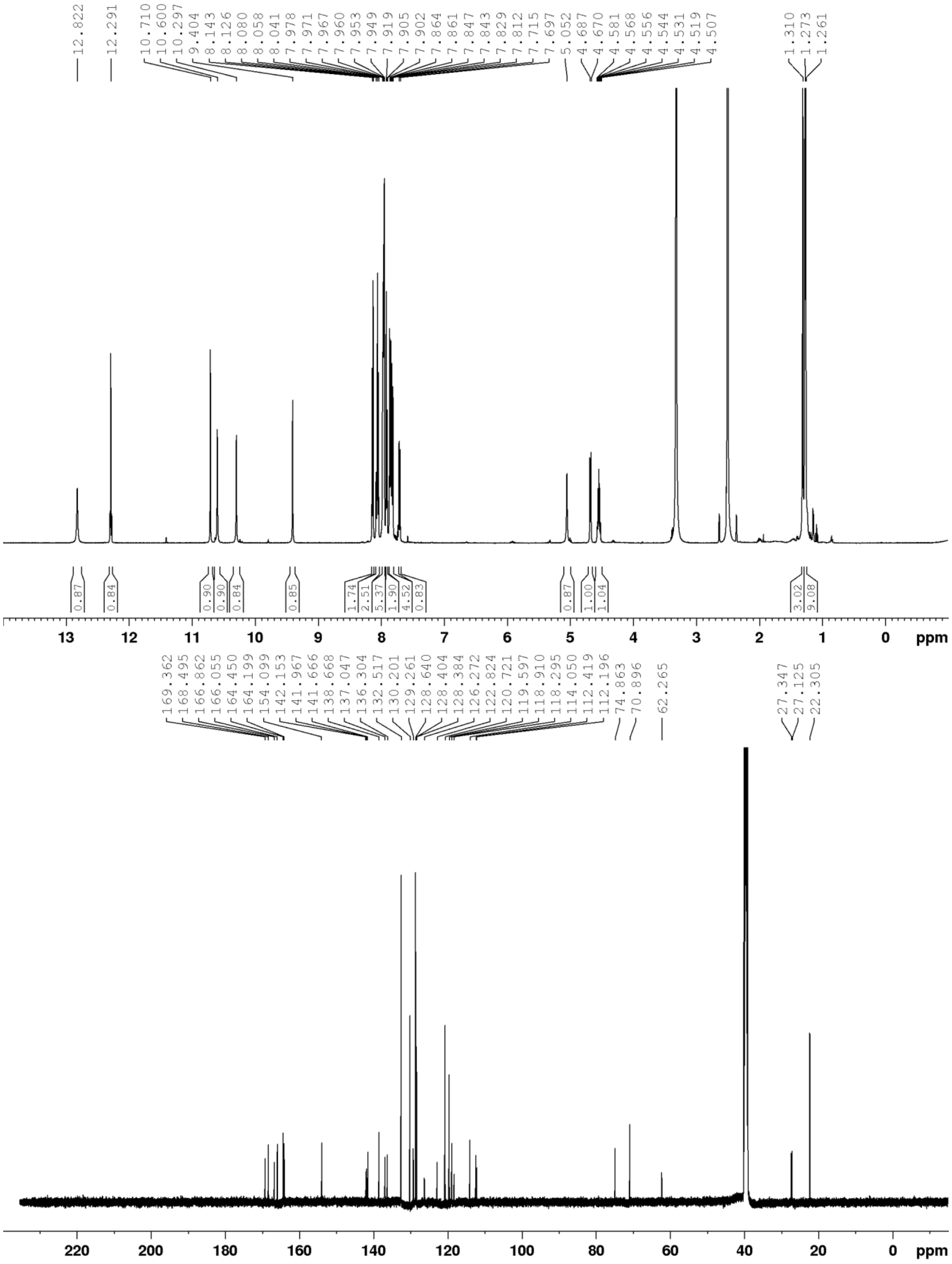

Figure S23. ^{1}H- (top) and ^{13}C-NMR (bottom) spectrum of compound **43**.

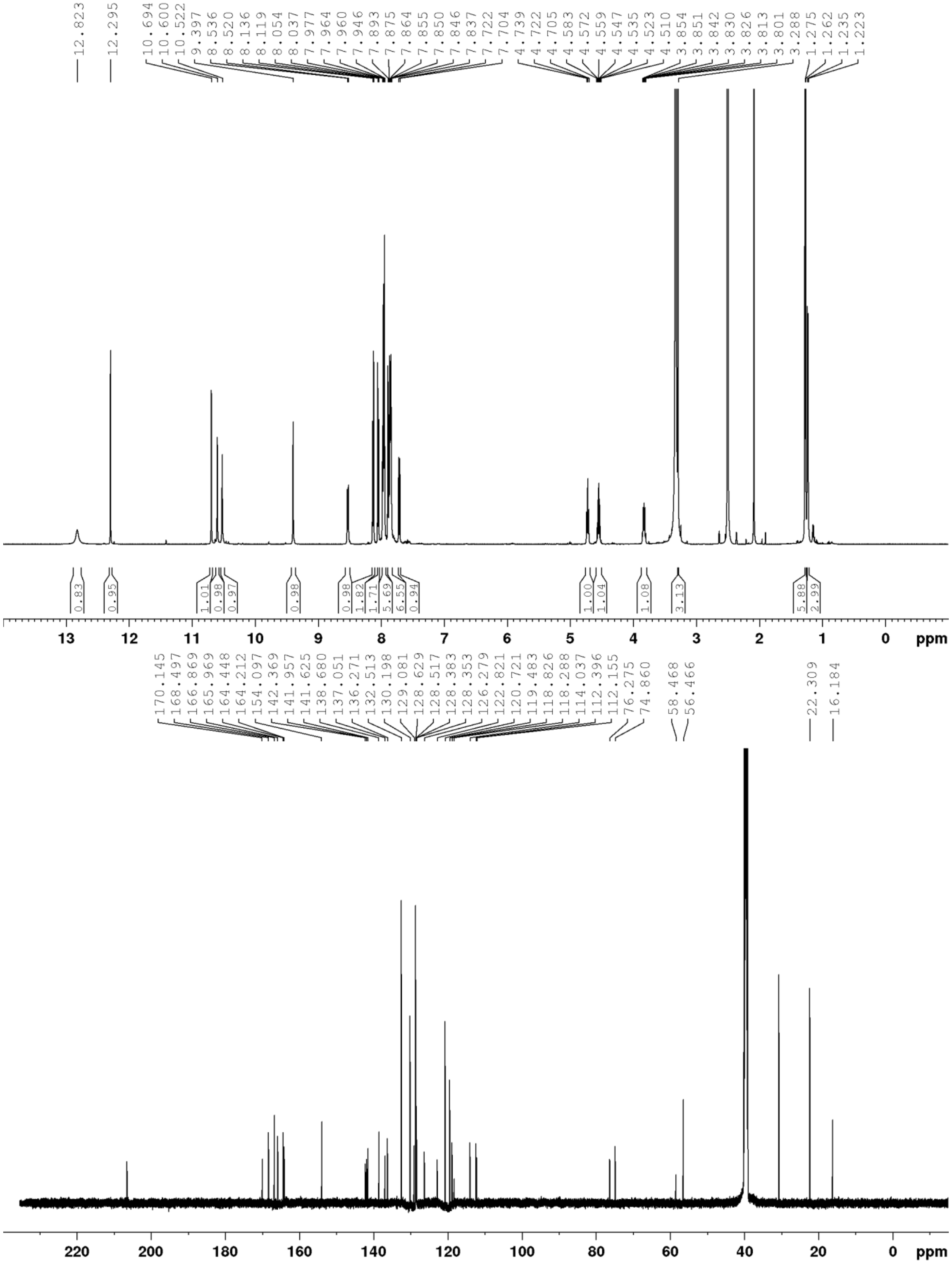

Figure S24. ^{1}H- (top) and ^{13}C-NMR (bottom) spectrum of compound **44**.

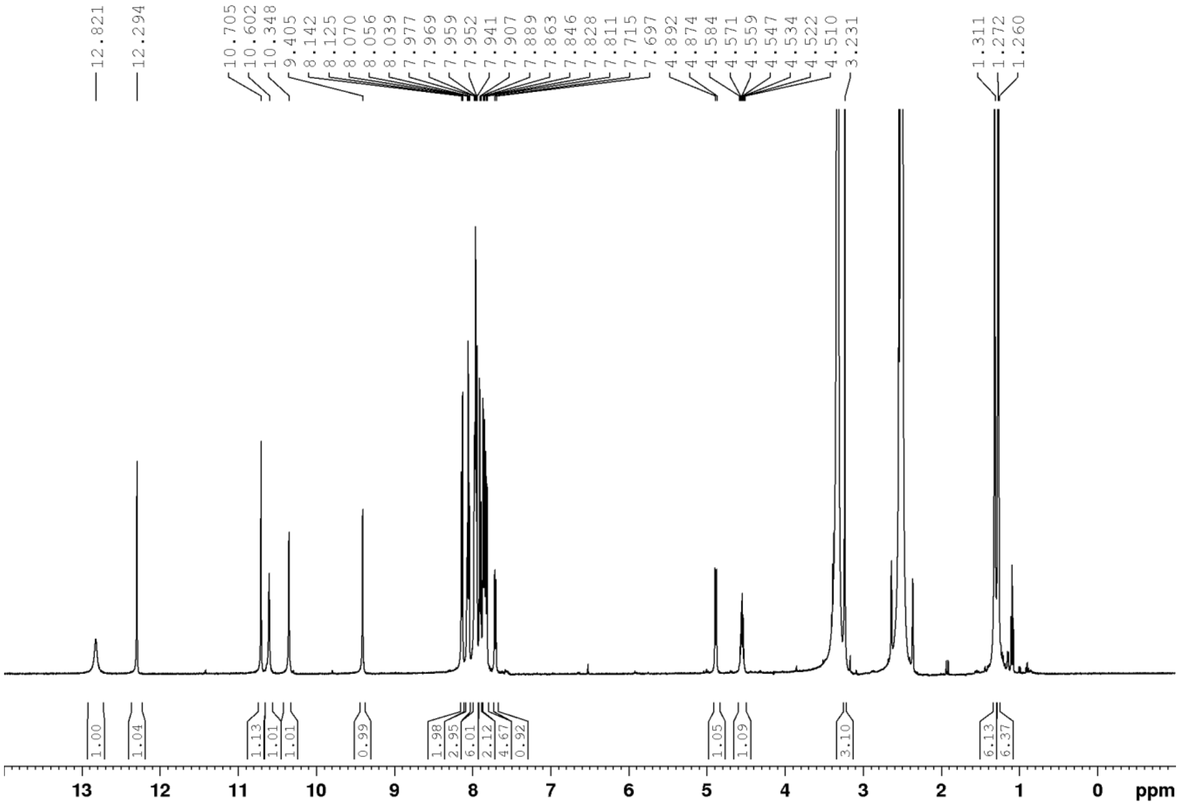

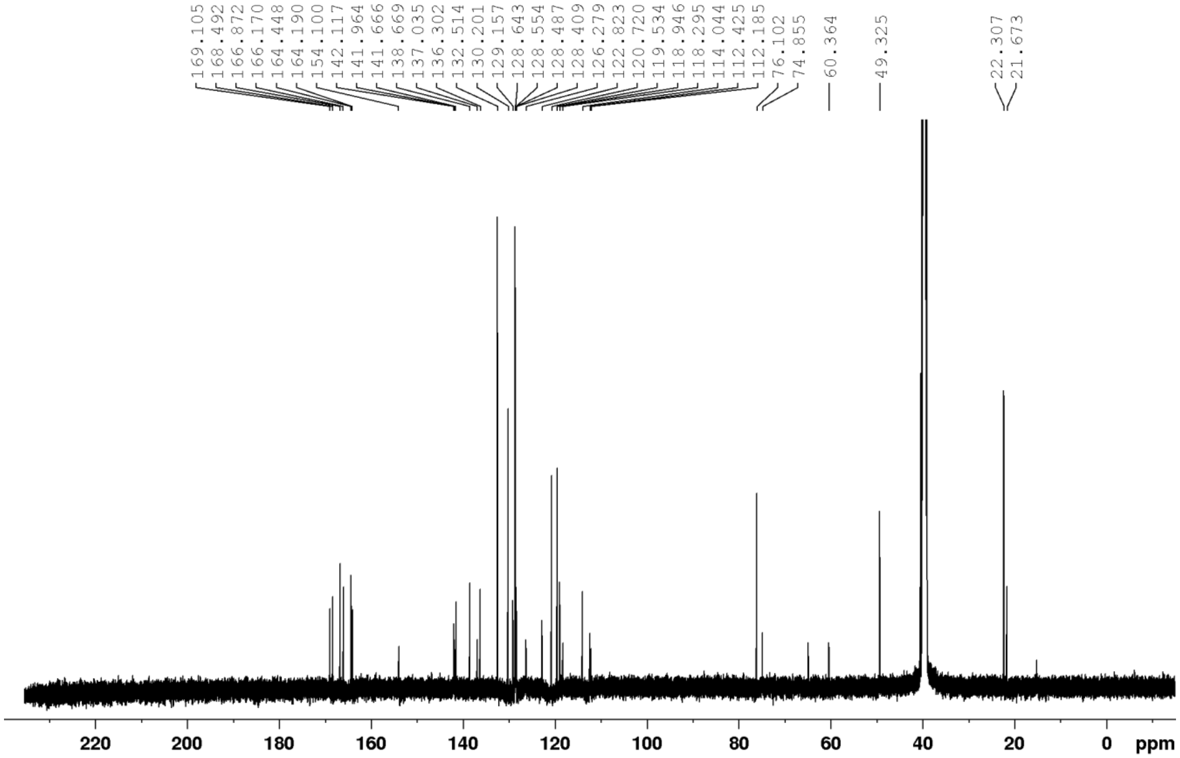

Figure S25. ^{1}H- (top) and ^{13}C-NMR (bottom) spectrum of compound **45**.

HPLC chromatograms

The HPLC chromatograms were determined as described in the manuscript with Phenomenex Aeris PEPTIDE XB-C18, 50 x 2.1 mm, 3.6 µm at a flow rate of 700 µl min^{-1}.

HPLC for compound **13**
Purity: 98.1 %

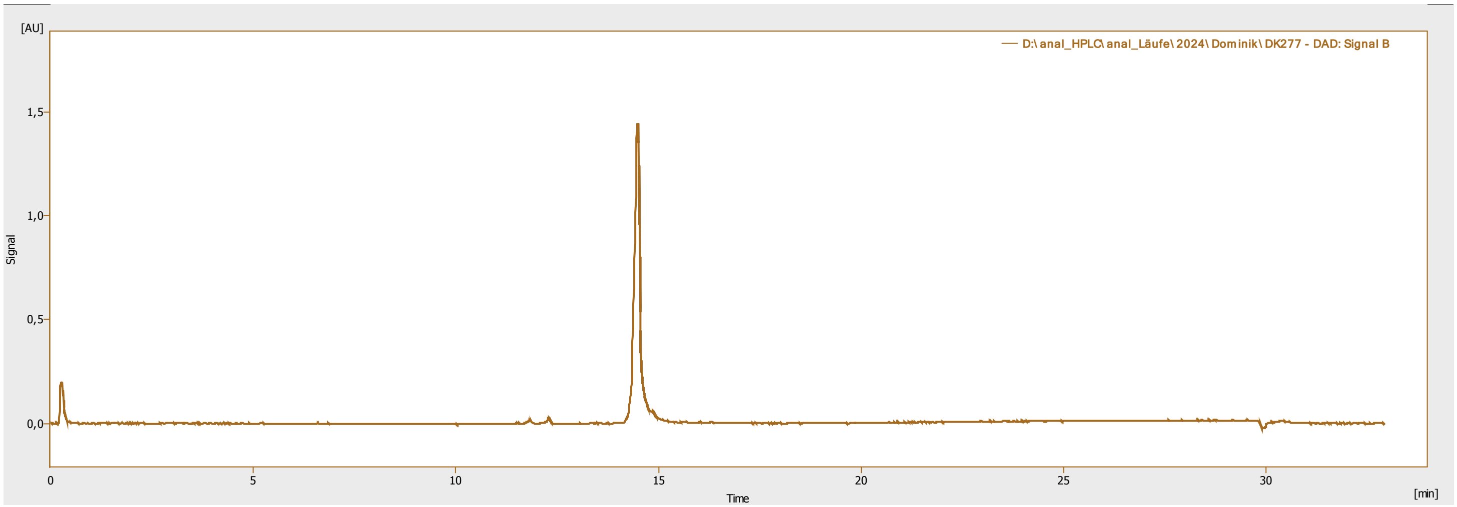

HPLC for compound **17**
Purity: 99.0 %

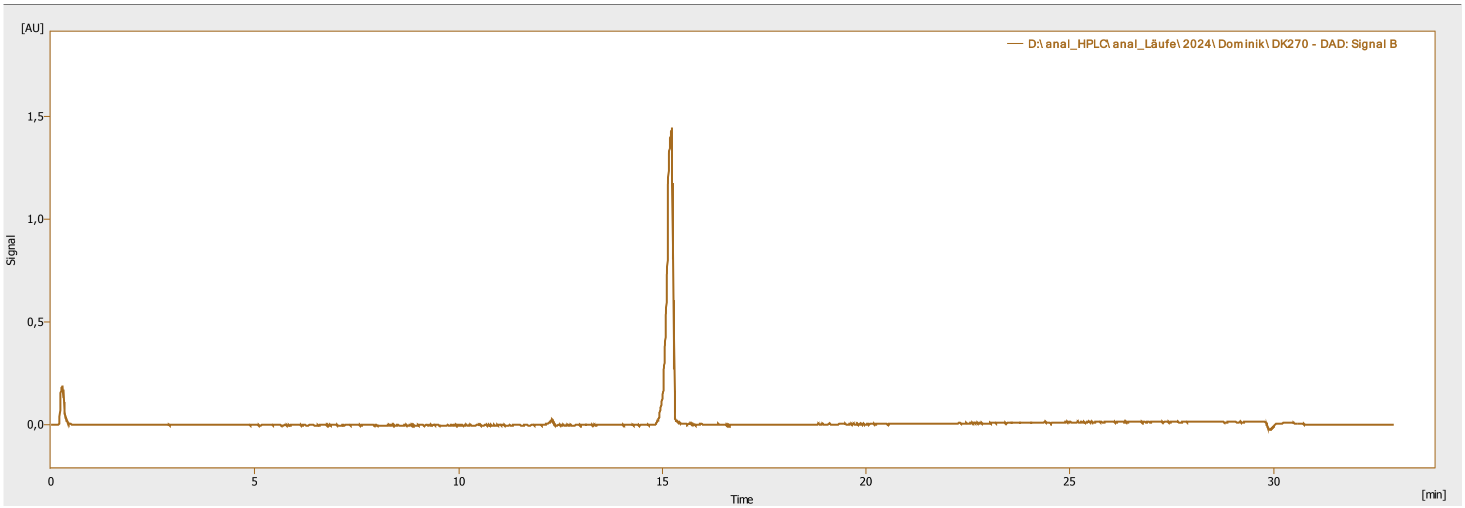

HPLC for compound **21**
Purity: 99.0 %

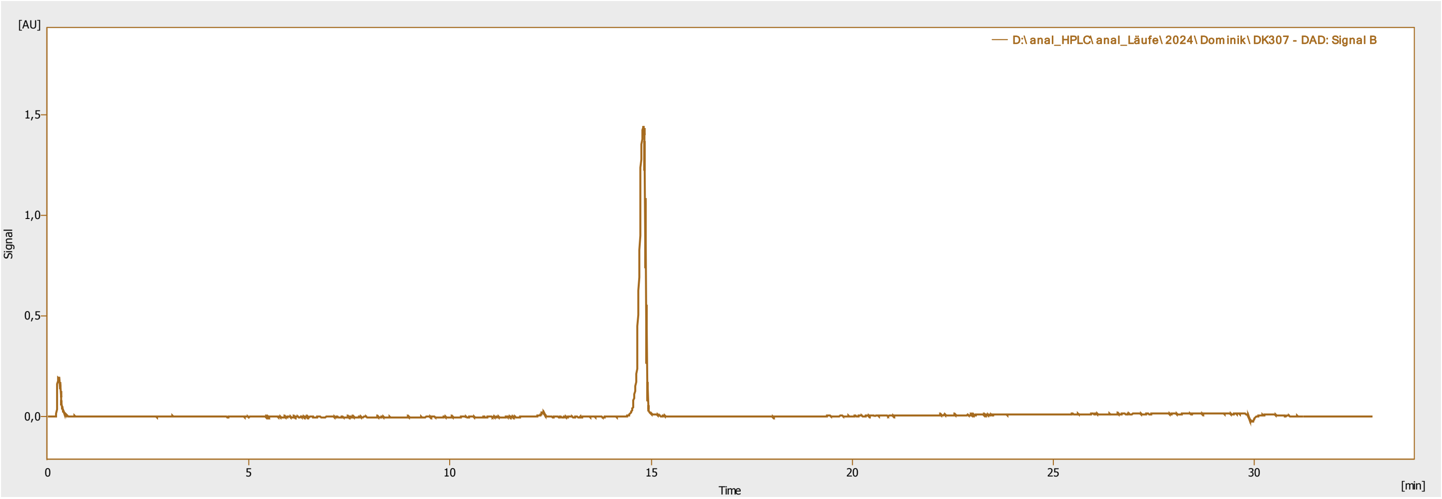

HPLC for compound **38**
Purity: 99.0 %

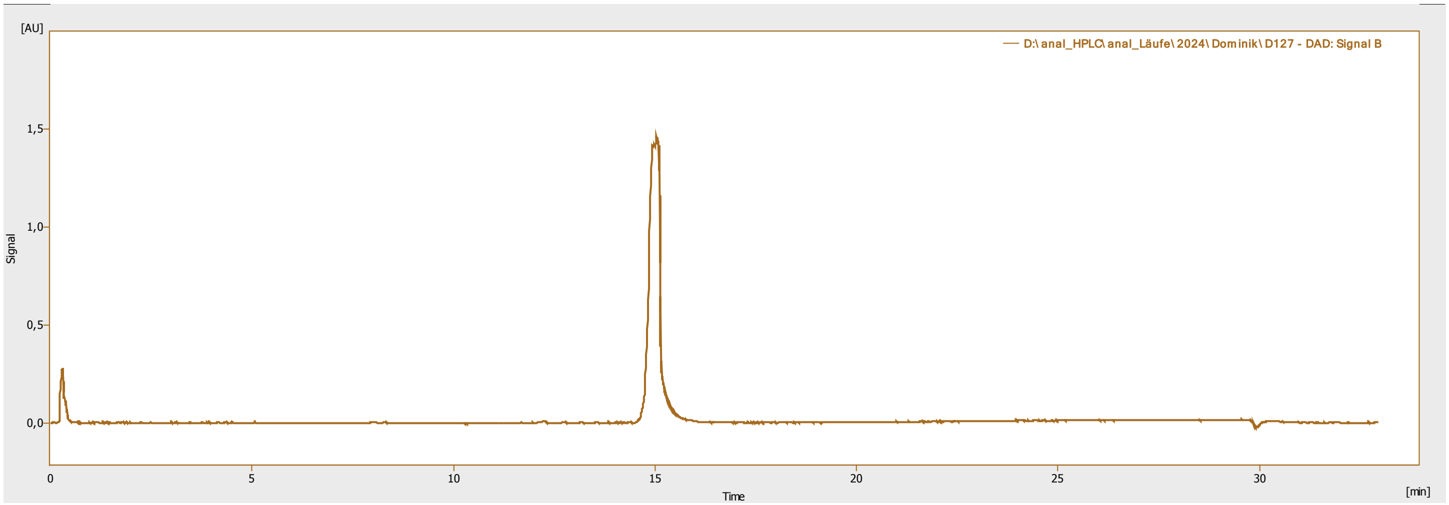

HPLC for compound **42**
Purity: 97.2 %

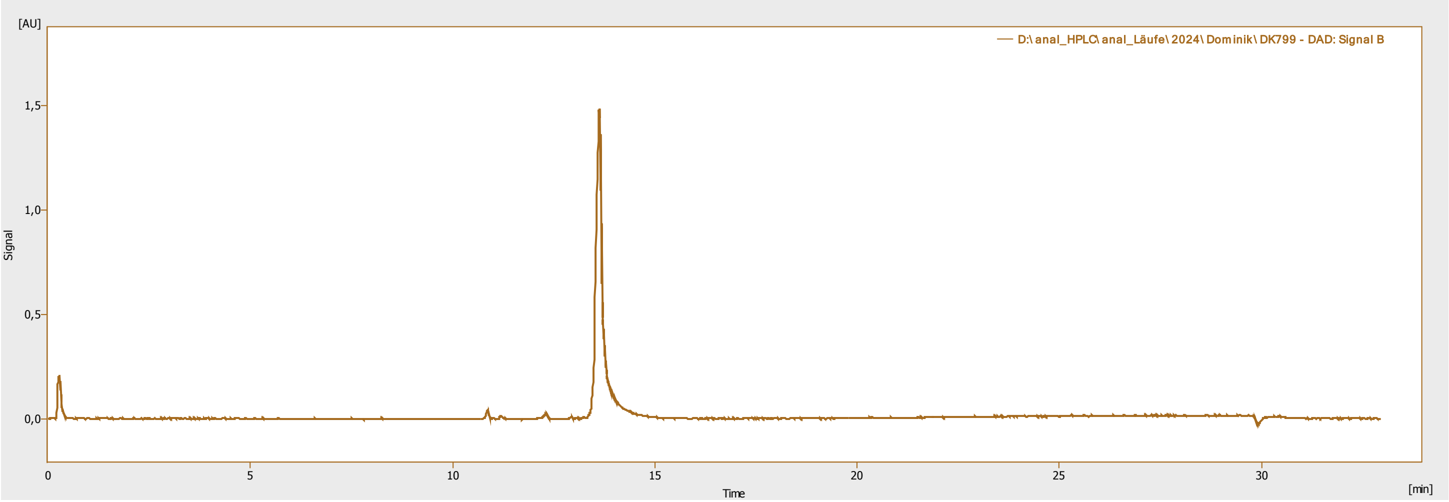

References

(1) Michalczyk, E.; Hommernick, K.; Behroz, I.; Kulike, M.; Pakosz-Stępień, Z.; Mazurek, L.; Seidel, M.; Kunert, M.; Santos, K.; von Moeller, H.; et al. Molecular mechanism of topoisomerase poisoning by the peptide antibiotic albicidin. *Nat. Catal.* **2023**, *6* (1), 52-67. DOI: 10.1038/s41929-022-00904-1.

(2) Friesner, R. A.; Banks, J. L.; Murphy, R. B.; Halgren, T. A.; Klicic, J. J.; Mainz, D. T.; Repasky, M. P.; Knoll, E. H.; Shelley, M.; Perry, J. K.; et al. Glide: A New Approach for Rapid, Accurate Docking and Scoring. 1. Method and Assessment of Docking Accuracy. *J. Med. Chem.* **2004**, *47* (7), 1739-1749. DOI: 10.1021/jm0306430.

(3) Krass, J. D.; Jastorff, B.; Genieser, H. G. Determination of lipophilicity by gradient elution high-performance liquid chromatography. *Anal Chem* **1997**, *69* (13), 2575-2581. DOI: 10.1021/ac961246i.

(4) Baumann, S.; Herrmann, J.; Raju, R.; Steinmetz, H.; Mohr, K. I.; Hüttel, S.; Harmrolfs, K.; Stadler, M.; Müller, R. Cystobactamids: Myxobacterial Topoisomerase Inhibitors Exhibiting Potent Antibacterial Activity. *Angew. Chem. Int. Ed.* **2014**, *53* (52), 14605-14609, https://doi.org/10.1002/anie.201409964. DOI: https://doi.org/10.1002/anie.201409964 (acccessed 2023/07/14).

(5) Wiegand, I.; Hilpert, K.; Hancock, R. E. W. Agar and broth dilution methods to determine the minimal inhibitory concentration (MIC) of antimicrobial substances. *Nat. Protoc.* **2008**, *3* (2), 163-175. DOI: 10.1038/nprot.2007.521.
